# Highly stereoselective nickel-catalyzed difluoroalkylation of aryl ketones to tetrasubstituted monofluoroalkenes and quaternary alkyl difluorides†
†Electronic supplementary information (ESI) available. CCDC 1565189 and 1880997. For ESI and crystallographic data in CIF or other electronic format see DOI: 10.1039/c9sc02806d


**DOI:** 10.1039/c9sc02806d

**Published:** 2019-08-20

**Authors:** Chao Li, Yi-Xuan Cao, Ruo-Xing Jin, Kang-Jie Bian, Zi-Yang Qin, Quan Lan, Xi-Sheng Wang

**Affiliations:** a Hefei National Laboratory for Physical Sciences at the Microscale , Department of Chemistry , Center for Excellence in Molecular Synthesis of CAS , University of Science and Technology of China , Hefei , Anhui 230026 , China . Email: xswang77@ustc.edu.cn

## Abstract

A nickel-catalyzed difluoroalkylation of α-C–H bonds of aryl ketones to furnish highly stereo-defined tetrasubstituted monofluoroalkenes or quaternary alkyl difluorides from secondary or tertiary ketones, respectively, has been established. Mechanistic investigations indicated that these C–H fluoroalkylations proceed *via* a Ni(i)/Ni(iii) catalytic cycle. An obvious fluorine effect was observed in the reaction, and this reaction has demonstrated high stereoselectivity, mild conditions, and broad substrate scopes, thus enabling the late-stage fluoroalkylation of bioactive molecules. This method offers a solution for expedient construction of monofluoroalkenes from readily available materials, and provides an efficient approach for the synthesis of bioactive fluorinated compounds for the discovery of lead compounds in medicinal chemistry.

## Abstract 2

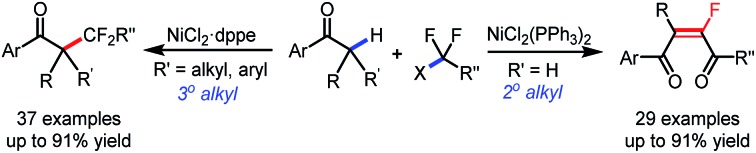
A nickel-catalyzed difluoroalkylation of aryl ketones to furnish highly stereo-defined tetrasubstituted monofluoroalkenes or quaternary alkyl difluorides has been established.

## Introduction

**Scheme 1:**
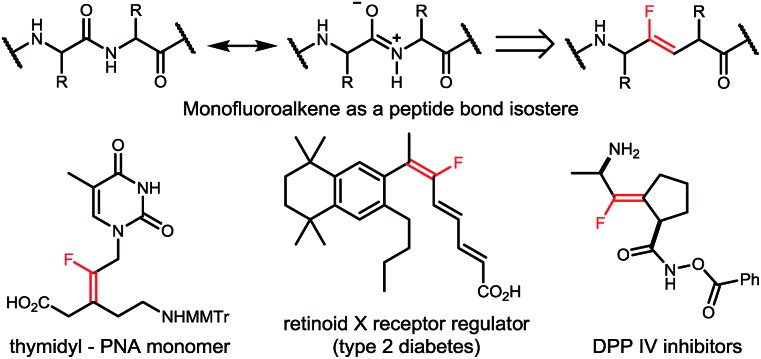
Biologically active molecules containing stereodefined monofluoroalkenes.

Organofluorine compounds have been widely used in pharmaceuticals, agrochemicals, and special materials, due to their unique chemical and physical properties brought by the selective incorporation of fluorine atom(s) or fluorinated moieties into organic molecules.^1^ For example, as an ideal peptide bond isostere in medicinal chemistry, monofluoroalkene exists widely in a great number of biologically active molecules with different pharmacological activities (Scheme 1).^2^ Moreover, monofluorinated olefins have recently drawn ever-increasing attention considering their potential application in materials science,^3^ and their capability as synthons for facile synthesis of fluorinated compounds in organic synthesis.^4^ Accordingly, various classical olefin-construction strategies, including Wittig,^5^ Julia,^6^ Horner–Wadsworth–Emmons^7^ and Peterson^8^ reactions, have been applied to synthesize monofluoroalkenes,^9^ but complete stereoselective control remains a big challenge, especially for the construction of tetrasubstituted monofluorinated olefins. Meanwhile, the multistep preparation of starting materials will inevitably affect the atom- and step-economy of the transformation, thus hampering their application in further organic derivations. Therefore, the development of a facile method for general and selective synthesis of tetrasubstituted monofluoroalkenes is still highly desirable.

**Scheme 2:**
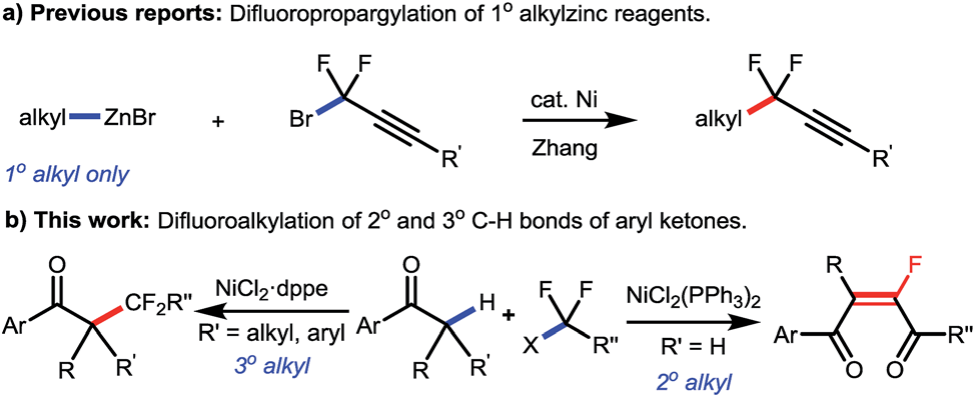
Nickel-catalyzed difluoroalkylation of alkyl reagents.

Transition-metal-catalyzed fluoroalkylation has long been realized as an expedient and efficient strategy to incorporate fluorine into organic molecules.^10^ Due to the ready availability, low cost, low or no toxicity, and unique catalytic characteristics, the first-row transition metals, including Ni, Co, Fe, *etc.*, have recently been widely used in fluoroalkylation of various organic compounds.^11^ In particular, as an economic alternative to palladium and copper catalysts, nickel is more nucleophilic and the oxidation of low-valent nickel species (Ni(0) or Ni(i)) prefers a single electron transfer process, thus offering an ideal solution to fluoroalkylation when relatively “harder” fluoroalkyl halides are used as the coupling partners.^12^ While various synthesis methods for fluoroalkylated arenes, alkenes and alkynes have been well established, nickel-catalyzed fluoroalkylation for selective construction of C(sp^3^)–CF_2_R bonds on the alkyl chain still remains a major problem,^13^ and the only example was limited to the manipulation of the primary alkylzinc species by the Zhang group (Scheme 2).^14^ Moreover, as the known methods to synthesize tetrasubstituted monofluoroalkenes were still hampered by the requirement of prefunctionalized substrates and/or poor stereocontrol,^15^ the stereoselective synthesis of tetrasubstituted monofluoroalkenes from readily available reagents still remains a key issue to be resolved.

Herein, we reported a nickel-catalyzed difluoroalkylation of secondary and tertiary C–H bonds in aryl ketones with fluoroalkyl halides, which furnished tetrasubstituted monofluoroalkenes and quaternary alkyl difluorides, respectively. This reaction has demonstrated high reactivity, broad scopes and mild conditions, thus enabling the late-stage fluorine-containing modification of bioactive molecules. This method offers a solution for expedient construction of monofluoroalkenes from readily available materials, and provides an efficient approach for the synthesis of bioactive fluorinated compounds for the discovery of lead compounds in medicinal chemistry.

## Results and discussion

Our study commenced with 1,2-diphenylethan-1-one (**1a**) as the pilot substrate in the presence of a catalytic amount of [NiCl_2_·(PPh_3_)_2_] (10 mol%) and XantPhos (10 mol%) in THF at –10 °C. When 2-bromo-2,2-difluoroacetate (**2a**) was used as the fluoroalkylating reagent, to our delight, the monofluoroalkene (**3a**) was obtained smoothly in 85% yield, albeit with a relatively lower *E*/*Z* selectivity (4/1). Considering that the size of the R group in fluoroalkylating reagents has an obvious effect on the *E*/*Z* ratio of produced alkene, to improve the *E*/*Z* selectivity, difluoroacetamides (**2b–2c**) were next tested in our catalytic system. As expected, *N*,*N′*-diethyl-2,2-difluoroacetamide (**2b**) gave monofluoroalkene **3b** in a higher *E*/*Z* ratio (7/1), and more bigger *N*,*N′*-diphenyl-2,2-difluoroacetamide (**2c**) afforded the desired **3c** with an excellent *E*/*Z* selectivity in high yield (91% yield, > 99/1 *E*/*Z*). The structure of *E*-isomer **3c** was confirmed by X-ray single crystal diffraction.^19^ In full compliance with the experimental data of base screening, the replacement of LDA with other inorganic bases indicated that ^*t*^BuOK quenched the reaction completely and LiHMDS and KHMDS could also promote the reaction albeit in lower yields (59% and 44%, entries 4–6), which clearly demonstrated that LDA plays an important role in the catalytic cycle. Next, a scrupulous catalyst screening, including different kinds of nickel sources, indicated that [NiCl_2_·(PPh_3_)_2_] was still the optimal catalyst (Table 1, entries 7–10, for more details, see Table S3 in the ESI†). While the addition of exogenous phosphines provided higher yields of the product, a broad ligand screening, including a great variety of phosphine, nitrogen and carbene ligands, has also been carried out (Table 1, entries 11–16, see also Table S4†). Of note is that XantPhos was still the best choice of ligand, furnishing the desired product **3c** with excellent yield and *E* : *Z* ratio (Table 1, entry 3). To our interest, this difluoroalkylation of secondary aryl ketones proceeded at a low temperature (–10 °C). The examination of reaction temperature showed that an even lower temperature of –30 °C still afforded the products with high yield, but higher temperature (0 °C) resulted in a remarkable reduction (Table 1, entries 17–18). Finally, the control experiment in the absence of [NiCl_2_·(PPh_3_)_2_] afforded none of the monofluoroalkene **3c** (Table 1, entry 19).

**Table 1:** Nickel-catalyzed arylfluoroalkylation: optimization of conditions^*a*^

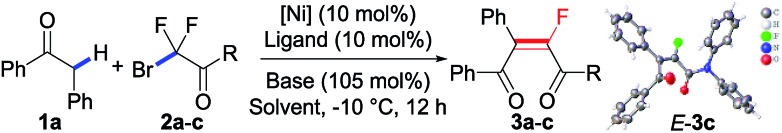					
Entry	Ni source	Ligand	Base	*E*/*Z*^*b*^	Yield^*c*^ (%)
1^*d*^	NiCl_2_(PPh_3_)_2_	XantPhos	LDA	4 : 1	85
2^*e*^	NiCl_2_(PPh_3_)_2_	XantPhos	LDA	7 : 1	72
3	NiCl_2_(PPh_3_)_2_	XantPhos	LDA	>99 : 1	91
4	NiCl_2_(PPh_3_)_2_	XantPhos	^*t*^BuOK	—	Trace
5	NiCl_2_(PPh_3_)_2_	XantPhos	LiHMDS	>99 : 1	59
6	NiCl_2_(PPh_3_)_2_	XantPhos	KHMDS	>99 : 1	44
7	NiCl_2_	XantPhos	LDA	>99 : 1	45
8	Ni(OTf)_2_	XantPhos	LDA	>99 : 1	14
9	NiCl_2_·glyme	XantPhos	LDA	>99 : 1	61
10	Ni(COD)_2_	XantPhos	LDA	>99 : 1	85
11	NiCl_2_(PPh_3_)_2_	PPh_3_	LDA	>99 : 1	38
12	NiCl_2_(PPh_3_)_2_	P(1-Naph)_3_	LDA	>99 : 1	32
13	NiCl_2_(PPh_3_)_2_	dppBz	LDA	>99 : 1	32
14	NiCl_2_(PPh_3_)_2_	Phen	LDA	>99 : 1	36
15	NiCl_2_(PPh_3_)_2_	dmbPy	LDA	>99 : 1	20
16	NiCl_2_(PPh_3_)_2_	IPr·HCl	LDA	>99 : 1	18
17^*f*^	NiCl_2_(PPh_3_)_2_	XantPhos	LDA	>99 : 1	91
18^*g*^	NiCl_2_(PPh_3_)_2_	XantPhos	LDA	>99 : 1	62
19	—	XantPhos	LDA	—	Trace

^*a*^Unless otherwise noted, the reaction conditions were as follows: **1a** (0.2 mmol), **2** (3.0 equiv.), [Ni] (10 mol%), ligand (10 mol%), base (105 mol%), solvent (2.0 mL), –10 °C, 12 h, N_2_.

^*b*^ *E*/*Z* ratio was determined by ^19^F NMR analysis.

^*c*^Yields of the isolated products given.

^*d*^BrCF_2_CO_2_Et was used as **2a**.

^*e*^BrCF_2_CONEt_2_ was used as **2b**.

^*f*^ *T* = –30 °C.

^*g*^ *T* = 0 °C. XantPhos = 4,5-bis(diphenylphosphino)-9,9-dimethylxanthene, dmbPy = 4,4′-dimethyl-2,2′-bipyridine, dppBz = 1,2-bis(diphenylphosphino)benzene, Phen = 1,10-phenanthroline.

With the optimized conditions in hand, we next started to investigate the substrate scope of this nickel-catalyzed sequential fluoroalkylation/defluorination reaction. As shown in Table 2, a great number of secondary C–H bonds on different kinds of aryl ketones were fluoroalkenylated successfully with high stereoselectivity and fluorinated tetrasubstituted olefins were obtained. The substituent effects of the both aryl rings were first examined. A variety of secondary aryl ketones **1** with *para*-, *meta*-, as well as *ortho*-substituents on both aryl rings were smoothly fluoroalkenylated to afford the corresponding monofluoroalkenes with high *E*-selectivity (>99/1). Both electron-donating groups, including Me (**3d**, **3q**) and OMe (**3e–3g**, **3l–3n**, **3o**), and electron-withdrawing groups such as F (**3x**, **3z**), Cl (**3s**, **3w**), Br (**3t**, **3v**) and CF_3_ (**3y**), on the phenyl rings were well compatible with the standard conditions. Of note is that the bromo substituent, as well as relatively inactive halides including chloro and fluoro atoms on the aryl rings were tolerant, offering the foreseeable potential for further synthetic elaboration of monofluoroalkenes. To our satisfaction, not just acyclic ketones, cyclic ketones (**3aa–3ad**) could also undergo the process smoothly under this catalytic system, albeit in a slightly lower yield.

**Table 2:** Nickel-catalyzed difluoroalkylation of 2° aryl ketones to monofluoroalkenes^*a*^

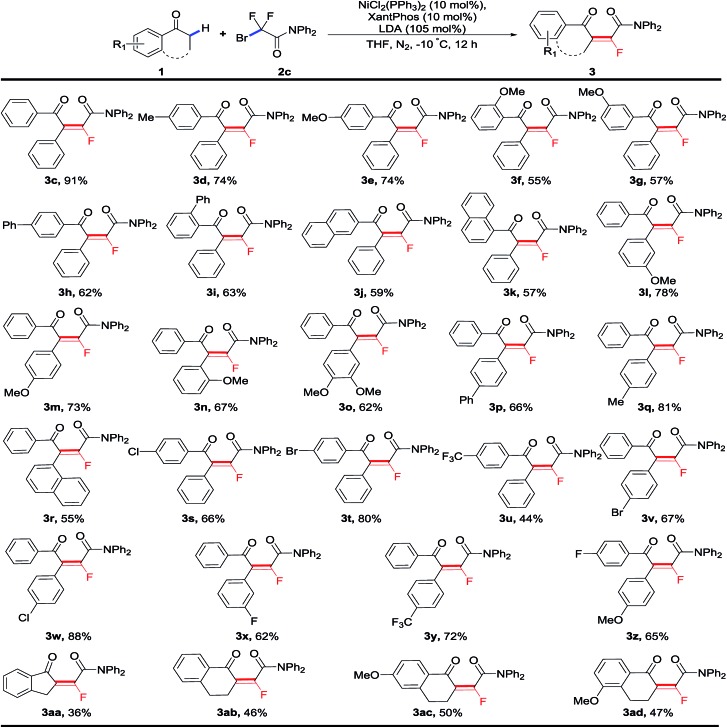

^*a*^Reaction conditions: **1** (0.2 mmol, 1.0 equiv.), **2** (0.6 mmol, 3.0 equiv.), NiCl_2_(PPh_3_)_2_ (10 mol%), XantPhos (10 mol%), LDA (105 mol%), THF (2.0 mL), –10 °C, 12 h, under a N_2_ atmosphere. *E*/*Z* > 99 : 1. XantPhos = 4,5-bis(diphenylphosphino)-9,9-dimethylxanthene.

After the nickel-catalyzed fluoroalkenylation had been successfully established, we next set out to explore the direct difluoroalkylation of 3 °C–H bonds in tertiary aryl ketones using the same strategy, where with the absence of active atoms in the fluoroalkylated aryl ketones, the following defluorination was inhibited. To construct such quaternary alkyl difluorides, the reoptimization of the reaction conditions with 1,2-diphenylpropan-1-one **4a** as the pilot substrate indicated that extra addition of 0.2 equiv. of ZnCl_2_ could increase the yield remarkably (for details, see Tables S14 and S15 in the ESI†).^16^ As shown in Table 3, a number of cyclic and acyclic aryl ketones were difluoroalkylated successfully, delivering the desired products **5** with difluoroalkylated quaternary carbon centers in good to excellent yields. Importantly, cyclic and acyclic aryl ketones bearing both electron-donating and electron-withdrawing groups on the phenyl rings were well tolerated in this catalytic reaction. Remarkably, the investigation of α-substituents (R_1_) of cyclic aryl ketones showed that various alkyl groups like Me (**5q**, **5s**), *n*-Bu (**5u**), and Bn (**5t**), were compatible with this nickel-catalyzed direct fluoroalkylation. Additionally, both five- and six-membered rings were suitable substrates in this transformation. In view of the fact that *gem*-difluoromethyl groups (CF_2_) acted as key motifs to improve the bioactivity of candidate drug molecules in medicinal chemistry, the examination of various fluoroalkylating reagents, including difluoromethylated heteroarene **5ae** and aromatic arene reagents **5af** and diverse bromodifluoroacetamides (**5ag–5ai**), indicated promising prospects of accessing various fluoroalkylated aryl ketones for drug design and screening.

**Table 3:** Nickel-catalyzed difluoroalkylation of 3° aryl ketones to alkyl difluorides^*a*^

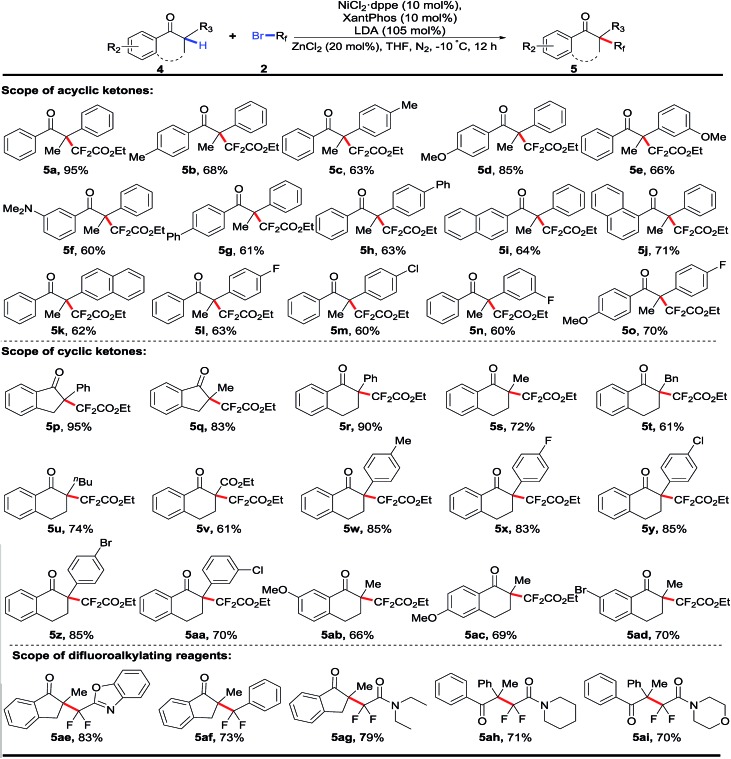

^*a*^Reaction conditions: **4** (0.2 mmol, 1.0 equiv.), **2** (3.0 equiv.), NiCl_2_·dppe (10 mol%), XantPhos (10 mol%), LDA (105 mol%), ZnCl_2_ (0.2 equiv.), THF (2.0 mL), –10 °C, 12 h, under a N_2_ atmosphere. XantPhos = 4,5-bis(diphenylphosphino)-9,9-dimethylxanthene.

**Scheme 3:**
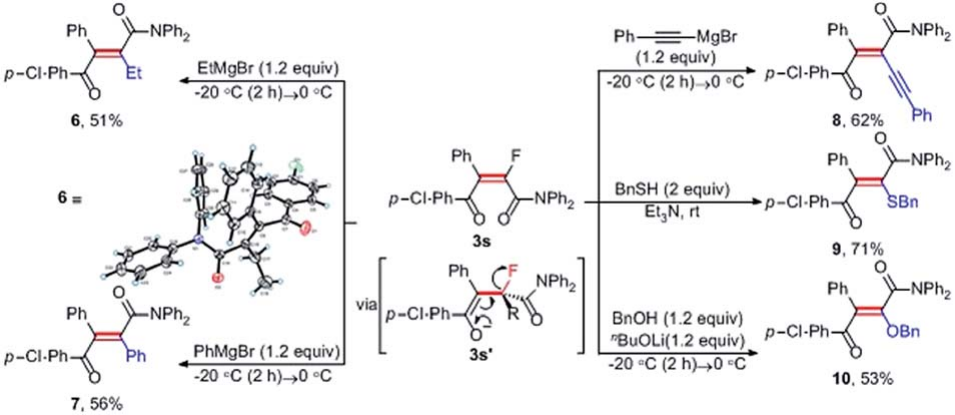
The synthetic application of monofluoroalkenes.

To demonstrate the synthetic potential of this catalytic method, further transformations of monofluorinated alkene **3s***via* nucleophile-promoted defluorination, which enabled the facile synthesis of tetrasubstituted alkenes, were studied. To our delight, as shown in Scheme 3, the treatment of **3s** with 1.2 equiv. of EtMgBr proceeded smoothly, affording the ethylated alkene **6** in 51% yield. The X-ray crystal structure of olefin **6** (ref. 19) unambiguously established the geometry of this all-carbon double bond, which was formed through defluorination of enol **3s′** to deliver the thermodynamically stabilized alkene. By using such a defluorination protocol, monofluoroalkene **3s** could also be transformed into arylated and alkynylated olefins (**7**, **8**), and heteroatom-substituted olefins (**9**, **10**) in good yields, respectively. As a vital structural motif existing in various functionalized molecules, stereodefined tetrasubstituted olefins have been widely explored for their potential application in molecular devices and liquid crystals, and used as key synthons in total synthesis of natural products and complexity-generating synthesis.^3^,^4^

**Scheme 4:**
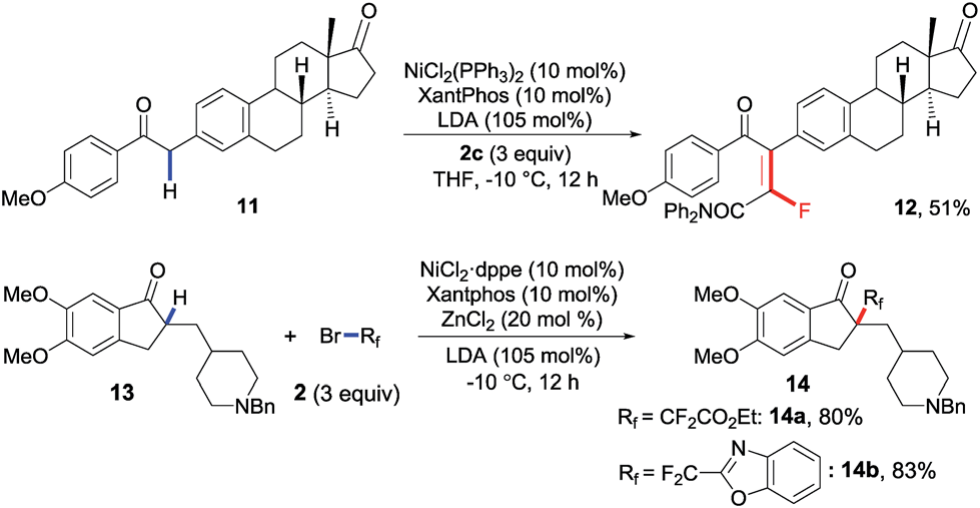
Late-stage modification of biologically active complex molecules.

Considering the good functional group tolerance and mild conditions revealed in these nickel-catalyzed reactions, the application prospects of both transformations were further demonstrated *via* late-stage fluoroalkylation of secondary and tertiary C–H bonds in biologically active complex molecules. As shown in Scheme 4, estrone derivative **11** was smoothly monofluoroalkenylated in 51% yield, and the fluorinated multifunctional compound **12** enabled the facile synthesis of more complex (non-)fluorinated derivatives *via* diverse transformations. Meanwhile, donepezil,^17^ known as an acetylcholinesterase inhibitor for Alzheimer's disease, could also be difluoroalkylated successfully with good yields, in which ester (**14a**) and benzo[*d*]oxazole (**14b**) on the fluoroalkylating reagents were well tolerated. All these late-stage modifications of complex molecules consistently proved that this newly developed catalytic system offered an efficient method for expedient synthesis of tetrasubstituted monofluoroalkenes and quaternary alkyl difluorides.

**Scheme 5:**
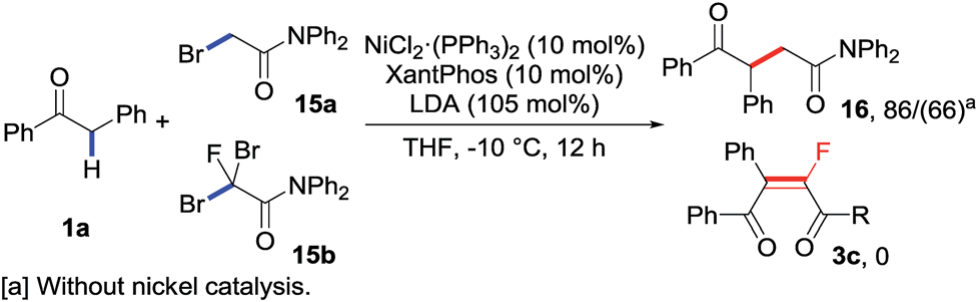
Fluorine effect.

As an extra advantage of this catalytic transformation, our control experiments further confirmed a clear fluorine effect.5*g* As shown in Scheme 5, the subjection of non-halogenated primary bromide **15a** to the standard conditions with or without the addition of the nickel catalyst could result in product **16** as expected, albeit with a relatively lower yield for the latter case. Compared with the control experiment using fluorinated reagent **2c** (entry 19, Table 1), in which none of the desired product **3c** was obtained, such results clearly demonstrated that the difluoroalkylating reagents exhibited totally different reactivity from their non-fluorinated analogues. Indeed, a similar analogue **15b**, in which only a fluorine atom was replaced by bromine, affords none of the desired monofluoroalkene **3c**, even if the bromine group could serve as a better leaving group. These interesting results revealed that the selective introduction of fluorine atom(s) into the substrates may influence the intrinsic reactivity of the substrates, and helped design new reaction patterns following the strategy by using fluorine-containing compounds.

**Scheme 6:**
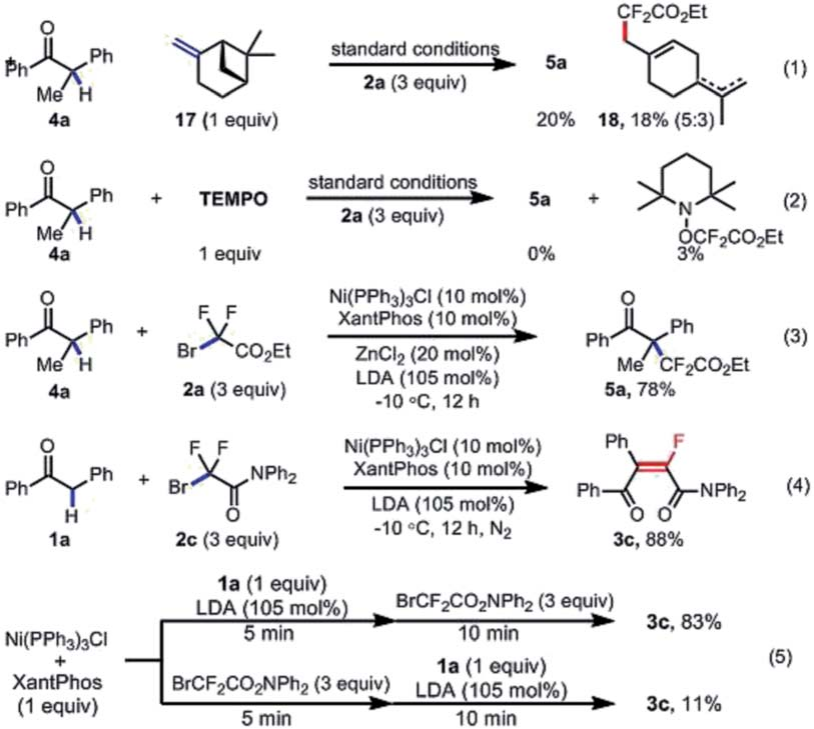
Mechanistic studies.

To gain some insights into the mechanism of this transformation, a series of control experiments were next carried out (Scheme 6). Firstly, the subjection of β-piene to the standard conditions could afford the cycle-opening product **18** in 18% yield along with 20% yield of the desired difluoroalkylated product **5a**. When the radical scavenger TEMPO was used as the additive, the model reactions were completely inhibited, and the TEMPO–CF_2_COOEt was determined by ^19^F NMR analysis (eqn (2)). These results indicated that a difluoroalkyl radical was *in situ* generated and involved in the catalytic cycle. Moreover, the pre-synthesized Ni(i)Cl(PPh_3_)_3_ could give almost the same result as Ni(ii) species used in the reaction system. All these results implied that the difluoroalkyl radical was generated by single-electron-oxidation of Ni(i) with difluoroalkyl bromide **2**, and Ni(i) served as an active catalytic species. Finally, the sequential addition of the enol **1a′**, which is *in situ* generated from the mixture of **1a** (1 equiv.)/LDA (1.05 equiv.), and then fluoroalkylating reagent **2c** (3 equiv.) into the prepared stoichiometric Ni(i) species furnished the fluoroalkenylated product **3c** in a comparable yield in eqn (5), but the reverse order of sequential addition gave only 11% yield of **3c**. These results demonstrated that the nickel-catalyzed single-electron-reduction of fluoroalkyl halides took place after the transmetallation step of Ni(i) species with the enol anion.

**Scheme 7:**
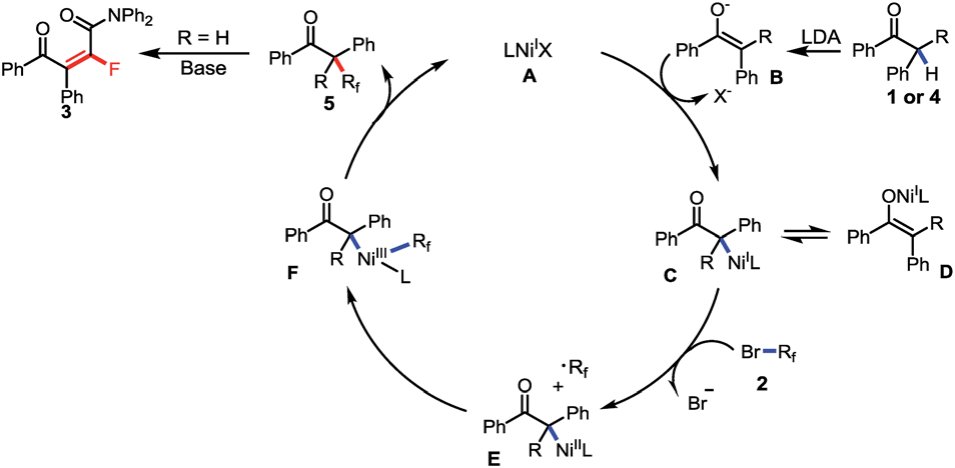
The possible reaction mechanism.

Based on the above mentioned results and the previous reports,^18^ a base-promoted C–H fluoroalkylation *via* a Ni(i)/Ni(iii) catalytic cycle involving a fluoroalkyl radical was proposed. As shown in Scheme 7 (for the generation of Ni(i) species, see ESI Fig. S6†), the transmetallation between Ni(i) catalyst **A** and *in situ* generated enol anion **B** gave the Ni(i) complex **C** and **D**, which furnished the Ni(ii) species **E** and the difluoroalkyl radical *via* a single-electron oxidation by fluoroalkyl bromide **2**. The following radical oxidation of Ni(ii) species **E** afforded Ni(iii) intermediated **F**, followed by reductive elimination resulting in alkyl difluoride **5** when tertiary aryl ketone was used as the substrate (R = aryl or alkyl). Instead, starting from a secondary ketone (R = H), defluorination took place through an E2 elimination process and furnished a tetrafluoroalkylated monofluoroalkene **3** as the final product.

## Conclusions

In summary, we have developed a nickel-catalyzed difluoroalkylation of α-C–H bonds of aryl ketones, which furnished highly stereo-defined tetrasubstituted monofluoroalkenes or quaternary alkyl difluorides from secondary or tertiary ketones. Mechanistic investigations indicated that these C–H fluoroalkylation reactions proceed *via* a Ni(i)/Ni(iii) catalytic cycle involving an *in situ* generated fluoroalkyl radical. An obvious fluorine effect was observed in the reaction, and this novel method has demonstrated high stereoselectivity, mild conditions, broad scope, and synthetic potential for further transformation and late-stage fluoroalkenylation (or fluoroalkylation) of complex molecules. Further exploration of the scope and other useful derivations are still underway in our laboratory.

## Conflicts of interest

There are no conflicts to declare.

## Supplementary Material

Supplementary information

Crystal structure data

## References

[cit1] Müller K., Faeh C., Diederich F. (2007). Science.

[cit2] Levenson A. S., Jordan V. C. (1999). Eur. J. Cancer.

[cit3] Schreivogel A., Maurer J., Winter R., Baro A., Laschat S. (2006). Eur. J. Org. Chem..

[cit4] Zhu Y., Wang Q., Cornwall R. G., Shi Y. (2014). Chem. Rev..

[cit5] Hirai G., Nishizawa E., Kakumoto D., Morita M., Okada M., Hashizume D., Nagashima S., Sodeoka M. (2015). Chem. Lett..

[cit6] Kumar R., Pradhan P., Zacj B. (2011). Chem. Commun..

[cit7] Opekar S., Pohl R., Beran P., Rulíšek L., Beier P. (2014). Chem.–Eur. J..

[cit8] Welch J. T., Lin J. (1996). Tetrahedron.

[cit9] Landelle G., Bergeron M., Turcotte-Savard M.-O., Paquin J.-F. (2001). Chem. Soc. Rev..

[cit10] Ma J.-A., Cahard D. (2004). Chem. Rev..

[cit11] Jiang X., Sakthivel S., Kulbitski K., Nisnevich G., Gandelman M. (2014). J. Am. Chem. Soc..

[cit12] Su B., Cao Z.-C., Shi Z.-J. (2015). Acc. Chem. Res..

[cit13] Aikawa K., Maruyama K., Nitta J., Hashimoto R., Mikami K. (2016). Org. Lett..

[cit14] An L., Xu C., Zhang X. (2017). Nat. Commun..

[cit15] Tsai H.-J. (1996). Tetrahedron Lett..

[cit16] Spielvogel D. J., Buchwald S. L. (2002). J. Am. Chem. Soc..

[cit17] Luo Z., Sheng J., Sun Y., Lu C., Yan J., Liu A., Luo H.-B., Huang L., Li X. (2013). J. Med. Chem..

[cit18] Liu C., Tang S., Liu D., Yuan J., Zheng L., Meng L., Lei A. (2012). Angew. Chem., Int. Ed..

[cit19] The crystallography data have been deposited at the Cambridge Crystallographic Data Center (CCDC) under accession numbers CCDC: 1565189 (**3c**), and CCDC: ; 1880997 (**6**).

