# Supplementary material for: Highly stereoselective nickel-catalyzed difluoroalkylation of aryl ketones to tetrasubstituted monofluoroalkenes and quaternary alkyl difluorides
Source: Chem Sci. 2019 Aug 20;10(40):9285–91. doi: 10.1039/c9sc02806d (PMC7003883; doi:10.1039/c9sc02806d)
Supplement: Supplementary file 1 [file SC-010-C9SC02806D-s001.pdf]

Electronic Supplementary Material (ESI) for Chemical Science.
This journal is © The Royal Society of Chemistry 2019

Supporting Information
For

Highly Stereoselective Nickel-Catalyzed Difluoroalkylation of Aryl Ketones to Tetrasubstituted Monofluoroalkenes and Quaternary Alkyl Difluorides

Chao Li+, Yi-Xuan Cao+, Ruo-Xing Jin, Kang-Jie Bian, Zi-Yang Qin, Quan Lan and Xi-Sheng Wang *

Hefei National Laboratory for Physical Sciences at the Microscale and Department of Chemistry,
Center for Excellence in Molecular Synthesis of CAS,
University of Science and Technology of China,
96 Jinzhai Road, Hefei, Anhui 230026, China

Tabel of Contents

General Information S2
Tables of the Optimization of Reaction Conditions of Monofluoroalkenes S3
Tables of the Optimization of Reaction Conditions of Quaternary Alkyl Difluorides S6
General Procedure for Monofluoroalkenes and Quaternary Alkyl Difluorides S10
Mechanistic Studies S27
References S31
X-Ray crystal data of 3c S32
X-Ray crystal data of 6 S37
^{1}H, ^{13}C, and ^{19}F NMR Spectra S43

General Information:

NMR spectra were recorded on Bruker-400 MHz NMR spectrometer (400 MHz for ^{1}H; 101 MHz for ^{13}C and 376 MHz for ^{19}F {^{1}H, ^{13}C decoupled}). ^{1}H NMR chemical shifts were determined relative to internal $(CH_3)_4Si$ (TMS) at δ 0.0 or at the signal of a residual protonated solvent: $CDCl_3$ δ 7.26. ^{13}C NMR chemical shifts were determined relative to $CDCl_3$ δ 77.16. ^{19}F NMR chemical shifts were determined relative to $CFCl_3$ at δ 0.0. Data for ^{1}H, ^{13}C, ^{19}F NMR are recorded as follows: chemical shift (δ, ppm), multiplicity (s = singlet, d = doublet, t = triplet, m = multiplet, q = quartet, br = broad). High resolution mass spectra were recorded on P-SIMS-Gly of BrukerDaltonics Inc. using ESI-TOF (electrospray ionization-time of flight). THF was distilled from sodium immediately before use. LDA was obtained from Energy-chemical used as received (2 mol/L). $NiCl_2(PPh_3)_2$ was obtained from Energy-chemical and used as received. XantPhos was obtained from aladdin and used as received. $ZnCl_2$ was evacuated and heated under oil bath of 150 °C for 8 h before use. $BrCF_2CO_2Et$ was obtained from Fluorochem Ltd (UK) (J&K purchased) and used as received.

Tables of the Optimization of Reaction Conditions of Monofluoroalkenes

Table S1. Fluoroalkylating Reagents Screening[a]

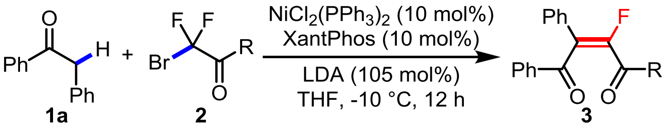

Entry	R	Yield (%)	*E*/*Z*[b]
1	OEt, **2a**	85	4:1
2	NEt_2, **2b**	72	7:1
3	NPh_2, **2c**	91	>99:1

[a] Reaction conditions: **1a** (0.2 mmol, 1 equiv), **2** (3 equiv), $NiCl_2(PPh_3)_2$ (10 mol%), XantPhos (10 mol%), LDA (105 mol%), THF (2 mL), -10 °C, 12 h, N_2. Isolated yields.
[b] *E*/*Z* ratio was determined by ^{19}F NMR analysis.

Table S2. Base Screening[a]

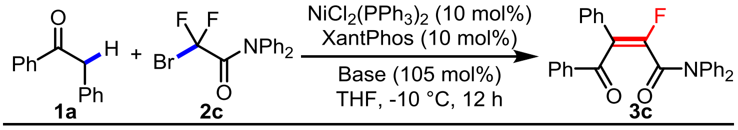

Entry	Base	Yield (%)[b]
1	^{t}BuOK	trace
2	NaH	trace
3	NaOMe	trace
4	K_2CO_3	trace
5	K_3PO_4	trace
6	LiHMDS	59
7	NaHMDS	50
8	KHMDS	44
9	LDA	91

[a] General conditions: **1a** (0.2 mmol), **2c** (3 eq.), $NiCl_2(PPh_3)_2$ (10 mol%), XantPhos (10 mol%), Base (105 mol%), THF (2 mL), 12 h, N_2, -10 °C. [b] Isolated Yield. E/Z > 99:1.

Table S3. Catalyst Screening[a]

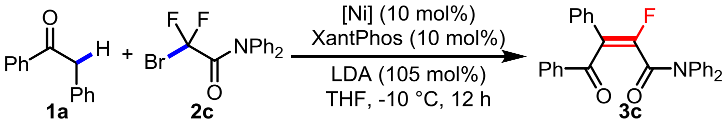

Entry	[Ni]	Yield (%)[b]	Entry	[Ni]	Yield (%)[b]
1	$NiCl_2$	45	7	$NiCl_2$•dppe	70
2	$NiBr_2$	53	8	$Ni(acac)_2$	15
3	NiI_2	59	9	$Ni(COD)_2$	85
4	$Ni(OTf)_2$	14	10	$Ni(NO_3)_2$•$6H_2O$	7
5	$NiCl_2$•dppf	55	11	$NiCl_2$•glyme	61
6	$Ni(OAc)_2$	7	12	$NiCl_2$•$(PPh_3)_2$	91

[a] General conditions: **1a** (0.2 mmol), **2c** (3 eq.), [Ni] (10 mol%), XantPhos (10 mol%), LDA (105 mol%), THF (2 mL), 12 h, N_2, -10 °C. [b] Isolated Yield. E/Z > 99:1.

Table S4. Ligand Screening[a]

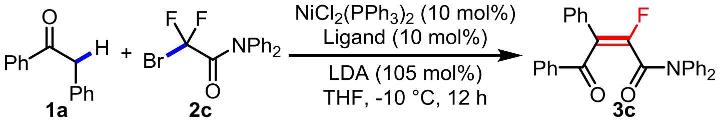

Entry	Ligand	Yield (%)[b]	Entry	Ligand	Yield (%)[b]
1	XantPhos	91	16	L9	23
2	x-phos	36	17	L10	41
3	PCy_3	43	18	L11	30
4	PPh_3	38	19	L12	33
5	BINAP	20	20	L13	24
6	IPr•HCl	18	21	L14	36
7	Phen	36	22	L15	13
8	L1	trace	23	L16	32
9	L2	14	24	L17	20
10	L3	17	25	L18	20
11	L4	28	26	L19	15
12	L5	32	27	L20	36
13	L6	32	28	L21	37
14	L7	33	29	L22	15
15	L8	42	30	L23	22

[a] General conditions: **1a** (0.2 mmol), **2c** (3 eq.), $NiCl_2(PPh_3)_2$ (10 mol%), Ligand (10 mol%), LDA (105 mol%), THF (2 mL), 12 h, N_2, -10 °C. [b] Isolated Yield. E/Z > 99:1.

$HPCy_3$•BF_4

L1

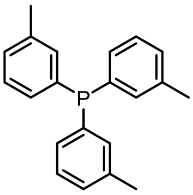

L2

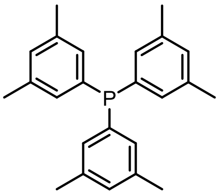

L3

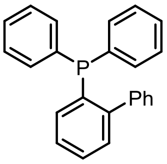

L4

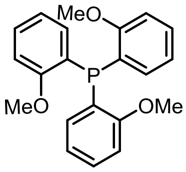

L5

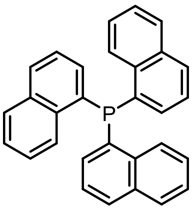

L6

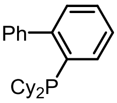

L7

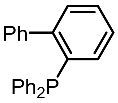

L8

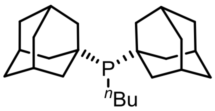

L9

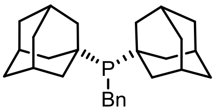

L10

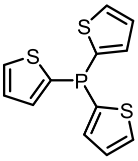

L11

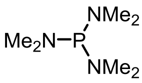

L12

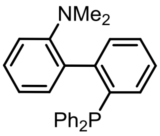

L13

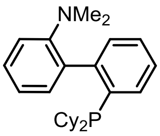

L14

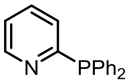

L15

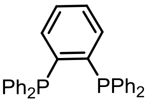

L16

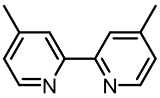

L17

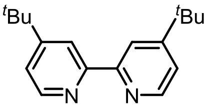

L18

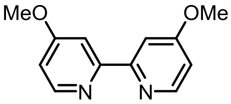

L19

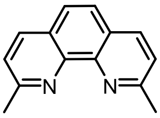

L20

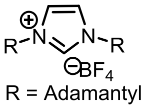

L21

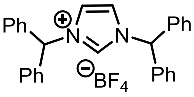

L22

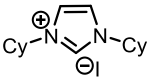

L23

Table S5. Loading of Reagent Screening[a]

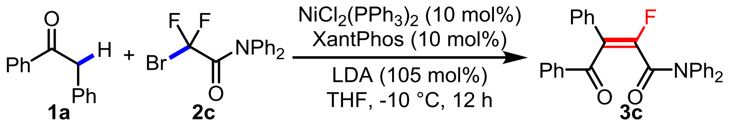

Entry	x	Yield (%)[b]	Entry	x	Yield (%)[b]
1	1	68	4	4	85
2	2	74	5	5	89
3	3	91			

[a] General conditions: **1a** (0.2 mmol), **2c** (x eq.), $NiCl_2(PPh_3)_2$ (10 mol%), XantPhos (10 mol%), LDA(105 mol%), THF (2 mL), 12 h, N_2, -10 °C. [b] Isolated Yield. E/Z > 99:1.

Table S6. Temperature Screening[a]

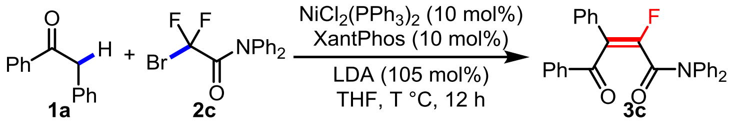

Entry	T	Yield (%)[b]
1	-30	91
2[c]	-10	91
3	0	62
4	50	45

[a] General conditions: **1a** (0.2 mmol), **2c** (3 eq.), $NiCl_2(PPh_3)_2$ (10 mol%), XantPhos (10 mol%), LDA (105 mol%), THF (2 mL), 12 h, N_2, T °C. [b] Isolated Yield. [c] t = 24 h. E/Z > 99:1.

Table S7. Solvent Screening[a]

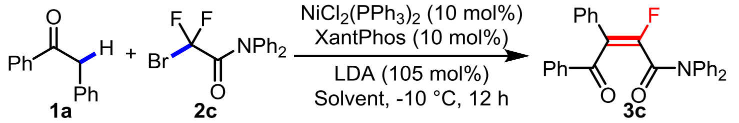

Entry	Solvent	Yield (%)[b]	Entry	Solvent	Yield (%)[b]
1	Toluene	trace	4	NMP	0
2	MeCN	0	5	Dioxane	53
3	DMF	0			

[a] General conditions: **1a** (0.2 mmol), **2c** (3 eq.), $NiCl_2(PPh_3)_2$ (10 mol%), XantPhos (10 mol%), LDA (105 mol%), solvent (2 mL), 12 h, N_2, -10 °C. [b] Isolated Yield. E/Z > 99:1.

Table S8. Time Screening[a]

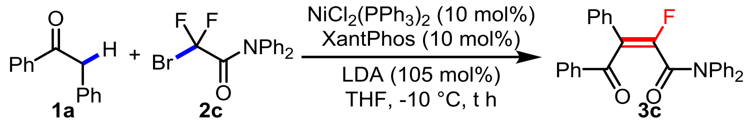

Entry	t	Yield (%)[b]	Entry	t	Yield (%)[b]
1	1	60	3	6	74
2	3	64	4	9	80

[a] General conditions: **1a** (0.2 mmol), **2c** (3 eq.), $NiCl_2(PPh_3)_2$ (10 mol%), XantPhos (10 mol%), LDA (105 mol%), THF (2 mL), t h, N_2, -10 °C. [b] Isolated Yield. E/Z > 99:1.

Tables of the Optimization of Reaction Conditions of Quaternary Alkyl Difluorides

Table S9. Base Screening[a]

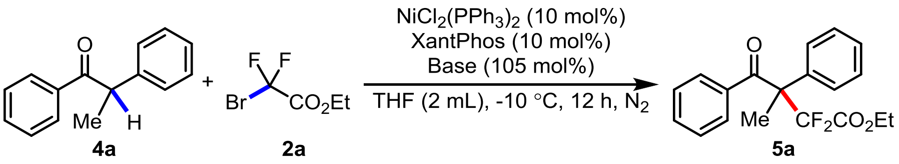

Entry	Base	Yield (%)[b]
1	^{t}BuOK	trace
2	^{t}BuOLi	trace
3	^{t}BuONa	trace
4	LDA	15
5	LiHMDS	12

[a] General conditions: **4a** (0.2 mmol), **2a** (3 eq.), $NiCl_2(PPh_3)_2$ (10 mol%), XantPhos (10 mol%), Base (105 mol%), THF (2 mL), 12 h, N_2. [b] Isolated Yield.

Table S10. Catalyst Screening[a]

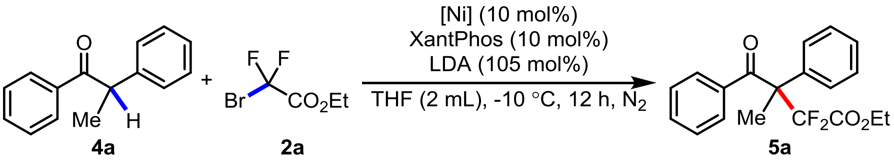

Entry	[Ni]	Yield (%)[b]	Entry	[Ni]	Yield (%)[b]
1	$NiCl_2$	49	7	$NiCl_2$·dppe	70
2	$NiBr_2$	53	8	$Ni(acac)_2$	15
3	NiI_2	45	9	$Ni(COD)_2$	56
4	$Ni(OTf)_2$	14	10	$Ni(NO_3)_2·6H_2O$	7
5	$NiCl_2$·dppf	55	11	$NiCl_2$·glyme	61
6	$Ni(OAc)_2$	7	12	$Ni(PPh_3)_4$	62

[a] General conditions: **4a** (0.2 mmol), **2a** (3 eq.), [Ni] (10 mol%), XantPhos (10 mol%), LDA (105 mol%), THF (2 mL), 12 h, N_2. [b] Isolated Yield.

Table S11. Loading of Reactant[a]

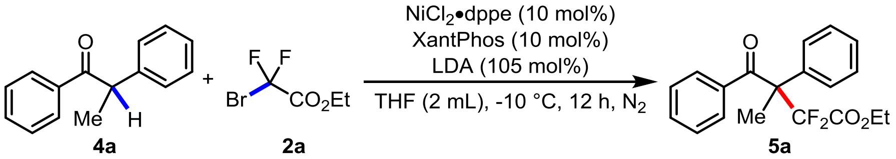

Entry	x	Yield (%)[b]	Entry	x	Yield (%)[b]
1	1	26	4	4	68
2	2	61	5	5	63
3	3	70	6	6	60

[a] General conditions: **4a** (0.2 mmol), **2a** (x eq.), $NiCl_2$•dppe (10 mol%), XantPhos (10 mol%), LDA (105 mol%), THF (2 mL), 12 h, N_2. [b] Isolated Yield.

Table S12. Ligand Screening[a]

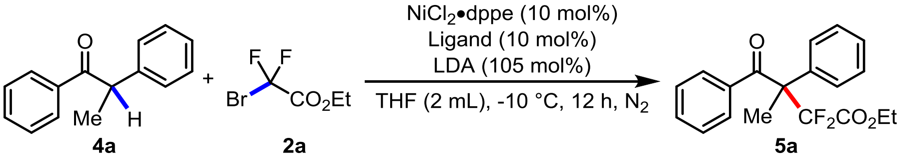

Entry	Ligand	Yield (%)[b]	Entry	Ligand	Yield (%)[b]
1	XantPhos	70	21	L14	38
2	x-phos	23	22	dppe	39
3	PCy_3	43	23	dppp	53
4	PPh_3	trace	24	L15	28
5	bpy	20	25	L16	47
6	dtbpy	18	26	L17	62
7	dppf	29	27	L18	35
8	L1	38	28	L19	11
9	L2	24	29	L20	25
10	L3	47	30	BINAP	32
11	L4	26	31	L21	7
12	L5	39	32	L22	20
13	L6	40	33	L23	23
14	L7	29	34	L24	24
15	L8	35	35	L25	40
16	L9	32	36	L26	30
17	L10	24	37	L27	20
18	L11	43	38	L28	7
19	L12	41	39	L29	21
20	L13	37	40	L30	22

[a] General conditions: **4a** (0.2 mmol), **2a** (3 eq.), $NiCl_2$•dppe (10 mol%), Ligand (10 mol%), LDA (105 mol%), THF (2 mL), 12 h, N_2. [b] Isolated Yield.

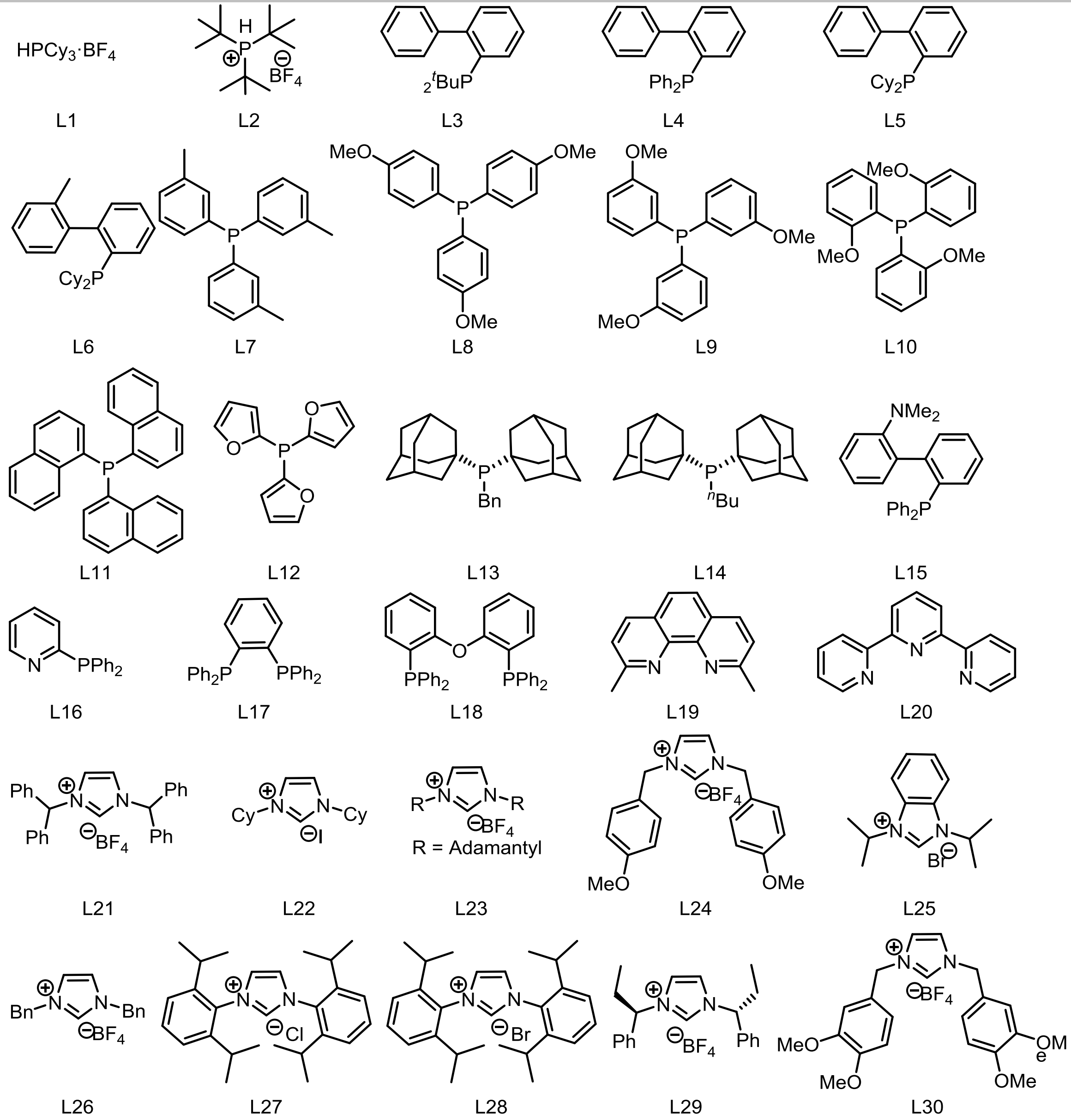

Table S13. Solvent Screening[a]

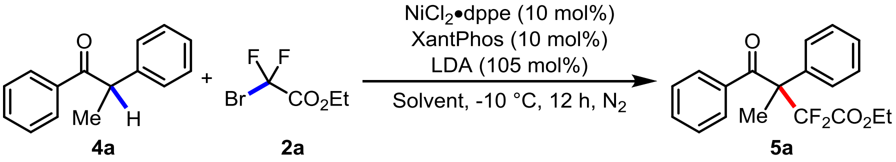

Entry	Solvent	Yield (%)[b]	Entry	Solvent	Yield (%)[b]
1	Toluene	trace	4	NMP	0
2	MeCN	0	5	Dioxane	38
3	DMF	0			

[a] General conditions: **4a** (0.2 mmol), **2a** (3 eq.), $NiCl_2$•dppe (10 mol%), XantPhos (10 mol%), LDA (105 mol%), Solvent (2 mL), 12 h, N_2. [b] Isolated Yield.

Table S14. Zinc Screening[a]

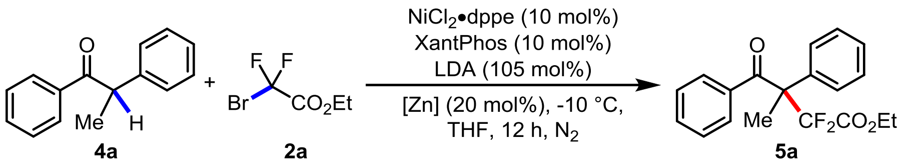

Entry	Zn	Yield (%)[b]	Entry	Zn	Yield (%)[b]
1	$ZnCl_2$	95	5	ZnO	57
2	$ZnBr_2$	69	6	$Zn(OAc)_2$	62
3	ZnI_2	57	7	$Zn(OTf)_2$	57
4	ZnF_2	50			

[a] General conditions: **4a** (0.2 mmol), **2a** (3 eq.), $NiCl_2$•dppe (10 mol%), XantPhos (10 mol%), LDA (105 mol%), THF(2 mL), [Zn] (20 mol%), 12 h, N_2. [b] Isolated Yield.

Table S15. Loading of Zinc Screening[a]

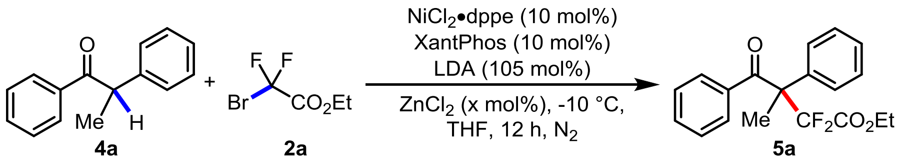

Entry	x	Yield (%)[b]	Entry	x	Yield (%)[b]
1	0.05	66	5	0.3	64
2	0.15	74	6	1	69
3	0.2	95	7	2.3	66
4	0.25	80			

[a] General conditions: **4a** (0.2 mmol), **2a** (3 eq.), $NiCl_2$•dppe (10 mol%), XantPhos (10 mol%), LDA (105 mol%), THF(2 mL), $ZnCl_2$ (x mol%), 12 h, N_2. [b] Isolated Yield.

Table S16. Time of Reaction Screening[a]

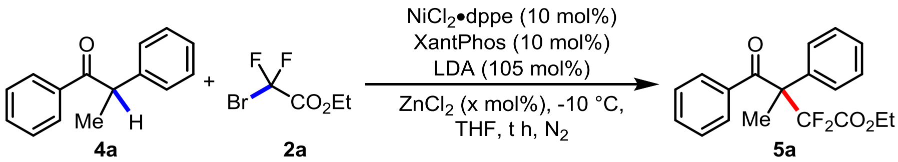

Entry	t	Yield (%)[b]	Entry	t	Yield (%)[b]
1	1	60	3	6	74
2	3	64	4	9	80

[a] General conditions: **4a** (0.2 mmol), **2a** (3 eq.), $NiCl_2$•dppe (10 mol%), XantPhos (10 mol%), LDA (105 mol%), THF(2 mL), $ZnCl_2$ (20 mol%), t h, N_2. [b] Isolated Yield.

General Procedure for Highly Stereoselective Nickel-Catalyzed Difluoroalkylation of Aryl Ketones to Tetrasubstituted Monofluoroalkenes and Quaternary Alkyl Difluorides

To a 50 mL of Schlenk tube was added ketone **1** (1.0 equiv, 0.2 mmol), $NiCl_2$•$(PPh_3)_2$ (10 mol%, 0.02 mmol) and XantPhos (10 mol%, 0.02 mmol) under air. The mixture was evacuated and backfilled with N_2 (3 times). THF (2 mL) was added then followed by LDA (105 mol%, 0.21 mmol) subsequently. The Schlenk tube was then sealed with a Teflon lined cap and put into a cooled bath (-10 °C). After stirring for 5 min, the reaction mixture was added 2-bromo-2,2-difluoro-N,N-diphenylacetamide **2c** (3.0 equiv, 0.6 mmol), then sealed with a Teflon lined cap and put into a cooled bath (-10 °C) for 12 h. And the mixture was added ethyl acetate 5 mL, then solvent was removed under reduced pressure and purified by flash column chromatography on silica gel to give product.

To a 50 mL of Schlenk tube was added ketone **4** (1.0 equiv, 0.2 mmol), $NiCl_2$•dppe (10 mol%, 0.02 mmol) and XantPhos (10 mol%, 0.02 mmol) under air, followed by $ZnCl_2$ (0.2 equiv, 0.04 mmol). The mixture was evacuated and backfilled with N_2 (3 times). THF (2 mL) was added then followed by LDA (105 mol%, 0.21 mmol) subsequently. The Schlenk tube was then sealed with a Teflon lined cap and put into a cooled bath (-10 °C). After stirring for 5 min, the reaction mixture was added bormdifluoroacetate **2a** (3.0 equiv, 0.6 mmol), then sealed with a Teflon lined cap and put into a cooled bath (-10 °C) for 12 h. And the mixture was added ethyl acetate 5 mL, then solvent was removed under reduced pressure and purified by flash column chromatography on silica gel to give product.

Preparation of non-fluorine substituted olefin.

A (20 mL) of Schlenk tube was evacuated and backfilled with N_2 (3 times). The ketone **3s** (1.0 equiv, 0.2 mmol) was added then followed by THF (5 ml) subsequently in N_2 atmosphere. The Schlenk tube was then sealed with a Teflon lined cap and put into a cooled bath (-20 ℃). And the reaction mixture was added EtMgBr (1.2 equiv, 0.24 mmol) dropwise, after 2 hours the temperature was up to 0 ℃, the reaction was detected by TLC. Then mixture was quenched by saturated NH_4Cl solution, and extract 3 times by adding ethyl acetate (10 mL×3), after dehydration by adding Na_2SO_4, the solvents were removed by reduced pressure and purified by flash column chromatography on silica gel to give product **6**.

A (20 mL) of Schlenk tube was evacuated and backfilled with N_2 (3 times). The ketone **3s** (1.0 equiv, 0.2 mmol) was added then followed by THF (5 ml) subsequently in N_2 atmosphere. The Schlenk tube was then sealed with a Teflon lined cap and put into a cooled bath (-20 ℃). And the reaction mixture was added PhMgBr (1.2 equiv, 0.24 mmol) dropwise, after 2 hours the temperature was up to 0 ℃, the reaction was detected by TLC. Then mixture was added saturated NH_4Cl solution, and extract 3 times by adding ethyl acetate (10 mL×3), after dehydration by adding Na_2SO_4, the solvents were removed by reduced pressure and purified by flash column chromatography on silica gel to give product **7**.

A (20 mL) of Schlenk tube was evacuated and backfilled with N_2 (3 times). The ketone **3s** (1.0 equiv, 0.2 mmol) was added then followed by THF (5 ml) subsequently in N_2 atmosphere. The Schlenk tube was then sealed with a Teflon lined cap and put into a cooled bath (-20 ℃). And the reaction mixture was added PhC≡CMgBr (1.2 equiv, 0.24 mmol) dropwise, after 2 hours the temperature was up to 0 ℃, the reaction was detected by TLC. Then mixture was added saturated NH_4Cl solution, and extract 3 times by adding ethyl acetate (10 mL×3), after dehydration by adding Na_2SO_4, the solvents were removed by reduced pressure and purified by flash column chromatography on silica gel to give product **8**.

A (20 ml) flask was added ketone **3s** (1.0 equiv, 0.2 mmol), dichloromethane (5 mL), Et_3N (2 equiv,

0.4 mmol) and BnSH (2 equiv, 0.4 mmol), stirred at room temperature. The reaction was detected by TLC. And the solvent was removed by reduced pressure and purified by flash column chromatography on silica gel to give product **9**.

A (20 mL) of Schlenk tube was evacuated and backfilled with N_2 (3 times). The ketone **3s** (1.0 equiv, 0.2 mmol) was added then followed by THF (5 ml) and BnOH (1.2 equiv, 0.24 mmol) subsequently in N_2 atmosphere. The Schlenk tube was then sealed with a Teflon lined cap and put into a cooled bath (-20 ℃). And the reaction mixture was added n-BuLi (1.2 equiv, 0.24 mmol) dropwise, after 2 hours the temperature was up to 0 ℃, the reaction was detected by TLC. Then mixture was quenched by saturated NH_4Cl solution, and extract 3 times by adding ethyl acetate (10 mL×3), after dehydration by adding Na_2SO_4 the solvents were removed by reduced pressure and purified by flash column chromatography on silica gel to give product **10**.

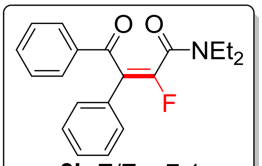

3b E/Z = 7:1

The product **3b** is purified with silica gel chromatography (petroleum ether/ethyl acetate = 10/1 as the eluent) as a yellow oil mixture (72% yield, E/Z = 7:1). ^{1}H NMR (400 MHz, Chloroform-*d*) δ 8.05 – 8.03 (m, 0.21H, Z), 7.92 – 7.89 (m, 1.62H, E), 7.58 – 7.43 (m, 3.24H), 7.38 - 7.24 (m, 4.69H), 3.46 – 3.26 (m, 4H), 1.27 – 1.23 (m, 2.86H), 1.03 – 0.92 (m, 3.24H). ^{13}C NMR (101 MHz, Chloroform-*d*) δ 192.72 (d, *J* = 7.7 Hz, E), 192.38 (d, *J* = 3.3 Hz, Z), 161.43 (d, *J* = 29.2 Hz, E), 161.00 (d, *J* = 32.4 Hz, Z), 151.65 (d, *J* = 287.5 Hz, E), 149.12(d, *J* = 269.7 Hz, Z), 136.63 (d, *J* = 3.9 Hz), 135.84, 134.15, 133.24, 131.14, 130.43 (d, *J* = 7.1 Hz), 129.71, 128.99, 128.94, 128.91, 128.89, 128.83, 128.79, 128.53, 128.25 (d, *J* = 2.9 Hz), 126.98 (d, *J* = 6.2 Hz), 42.99, 42.92, 42.89, 40.25 (E), 39.06 (Z), 14.42 (E), 14.02 (Z), 12.16 (E), 11.96 (Z). ^{19}F NMR (376 MHz, Chloroform-*d*) δ -105.45 (Z), -113.26 (E). HRMS (ESI) (*m/z*): $[M+H]^+$ calcd. for $C_{20}H_{21}FNO_2$: 326.1556, found: 326.1584.

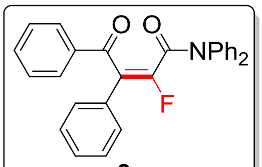

3c

The product **3c** is purified with silica gel chromatography (petroleum ether/ethyl acetate = 10/1 as the eluent) as a white solid (91% yield). ^{1}H NMR (400 MHz, Chloroform-*d*) δ 7.84 (d, *J* = 7.4 Hz, 2H), 7.62 – 7.07 (m, 18H). ^{13}C NMR (101 MHz, Chloroform-*d*) δ 192.66 (d, *J* = 6.8 Hz), 162.17 (d, *J* = 28.7 Hz), 151.04 (d, *J* = 286.6 Hz), 141.98, 136.45 (d, *J* = 3.9 Hz), 133.38, 131.10, 129.95, 129.41, 129.16, 128.88, 128.83, 128.62, 127.33, 126.81. ^{19}F NMR (376 MHz, Chloroform-*d*) δ -113.23. HRMS (ESI) (m/z): $[M+Na]^+$ calcd. for $C_{28}H_{20}O_2NFNa$: 444.1370, found: 444.1376.

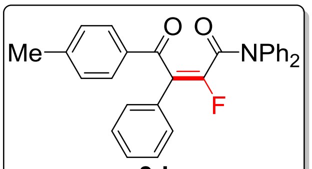

3d

The product **3d** is purified with silica gel chromatography (petroleum ether/ethyl acetate = 10/1 as the eluent) as a white solid (74% yield). ^{1}H NMR (400 MHz, Chloroform-*d*) δ 7.74 (d, *J* = 8.2 Hz, 2H), 7.39 – 7.11 (m, 17H), 2.33 (s, 3H). ^{13}C NMR (101 MHz, Chloroform-*d*) δ 192.29 (d, *J* = 6.9 Hz), 162.18 (d, *J* = 28.8 Hz), 150.83 (d, *J* = 286.1 Hz), 144.33, 142.00, 134.04 (d, *J* = 4.0 Hz), 131.26, 130.09, 129.35, 129.07, 128.96, 128.90, 128.86, 128.84, 128.81, 127.29, 126.77, 21.85. ^{19}F NMR (376 MHz, Chloroform-*d*) δ -113.76. HRMS (ESI) (m/z): $[M+H]^+$ calcd. for $C_{29}H_{23}FO_2N$: 436.1711, found: 436.1707.

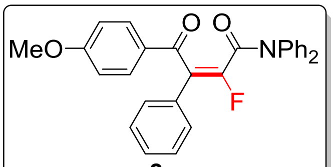

3e

The product **3e** is purified with silica gel chromatography (petroleum ether/ethyl acetate = 10/1 as the eluent) as a white solid (74% yield). ^{1}H NMR (400 MHz, Chloroform-*d*) δ 7.81 (d, *J* = 8.9 Hz, 2H), 7.37 – 7.18 (m, 15H), 6.82 (d, *J* = 8.9 Hz, 2H), 3.78 (s, 3H). ^{13}C NMR (101 MHz, Chloroform-*d*) δ 191.25 (d, *J* = 7.0 Hz), 163.76, 162.23 (d, *J* = 29.0 Hz), 150.73 (d, *J* = 285.6 Hz), 141.99, 132.32, 131.38, 129.59 (d, *J* = 4.0 Hz), 129.34, 129.03, 128.82, 128.77, 127.26, 126.77, 113.91, 55.56. ^{19}F NMR (376 MHz, Chloroform-*d*) δ -114.02. HRMS (ESI) (m/z): [M+Na]$^{+}$ calcd. for $C_{29}H_{22}FO_{3}NNa$: 474.1476, found: 474.1481.

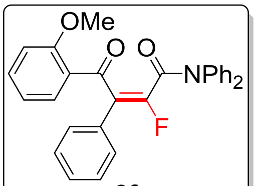

3f

The product **3f** is purified with silica gel chromatography (petroleum ether/ethyl acetate = 10/1 as the eluent) as a white solid (55% yield). ^{1}H NMR (400 MHz, Chloroform-*d*) δ 7.81 – 7.67 (m, 1H), 7.55 – 7.06 (m, 16H), 6.92 (t, 1H), 6.72 (d, *J* = 8.4 Hz, 1H), 3.55 (s, 3H). ^{13}C NMR (101 MHz, Chloroform-*d*) δ 192.14 (d, *J* = 8.6 Hz), 162.40 (d, *J* = 29.3 Hz), 158.77, 153.57, 152.17 (d, *J* = 282.3 Hz), 134.39, 131.69, 131.20, 129.86 (d, *J* = 7.8 Hz), 129.30, 129.11, 129.07, 128.45, 128.06, 127.28, 126.96, 126.73 (d, *J* = 3.5 Hz), 120.53, 111.64, 55.40. ^{19}F NMR (376 MHz, Chloroform-*d*) δ -112.49. HRMS (ESI) (*m/z*): [M+H]$^{+}$ calcd. for $C_{29}H_{23}FO_{3}N$: 452.1656, found: 452.1661.

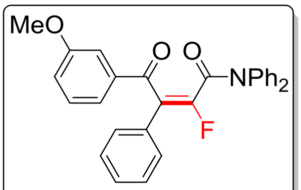

3g

The product **3g** is purified with silica gel chromatography (petroleum ether/ethyl acetate = 10/1 as the eluent) as a white solid (57% yield). ^{1}H NMR (400 MHz, Chloroform-*d*) δ 7.44 – 7.10 (m, 18H), 7.04 – 6.97 (m, 1H), 3.76 (s, 3H). ^{13}C NMR (101 MHz, Chloroform-*d*) δ 192.33 (d, *J* = 6.9 Hz), 162.11 (d, *J* = 28.6 Hz), 159.72, 150.97 (d, *J* = 286.6 Hz), 141.99, 137.70 (d, *J* = 3.9 Hz), 131.20, 129.61, 129.39, 129.17, 129.02, 128.96, 128.88, 128.83, 128.78, 127.35, 126.76, 123.03, 120.28, 113.45, 55.44. ^{19}F NMR (376 MHz, Chloroform-*d*) δ -113.19. HRMS (ESI) (*m/z*): [M+H]$^{+}$ calcd. for $C_{29}H_{23}FO_{3}N$: 452.1656, found: 452.1662.

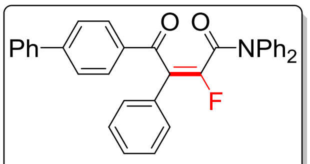

3h

The product **3h** is purified with silica gel chromatography (petroleum ether/ethyl acetate = 10/1 as the eluent) as a white solid (62% yield). ^{1}H NMR (400 MHz, Chloroform-*d*) δ 7.92 (d, *J* = 8.2 Hz, 2H), 7.61 – 7.51 (m, 4H), 7.46 – 7.40 (m, 2H), 7.39 – 7.20 (m, 16H). ^{13}C NMR (101 MHz, Chloroform-*d*) δ 192.23 (d, *J* = 6.9 Hz), 162.21 (d, *J* = 28.7 Hz), 150.91 (d, *J* = 286.7 Hz), 146.11, 141.99, 140.08, 135.24 (d, *J* = 3.9 Hz), 131.13, 130.52, 129.42, 129.21, 129.02, 128.98, 128.94, 128.90, 128.85, 128.31, 127.40, 127.05, 126.78. ^{19}F NMR (376 MHz, Chloroform-*d*) δ -113.48. HRMS (ESI) (*m/z*): [M+H]$^{+}$ calcd. for $C_{34}H_{25}FO_{2}N$: 498.1864, found: 498.1863.

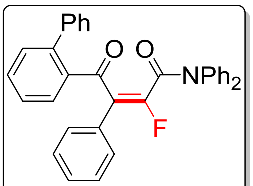

3i

The product **3i** is purified with silica gel chromatography (petroleum ether/ethyl acetate = 10/1 as the eluent) as a white solid (63% yield). ^{1}H NMR (400 MHz, Chloroform-*d*) δ 7.67 (d, *J* = 7.6 Hz, 1H), 7.51 – 7.21 (m, 15H), 7.13 (dd, *J* = 18.3, 7.4 Hz, 4H), 7.04 (t, *J* = 7.4 Hz, 2H), 6.60 (d, *J* = 7.7 Hz, 2H). ^{13}C NMR (101 MHz, Chloroform-*d*) δ 195.20 (d, *J* = 9.2 Hz), 162.19, 154.93 (d, *J* = 287.1 Hz), 153.50, 142.15, 141.65, 140.13, 136.98, 136.94, 131.54, 130.92, 130.83, 130.24, 129.55, 129.33, 129.29, 128.25, 128.17, 127.93, 127.60, 127.15, 127.04, 126.95, 126.72, 126.56. ^{19}F NMR (376 MHz, Chloroform-*d*) δ -104.93. HRMS (ESI) (*m/z*): [M+H]$^{+}$ calcd. for $C_{34}H_{25}FO_{2}N$: 498.1864, found: 498.1868.

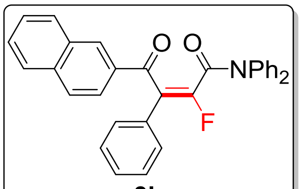

3j

The product **3j** is purified with silica gel chromatography (petroleum ether/ethyl acetate = 10/1 as the eluent) as a white solid (59% yield). ^{1}H NMR (400 MHz, Chloroform-*d*) δ 8.30 (s, 1H), 7.93 (d, *J* = 8.6 Hz, 1H), 7.85 – 7.76 (m, 3H), 7.58 – 7.51 (m, 1H), 7.50 – 7.45 (m, 1H), 7.40 – 7.16 (m, 15H). ^{13}C NMR (101 MHz, Chloroform-*d*) δ 192.60 (d, *J* = 7.0 Hz), 162.16 (d, *J* = 28.7 Hz), 151.10 (d, *J* = 286.5 Hz), 141.97, 135.79, 133.95 (d, *J* = 3.9 Hz), 132.49, 132.34, 131.27, 129.85, 129.38, 129.18, 128.97, 128.91, 128.87, 128.71, 128.52, 127.84, 127.29, 126.85, 126.72, 124.94. ^{19}F NMR (376 MHz, Chloroform-*d*) δ -112.89. HRMS (ESI) (*m/z*): [M+H]$^+$ calcd. for $C_{32}H_{23}FO_2N$:472.1707, found: 472.1719.

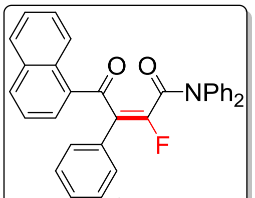

3k

The product **3k** is purified with silica gel chromatography (petroleum ether/ethyl acetate = 10/1 as the eluent) as a white solid (57% yield). ^{1}H NMR (400 MHz, Chloroform-*d*) δ 9.05 – 8.79 (m, 1H), 7.96 – 7.76 (m, 2H), 7.70 – 7.57 (m, 2H), 7.55 – 7.00 (m, 17H). ^{13}C NMR (101 MHz, Chloroform-*d*) δ 194.78 (d, *J* = 7.9 Hz), 162.23 (d, *J* = 29.4 Hz), 153.05 (d, *J* = 285.6 Hz), 141.91, 133.90, 133.76, 133.11 (d, *J* = 3.8 Hz), 132.54, 131.64, 131.17, 129.41, 128.88, 128.83, 128.69, 128.51 (d, *J* = 5.1 Hz), 127.39, 126.95, 126.56, 125.95, 124.32. ^{19}F NMR (376 MHz, Chloroform-*d*) δ -109.34. HRMS (ESI) (*m/z*): [M+H]$^+$ calcd. for $C_{32}H_{23}FO_2N$:472.1707, found: 472.1712.

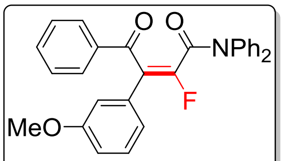

3l

The product **3l** is purified with silica gel chromatography (petroleum ether/ethyl acetate = 10/1 as the eluent) as a white solid (78% yield). ^{1}H NMR (400 MHz, Chloroform-*d*) δ 7.84 (d, *J* = 7.5 Hz, 2H), 7.45 (t, *J* = 7.4 Hz, 1H), 7.27 (m, 13H), 6.88 (d, *J* = 8.4 Hz, 2H), 6.80 (d, *J* = 8.7 Hz, 1H), 3.70 (s, 3H). ^{13}C NMR (101 MHz, Chloroform-*d*) δ 192.51 (d, *J* = 6.9 Hz), 162.27, 161.99, 159.67, 151.13 (d, *J* = 287.0 Hz), 141.94, 136.44, 136.41, 133.38, 132.17, 129.89, 129.86, 129.39, 128.80, 128.74, 128.60, 121.29 (d, *J* = 4.7 Hz), 114.74, 114.38 (d, *J* = 5.3 Hz), 55.32. ^{19}F NMR (376 MHz, Chloroform-*d*) δ -112.53. HRMS (ESI) (*m/z*): [M+H]$^+$ calcd. for $C_{29}H_{23}FO_3N$:452.1656, found: 452.1663.

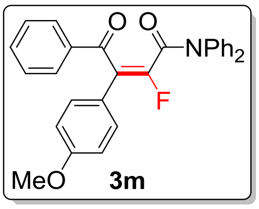

3m

The product **3m** is purified with silica gel chromatography (petroleum ether/ethyl acetate = 10/1 as the eluent) as a white solid (73% yield). ^{1}H NMR (400 MHz, Chloroform-*d*) δ 7.86 (d, *J* = 7.5 Hz, 2H), 7.60 – 7.08 (m, 15H), 6.80 (d, *J* = 8.8 Hz, 2H), 3.73 (s, 3H). ^{13}C NMR (101 MHz, Chloroform-*d*) δ 192.88 (d, *J* = 6.9 Hz), 162.37 (d, *J* = 28.5 Hz), 160.14, 149.83 (d, *J* = 284.8 Hz). 142.14, 136.57 (d, *J* = 3.9 Hz), 133.29, 130.44, 130.39, 129.89, 129.36, 129.02, 128.96, 128.61, 127.24, 126.78, 123.40, 114.35, 55.34. ^{19}F NMR (376 MHz, Chloroform-*d*) δ -115.72. HRMS (ESI) (*m/z*): [M+H]$^+$ calcd. for $C_{29}H_{23}FO_3N$:452.1656, found: 452.1663.

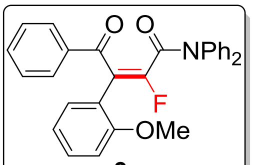

3n

The product **3n** is purified with silica gel chromatography (petroleum ether/ethyl acetate = 10/1 as the eluent) as a white solid (67% yield). ^{1}H NMR (400 MHz, Chloroform-*d*) δ 7.59 (d, *J* = 7.5 Hz, 2H), 7.40 (t, *J* = 7.4 Hz, 1H), 7.34 – 7.13 (m, 14H), 6.91 (t, *J* = 7.5 Hz, 1H), 6.71 (d, *J* = 8.6 Hz, 1H), 3.54 (s, 3H). ^{13}C NMR (101 MHz, Chloroform-*d*) δ 192.91 (d, *J* = 8.7 Hz), 162.15, 161.85, 154.28 (d, *J* = 281.2 Hz), 152.88, 141.90, 137.01 (d, *J* = 3.6 Hz), 132.60, 130.63, 130.47, 130.43, 129.30, 129.19, 128.01, 127.41, 126.55, 123.60, 123.51, 121.11, 120.83, 111.50, 55.42. ^{19}F NMR (376 MHz, Chloroform-*d*) δ -104.26. HRMS (ESI) (*m/z*): [M+H]$^+$ calcd. for $C_{29}H_{23}FO_3N$:452.1656, found: 452.1668.

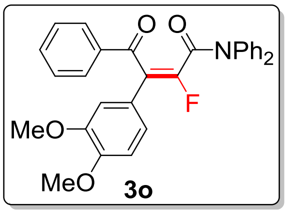

The product **3o** is purified with silica gel chromatography (petroleum ether/ethyl acetate = 10/1 as the eluent) as a white solid (62% yield). ^{1}H NMR (400 MHz, Chloroform-*d*) δ 7.91 – 7.82 (m, 2H), 7.51 – 7.16 (m, 13H), 6.87 (d, *J* = 8.5 Hz, 2H), 6.75 (d, *J* = 8.2 Hz, 1H), 3.82 (s, 3H), 3.76 (s, 3H). ^{13}C NMR (101 MHz, Chloroform-*d*) δ 192.82 (d, *J* = 6.8 Hz), 162.36 (d, *J* = 28.4 Hz), 150.01 (d, *J* = 286.8 Hz), 149.83, 148.96, 142.10, 136.58 (d, *J* = 3.8 Hz), 133.33, 129.84, 129.38, 129.21, 129.15, 128.62, 127.25, 126.75, 123.52, 122.30, 122.25, 111.72, 111.66, 111.16, 55.95, 55.92. ^{19}F NMR (376 MHz, Chloroform-*d*) δ -115.28. HRMS (ESI) (*m/z*): [M+H]$^+$ calcd. for $C_{30}H_{25}FO_4N$:482.1762, found: 482.1768.

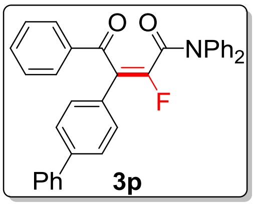

The product **3p** is purified with silica gel chromatography (petroleum ether/ethyl acetate = 10/1 as the eluent) as a white solid (66% yield). ^{1}H NMR (400 MHz, Chloroform-*d*) δ 7.85 – 7.78 (m, 2H), 7.49 – 7.09 (m, 22H). ^{13}C NMR (101 MHz, Chloroform-*d*) δ 192.70 (d, *J* = 6.8 Hz), 162.19 (d, *J* = 28.5 Hz), 150.92 (d, *J* = 287.1 Hz), 142.01, 141.89, 140.11, 136.50 (d, *J* = 3.8 Hz), 133.46, 129.98, 129.43, 129.33, 129.28, 128.96, 128.79, 128.73, 128.69, 127.89, 127.53, 127.12, 126.79. ^{19}F NMR (376 MHz, Chloroform-*d*) δ -113.25. HRMS (ESI) (*m/z*): [M+H]$^+$ calcd. for $C_{34}H_{25}FO_2N$:498.1864, found: 498.1875.

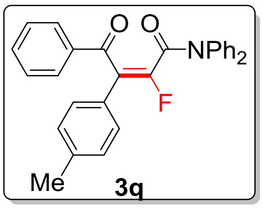

The product **3q** is purified with silica gel chromatography (petroleum ether/ethyl acetate = 10/1 as the eluent) as a white solid (81% yield). ^{1}H NMR (400 MHz, Chloroform-*d*) δ 7.84 (d, *J* = 7.3 Hz, 2H), 7.45 (t, *J* = 7.4 Hz, 1H), 7.38 – 7.18 (m, 14H), 7.09 (d, *J* = 8.0 Hz, 2H), 2.27 (s, 3H). ^{13}C NMR (101 MHz, Chloroform-*d*) δ 192.82 (d, *J* = 6.9 Hz), 162.45, 162.17, 150.53 (d, *J* = 285.8 Hz), 142.06, 139.39, 136.52 (d, *J* = 3.9 Hz), 133.31, 129.93, 129.63, 129.38, 129.11, 129.05, 128.79, 128.74, 128.60, 128.19, 127.27, 126.77, 21.43. ^{19}F NMR (376 MHz, Chloroform-*d*) δ -114.20. HRMS (ESI) (*m/z*): [M+H]$^+$ calcd. for $C_{29}H_{23}FO_2N$:436.1711, found: 436.1711.

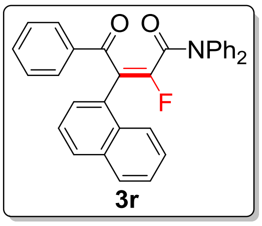

The product **3r** is purified with gel chromatography (petroleum ether/ethyl acetate = 10/1 as the eluent) as a white solid (55% yield). ^{1}H NMR (400 MHz, Chloroform-*d*) δ 7.80 – 7.68 (m, 5H), 7.53 – 7.38 (m, 4H), 7.38 – 7.26 (m, 9H), 7.24 – 7.10 (m, 4H). ^{13}C NMR (101 MHz, Chloroform-*d*) δ 192.80 (d, *J* = 8.2 Hz), 161.89 (d, *J* = 29.7 Hz), 154.21 (d, *J* = 284.0 Hz), 141.73, 136.56 (d, *J* = 3.8 Hz), 133.89, 133.21, 130.87, 129.68, 129.55, 129.29, 128.73, 128.51, 128.38, 127.52 (d, *J* = 1.7 Hz), 126.86, 126.23, 126.19, 126.09, 125.38 (d, *J* = 1.8 Hz), 125.25. ^{19}F NMR (376 MHz, Chloroform-*d*) δ -100.88. HRMS (ESI) (*m/z*): [M+H]$^+$ calcd. for $C_{32}H_{23}FO_2N$:472.1707, found: 472.1711.

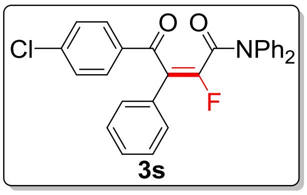

The product **3s** is purified with silica gel chromatography (petroleum ether/ethyl acetate = 10/1 as the eluent) as a white solid (66% yield). ^{1}H NMR (400 MHz, Chloroform-*d*) δ 7.76 (d, *J* = 8.5 Hz, 2H), 7.39 – 7.19 (m, 17H). ^{13}C NMR (101 MHz, Chloroform-*d*) δ 191.44 (d, *J* = 7.0 Hz), 162.08 (d, *J* = 28.3 Hz), 151.07 (d, *J* = 287.9 Hz), 141.89, 139.79, 134.85, 131.25, 130.73, 129.45, 129.36, 129.01, 128.99, 128.80, 128.75, 128.70, 128.64, 127.48, 126.79. ^{19}F NMR (376 MHz, Chloroform-*d*) δ -112.88. HRMS (ESI) (*m/z*): [M+H]$^+$ calcd. for $C_{28}H_{20}FClO_2N$: 456.1161, found: 456.1166.

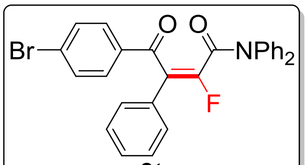
3t

The product **3t** is purified with silica gel chromatography (petroleum ether/ethyl acetate = 10/1 as the eluent) as a white solid (80% yield). ^{1}H NMR (400 MHz, Chloroform-*d*) δ 7.61 (d, *J* = 8.5 Hz, 2H), 7.41 (d, *J* = 8.5 Hz, 2H), 7.31 – 7.08 (m, 15H). ^{13}C NMR (101 MHz, Chloroform-*d*) δ 191.63 (d, *J* = 7.0 Hz), 162.06 (d, *J* = 28.3 Hz), 151.08 (d, *J* = 287.9 Hz), 141.88, 135.24 (d, *J* = 4.0 Hz), 131.97, 131.33, 130.70, 129.45, 129.37, 129.01, 128.80, 128.75, 128.66, 128.61, 128.60, 127.42, 126.76. ^{19}F NMR (376 MHz, Chloroform-*d*) δ -112.79. HRMS (ESI) (*m/z*): [M+Na]$^+$ calcd. for $C_{28}H_{19}BrO_2NFNa$: 522.0475, found: 522.0481.

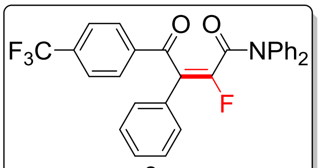
3u

The product **3u** is purified with silica gel chromatography (petroleum ether/ethyl acetate = 10/1 as the eluent) as a white solid (44% yield). ^{1}H NMR (400 MHz, Chloroform-*d*) δ 7.95 (d, *J* = 8.0 Hz, 2H), 7.60 (d, *J* = 8.2 Hz, 2H), 7.46 – 7.07 (m, 15H). ^{13}C NMR (101 MHz, Chloroform-*d*) δ 191.50 (d, *J* = 7.0 Hz), 162.02 (d, *J* = 28.1 Hz), 151.17 (d, *J* = 288.9 Hz), 141.84, 139.13, 134.39 (q, *J* = 32.6 Hz), 130.39, 130.09, 129.52, 129.10, 128.79, 128.74, 128.67, 127.15 (d, *J* = 77.8 Hz), 125.70 (q, *J* = 3.7 Hz), 124.99. ^{19}F NMR (376 MHz, Chloroform-*d*) δ -63.16, -112.38. HRMS (ESI) (*m/z*): [M+H]$^+$ calcd. for $C_{29}H_{20}F_4O_2N$: 490.1425, found: 490.1433.

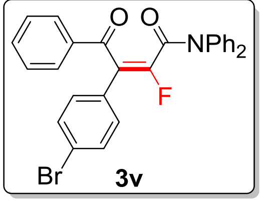
3v

The product **3v** is purified with silica gel chromatography (petroleum ether/ethyl acetate = 10/1 as the eluent) as a white solid (67% yield). ^{1}H NMR (400 MHz, Chloroform-*d*) δ 7.81 (d, *J* = 7.5 Hz, 2H), 7.55 – 7.10 (m, 17H). ^{13}C NMR (101 MHz, Chloroform-*d*) δ 192.25 (d, *J* = 6.7 Hz), 161.86 (d, *J* = 28.5 Hz), 151.38 (d, *J* = 288.0 Hz), 141.83, 136.23, 136.19, 133.60, 132.16, 130.37, 130.32, 129.95, 129.92, 129.43, 128.72, 127.74, 127.69, 127.52, 126.75. ^{19}F NMR (376 MHz, Chloroform-*d*) δ -111.84. HRMS (ESI) (*m/z*): [M+H]$^+$ calcd. for $C_{28}H_{20}FBrO_2N$: 500.0656, found: 500.0663.

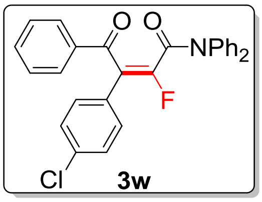
3w

The product **3w** is purified with silica gel chromatography (petroleum ether/ethyl acetate = 10/1 as the eluent) as a white solid (88% yield). ^{1}H NMR (400 MHz, Chloroform-*d*) δ 7.81 (d, *J* = 7.3 Hz, 2H), 7.49 (t, *J* = 7.4 Hz, 1H), 7.40 – 7.14 (m, 16H). ^{13}C NMR (101 MHz, Chloroform-*d*) δ 192.33 (d, *J* = 6.8 Hz), 161.88 (d, *J* = 28.5 Hz), 151.38 (d, *J* = 287.7 Hz), 141.86, 136.26, 136.22, 135.27, 133.60, 130.17, 130.11, 129.93, 129.50, 129.44, 129.22, 128.73, 127.72, 127.66, 127.46, 126.81. ^{19}F NMR (376 MHz, Chloroform-*d*) δ -112.10. HRMS (ESI) (*m/z*): [M+H]$^+$ calcd. for $C_{28}H_{20}FClO_2N$: 456.1161, found: 456.1169.

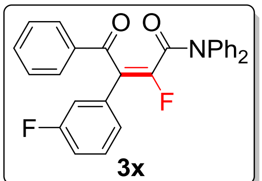
3x

The product **3x** is purified with silica gel chromatography (petroleum ether/ethyl acetate = 10/1 as the eluent) as a white solid (62% yield). ^{1}H NMR (400 MHz, Chloroform-*d*) δ 7.82 (d, *J* = 7.6 Hz, 2H), 7.57 – 6.88 (m, 17H). ^{13}C NMR (101 MHz, Chloroform-*d*) δ 192.13 (d, *J* = 6.7 Hz), 163.94, 161.93, 161.65, 161.48, 151.63 (d, *J* = 288.6 Hz), 141.80, 136.22 (d, *J* = 3.8 Hz), 133.61, 132.90 (d, *J* = 8.2 Hz), 130.42 (d, *J* = 8.4 Hz), 129.91, 129.44, 128.71, 127.69 – 127.52 (m), 127.38, 126.83, 124.79 – 124.42 (m), 116.23 (d, *J* = 21.1 Hz), 115.98 (d, *J* = 5.8 Hz), 115.75 (d, *J* = 5.9 Hz). ^{19}F NMR (376 MHz, Chloroform-*d*) δ -111.31, -111.62. HRMS (ESI) (*m/z*): [M+H]$^+$ calcd. for $C_{28}H_{20}F_2O_2N$: 440.1457, found: 440.1467.

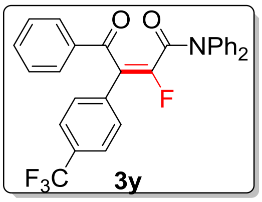

The product **3y** is purified with silica gel chromatography (petroleum ether/ethyl acetate = 10/1 as the eluent) as a white solid (72% yield). ^{1}H NMR (400 MHz, Chloroform-*d*) δ 7.82 (d, *J* = 7.9 Hz, 2H), 7.61 – 7.12 (m, 17H). ^{13}C NMR (101 MHz, Chloroform-*d*) δ 192.00 (d, *J* = 6.7 Hz), 161.59 (d, *J* = 28.5 Hz), 152.24 (d, *J* = 289.5 Hz), 141.68, 136.10 (d, *J* = 3.8 Hz), 134.61, 133.73, 130.86 (q, *J* = 32.7 Hz), 129.92, 129.45, 129.13, 129.08, 128.77, 127.83, 127.66, 127.20 (d, *J* = 5.7 Hz), 126.59, 125.82 (q, *J* = 3.7 Hz), 125.13. ^{19}F NMR (376 MHz, Chloroform-*d*) δ -62.94, -110.36. HRMS (ESI) (*m/z*): $[M+H]^+$ calcd. for $C_{29}H_{20}F_4O_2N$: 490.1425, found: 490.1430.

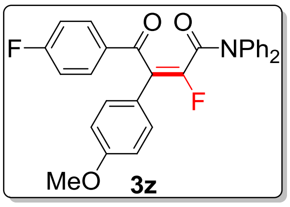

The product **3z** is purified with silica gel chromatography (petroleum ether/ethyl acetate = 10/1 as the eluent) as a white solid (65% yield). ^{1}H NMR (400 MHz, Chloroform-*d*) δ 7.79 (dd, *J* = 8.7, 5.5 Hz, 2H), 7.19 (m, 12H), 6.92 (t, *J* = 8.6 Hz, 2H), 6.71 (d, *J* = 8.9 Hz, 2H), 3.64 (s, 3H). ^{13}C NMR (101 MHz, Chloroform-*d*) δ 191.32 (d, *J* = 7.0 Hz), 167.00, 164.46, 162.30 (d, *J* = 28.3 Hz), 160.23, 149.78 (d, *J* = 285.3 Hz), 142.04, 133.08 – 132.95 (m), 132.52, 132.43, 130.32 (d, *J* = 5.8 Hz), 129.36, 128.78 (d, *J* = 6.0 Hz), 127.29, 126.70, 123.10, 115.88, 115.67, 114.41, 55.31. ^{19}F NMR (376 MHz, Chloroform-*d*) δ -104.59, -115.54. HRMS (ESI) (*m/z*): $[M+Na]^+$ calcd. for $C_{29}H_{21}F_2O_3NNa$: 492.1382, found: 492.1387.

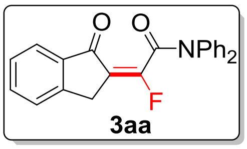

The product **3aa** is purified with silica gel chromatography (petroleum ether/ethyl acetate = 10/1 as the eluent) as a white solid (36% yield). ^{1}H NMR (400 MHz, Chloroform-*d*) δ 7.77 (d, *J* = 7.6 Hz, 1H), 7.64 – 7.49 (m, 3H), 7.47 – 7.33 (m, 4H), 7.34 – 7.17 (m, 6H), 3.56 (d, *J* = 1.7 Hz, 2H). ^{13}C NMR (101 MHz, Chloroform-*d*) δ 191.64 (d, *J* = 15.4 Hz), 160.93 (d, *J* = 29.8 Hz), 156.34 (d, *J* = 284.9 Hz), 148.24, 141.53, 141.25, 138.17 (d, *J* = 4.5 Hz), 129.36, 129.31, 128.45, 127.97, 127.53, 127.35, 126.62, 126.39, 124.20, 117.41 (d, *J* = 11.3 Hz), 28.33 (d, *J* = 1.7 Hz). ^{19}F NMR (376 MHz, Chloroform-*d*) δ -95.76. HRMS (ESI) (*m/z*): $[M+Na]^+$ calcd. for $C_{23}H_{16}FO_2NNa$: 380.1063, found: 380.1044.

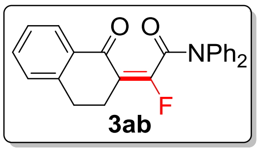

The product **3ab** is purified with silica gel chromatography (petroleum ether/ethyl acetate = 10/1 as the eluent) as a white solid (46% yield). ^{1}H NMR (400 MHz, Chloroform-*d*) δ 8.03 (d, *J* = 7.8 Hz, 1H), 7.54 (d, *J* = 8.0 Hz, 2H), 7.42 (dt, *J* = 14.9, 7.6 Hz, 3H), 7.31 (t, *J* = 7.6 Hz, 1H), 7.25 (t, *J* = 7.4 Hz, 1H), 7.22 – 7.10 (m, 6H), 2.66 – 2.53 (m, 4H). ^{13}C NMR (101 MHz, Chloroform-*d*) δ 186.15 (d, *J* = 13.7 Hz), 161.97 (d, *J* = 30.4 Hz), 156.48 (d, *J* = 277.8 Hz), 143.26, 141.58 (d, *J* = 43.8 Hz), 133.72, 132.88 (d, *J* = 4.6 Hz), 129.21, 129.10, 128.40, 128.11, 127.80, 127.71, 127.14, 126.92, 126.37, 118.21 (d, *J* = 13.2 Hz), 28.10 (d, *J* = 1.7 Hz), 22.82 (d, *J* = 5.6 Hz). ^{19}F NMR (376 MHz, Chloroform-*d*) δ -99.64. HRMS (ESI) (*m/z*): $[M+Na]^+$ calcd. for $C_{24}H_{18}O_2NFNa$: 394.1214, found: 394.1219.

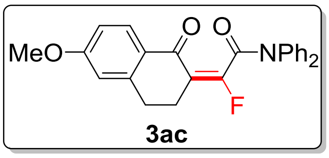

The product **3ac** is purified with silica gel chromatography (petroleum ether/ethyl acetate = 10/1 as the eluent) as a white solid (50% yield). ^{1}H NMR (400 MHz, Chloroform-*d*) δ 8.01 (d, *J* = 8.8 Hz, 1H), 7.53 (d, *J* = 7.9 Hz, 2H), 7.39 (t, *J* = 7.8 Hz, 2H), 7.31 – 7.11 (m, 6H), 6.82 (dd, *J* = 8.8, 2.2 Hz, 1H), 6.57 (d, *J* = 1.8 Hz, 1H), 3.83 (s, 3H), 2.56 (dd, *J* = 17.8, 5.9 Hz, 4H). ^{13}C NMR (101 MHz, Chloroform-*d*) δ 184.86 (d, *J* = 13.5 Hz), 163.87, 162.12 (d, *J* = 30.5 Hz), 156.01 (d, *J* = 276.2 Hz), 145.78, 141.88, 141.44, 130.23, 129.17, 129.04, 128.02, 127.85, 126.83, 126.48 (d, *J* = 4.9 Hz), 126.37, 118.29 (d, *J* =

12.9 Hz), 113.43, 112.52, 55.59, 28.54, 22.91 (d, J = 5.7 Hz). ^{19}F NMR (376 MHz, Chloroform-d) δ -101.15. HRMS (ESI) (m/z): $[M+Na]^+$ calcd. for $C_{25}H_{20}FO_3NNa$: 424.1319, found: 424.1306.

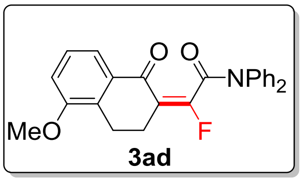

The product **3ad** is purified with silica gel chromatography (petroleum ether/ethyl acetate = 10/1 as the eluent) as a white solid (47% yield). ^{1}H NMR (400 MHz, Chloroform-d) δ 7.70 – 7.62 (m, 1H), 7.58 – 7.46 (m, 2H), 7.44 – 7.34 (m, 2H), 7.31 – 7.09 (m, 7H), 7.02 – 6.95 (m, 1H), 3.81 (s, 3H), 2.76 – 2.38 (m, 4H). ^{13}C NMR (101 MHz, Chloroform-d) δ 186.36 (d, J = 13.7 Hz), 162.04 (d, J = 30.5 Hz), 157.57, 156.36, 154.82, 141.79, 141.39, 133.87 (d, J = 4.5 Hz), 132.41, 129.20, 129.07, 128.07, 127.77, 127.25, 126.90, 126.39, 119.35, 118.28, 118.14, 114.72, 55.86, 22.17, 20.90. ^{19}F NMR (376 MHz, Chloroform-d) δ -100.51. HRMS (ESI) (m/z): $[M+Na]^+$ calcd. for $C_{25}H_{21}FO_3NNa$: 402.1505, found: 402.1508.

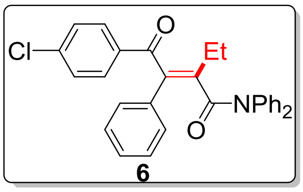

The product **6** is purified with silica gel chromatography (petroleum ether/ethyl acetate = 6/1 as the eluent) as a white solid (51% yield, E/Z>20:1). ^{1}H NMR (400 MHz, Chloroform-d) δ 7.44 (d, J = 8.3 Hz, 2H), 7.38 – 7.11 (m, 15H), 6.60 (d, J = 7.6 Hz, 2H), 2.58 – 2.07 (m, 2H), 1.24 (t, J = 7.5 Hz, 3H). ^{13}C NMR (101 MHz, Chloroform-d) δ 195.24, 169.48, 142.29, 141.89, 140.27, 138.78, 138.55, 135.68, 134.88, 131.22, 129.20, 129.10, 129.02, 128.85, 128.62, 128.32, 127.62, 126.60, 126.38, 27.21, 13.90. HRMS (ESI) (m/z): $[M+H]^+$ calcd. for $C_{30}H_{25}ClNO_2$: 446.1574, found: 446.1571.

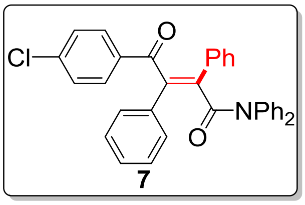

The product **7** is purified with silica gel chromatography (petroleum ether/ethyl acetate = 6/1 as the eluent) as a yellow liquid (56% yield, E/Z>20:1). ^{1}H NMR (400 MHz, Chloroform-d) δ 7.69 – 7.59 (m, 4H), 7.47 – 7.26 (m, 8H), 7.22 – 7.12 (m, 8H), 7.08 (dd, J = 4.9, 2.8 Hz, 2H), 6.53 – 6.46 (m, 2H). ^{13}C NMR (101 MHz, Chloroform-d) δ 195.33, 168.16, 142.07, 141.23, 139.81, 139.11, 137.95, 135.54, 134.93, 134.14, 130.89, 129.23, 129.19, 129.07, 128.98, 128.81, 128.59, 128.54, 128.29, 128.03, 126.63, 126.27. HRMS (ESI) (m/z): $[M+H]^+$ calcd. for $C_{34}H_{25}ClNO_2$: 514.1568, found: 514.1564.

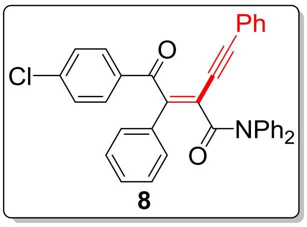

The product **8** is purified with silica gel chromatography (petroleum ether/ethyl acetate = 6/1 as the eluent) as a yellow liquid (62% yield, E/Z>20:1). ^{1}H NMR (400 MHz, Chloroform-d) δ 7.77 (d, J = 8.5 Hz, 2H), 7.57 (d, J = 6.5 Hz, 4H), 7.41 – 7.13 (m, 18H). ^{13}C NMR (101 MHz, Chloroform-d) δ 195.42, 166.69, 150.93, 142.69, 139.56, 135.03, 134.50, 131.54, 131.53, 129.60, 129.19, 129.06, 128.85, 128.66, 128.64, 128.39, 128.02, 126.84, 123.76, 122.44, 99.36, 84.83. HRMS (ESI) (m/z): $[M+H]^+$ calcd. for $C_{36}H_{25}ClNO_2$: 538.1568, found: 538.1565.

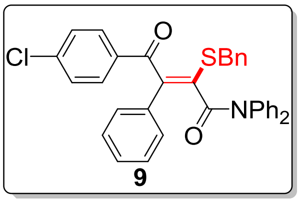

The product **9** is purified with silica gel chromatography (petroleum ether/ethyl acetate = 6/1 as the eluent) as a yellow liquid (71% yield, Z/E>20:1). ^{1}H NMR (400 MHz, Chloroform-d) δ 7.44 – 7.07 (m, 22H), 6.96 – 6.87 (m, 2H), 4.13 (s, 2H). ^{13}C NMR (101 MHz, Chloroform-d) δ 192.00, 165.49, 144.33, 142.62, 142.22, 138.67, 138.45, 136.47, 135.83, 135.47, 131.24, 129.73, 129.41, 129.16, 129.14, 128.85, 128.64, 128.56, 128.18, 127.84, 127.66, 127.60, 127.02, 126.84, 37.64. HRMS (ESI) (m/z): $[M+Na]^+$ calcd. for $C_{35}H_{26}ClNO_2SNa$: 582.1265, found: 582.1263.

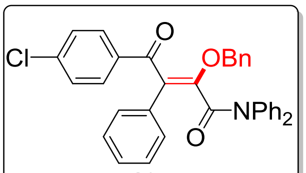

10

The product **10** is purified with silica gel chromatography (petroleum ether/ethyl acetate = 6/1 as the eluent) as a yellow liquid (53% yield, Z/E>20:1). ^{1}H NMR (400 MHz, Chloroform-*d*) δ 7.63 – 7.04 (m, 22H), 7.03 – 6.91 (m, 2H), 5.08 (s, 2H). ^{13}C NMR (101 MHz, Chloroform-*d*) δ 193.88, 164.83, 154.38, 142.31, 142.04, 138.34, 136.16, 135.73, 133.84, 131.25, 129.59, 129.30 (d, *J* = 7.4 Hz), 128.63, 128.57, 128.37, 128.33, 128.19, 127.87, 127.59, 127.23, 126.92, 126.71 (d, *J* = 4.0 Hz), 125.67, 73.35. HRMS (ESI) (m/z): $[M+Na]^+$ calcd. for $C_{35}H_{27}ClNO_3Na$:544.1679, found: 544.1669.

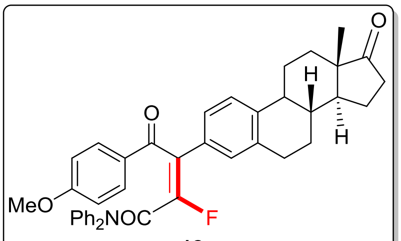

12

The product **12** is purified with silica gel chromatography (petroleum ether/ethyl acetate = 6/1 as the eluent) as a white solid (51% yield, E/Z>20:1). ^{1}H NMR (400 MHz, Chloroform-*d*) δ 7.79 (d, *J* = 8.4 Hz, 2H), 7.36 – 6.96 (m, 13H), 6.78 (d, *J* = 8.2 Hz, 2H), 3.73 (s, 3H), 2.73 (d, *J* = 4.9 Hz, 2H), 2.41 (dd, *J* = 18.9, 8.7 Hz, 1H), 2.32 – 1.80 (m, 6H), 1.60 – 1.25 (m, 6H), 0.79 (s, 3H). ^{13}C NMR (101 MHz, Chloroform-*d*) δ 191.39 (d, *J* = 6.9 Hz), 163.76, 162.38 (d, *J* = 28.8 Hz), 150.04 (d, *J* = 285.3 Hz), 142.12, 141.06, 137.09, 132.32, 129.77 (d, *J* = 3.8 Hz), 129.35, 129.24, 129.17, 129.12, 128.79, 127.66 – 126.61 (m), 126.27 (d, *J* = 5.4 Hz), 125.84, 113.96, 55.58, 50.60, 48.03, 44.53, 37.96, 35.94, 31.64, 29.40, 26.42, 25.58, 21.67, 13.92. ^{19}F NMR (376 MHz, Chloroform-*d*) δ -115.32. HRMS (ESI) (*m/z*): $[M+H]^+$ calcd. for $C_{41}H_{39}NO_4F$:628.2858, found: 628.2861.

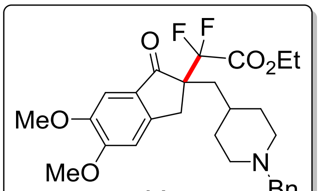

14a

The product **14a** is purified with silica gel chromatography (petroleum ether/ethyl acetate = 1/1 as the eluent) as a white solid (80% yield). ^{1}H NMR (400 MHz, Chloroform-*d*) δ 7.34 – 7.18 (m, 5H), 7.15 (s, 1H), 6.86 (s, 1H), 4.19 (q, *J* = 7.1 Hz, 2H), 3.98 (s, 3H), 3.90 (s, 3H), 3.53 – 3.34 (m, 3H), 3.04 (d, *J* = 17.8 Hz, 1H), 2.72 (dd, *J* = 23.2, 11.3 Hz, 2H), 2.08 (dt, *J* = 17.6, 8.8 Hz, 1H), 1.94 – 1.67 (m, 4H), 1.50 (d, *J* = 12.5 Hz, 1H), 1.37 (t, *J* = 11.1 Hz, 2H), 1.28 – 1.15 (m, 4H). ^{13}C NMR (101 MHz, Chloroform-*d*) δ 200.72 (t, *J* = 2.7 Hz), 162.98 (t, *J* = 32.8 Hz), 156.15, 149.89, 147.76, 138.35, 129.31, 128.22, 127.03, 116.22 (t, *J* = 259.2 Hz), 107.12, 104.61, 63.39, 63.12, 57.75 (t, *J* = 20.5 Hz), 56.44, 56.24, 53.68, 53.60, 37.34, 34.11, 33.60 (t, *J* = 3.2 Hz), 33.50, 32.22, 13.88. ^{19}F NMR (376 MHz, Chloroform-*d*) δ -109.11 (d, *J* = 257.5 Hz, 1F), -110.24 (d, *J* = 257.4 Hz, 1F). HRMS (ESI) (*m/z*): $[M+H]^+$ calcd. for $C_{28}H_{34}F_2NO_5$:502.2400, found: 502.2419.

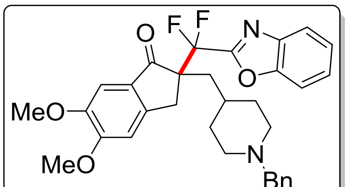

14b

The product **14b** is purified with silica gel chromatography (petroleum ether/ethyl acetate = 1/1 as the eluent) as a white solid (83% yield). ^{1}H NMR (400 MHz, Chloroform-*d*) δ 7.68 (d, *J* = 7.7 Hz, 1H), 7.49 (d, *J* = 7.9 Hz, 1H), 7.31 (dt, *J* = 15.8, 7.4 Hz, 2H), 7.16 (m, 5H), 7.06 (s, 1H), 6.77 (s, 1H), 3.87 (s, 3H), 3.80 (s, 3H), 3.60 (d, *J* = 17.7 Hz, 1H), 3.31 (s, 2H), 3.08 (d, *J* = 17.8 Hz, 1H), 2.64 (dd, *J* = 27.6, 11.7 Hz, 2H), 2.19 (dd, *J* = 14.1, 6.3 Hz, 1H), 1.93 (dd, *J* = 14.2, 3.4 Hz, 1H), 1.69 (dt, *J* = 43.1, 12.0 Hz, 2H), 1.43 (d, *J* = 12.4 Hz, 1H), 1.38 – 1.06 (m, 4H). ^{13}C NMR (101 MHz, Chloroform-*d*) δ 200.50, 156.80 (t, *J* = 34.1 Hz), 156.12, 150.42, 149.79, 147.87, 140.01, 138.30, 129.46, 129.26, 128.19, 127.01, 126.77, 125.26, 121.32, 117.44 (t, *J* = 251.5 Hz), 111.43, 107.08, 104.67, 63.30, 58.86 (t, *J* = 21.5 Hz), 56.37, 56.17, 53.64, 53.56, 37.56, 34.00, 33.95, 33.44, 32.24; ^{19}F NMR (376 MHz, Chloroform-*d*) δ -101.79 (d, *J* = 272.1 Hz, 1F), -105.06 (d, *J* =

272.2 Hz, 1F). HRMS (ESI) (m/z): $[M+H]^+$ calcd. for $C_{32}H_{33}F_2N_2O_4$: 547.2408, found: 547.2411.

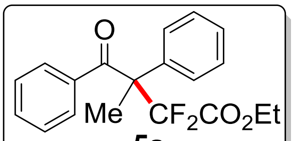

5a

The product **5a** is purified with silica gel chromatography (petroleum ether/ethyl acetate = 10/1 as the eluent) as a yellow oil (95% yield). ^{1}H NMR (400 MHz, Chloroform-*d*) δ 7.48 (d, *J* = 8.0 Hz, 2H), 7.44 – 7.38 (m, 6H), 7.29 – 7.20 (m, 2H), 4.41 – 4.29 (m, 2H), 2.12 (s, 3H), 1.36 (t, *J* = 7.1 Hz, 3H). ^{13}C NMR (101 MHz, Chloroform-*d*) δ 199.36 (d, *J* = 4.6 Hz), 163.98 (t, *J* = 32.3 Hz), 136.00, 134.55, 132.95, 130.24, 129.43, 128.69, 128.44, 128.28, 114.66 (dd, *J* = 262.6, 252.8 Hz), 62.72, 61.90 (dd, *J* = 22.8, 20.0 Hz), 20.45 (dd, *J* = 5.9, 3.3 Hz), 13.96. ^{19}F NMR (376 MHz, Chloroform-*d*) δ -106.64 (d, *J* = 270.4 Hz, 1F), -111.89 (d, *J* = 270.5 Hz, 1F). HRMS (ESI) (*m/z*): $[M+H]^+$ calcd. for $C_{19}H_{19}O_3F_2$: 333.1297, found: 333.1294.

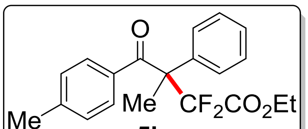

5b

The product **5b** is purified with silica gel chromatography (petroleum ether/ethyl acetate = 10/1 as the eluent) as a yellow oil (68% yield). ^{1}H NMR (400 MHz, Chloroform-*d*) δ 7.41 – 7.35 (m, 7H), 7.05 (d, *J* = 8.1 Hz, 2H), 4.41 – 4.30 (m, 2H), 2.31 (s, 3H), 2.13 (s, 3H), 1.37 (t, *J* = 7.1 Hz, 3H). ^{13}C NMR (101 MHz, Chloroform-*d*) δ 198.94 (d, *J* = 4.6 Hz), 164.06 (t, *J* = 32.3 Hz), 143.89, 136.33, 131.84, 130.48, 129.51, 128.98, 128.59, 128.36 ,114.72 (dd, *J* = 262.5, 252.5 Hz), 62.67, 61.95 (dd, *J* = 22.9, 19.9 Hz), 21.65, 20.58 (dd, *J* = 6.0, 3.3 Hz), 13.97. ^{19}F NMR (376 MHz, Chloroform-*d*) δ -106.79 (d, *J* = 270.0 Hz, 1F), -112.07 (d, *J* = 270.0 Hz, 1F). HRMS (ESI) (*m/z*): $[M+H]^+$ calcd. for $C_{20}H_{21}O_3F_2$: 347.1453, found: 347.1455.

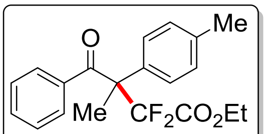

5c

The product **5c** is purified with silica gel chromatography (petroleum ether/ethyl acetate = 10/1 as the eluent) as a yellow oil (63% yield). ^{1}H NMR (400 MHz, Chloroform-*d*) δ 7.42 – 7.40 (m, 2H), 7.36 – 7.30 (m, 1H), 7.21 – 7.14 (m, 4H), 7.09 (d, *J* = 8.1 Hz, 2H), 4.32 – 4.21 (m, 2H), 2.28 (s, 3H), 2.01 (s, 3H), 1.28 (t, *J* = 7.1 Hz, 3H). ^{13}C NMR (101 MHz, Chloroform-*d*) δ 199.54 (d, *J* = 4.4 Hz), 164.05 (t, *J* = 32.4 Hz), 138.56, 134.62, 132.88, 130.23, 129.23, 129.18, 128.25, 114.67 (dd, *J* = 262.1, 252.5 Hz), 62.68, 61.61 (dd, *J* = 22.9, 20.1 Hz), 21.25, 20.40 (dd, *J* = 6.0, 3.3 Hz), 13.96. ^{19}F NMR (376 MHz, Chloroform-*d*) δ -106.68 (d, *J* = 270.2 Hz, 1F), -111.90 (d, *J* = 270.2 Hz, 1F). HRMS (ESI) (*m/z*): $[M+H]^+$ calcd. for $C_{20}H_{21}O_3F_2$: 347.1453, found: 347.1458.

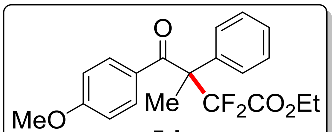

5d

The product **5d** is purified with silica gel chromatography (petroleum ether/ethyl acetate = 10/1 as the eluent) as a yellow oil (85% yield). ^{1}H NMR (400 MHz, Chloroform-*d*) δ 7.50 (d, *J* = 8.9 Hz, 2H), 7.45 – 7.29 (m, 5H), 6.72 (d, *J* = 8.9 Hz, 2H), 4.41 – 4.30 (m, 2H), 3.78 (s, 3H), 2.13 (s, 3H), 1.37 (t, *J* = 7.1 Hz, 3H). ^{13}C NMR (101 MHz, Chloroform-*d*) δ 197.86 (d, *J* = 4.8 Hz), 164.12 (t, *J* = 32.3 Hz), 163.25, 136.53 (d, *J* = 2.1 Hz), 132.82, 129.53, 128.55, 128.31, 126.82, 114.68 (dd, *J* = 262.5, 251.9 Hz), 113.48, 62.66, 61.89 (dd, *J* = 22.9, 20.0 Hz), 55.52 , 20.75 (dd, *J* = 6.0, 3.3 Hz), 13.98. ^{19}F NMR (376 MHz, Chloroform-*d*) δ -106.96 (d, *J* = 269.5 Hz, 1F), -112.27 (d, *J* = 269.5 Hz, 1F). HRMS (ESI) (*m/z*): $[M+H]^+$ calcd. for $C_{20}H_{21}O_4F_2$: 363.1402, found: 363.1398.

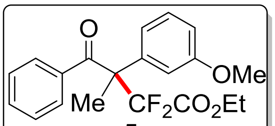

5e

The product **5e** is purified with silica gel chromatography (petroleum ether/ethyl acetate = 10/1 as the eluent) as a yellow oil (66% yield). ^{1}H NMR (400 MHz, Chloroform-*d*) δ 7.51 – 7.49 (m, 2H), 7.45 – 7.39 (m, 1H), 7.30 – 7.23 (m, 3H), 6.98 – 6.91 (m, 3H), 4.41 – 4.29 (m, 2H), 3.77 (s, 3H), 2.10 (s, 3H), 1.36 (t, *J* =

7.1 Hz, 3H). ^{13}C NMR (101 MHz, Chloroform-*d*) δ 199.20 (d, *J* = 4.7 Hz), 163.94 (t, *J* = 32.3 Hz), 159.56, 137.39, 134.68, 132.91, 130.17, 129.29, 128.27, 122.10, 115.69,114.65 (dd, *J* = 263.3, 252.6 Hz), 113.71, 62.71, 61.83 (dd, *J* = 22.9, 19.8 Hz), 55.39, 20.47 (dd, *J* = 6.0, 3.3 Hz), 13.95. ^{19}F NMR (376 MHz, Chloroform-*d*) δ -106.45 (d, *J* = 270.2 Hz, 1F), -111.73 (d, *J* = 270.2 Hz, 1F). HRMS (ESI) (*m/z*): $[M+H]^+$ calcd. for $C_{20}H_{21}O_4F_2$: 363.1402, found: 363.1402.

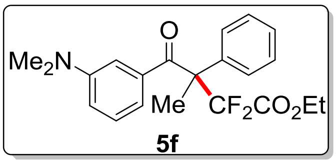

The product **5f** is purified with silica gel chromatography (petroleum ether/ethyl acetate = 10/1 as the eluent) as a yellow oil (60% yield). ^{1}H NMR (400 MHz, Chloroform-*d*) δ 7.41 – 7.36 (m, 5H), 7.10 – 7.02 (m, 1H), 6.90 (s, 1H), 6.78 – 6.72 (m, 2H), 4.36 (q, *J* = 7.0 Hz, 2H), 2.79 (s, 6H), 2.13 (s, 3H), 1.37 (t, *J* = 7.1 Hz, 3H). ^{13}C NMR (101 MHz, Chloroform-*d*) δ 200.02 (d, *J* = 4.4 Hz), 164.07 (t, *J* = 32.3 Hz), 150.16 , 136.53 , 135.13 , 129.52 , 128.76 , 128.51 , 128.30 , 118.40 , 116.70 , 114.80 (dd, *J* = 262.1, 252.5 Hz), 114.02 , 62.66 , 62.10 (dd, *J* = 22.8, 19.9 Hz), 40.36 , 20.53 (dd, *J* = 6.0, 3.4 Hz), 13.98. ^{19}F NMR (376 MHz, Chloroform-*d*) δ -106.77 (d, *J* = 269.9 Hz, 1F), -111.85 (d, *J* = 269.9 Hz, 1F). HRMS (ESI) (*m/z*): $[M+H]^+$ calcd. for $C_{21}H_{24}O_3NF_2$: 376.1719, found: 376.1720.

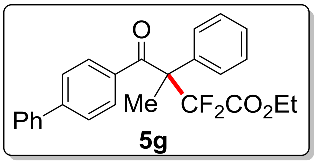

The product **5g** is purified with silica gel chromatography (petroleum ether/ethyl acetate = 10/1 as the eluent) as a yellow oil (61% yield). ^{1}H NMR (400 MHz, Chloroform-*d*) δ 7.61 – 7.57 (m, 2H), 7.55 – 7.53 (m, 2H), 7.51 – 7.47 (m, 2H), 7.45 – 7.35 (m, 8H), 4.44 – 4.32 (m, 2H), 2.19 (s, 3H), 1.39 (t, *J* = 7.1 Hz, 3H). ^{13}C NMR (101 MHz, Chloroform-*d*) δ 198.85 (d, *J* = 4.7 Hz), 164.02 (t, *J* = 32.3 Hz), 145.59 , 139.67 , 136.19 , 133.14 , 130.93 , 129.52 , 129.04 , 128.70 , 128.48 , 128.42 , 127.28 , 126.86 , 114.71 (dd, *J* = 262.6, 252.8 Hz), 62.72 , 61.99 (dd, *J* = 22.9, 20.0 Hz), 20.54 (dd, *J* = 6.0, 3.3 Hz), 13.98. ^{19}F NMR (376 MHz, Chloroform-*d*) δ -106.64 (d, *J* = 270.3 Hz, 1F), -111.93 (d, *J* = 270.3 Hz, 1F). HRMS (ESI) (*m/z*): $[M+H]^+$ calcd. for $C_{25}H_{23}O_3F_2$: 409.1610, found: 409.1607.

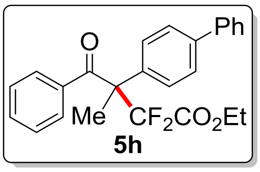

The product **5h** is purified with silica gel chromatography (petroleum ether/ethyl acetate = 10/1 as the eluent) as a yellow oil (63% yield). ^{1}H NMR (400 MHz, Chloroform-*d*) δ 7.64 – 7.51 (m, 4H), 7.49 – 7.44 (m, 2H), 7.41 – 7.35 (m, 5H), 7.32 – 7.27 (m, 1H), 7.21 – 7.17 (m, 2H), 4.35 – 4.23 (m, 2H), 2.08 (s, 3H), 1.30 (t, *J* = 7.1 Hz, 3H). ^{13}C NMR (101 MHz, Chloroform-*d*) δ 199.37 (d, *J* = 4.4 Hz), 163.99 (t, *J* = 32.3 Hz), 141.33, 140.17, 134.97, 134.55, 133.02, 130.28, 129.83, 128.96, 128.34, 127.78, 127.18, 127.02, 114.70 (dd, *J* = 262.4, 252.9 Hz), 62.78, 61.73 (dd, *J* = 22.8, 20.1 Hz), 20.49 (dd, *J* = 6.0, 3.2 Hz), 13.99. ^{19}F NMR (376 MHz, Chloroform-*d*) δ -106.57 (d, *J* = 270.4 Hz, 1F), -111.70 (d, *J* = 270.4 Hz, 1F). HRMS (ESI) (*m/z*): $[M+H]^+$ calcd. for $C_{25}H_{23}O_3F_2$: 409.1610, found: 409.1606.

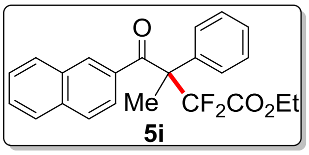

The product **5i** is purified with silica gel chromatography (petroleum ether/ethyl acetate = 10/1 as the eluent) as a yellow oil (64% yield). ^{1}H NMR (400 MHz, Chloroform-*d*) δ 8.05 (s, 1H), 7.78 (d, *J* = 8.1 Hz, 1H), 7.73 – 7.66 (m, 2H), 7.57 – 7.52 (m, 2H), 7.49 – 7.44 (m, 3H), 7.41 – 7.37 (m, 3H), 4.45 – 4.33 (m, 2H), 2.21 (s, 3H), 1.40 (t, *J* = 7.1 Hz, 3H). ^{13}C NMR (101 MHz, Chloroform-*d*) δ 199.28 (d, *J* = 4.7 Hz), 164.05 (t, *J* = 32.2 Hz), 136.26 (d, *J* = 2.0 Hz), 135.25 , 132.43 , 132.21 , 131.82 , 129.82 , 129.55 , 128.85 , 128.73 , 128.50 , 127.92 , 127.67 , 126.82 , 125.59 , 114.76 (dd, *J* = 262.7, 252.7 Hz), 62.74 , 62.12 (dd, *J* = 22.9, 20.0 Hz), 20.59 (dd, *J* = 5.9, 3.4 Hz), 14.00. ^{19}F NMR (376 MHz, Chloroform-*d*) δ -106.64 (d, *J* = 270.3 Hz, 1F), -111.93 (d, *J* = 270.3 Hz, 1F). HRMS (ESI) (*m/z*): $[M+H]^+$ calcd. for

$C_{23}H_{21}O_3F_2$: 383.1453, found: 383.1451.

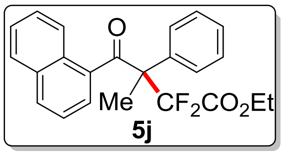

5j

The product **5j** is purified with silica gel chromatography (petroleum ether/ethyl acetate = 10/1 as the eluent) as a yellow oil (71% yield). ^{1}H NMR (400 MHz, Chloroform-*d*) δ 8.18 (d, *J* = 8.4 Hz, 1H), 7.90 – 7.78 (m, 1H), 7.61 – 7.45 (m, 7H), 7.17 – 7.09 (m, 1H), 6.86 (d, *J* = 7.2 Hz, 1H), 4.46 – 4.32 (m, 2H), 1.94 (s, 3H), 1.37 (t, *J* = 7.0 Hz, 3H). ^{13}C NMR (101 MHz, Chloroform-*d*) δ 203.35 (d, *J* = 3.5 Hz), 163.89 (t, *J* = 32.4 Hz), 135.26, 135.12, 134.01, 131.57, 130.93, 129.29, 128.89, 128.60, 128.53, 127.83, 126.58, 125.67, 125.48, 123.85, 115.22 (dd, *J* = 263.3, 255.6 Hz), 62.97, 62.52 (dd, *J* = 22.1, 19.2 Hz), 19.21 (dd, *J* = 5.7, 3.5 Hz), 13.97. ^{19}F NMR (376 MHz, Chloroform-*d*) δ -104.68 (d, *J* = 272.9 Hz, 1F), -108.53 (d, *J* = 272.9 Hz, 1F). HRMS (ESI) (*m/z*): $[M+H]^+$ calcd. for $C_{23}H_{21}O_3F_2$: 383.1453, found: 383.1457.

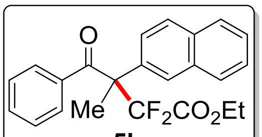

5k

The product **5k** is purified with silica gel chromatography (petroleum ether/ethyl acetate = 10/1 as the eluent) as a yellow oil (62% yield). ^{1}H NMR (400 MHz, Chloroform-*d*) δ 7.98 (s, 1H), 7.87 – 7.81 (m, 3H), 7.64 – 7.48 (m, 4H), 7.46 – 7.39 (m, 2H), 7.25 – 7.15 (m, 2H), 4.43 – 4.31 (m, 2H), 2.25 (s, 3H), 1.36 (t, *J* = 7.1 Hz, 3H). ^{13}C NMR (101 MHz, Chloroform-*d*) δ 199.44 (d, *J* = 4.4 Hz), 164.03 (t, *J* = 32.2 Hz), 134.67, 133.62 (d, *J* = 2.1 Hz), 133.15, 133.11, 133.00, 130.24, 128.54, 128.35, 127.89, 127.71, 127.32, 127.30, 126.93, 126.55, 114.85 (dd, *J* = 262.6, 252.8 Hz), 62.77, 62.07 (dd, *J* = 22.8, 20.0 Hz), 20.67 (dd, *J* = 6.0, 3.3 Hz), 13.97. ^{19}F NMR (376 MHz, Chloroform-*d*) δ -106.33 (d, *J* = 277.2 Hz, 1F), -111.49 (d, *J* = 270.7 Hz, 1F). HRMS (ESI) (*m/z*): $[M+H]^+$ calcd. for $C_{23}H_{21}O_3F_2$: 383.1453, found: 383.1453.

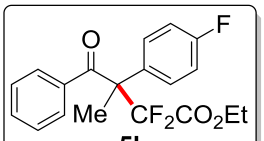

5l

The product **5l** is purified with silica gel chromatography (petroleum ether/ethyl acetate = 10/1 as the eluent) as a yellow oil (63% yield). ^{1}H NMR (400 MHz, Chloroform-*d*) δ 7.49 – 7.47 (m, 2H), 7.44 (d, *J* = 7.4 Hz, 1H), 7.39 – 7.36 (m, 2H), 7.29 – 7.25 (m, 2H), 7.12 – 7.03 (m, 2H), 4.42 - 4.31 (m, 2H), 2.11 (s, 3H), 1.38 (t, *J* = 7.1 Hz, 3H). ^{13}C NMR (101 MHz, Chloroform-*d*) δ 199.09 (d, *J* = 4.4 Hz), 163.87 (t, *J* = 32.3), 162.96 (d, *J* = 249.5), 134.38, 133.12, 131.89, 131.24 (dd, *J* = 8.2, 1.3 Hz), 130.22, 128.40, 115.52 (d, *J* = 21.5 Hz), 114.52 (dd, *J* = 262.3, 253.1 Hz), 62.81, 61.44 (dd, *J* = 22.9, 20.1 Hz), 20.64 (dd, *J* = 6.1, 3.2 Hz), 13.99. ^{19}F NMR (376 MHz, Chloroform-*d*) δ -106.83 (d, *J* = 271.0 Hz, 1F), -112.03 (d, *J* = 271.0 Hz, 1F), -113.10 (s, 1F). HRMS (ESI) (*m/z*): $[M+H]^+$ calcd. for $C_{19}H_{18}O_3F_3$: 351.1203, found: 351.1205.

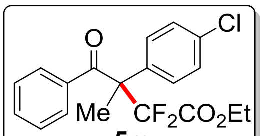

5m

The product **5m** is purified with silica gel chromatography (petroleum ether/ethyl acetate = 10/1 as the eluent) as a yellow oil (60% yield). ^{1}H NMR (400 MHz, Chloroform-*d*) δ 7.50 – 7.43 (m, 3H), 7.37 – 7.23 (m, 6H), 4.42 – 4.30 (m, 2H), 2.11 (s, 3H), 1.37 (t, *J* = 7.1 Hz, 3H). ^{13}C NMR (101 MHz, Chloroform-*d*) δ 198.91 (d, *J* = 4.4 Hz), 163.78 (t, *J* = 32.1 Hz), 134.97, 134.68 (d, J = 2.1 Hz), 134.23 (d, *J* = 2.1 Hz), 133.19, 130.81, 130.21, 128.69, 128.42, 114.44 (dd, *J* = 262.4, 253.4 Hz), 62.84, 61.54 (dd, *J* = 22.9, 20.0 Hz), 20.51 (dd, *J* = 6.0, 3.2 Hz), 13.97. ^{19}F NMR (376 MHz, Chloroform-*d*) δ -106.70 (d, *J* = 271.3 Hz, 1F), -111.83 (d, *J* = 271.2 Hz, 1F). HRMS (ESI) (*m/z*): $[M+H]^+$ calcd. for $C_{19}H_{18}O_3ClF_2$: 367.0907, found: 367.0907.

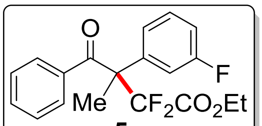

5n

The product **5n** is purified with silica gel chromatography (petroleum ether/ethyl acetate = 10/1 as the eluent) as a yellow oil (60% yield). ^{1}H NMR (400 MHz, Chloroform-*d*) δ 7.45 – 7.39 (m, 2H), 7.37 (d, *J* = 7.4 Hz, 1H), 7.26 (q, *J* = 7.7 Hz, 1H), 7.23 – 7.17 (m, 2H), 7.08 (d, *J* = 8.4 Hz, 2H), 7.06 – 6.99 (m, 1H), 4.29 (dtt,

J = 10.7, 6.9, 3.6 Hz, 2H), 2.04 (s, 3H), 1.30 (t, J = 7.1 Hz, 3H). ^{13}C NMR (101 MHz, Chloroform-*d*) δ 198.74 (d, J = 4.4 Hz), 163.75 (t, J = 32.3 Hz), 162.75 (d, J = 247.4 Hz), 138.50 (dd, J = 7.2, 1.6 Hz), 134.28 , 133.19 , 130.19 , 129.80 (d, J = 8.2 Hz), 128.42 , 125.67 – 125.43 (m), 116.52 (d, J = 22.9 Hz), 115.80 (d, J = 20.9 Hz), 114.45 (dd, J = 263.1, 253.3 Hz), 62.86 , 61.72 (dd, J = 22.8, 19.7 Hz), 20.50 (dd, J = 6.0, 3.3 Hz), 13.97. ^{19}F NMR (376 MHz, Chloroform-*d*) δ -106.82 (d, J = 270.7 Hz, 1F), 112.02 (d, J = 270.7 Hz, 1F), -113.09 (s, 1F). HRMS (ESI) (*m/z*): $[M+H]^+$ calcd. for $C_{19}H_{18}O_3F_3$: 351.1203, found: 351.1205.

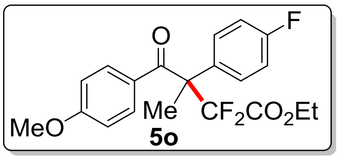

5o

The product **5o** is purified with silica gel chromatography (petroleum ether/ethyl acetate = 10/1 as the eluent) as a yellow oil (70% yield). ^{1}H NMR (400 MHz, Chloroform-*d*) δ 7.50 (d, J = 8.8 Hz, 2H), 7.37 – 7.34 (m, 2H), 7.11 – 7.00 (m, 2H), 6.74 (d, J = 8.9 Hz, 2H), 4.42 – 4.31 (m, 2H), 3.79 (s, 3H), 2.13 (s, 3H), 1.38 (t, J = 7.1 Hz, 3H). ^{13}C NMR (101 MHz, Chloroform-*d*) δ 197.55 (d, J = 4.8 Hz), 164.01 (t, J = 32.3 Hz), 163.42, 162.86 (d, J = 249.5 Hz), 132.81, 132.42, 131.32 (d, J = 8.0 Hz), 126.65, 115.48, 115.27, 114.50 (dd, J = 212.1, 254.5 Hz), 113.61, 62.73, 61.43 (dd, J = 22.9, 20.0 Hz), 55.57, 20.94 (dd, J = 6.0, 3.2 Hz), 14.00. ^{19}F NMR (376 MHz, Chloroform-*d*) δ -107.08 (d, J = 270.1 Hz, 1F), -112.37 (d, J = 270.0 Hz, 1F), -113.36 (s, 1F). HRMS (ESI) (*m/z*): $[M+H]^+$ calcd. for $C_{20}H_{20}O_4F_3$:381.1308, found: 381.1306.

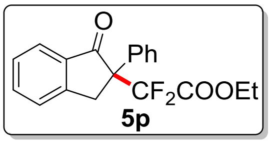

5p

The product **5p** is purified with silica gel chromatography (petroleum ether/ethyl acetate = 10/1 as the eluent) as a yellow oil (95% yield). ^{1}H NMR (400 MHz, Chloroform-*d*) δ 7.84 (d, J = 7.6 Hz, 1H), 7.63 (m, 3H), 7.39 (m, 5H), 4.19 – 4.06 (m, 3H), 3.73 (d, J = 16.9 Hz, 1H), 1.09 (t, J = 7.1 Hz, 3H). ^{13}C NMR (101 MHz, Chloroform-*d*) δ 200.32 (t, J = 2.4 Hz), 162.74 (t, J = 32.7 Hz), 151.51, 135.89, 135.79, 134.99, 128.97, 128.52, 128.41, 128.09, 126.26, 124.91, 115.43 (t, J = 260.6 Hz), 63.12, 61.38 (t, J = 21.2 Hz), 38.13 (t, J = 3.5 Hz), 13.66. ^{19}F NMR (376 MHz, Chloroform-*d*) δ -105.43 (d, J = 266.9 Hz, 1F), -107.22 (d, J = 263.2 Hz, 1F). HRMS (ESI) (*m/z*): $[M+H]^+$ calcd. for $C_{19}H_{17}O_3F_2$: 331.1140, found: 331.1144.

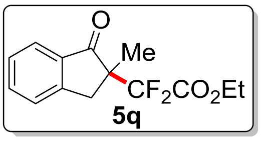

5q

The product **5q** is purified with silica gel chromatography (petroleum ether/ethyl acetate = 10/1 as the eluent) as a yellow oil (83% yield). ^{1}H NMR (400 MHz, Chloroform-*d*) δ 7.78 (d, J = 7.6 Hz, 1H), 7.66 – 7.58 (m, 1H), 7.46 (d, J = 7.6 Hz, 1H), 7.43 – 7.37 (m, 1H), 4.21 (q, J = 7.1 Hz, 2H), 3.59 (d, J = 17.6 Hz, 1H), 2.94 (d, J = 17.7 Hz, 1H), 1.49 (s, 3H), 1.20 (t, J = 7.1 Hz, 3H). ^{13}C NMR (101 MHz, Chloroform-*d*) δ 202.57 (t, J = 2.7 Hz), 162.91 (t, J = 32.8 Hz), 151.49, 135.60, 135.34 (t, J = 2.1 Hz), 128.09, 126.60, 124.85, 115.93 (t, J = 257.8 Hz), 63.17, 53.77 (t, J = 21.7 Hz), 37.06, 18.96 (t, J = 4.6 Hz), 13.80. ^{19}F NMR (376 MHz, Chloroform-*d*) δ -109.86 (d, J = 264.1 Hz, 1F), -110.58 (d, J = 264.2 Hz, 1F). HRMS (ESI) (*m/z*): $[M+H]^+$ calcd. for $C_{14}H_{15}O_3F_2$: 269.0984, found: 269.0994.

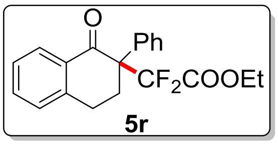

5r

The product **5r** is purified with silica gel chromatography (petroleum ether/ethyl acetate = 10/1 as the eluent) as a yellow oil (90% yield). ^{1}H NMR (400 MHz, Chloroform-*d*) δ 8.16 (d, J = 7.9, Hz, 1H), 7.49 – 7.41 (m, 1H), 7.26 – 7.34 (m, 6H), 7.10 (d, J = 7.6 Hz, 1H), 4.26 (qq, J = 10.7, 7.1 Hz, 2H), 3.01 (td, J = 13.5, 4.5 Hz, 1H), 2.92 – 2.83 (m, 2H), 2.73 – 2.64 (m, 1H), 1.22 (t, J = 7.1 Hz, 3H). ^{13}C NMR (101 MHz, Chloroform-*d*) δ 196.07 (t, J = 2.9 Hz), 163.56 (t, J = 32.4 Hz), 143.17, 134.19, 132.29, 131.92, 129.02, 128.74, 128.39, 128.36, 128.16, 127.00, 115.22 (t, J = 257.6 Hz), 62.63, 60.87 (dd, J = 22.4, 20.9 Hz),

28.51 (dd, J = 5.8, 3.2 Hz), 25.27, 13.77. ^{19}F NMR (376 MHz, Chloroform-d) δ -107.22 (d, J = 272.2 Hz, 1F), -110.19 (d, J = 272.1 Hz, 1F). HRMS (ESI) (m/z): $[M+H]^+$ calcd. for $C_{20}H_{19}O_3F_2$: 345.1297, found: 345.1301.

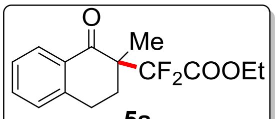

5s

The product **5s** is purified with silica gel chromatography (petroleum ether/ethyl acetate = 10/1 as the eluent) as a yellow oil (72% yield). ^{1}H NMR (400 MHz, Chloroform-d) δ 8.01 (d, J = 7.7 Hz, 1H), 7.57 – 7.45 (m, 1H), 7.36 – 7.28 (m, 1H), 7.25 (d, J = 8.0 Hz, 1H), 4.37 (q, J = 7.1 Hz, 2H), 3.14 – 3.01 (m, 2H), 2.64 – 2.56 (m, 1H), 2.11 (dt, J = 13.7, 4.5 Hz, 1H), 1.52 (s, 3H), 1.37 (t, J = 7.1 Hz, 3H). ^{13}C NMR (101 MHz, Chloroform-d) δ 196.59 (t, J = 3.1 Hz), 163.74 (t, J = 32.3 Hz), 142.87, 134.08, 131.18, 128.81, 128.23, 127.06, 116.41 (t, J = 257.2 Hz), 62.91, 51.93 (t, J = 21.9 Hz), 28.54 (t, J = 4.1 Hz), 24.86, 16.75 (t, J = 4.2 Hz), 13.98. ^{19}F NMR (376 MHz, Chloroform-d) δ -111.56 (d, J = 266.0 Hz, 1F), -112.33 (d, J = 266.0 Hz, 1F). HRMS (ESI) (m/z): $[M+H]^+$ calcd. for $C_{15}H_{17}O_3F_2$: 283.1140, found: 283.1145.

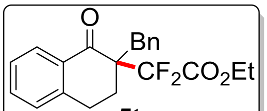

5t

The product **5t** is purified with silica gel chromatography (petroleum ether/ethyl acetate = 10/1 as the eluent) as a yellow oil (61% yield). ^{1}H NMR (400 MHz, Chloroform-d) δ 8.04 (d, J = 7.8 Hz, 1H), 7.48 – 7.42 (m, 1H), 7.33 – 7.27 (m, 1H), 7.20 – 7.14 (m, 5H), 7.16 (d, J = 5.9 Hz, 1H), 4.40 – 4.28 (m, 2H), 3.65 (d, J = 13.8 Hz, 1H), 3.12 – 3.04 (m, 1H), 3.04 (d, J = 13.8 Hz, 1H), 2.74 (dt, J = 17.1, 6.3 Hz, 1H), 2.47 – 2.41 (m, 1H), 2.20 – 2.13 (m, 1H), 1.33 (t, J = 7.1 Hz, 3H). ^{13}C NMR (101 MHz, Chloroform-d) δ 195.35 (t, J = 2.7 Hz), 163.34 (t, J = 32.5 Hz), 143.41, 135.84, 133.98, 132.49, 131.00, 128.71, 128.35, 128.15, 127.01, 126.89, 117.11 (t, J = 260.8 Hz), 63.20, 55.49 (t, J = 19.7 Hz), 37.28 (t, J = 4.0 Hz), 26.20 (t, J = 3.2 Hz), 25.42, 13.97. ^{19}F NMR (376 MHz, Chloroform-d) δ -106.66 (d, J = 262.6 Hz, 1F), -107.38 (d, J = 262.5 Hz, 1F). HRMS (ESI) (m/z): $[M+H]^+$ calcd. for $C_{21}H_{21}O_3F_2$: 359.1453, found: 359.1451.

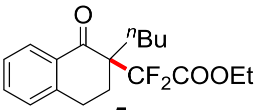

5u

The product **5u** is purified with silica gel chromatography (petroleum ether/ethyl acetate = 10/1 as the eluent) as a yellow oil (74% yield). ^{1}H NMR (400 MHz, Chloroform-d) δ 8.02 (d, J = 7.8 Hz, 1H), 7.60 – 7.44 (m, 1H), 7.40 – 7.28 (m, 1H), 7.25 (d, J = 7.9 Hz, 1H), 4.33 (q, J = 7.1 Hz, 2H), 3.08 – 2.97 (m, 2H), 2.63 – 2.55 (m, 1H), 2.35 (dt, J = 13.6, 4.7 Hz, 1H), 1.96 – 1.78 (m, 2H), 1.58 – 1.49 (m, 1H), 1.43 - 1.28 (m, 6H), 0.90 (t, J = 7.1 Hz, 3H). ^{13}C NMR (101 MHz, Chloroform-d) δ 196.82 (t, J = 3.5 Hz), 163.78 (t, J = 32.4 Hz), 143.09, 134.00, 131.39, 128.82, 128.23, 127.02, 117.02 (dd, J = 260.0, 256.6 Hz), 62.87, 55.10 (t, J = 20.5 Hz), 30.19 (t, J = 3.1 Hz), 26.64, 25.21 (dd, J = 5.3, 3.3 Hz), 25.04, 23.40, 13.97, 13.95. ^{19}F NMR (376 MHz, Chloroform-d) δ -106.55 (d, J = 269.9 Hz, 1F), -107.72 (d, J = 269.9 Hz, 1F). HRMS (ESI) (m/z): $[M+Na]^+$ calcd. for $C_{18}H_{22}O_3F_2Na$: 347.1429, found: 347.1434.

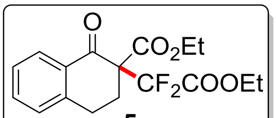

5v

The product **5v** is purified with silica gel chromatography (petroleum ether/ethyl acetate = 10/1 as the eluent) as a yellow oil (61% yield). ^{1}H NMR (400 MHz, Chloroform-d) δ 7.99 (d, J = 7.7 Hz, 1H), 7.44 (dd, J = 9.4, 5.7 Hz, 1H), 7.26 (t, J = 7.7 Hz, 1H), 7.22 – 7.12 (m, 1H), 4.34 (q, J = 7.2 Hz, 2H), 4.23 – 4.08 (m, 2H), 3.06 – 2.81 (m, 2H), 2.75 (d, J = 13.6 Hz, 1H), 2.55 (td, J = 13.3, 4.3 Hz, 1H), 1.31 (t, J = 6.8 Hz, 3H), 1.10 (t, J = 7.0 Hz, 3H). ^{13}C NMR (101 MHz, Chloroform-d) δ 191.01 (t, J = 2.1 Hz), 166.84 (dd, J = 3.5, 1.5 Hz), 163.01 (t, J = 31.2 Hz), 142.54, 134.32, 131.61, 128.81, 128.11, 127.26, 113.84 (t, J = 3.7 Hz), 63.31, 62.94 (t, J = 22.2 Hz), 62.58, 27.18 (t, J = 4.2 Hz), 25.63, 13.94. ^{19}F NMR (376 MHz, Chloroform-d) δ -110.92 (d, J = 274.5 Hz, 1F), -111.69 (d, J = 274.5 Hz, 1F). HRMS (ESI) (m/z): $[M+H]^+$

calcd. for $C_{17}H_{19}O_5F_2$: 341.1195, found: 341.1200.

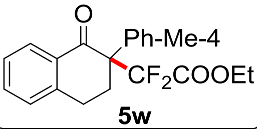

5w

The product **5w** is purified with silica gel chromatography (petroleum ether/ethyl acetate = 10/1 as the eluent) as a yellow oil (85% yield). ^{1}H NMR (400 MHz, Chloroform-*d*) δ 8.15 (d, *J* = 7.8 Hz, 1H), 7.48 – 7.39 (m, 1H), 7.35 – 7.28 (m, 1H), 7.23 – 7.16 (m, 2H), 7.12 – 7.08 (m, 3H), 4.34 – 4.21 (m, 2H), 2.99 (td, *J* = 13.4, 4.3 Hz, 1H), 2.88 – 2.84 (m, 2H), 2.76 – 2.68 (m, 1H), 2.30 (s, 3H), 1.26 (t, *J* = 7.3 Hz, 3H). ^{13}C NMR (101 MHz, Chloroform-*d*) δ 196.13 (t, *J* = 3.1 Hz), 163.64 (t, *J* = 32.3 Hz), 143.14, 138.18, 134.08, 131.90, 129.12, 128.89, 128.71, 128.09, 126.92, 115.19 (t, *J* = 258.6 Hz), 62.62, 60.55 (t, *J* = 22.2 Hz), 28.33 (dd, *J* = 5.8, 3.1 Hz), 25.27, 21.09, 13.79. ^{19}F NMR (376 MHz, Chloroform-*d*) δ -107.13 (d, *J* = 271.8 Hz, 1F), -110.44 (d, *J* = 271.7 Hz, 1F). HRMS (ESI) (*m/z*): $[M+H]^+$ calcd. for $C_{21}H_{21}O_3F_2$: 359.1453, found: 359.1459.

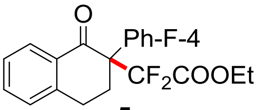

5x

The product **5x** is purified with silica gel chromatography (petroleum ether/ethyl acetate = 10/1 as the eluent) as a yellow oil (83% yield). ^{1}H NMR (400 MHz, Chloroform-*d*) δ 8.14 (d, *J* = 7.9 Hz, 1H), 7.54 – 7.41 (m, 1H), 7.34 – 7.26 (m, 3H), 7.11 (d, *J* = 7.7 Hz, 1H), 7.05 – 6.92 (m, 2H), 4.33 – 4.20 (m, 2H), 2.99 (td, *J* = 14.2, 13.6, 4.6 Hz, 1H), 2.90 – 2.81 (m, 2H), 2.71 – 2.62 (m, 1H), 1.24 (t, *J* = 7.1 Hz, 3H). ^{13}C NMR (101 MHz, Chloroform-*d*) δ 195.83 (t, *J* = 2.9 Hz), 163.47 (t, *J* = 32.3 Hz), 162.71 (d, *J* = 248.2 Hz), 143.01, 134.39, 131.71, 130.84 (d, *J* = 8.2 Hz), 128.79, 128.24, 127.95, 127.13, 115.05 (t, *J* = 258.6 Hz), 115.44 (d, *J* = 21.5 Hz), 62.75, 60.29 (dd, *J* = 22.7, 21.1 Hz), 28.49 (dd, *J* = 5.7, 3.2 Hz), 25.15, 13.8. ^{19}F NMR (376 MHz, Chloroform-*d*) δ -107.44 (d, *J* = 272.4 Hz, 1F), -110.43 (d, *J* = 272.4 Hz, 1F), -113.53 (s, 1F). HRMS (ESI) (*m/z*): $[M+H]^+$ calcd. for $C_{20}H_{18}O_3F_3$: 363.1203, found: 363.1208.

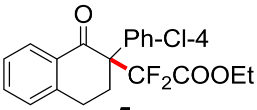

5y

The product **5y** is purified with silica gel chromatography (petroleum ether/ethyl acetate = 10/1 as the eluent) as a yellow oil (85% yield). ^{1}H NMR (400 MHz, Chloroform-*d*) δ 8.19 – 8.07 (m, 1H), 7.51 – 7.42 (m, 1H), 7.37 – 7.30 (m, 1H), 7.30 – 7.26 (m, 2H), 7.26 – 7.21 (m, 2H), 7.11 (d, *J* = 7.7 Hz, 1H), 4.33 – 4.21 (m, 2H), 3.04 – 2.95 (m, 1H), 2.90 – 2.80 (m, 2H), 2.70 – 2.61 (m, 1H), 1.25 (t, *J* = 7.1 Hz, 3H). ^{13}C NMR (101 MHz, Chloroform-*d*) δ 195.71 (t, *J* = 2.9 Hz), 163.43 (t, *J* = 32.3 Hz), 142.99, 134.62, 134.47, 131.66, 130.87, 130.43, 128.83, 128.67, 128.25, 127.18, 114.98 (t, *J* = 257.9 Hz), 62.84, 60.38 (dd, *J* = 22.7, 21.0 Hz), 28.37 (dd, *J* = 5.8, 3.2 Hz), 25.14, 13.84. ^{19}F NMR (376 MHz, Chloroform-*d*) δ -107.37 (d, *J* = 272.6 Hz, 1F), -110.35 (d, *J* = 272.6 Hz, 1F). HRMS (ESI) (*m/z*): $[M+H]^+$ calcd. for $C_{20}H_{18}O_3ClF_2$: 379.0907, found: 379.0909.

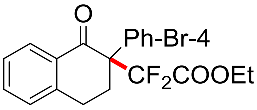

5z

The product **5z** is purified with silica gel chromatography (petroleum ether/ethyl acetate = 10/1 as the eluent) as a yellow oil (85% yield). ^{1}H NMR (400 MHz, Chloroform-*d*) δ 8.18 – 8.09 (m, 1H), 7.52 – 7.38 (m, 3H), 7.36 – 7.29 (m, 1H), 7.17 (d, *J* = 8.5 Hz, 2H), 7.11 (d, *J* = 7.7 Hz, 1H), 4.33 – 4.21 (m, 2H), 3.03 – 2.95 (m, 1H), 2.90 – 2.80 (m, 2H), 2.70 – 2.61 (m, 1H), 1.25 (t, *J* = 7.1 Hz, 3H). ^{13}C NMR (101 MHz, Chloroform-*d*) δ 195.66 (t, *J* = 2.9 Hz), 163.39 (t, *J* = 32.2 Hz), 142.97, 134.48, 131.60, 131.40, 130.72, 128.83, 128.22, 127.16, 122.84, 114.89 (t, *J* = 257.9 Hz), 62.84, 60.41 (dd, *J* = 22.7, 21.0 Hz), 28.28 (dd, *J* = 5.7, 3.3 Hz), 25.11, 13.83. ^{19}F NMR (376 MHz, Chloroform-*d*) δ -107.44 (d, *J* = 272.7 Hz, 1F), -110.39 (d, *J* = 272.6 Hz, 1F). HRMS (ESI) (*m/z*): $[M+H]^+$ calcd. for $C_{20}H_{18}O_3BrF_2$: 423.0402, found: 423.0400.

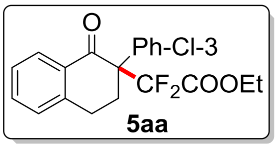

The product **5aa** is purified with silica gel chromatography (petroleum ether/ethyl acetate = 10/1 as the eluent) as a yellow oil (70% yield). ^{1}H NMR (400 MHz, Chloroform-*d*) δ 8.15 (d, *J* = 7.9 Hz, 1H), 7.52 – 7.41 (m, 1H), 7.35 – 7.11 (m, 6H), 4.33 – 4.18 (m, 2H), 3.00 (td, *J* = 14.2, 13.5, 4.6 Hz, 1H), 2.90 – 2.82 (m, 2H), 2.72 – 2.63 (m, 1H), 1.22 (t, *J* = 7.1 Hz, 3H). ^{13}C NMR (101 MHz, Chloroform-*d*) δ 195.40 (t, *J* = 2.9 Hz), 163.28 (t, *J* = 32.3 Hz), 143.05, 134.50, 131.61, 129.55, 129.16, 128.82, 128.70, 128.31, 127.36, 127.20, 114.96 (t, *J* = 259.6 Hz), 62.78, 60.62 (dd, *J* = 22.7, 21.0 Hz), 28.45 (dd, *J* = 5.7, 3.3 Hz), 25.16, 13.76. ^{19}F NMR (376 MHz, Chloroform-*d*) δ -107.37 (d, *J* = 272.8 Hz, 1F), -110.00 (d, *J* = 272.7 Hz, 1F). HRMS (ESI) (*m/z*): [M+H]$^+$ calcd. for $C_{20}H_{18}O_3ClF_2$: 379.0907, found: 379.0914.

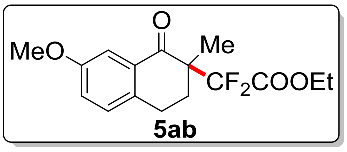

The product **5ab** is purified with silica gel chromatography (petroleum ether/ethyl acetate = 10/1 as the eluent) as a yellow oil (66% yield). ^{1}H NMR (400 MHz, Chloroform-*d*) δ 7.47 (d, *J* = 2.8 Hz, 1H), 7.15 (d, *J* = 8.4 Hz, 1H), 7.08 (dd, *J* = 8.4, 2.8 Hz, 1H), 4.37 (q, *J* = 7.1 Hz, 2H), 3.81 (s, 3H), 3.06 – 2.93 (m, 2H), 2.60 – 2.53 (m, 1H), 2.08 (dt, *J* = 13.6, 4.6 Hz, 1H), 1.50 (s, 3H), 1.37 (t, *J* = 7.1 Hz, 3H). ^{13}C NMR (101 MHz, Chloroform-*d*) δ 196.66 (t, *J* = 3.1 Hz), 163.77 (t, *J* = 32.3 Hz), 158.60, 135.47, 131.79, 130.05, 122.76, 116.39 (t, *J* = 257.1 Hz), 109.77, 62.89, 55.56, 51.86 (t, *J* = 22.0 Hz), 28.60 (dd, *J* = 5.1, 4.0 Hz), 24.02, 16.64 (t, *J* = 4.1 Hz), 13.99. ^{19}F NMR (376 MHz, Chloroform-*d*) δ -111.59 (d, *J* = 266.2 Hz, 1F), -112.41 (d, *J* = 266.2 Hz, 1F). HRMS (ESI) (*m/z*): [M+H]$^+$ calcd. for $C_{16}H_{19}O_4F_2$: 313.1246, found: 313.1244.

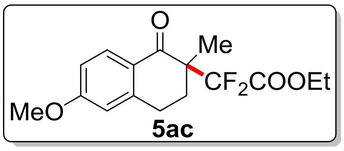

The product **5ac** is purified with silica gel chromatography (petroleum ether/ethyl acetate = 10/1 as the eluent) as a yellow oil (69% yield). ^{1}H NMR (400 MHz, Chloroform-*d*) δ 7.98 (d, *J* = 8.8 Hz, 1H), 6.83 (dd, *J* = 8.8, 2.4 Hz, 1H), 6.68 (d, *J* = 2.1 Hz, 1H), 4.37 (q, *J* = 7.1 Hz, 2H), 3.85 (s, 3H), 3.11 - 2.96 (m, 2H), 2.58 (ddd, *J* = 13.7, 11.1, 5.6 Hz, 1H), 2.11 – 2.04 (m, 1H), 1.50 (s, 3H), 1.37 (t, *J* = 7.1 Hz, 3H). ^{13}C NMR (101 MHz, Chloroform-*d*) δ 195.34 (t, *J* = 2.8 Hz), 164.20, 163.86 (t, *J* = 32.3Hz), 145.44 , 130.79 , 124.69 , 116.57 (t, *J* = 258.0Hz), 113.81, 112.47, 62.86, 55.62, 51.79 (t, *J* = 21.9 Hz), 28.56 (dd, *J* = 5.3, 3.4 Hz), 25.31, 16.99 (t, *J* = 4.2 Hz), 14.01. ^{19}F NMR (376 MHz, Chloroform-*d*) δ -111.62 (d, *J* = 264.7 Hz, 1F), -112.54 (d, *J* = 264.8 Hz, 1F). HRMS (ESI) (*m/z*): [M+H]$^+$ calcd. for $C_{16}H_{19}O_4F_2$: 313.1246, found: 313.1252.

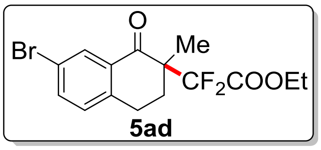

The product **5ad** is purified with silica gel chromatography (petroleum ether/ethyl acetate = 10/1 as the eluent) as a yellow oil (70% yield). ^{1}H NMR (400 MHz, Chloroform-*d*) δ 8.12 (d, *J* = 2.2 Hz, 1H), 7.60 (dd, *J* = 8.2, 2.2 Hz, 1H), 7.14 (d, *J* = 8.2 Hz, 1H), 4.36 (q, *J* = 7.1 Hz, 2H), 3.01 (dd, *J* = 8.0, 4.9 Hz, 2H), 2.57 (dt, *J* = 13.9, 8.1 Hz, 1H), 2.10 (dt, *J* = 13.8, 4.8 Hz, 1H), 1.48 (s, 3H), 1.36 (t, *J* = 7.2 Hz, 3H). ^{13}C NMR (101 MHz, Chloroform-*d*) δ 195.32 (t, *J* = 2.8 Hz), 163.52 (t, *J* = 32.2 Hz), 141.58, 136.83, 132.69, 130.94, 130.66, 121.07, 116.22 (t, *J* = 257.8 Hz), 63.05, 51.63 (t, *J* = 21.9 Hz), 28.47 (t, *J* = 4.2 Hz), 24.47, 16.72 (t, *J* = 4.2 Hz), 13.98. ^{19}F NMR (376 MHz, Chloroform-*d*) δ -111.57 (s, 2F). HRMS (ESI) (*m/z*): [M+H]$^+$ calcd. for $C_{15}H_{16}O_3BrF_2$: 361.0245, found: 361.0253.

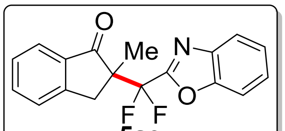

5ae

The product **5ae** is purified with silica gel chromatography (petroleum ether/ethyl acetate = 10/1 as the eluent) as a yellow oil (83% yield). ^{1}H NMR (400 MHz, Chloroform-*d*) δ 7.79 (d, *J* = 7.7 Hz, 1H), 7.74 (d, *J* = 7.4 Hz, 1H), 7.63 – 7.57 (m, 2H), 7.49 – 7.33 (m, 4H), 3.83 (d, *J* = 17.6 Hz, 1H), 3.07 (d, *J* = 17.6 Hz, 1H), 1.64 (s, 3H). ^{13}C NMR (101 MHz, Chloroform-*d*) δ 202.43 (t, *J* = 2.2 Hz), 156.64 (t, *J* = 33.9 Hz), 151.56, 150.51, 139.98, 135.58, 135.36, 128.02, 126.88, 126.59, 125.30, 124.94, 121.43, 117.14 (t, *J* = 249.5 Hz), 111.50, 55.00 (t, *J* = 22.6 Hz), 37.47 (t, *J* = 2.8 Hz), 19.33 (t, *J* = 4.5 Hz). ^{19}F NMR (376 MHz, Chloroform-*d*) δ -102.04 (d, *J* = 277.0 Hz, 1F), -105.22 (d, *J* = 277.1 Hz, 1F). HRMS (ESI) (*m/z*): $[M+H]^+$ calcd. for $C_{18}H_{14}F_2NO_2$: 314.0987, found: 314.1005.

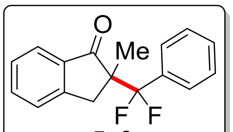

5af

The product **5af** is purified with silica gel chromatography (petroleum ether/ethyl acetate = 10/1 as the eluent) as a yellow oil (73% yield). ^{1}H NMR (400 MHz, Chloroform-*d*) δ 7.71 (d, *J* = 7.7 Hz, 1H), 7.59 – 7.52 (m, 1H), 7.46 – 7.30 (m, 7H), 3.67 (d, *J* = 17.7 Hz, 1H), 2.91 (d, *J* = 17.7 Hz, 1H), 1.45 (s, 3H). ^{13}C NMR (101 MHz, Chloroform-*d*) δ 203.81 (t, *J* = 2.9 Hz), 151.77, 135.91 (t, *J* = 2.0 Hz), 135.34, 134.40 (t, *J* = 26.9 Hz), 129.99, 127.87, 127.76, 126.82 (t, *J* = 6.7 Hz), 126.31, 124.56, 122.59 (dd, *J* = 251.6, 249.7 Hz). 56.06 (t, *J* = 26.2 Hz), 37.37 (t, *J* = 3.1 Hz), 19.78 (t, *J* = 4.5 Hz). ^{19}F NMR (376 MHz, Chloroform-*d*) δ -98.28 (d, *J* = 249.5 Hz, 1F), -101.63 (d, *J* = 249.5 Hz, 1F). HRMS (ESI) (*m/z*): $[M+Na]^+$ calcd. for $C_{17}H_{14}F_2ONa$: 295.0905, found: 295.0923.

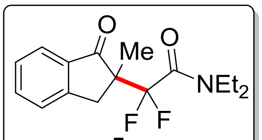

5ag

The product **5ag** is purified with silica gel chromatography (petroleum ether/ethyl acetate = 10/1 as the eluent) as a yellow oil (79% yield). ^{1}H NMR (400 MHz, Chloroform-*d*) δ 7.81 (d, *J* = 7.6 Hz, 1H), 7.60 – 7.52 (m, 1H), 7.45 – 7.33 (m, 2H), 3.65 – 3.47 (m, 2H), 3.42 (d, *J* = 16.9 Hz, 1H), 3.30 – 3.21 (m, 2H), 2.89 (d, *J* = 16.9 Hz, 1H), 1.40 (s, 3H), 1.22 (t, *J* = 7.0 Hz, 3H), 1.05 (t, *J* = 7.1 Hz, 3H). ^{13}C NMR (101 MHz, Chloroform-*d*) δ 203.26 (d, *J* = 6.2 Hz), 161.44 (t, *J* = 29.6 Hz), 150.53, 135.83 (dd, *J* = 4.9, 1.1 Hz), 134.55, 127.64, 126.57, 124.70, 120.77 (dd, *J* = 266.8, 261.4 Hz), 53.65 (dd, *J* = 21.5, 19.6 Hz), 41.55 (t, *J* = 6.4 Hz), 41.24, 38.97 (dd, *J* = 4.2, 2.2 Hz), 18.88 (dd, *J* = 8.2, 3.5 Hz), 14.30, 12.38. ^{19}F NMR (376 MHz, Chloroform-*d*) δ -100.84 (d, *J* = 292.3 Hz, 1F), -103.17 (d, *J* = 292.3 Hz, 1F). HRMS (ESI) (*m/z*): $[M+H]^+$ calcd. for $C_{16}H_{20}NO_2F_2$: 296.1457, found: 296.1467.

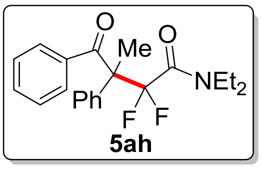

5ah

The product **5ah** is purified with silica gel chromatography (petroleum ether/ethyl acetate = 10/1 as the eluent) as a yellow oil (71% yield). ^{1}H NMR (400 MHz, Chloroform-*d*) δ 7.55 – 7.49 (m, 2H), 7.45 – 7.33 (m, 6H), 7.27 – 7.20 (m, 2H), 3.67 – 3.48 (m, 4H), 2.05 (s, 3H), 1.73 – 1.48 (m, 6H). ^{13}C NMR (101 MHz, Chloroform-*d*) δ 200.26 (dd, *J* = 5.1, 2.1 Hz), 160.89 (t, *J* = 29.0 Hz), 137.76 (d, *J* = 3.5 Hz), 136.13 (d, *J* = 3.1 Hz), 131.45, 129.40, 128.95, 128.49, 128.36, 127.99, 118.54 (dd, *J* = 272.1, 257.8 Hz), 60.38 (dd, *J* = 21.4, 17.2 Hz), 47.06 (dd, *J* = 8.9, 5.6 Hz), 44.78, 26.55, 25.80, 24.60, 21.41 (dd, *J* = 6.0, 3.1 Hz). ^{19}F NMR (376 MHz, Chloroform-*d*) δ -96.42 (d, *J* = 285.6 Hz, 1F), -104.50 (d, *J* = 285.7 Hz, 1F). HRMS (ESI) (*m/z*): $[M+H]^+$ calcd. for $C_{22}H_{24}O_2F_2N$: 372.1770, found: 372.1774.

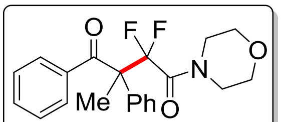

5ai

The product **5ai** is purified with silica gel chromatography (petroleum ether/ethyl acetate = 10/1 as the eluent) as a yellow oil (70% yield). ^{1}H NMR (400 MHz, Chloroform-*d*) δ 7.53 – 7.46 (m, 2H), 7.45 – 7.33 (m, 6H), 7.29 – 7.22 (m, 2H), 3.82 – 3.58 (m, 8H), 2.06 (s, 3H). ^{13}C NMR (101 MHz, Chloroform-*d*) δ 199.97

(dd, J = 5.1, 1.1 Hz), 161.37 (t, J = 29.4 Hz), 137.18 (d, J = 3.5 Hz), 135.82 (d, J = 4.1 Hz), 131.79, 129.42, 129.14, 128.57, 128.55, 128.09, 118.24 (dd, J = 271.1, 256.6 Hz), 66.87, 60.49 (dd, J = 21.4, 17.1 Hz), 46.85 (dd, J = 9.0, 5.1 Hz), 43.69, 21.17 (dd, J = 5.8, 3.2 Hz). ^{19}F NMR (376 MHz, Chloroform-*d*) δ -95.48 (d, J = 287.4 Hz, 1F), -103.97 (d, J = 287.4 Hz, 1F). HRMS (ESI) (*m/z*): $[M+H]^+$ calcd. for $C_{21}H_{22}O_3F_2N$: 374.1562, found: 374.1567.

Mechanistic Studies

1. Procedure of other fluoromethylation reagents or non-fluoromethylation reagents:

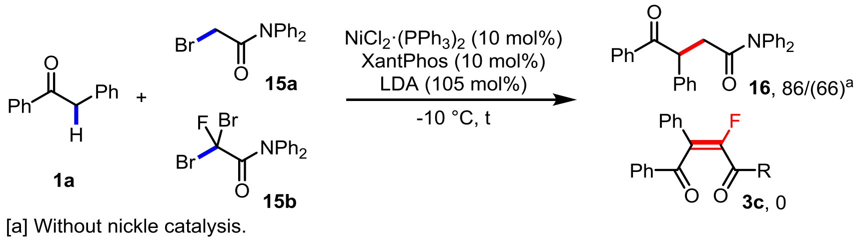

Figure S1. Fluorine effect

To a 50 mL of Schlenk tube were added ketone **1a** (1.0 equiv, 0.2 mmol), $NiCl_2$•$(PPh_3)_2$ (10 mol %, 0.02 mmol) and XantPhos (10 mol %, 0.02 mmol) under air. The mixture was evacuated and backfilled with N_2 (3 times). THF (2 mL) was added then followed by LDA (105 mol%, 0.21 mmol) subsequently. The Schlenk tube was then sealed with a Teflon lined cap and put into a cooled bath (-10 ℃). After stirring for 5 min, the reaction mixture was added cross-coupling reagents **15** (3.0 equiv, 0.6 mmol), then sealed with a Teflon lined cap and put into a cooled bath (-10 ℃) for 12 h. And the mixture was added ethyl acetate 5 mL, then mixture was removed under reduced pressure and purified by flash column chromatography on silica gel to give product as shown above.

2. Radical clock experiment:

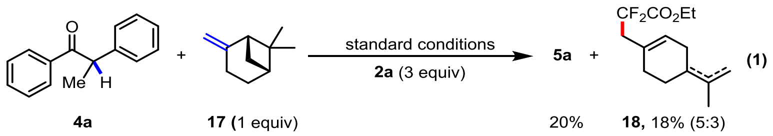

Figure S2. Radical trapping experiment with β-piene

To a 50 mL of Schlenk tube were added ketone **4a** (1.0equiv, 0.2 mmol), $NiCl_2$•dppe (10 mol %, 0.02 mmol) and XantPhos (10 mol %, 0.02 mmol) under air, followed by $ZnCl_2$ (20 mol%, 0.04 mmol). The mixture was evacuated and backfilled with N_2 (3 times). THF (2 mL) was added then followed by LDA (105 mol%, 0.21 mmol) subsequently. The Schlenk tube was then sealed with a Teflon lined cap and put into a cooled bath (-10 ℃). After stirring for 5 min, the reaction mixture was added bormdifluoroacetate (3.0 equiv, 0.6 mmol) and β-pinene (1.0 equiv, 0.2 mmol), then sealed with a Teflon lined cap and put into a cooled bath (-10 ℃) for 12 h. And the mixture was added ethyl acetate 5 mL, then mixture was removed under reduced pressure and purified by flash column chromatography on silica gel to give product **5a** in 20% yield and ring-open diene product **18** in 18% yield (5 : 3 isomer ratio).

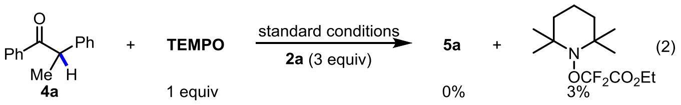

Figure S3. Radical trapping experiment with TEMPO

To a 50 mL of Schlenk tube were added ketone **4a** (1.0 equiv, 0.2 mmol), $NiCl_2$•dppe (10 mol %, 0.02 mmol), XantPhos (10 mol %, 0.02 mmol) and $ZnCl_2$ (20 mol%, 0.04 mmol) under air, followed by TEMPO (1.0 equiv, 0.2 mmol). The mixture was evacuated and backfilled with N_2 (3 times). THF (2 mL) was added then followed by LDA (105 mol%, 0.21 mmol) subsequently. The Schlenk tube was then sealed with a Teflon lined cap and put into a cooled bath (-10 ℃). After stirring for 5 min, the reaction mixture was added bormdifluoroacetate (3.0 equiv, 0.6 mmol), then sealed with a Teflon lined cap and put into a cooled bath (-10 ℃) for 12 h. And the mixture was added ethyl acetate 5 mL, then mixture was determined by ^{19}F NMR analysis.

3. Procedure of difluoroalkylation with Ni^{I} used as the catalyst[1-3]:

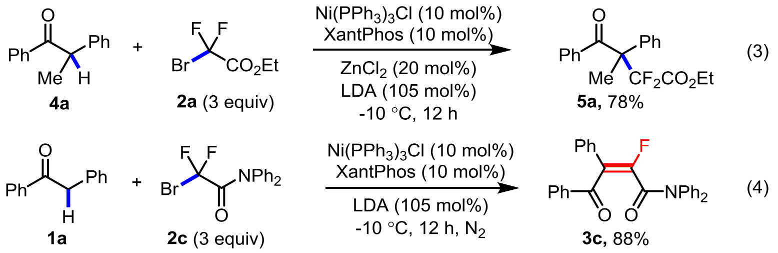

Figure S4. Control experiments using Nickle(I) complex

To a 50 mL of Schlenk tube were added ketone **4a** (1.0 equiv, 0.2 mmol), $Ni(PPh_3)_3Cl$ (10 mol%, 0.02 mmol), $ZnCl_2$ (20 mol%, 0.04 mmol) and XantPhos (10 mol %, 0.02 mmol) in glove box. THF (2 mL) was added then followed by LDA (105 mol%, 0.21 mmol) subsequently. The Schlenk tube was then sealed with a Teflon lined cap and put into a cooled bath (-10 ℃). After stirring for 5 min, the reaction mixture was added bormdifluoroacetate **2a** (3.0 equiv, 0.6 mmol), then sealed with a Teflon lined cap and put into a cooled bath (-10 ℃) for 12 h. And the mixture was added ethyl acetate 5 mL, then mixture was removed under reduced pressure and purified by flash column chromatography on silica gel to give product **5a** in 78% yield.

To a 50 mL of Schlenk tube were added ketone **1a** (1.0 equiv, 0.2 mmol), $Ni(PPh_3)_3Cl$ (10 mol%, 0.02 mmol) and XantPhos (10 mol %, 0.02 mmol) in glove box. THF (2 mL) was added then followed by LDA (105 mol%, 0.21 mmol) subsequently. The Schlenk tube was then sealed with a Teflon lined cap and put into a cooled bath (-10 ℃). After stirring for 5 min, the reaction mixture was added 2-bromo-2,2-difluoro-N,N-diphenylacetamide **2c** (3.0 equiv, 0.6 mmol), then sealed with a Teflon lined cap and put into a cooled bath (-10 ℃) for 12 h. And the mixture was added ethyl acetate 5 mL, then mixture was removed under reduced pressure and purified by flash column chromatography on silica gel to give product **3c** in 88% yield.

4. The operation of an initial transmetallation:

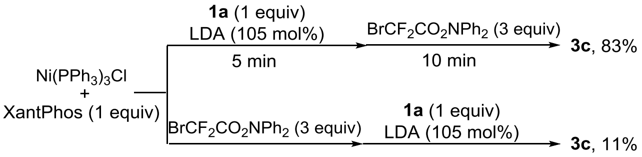

Figure S5. The control experiments of transmetallation step and activation of fluoroalkyl bromide

To a 50 mL of Schlenk tube was added $Ni(PPh_3)_3Cl$ (1.0 equiv, 0.2 mmol) and XantPhos (1.0 equiv, 0.2 mmol) under air, then was added by **1a** (1.0 equiv, 0.2 mmol). THF (2 mL) was added then followed

by LDA (105 mol%, 0.21 mmol) subsequently. The Schlenk tube was then sealed with a Teflon lined cap and put into a cooled bath (- 10 ℃). After stirring for 5 minutes, 2-bromo-2,2-difluoro-N,N-diphenylacetamide **2c** (3.0 equiv, 0.6 mmol) was added to the reaction mixture, and the Schlenk tube was then resealed with a Teflon lined cap and put back into the cooled bath (- 10 ℃). After stirring for another 10 minutes, the reaction mixture was diluted with ethyl acetate (5 mL). The solvent was removed under reduced pressure, and the residue was purified by flash column chromatography on silica gel to give the desired product of **3c**. To another 50 mL of Schlenk tube was added $Ni(PPh_3)_3Cl$ (1.0 equiv, 0.2 mmol) and XantPhos (1.0 equiv, 0.2 mmol) under air, then was added by 2-bromo-2,2-difluoro-N,N-diphenylacetamide **2c** (3.0 equiv, 0.6 mmol) and THF (2 mL). The Schlenk tube was then sealed with a Teflon lined cap and put into a cooled bath (- 10 ℃). After stirring for 5 minutes, **1a** (1.0 equiv, 0.2 mmol) and LDA (105 mol%, 0.21 mmol) were added to the reaction mixture, and the Schlenk tube was then resealed with a Teflon lined cap and put back into the cooled bath (- 10 ℃). After stirring for another 10 minutes, the reaction mixture was diluted with ethyl acetate (5 mL). The solvent was removed under reduced pressure, and the residue was purified by flash column chromatography on silica gel to give the desired product of **3c.**

5. Generation of Ni(I) species and possible catalytic cycle:

First of all, Ni (II) was treated with lithium diisopropylamide (LDA)[4] to form an active species of Ni(0).[5] The high resolution mass spectra revealed the formation of $Me_2C{=}NiPr$, suggesting that the reduction of Ni(II) occurred through α-hydride transfer[4] from LDA to Ni(II). Alternatively, the transmetallation with LDA could form a Ni(II)-amido species first and then followed β-hydride elimination to generate the observed imine byproduct. Consequently, comproportionation between Ni(II) and Ni(0), afforded the corresponding Ni(I) complex A.[6] Then transmetallation between Ni(I) catalyst **A** and in-situ generated enol anion **B** gave the Ni(I) complex **C** and **D**, which furnished the Ni(II) species **E** and the difluoroalkyl radical via a single-electron oxidation by fluoroalkyl bromide **2**. The following radical oxidation of Ni(II) species **E** afforded Ni(III) intermediated **F**, followed by reductive elimination resulted in alkyl difluoride **5** when tertiary aryl ketone was used as the substrate (R = aryl or alkyl). Instead, starting from a secondary ketone (R = H), a defluorination took place through a E2 elimination process and furnished a tetrafluoroalkylated monofluoroalkene **3** as the final product.

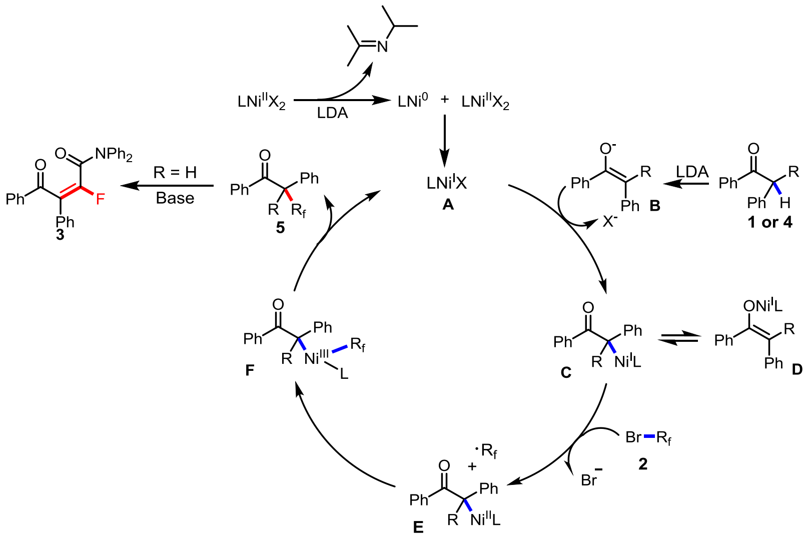

Figure S6. The possible reaction mechanism.

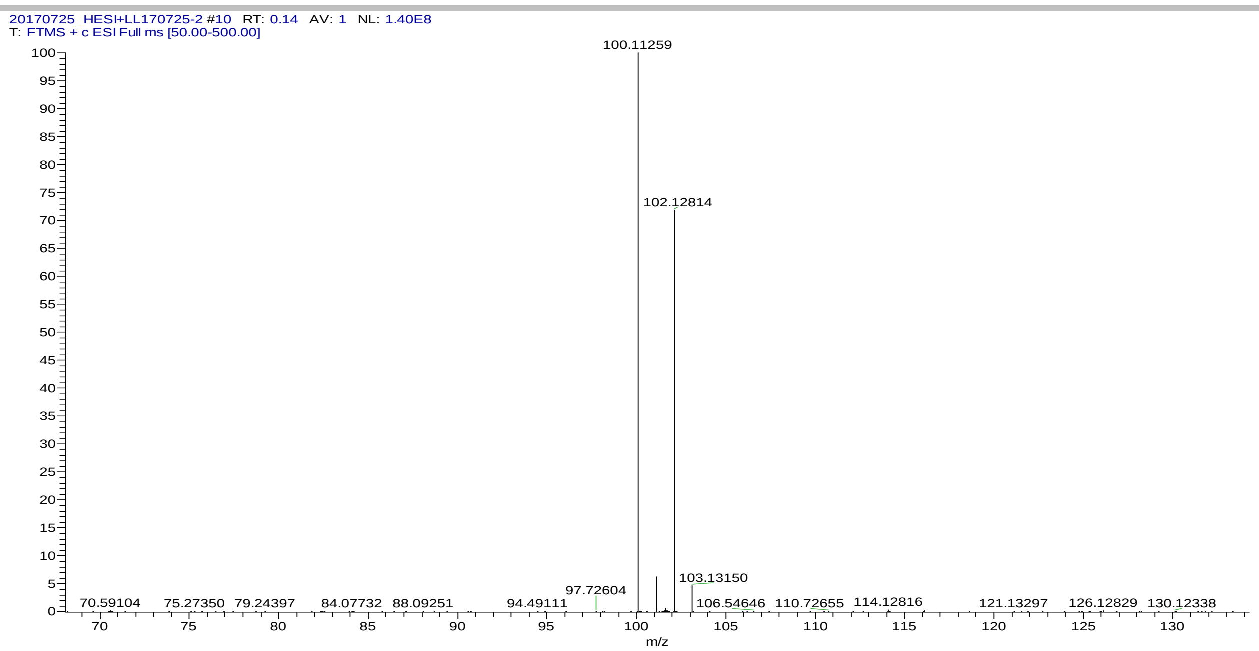

Figure S7. The HRMS spectrum of $Me_2C{=}N^iPr$.

References:

(1) C. Liu, S. Tang, D. Liu, J. Yuan, L. Zheng, L. Meng and A. Lei, *Angew. Chem. Int. Ed.* 2012, **51**, 3638.

(2) A. Wilsily, F. Tramutola, N. A. Owston and G. C. Fu, *J. Am. Chem. Soc.* 2012, **134**, 5794.

(3) S. L. Zultanski and G. C. Fu, *J. Am. Chem. Soc.* 2013, **135**, 624.

(4) C. Kowalski, X. Creary, A. J. Rollin and M. C. Burke, *J. Org.Chem.* 1978, **43**, 2601.

(5) L. Ackermann, *et al. Eur. J. Inorg. Chem.* 2014, **2014**, 818.

(6) S. J. Hwang, D. C., Powers, A. G. Maherab and D. G. Nocera, *Chem. Sci.* 2015, **6**, 917.

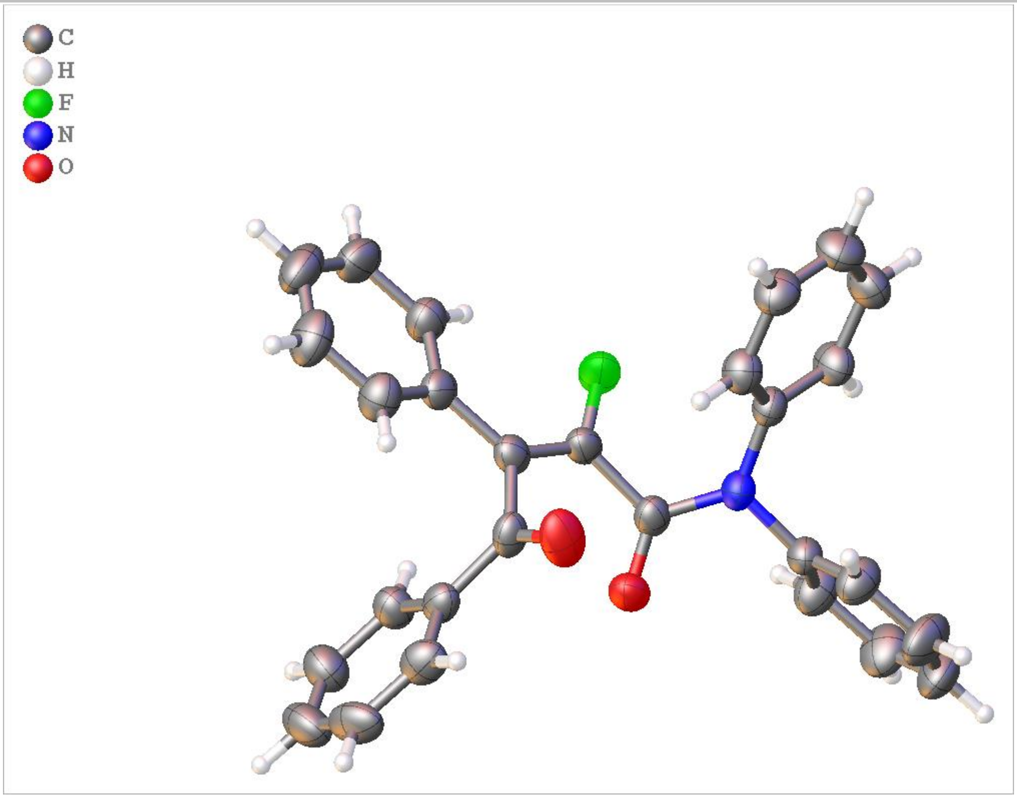

Figure S6. X-Ray crystal data of **3c**

Table S16. Crystal data and structure refinement for 3c.

Identification code	3c
Empirical formula	$C_{28}H_{20}FNO_2$
Formula weight	421.45
Temperature/K	291(2)
Crystal system	orthorhombic
Space group	P212121
a/Å	5.9409(2)
b/Å	10.8429(2)
c/Å	32.9352(5)
α/°	90
β/°	90
γ/°	90
Volume/Å^3	2121.57(9)
Z	4
ρcalcg/cm^3	1.319
μ/mm^{-1}	0.718
F(000)	880.0
Crystal size/mm^3	0.230 × 0.220 × 0.220
Radiation	CuK$^{\alpha}$ (λ = 1.54184)
2$^{\Theta}$ range for data collection/ °	8.586 to 142.534
Index ranges	-6 ≤ h ≤ 5, -13 ≤ k ≤ 11, -40 ≤ l ≤ 40
Reflections collected	10995
Independent reflections	3964 [R_{int} = 0.0279, R_{sigma} = 0.0247]
Data/restraints/parameters	3964/0/289
Goodness-of-fit on F^2	1.020
Final R indexes [I>=2σ (I)]	R_1 = 0.0438, wR_2 = 0.1031

Final R indexes [all data]	R_1 = 0.0457, wR_2 = 0.1041
Largest diff. peak/hole / e Å^{-3}	0.14/-0.20
Flack parameter	0.06(9)

Table S17. Fractional Atomic Coordinates (×10^4) and Equivalent Isotropic Displacement Parameters (Å^2×10^3) for 3c. U_{eq} is defined as 1/3 of of the trace of the orthogonalised U_{IJ} tensor.

Atom	x	y	z	U(eq)
F1	4936(4)	3869.8(17)	5819.1(6)	54.9(5)
O2	6256(4)	2638(2)	6736.5(6)	51.2(6)
N1	5595(4)	1516(2)	6167.8(7)	38.0(6)
O1	11070(5)	3305(2)	6414.8(8)	65.4(7)
C7	8063(6)	4540(3)	6191.9(9)	39.2(7)
C15	6475(6)	3718(3)	6120.1(8)	39.1(7)
C17	5903(5)	1322(3)	5739.9(9)	38.2(6)
C16	6107(6)	2576(3)	6368.3(9)	39.9(7)
C23	5064(6)	433(3)	6402.8(8)	39.9(7)
C1	8456(6)	5721(3)	5972.3(9)	42.8(7)
C8	9846(5)	4150(3)	6501.6(10)	42.1(7)
C9	10082(6)	4864(3)	6884.9(9)	42.6(7)
C22	7880(6)	1634(3)	5550(1)	47.0(8)
C6	10505(6)	6328(3)	6010.1(11)	54.4(9)
C14	8415(6)	5653(3)	7015.7(10)	48.6(8)
C19	4531(7)	461(3)	5119.9(10)	56.0(9)
C18	4233(6)	724(3)	5525(1)	47.7(8)
C24	6567(7)	-520(3)	6403.9(11)	54.1(9)
C2	6824(7)	6265(3)	5725.2(10)	51.0(8)
C28	3069(6)	342(3)	6611.3(11)	53.3(9)
C10	12053(6)	4736(3)	7109.9(10)	53.3(9)
C20	6493(7)	791(4)	4926.2(10)	59.7(10)
C12	10701(8)	6268(4)	7576.3(11)	66.9(12)
C21	8160(7)	1380(3)	5138.9(10)	55.6(9)
C11	12336(7)	5454(4)	7456.1(11)	65.3(11)
C13	8737(8)	6362(4)	7361.3(10)	61.5(10)
C27	2589(8)	-740(4)	6817.5(12)	65.6(11)
C5	10924(8)	7422(4)	5810.1(13)	65.9(10)
C26	4068(9)	-1705(4)	6813.4(12)	69.5(12)
C3	7274(8)	7352(3)	5521.3(11)	62.4(10)
C4	9296(8)	7932(3)	5561.4(12)	66.7(11)
C25	6071(9)	-1593(4)	6607.2(12)	72.5(12)

Table S18. Anisotropic Displacement Parameters (Å^2 ×10^3) for 3c. The Anisotropic displacement factor exponent takes the form: $-2\pi^2[h^2a^{*2}U_{11}+2hka^*b^*U_{12}+\ldots]$.

Atom	U_{11}	U_{22}	U_{33}	U_{23}	U_{13}	U_{12}
F1	65.2(13)	44(1)	55.5(11)	6.2(8)	-28.7(10)	-2.5(10)
O2	70.0(16)	42.6(12)	40.9(11)	0.0(9)	-15.3(11)	2.7(12)
N1	42.8(15)	30.5(12)	40.6(12)	-0.4(10)	-0.2(11)	-4.1(11)
O1	58.7(16)	50.7(14)	86.8(18)	-14.5(13)	-19.3(14)	20.2(13)
C7	43.9(17)	33.7(15)	39.8(14)	-5.3(12)	-3.8(13)	5.7(14)
C15	46.3(18)	33.8(15)	37.2(14)	-1.2(12)	-10.6(13)	5.0(14)
C17	42.9(17)	30.6(14)	41.2(14)	0.6(12)	1.2(13)	-3.1(13)
C16	43.0(18)	31.9(14)	44.9(15)	-0.7(12)	-8.4(13)	4.0(14)
C23	49.6(18)	33.4(15)	36.7(14)	-0.8(11)	0.1(13)	-3.8(15)
C1	55(2)	32.4(15)	40.6(15)	-3.3(12)	0.6(14)	6.9(15)
C8	40.5(17)	28.9(14)	56.7(18)	-0.5(13)	-8.4(15)	4.2(13)
C9	44.3(18)	36.4(16)	47.0(16)	4.9(12)	-11.3(14)	-7.0(14)
C22	44.4(19)	43.4(18)	53.1(18)	-1.6(14)	0.8(15)	-3.5(15)
C6	51(2)	47.5(19)	65(2)	2.2(16)	-1.6(17)	-3.0(17)
C14	54(2)	44.2(18)	47.8(17)	-2.9(14)	-10.2(15)	0.1(16)
C19	60(2)	57(2)	51.0(18)	-9.4(16)	-8.2(17)	-8.6(18)
C18	46.7(19)	47.7(18)	48.7(17)	-0.7(14)	1.7(14)	-12.3(16)
C24	60(2)	43.8(18)	58.6(19)	4.7(15)	13.8(17)	6.6(17)
C2	56(2)	40.6(18)	55.8(18)	1.4(15)	-6.4(17)	2.5(16)
C28	51(2)	44.8(19)	64(2)	3.4(16)	10.1(17)	4.9(17)
C10	44(2)	56(2)	60(2)	9.0(16)	-12.0(16)	-5.0(17)
C20	73(3)	64(2)	41.7(17)	-6.1(16)	8.2(18)	0(2)
C12	81(3)	76(3)	43.9(18)	-7.4(18)	-6.5(19)	-26(3)
C21	60(2)	54(2)	53.1(18)	2.1(16)	15.0(17)	-2.0(18)
C11	59(2)	86(3)	51(2)	11.7(19)	-21.7(18)	-26(2)
C13	74(3)	59(2)	51.7(19)	-9.2(17)	-2.7(19)	-7(2)
C27	67(3)	59(2)	71(2)	6.5(19)	19(2)	-15(2)
C5	65(3)	48(2)	85(3)	6(2)	11(2)	-10(2)
C26	104(3)	42(2)	63(2)	10.0(17)	11(2)	-12(2)
C3	85(3)	44(2)	58(2)	8.4(17)	-6(2)	11(2)
C4	92(3)	41.0(19)	67(2)	10.5(17)	16(2)	2(2)
C25	103(3)	40.5(19)	74(2)	10.0(18)	16(3)	20(2)

Table S19. Bond Lengths for 3c.

Atom	Atom	Length/Å	Atom	Atom	Length/Å
F1	C15	1.359(3)	C9	C10	1.392(5)
O2	C16	1.218(3)	C22	C21	1.392(5)
N1	C16	1.360(4)	C6	C5	1.380(5)
N1	C17	1.437(4)	C14	C13	1.387(5)
N1	C23	1.441(4)	C19	C18	1.376(5)
O1	C8	1.204(4)	C19	C20	1.376(5)
C7	C15	1.320(4)	C24	C25	1.374(5)
C7	C1	1.489(4)	C2	C3	1.382(5)
C7	C8	1.530(4)	C28	C27	1.385(5)
C15	C16	1.500(4)	C10	C11	1.391(5)
C17	C22	1.373(5)	C20	C21	1.371(5)
C17	C18	1.380(4)	C12	C13	1.369(6)
C23	C24	1.366(5)	C12	C11	1.371(6)
C23	C28	1.373(5)	C27	C26	1.366(6)
C1	C6	1.390(5)	C5	C4	1.383(6)
C1	C2	1.397(5)	C26	C25	1.375(6)
C8	C9	1.487(4)	C3	C4	1.362(6)

Table S20. Bond Angles for 3c.

Atom	Atom	Atom	Angle/°	Atom	Atom	Atom	Angle/°
C16	N1	C17	124.8(2)	C14	C9	C10	120.0(3)
C16	N1	C23	118.5(2)	C14	C9	C8	121.4(3)
C17	N1	C23	115.8(2)	C10	C9	C8	118.6(3)
C15	C7	C1	127.3(3)	C17	C22	C21	119.8(3)
C15	C7	C8	115.3(3)	C5	C6	C1	121.5(4)
C1	C7	C8	117.0(3)	C9	C14	C13	120.1(3)
C7	C15	F1	122.0(3)	C18	C19	C20	120.3(3)
C7	C15	C16	124.4(3)	C19	C18	C17	120.1(3)
F1	C15	C16	113.6(3)	C23	C24	C25	120.1(4)
C22	C17	C18	119.8(3)	C3	C2	C1	120.6(4)
C22	C17	N1	121.3(3)	C23	C28	C27	118.9(3)
C18	C17	N1	118.7(3)	C11	C10	C9	118.9(4)
O2	C16	N1	123.1(3)	C21	C20	C19	119.7(3)
O2	C16	C15	119.1(3)	C13	C12	C11	120.2(3)
N1	C16	C15	117.8(2)	C20	C21	C22	120.2(3)
C24	C23	C28	120.6(3)	C12	C11	C10	120.7(4)
C24	C23	N1	118.3(3)	C12	C13	C14	120.0(4)
C28	C23	N1	121.1(3)	C26	C27	C28	120.7(4)
C6	C1	C2	117.4(3)	C6	C5	C4	120.0(4)
C6	C1	C7	120.1(3)	C27	C26	C25	119.6(3)
C2	C1	C7	122.5(3)	C4	C3	C2	121.1(4)
O1	C8	C9	122.7(3)	C3	C4	C5	119.3(4)
O1	C8	C7	118.0(3)	C24	C25	C26	120.0(4)
C9	C8	C7	119.1(3)				

Table S21. Torsion Angles for 3c.

A	B	C	D	Angle/°	A	B	C	D	Angle/°
C1	C7	C15	F1	0.1(5)	C18	C17	C22	C21	2.0(5)
C8	C7	C15	F1	-172.0(3)	N1	C17	C22	C21	177.0(3)
C1	C7	C15	C16	-176.7(3)	C2	C1	C6	C5	-0.4(5)
C8	C7	C15	C16	11.2(4)	C7	C1	C6	C5	179.9(3)
C16	N1	C17	C22	47.2(4)	C10	C9	C14	C13	2.9(5)
C23	N1	C17	C22	-121.9(3)	C8	C9	C14	C13	-176.1(3)
C16	N1	C17	C18	-137.7(3)	C20	C19	C18	C17	0.0(6)
C23	N1	C17	C18	53.2(4)	C22	C17	C18	C19	-1.1(5)
C17	N1	C16	O2	-164.9(3)	N1	C17	C18	C19	-176.3(3)
C23	N1	C16	O2	4.0(5)	C28	C23	C24	C25	1.4(5)
C17	N1	C16	C15	15.9(5)	N1	C23	C24	C25	-176.6(3)
C23	N1	C16	C15	-175.2(3)	C6	C1	C2	C3	1.3(5)
C7	C15	C16	O2	41.2(5)	C7	C1	C2	C3	-178.9(3)
F1	C15	C16	O2	-135.8(3)	C24	C23	C28	C27	-1.0(5)
C7	C15	C16	N1	-139.5(3)	N1	C23	C28	C27	177.0(3)
F1	C15	C16	N1	43.5(4)	C14	C9	C10	C11	-2.7(5)
C16	N1	C23	C24	-110.1(3)	C8	C9	C10	C11	176.4(3)
C17	N1	C23	C24	59.8(4)	C18	C19	C20	C21	0.3(6)
C16	N1	C23	C28	71.8(4)	C19	C20	C21	C22	0.6(6)
C17	N1	C23	C28	-118.3(3)	C17	C22	C21	C20	-1.7(5)
C15	C7	C1	C6	-163.2(3)	C13	C12	C11	C10	1.7(6)
C8	C7	C1	C6	8.8(4)	C9	C10	C11	C12	0.4(6)
C15	C7	C1	C2	17.1(5)	C11	C12	C13	C14	-1.4(6)
C8	C7	C1	C2	-170.9(3)	C9	C14	C13	C12	-0.9(6)
C15	C7	C8	O1	65.9(4)	C23	C28	C27	C26	-0.2(6)
C1	C7	C8	O1	-107.1(3)	C1	C6	C5	C4	-0.8(6)
C15	C7	C8	C9	-117.4(3)	C28	C27	C26	C25	1.0(6)
C1	C7	C8	C9	69.6(4)	C1	C2	C3	C4	-1.2(6)
O1	C8	C9	C14	-166.8(3)	C2	C3	C4	C5	0.1(6)
C7	C8	C9	C14	16.6(5)	C6	C5	C4	C3	0.9(6)
O1	C8	C9	C10	14.1(5)	C23	C24	C25	C26	-0.7(6)
C7	C8	C9	C10	-162.4(3)	C27	C26	C25	C24	-0.5(7)

Table S22. Hydrogen Atom Coordinates (Å×10^4) and Isotropic Displacement Parameters (Å^2 ×10^3) for 3c.

Atom	x	y	z	U(eq)
H22	9028	2014	5696	56
H6	11620	5989	6174	65
H14	7071	5709	6872	58
H19	3401	58	4976	67
H18	2903	499	5654	57
H24	7929	-443	6267	65
H2	5422	5893	5698	61
H28	2056	996	6614	64
H10	13160	4181	7030	64
H20	6688	615	4652	72
H12	10927	6758	7804	80
H21	9481	1611	5008	67

H11	13650	5380	7608	78
H13	7617	6903	7447	74
H27	1245	-811	6960	79
H5	12302	7817	5843	79
H26	3723	-2432	6949	83
H3	6177	7693	5354	75
H4	9578	8663	5423	80
H25	7090	-2245	6605	87

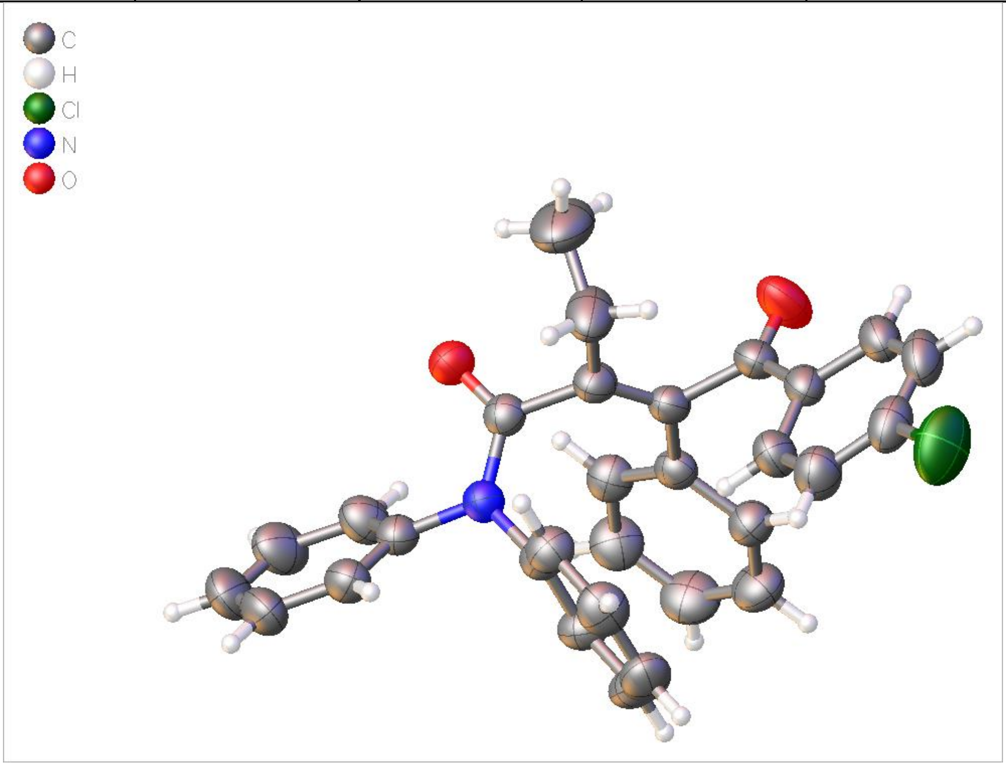

Figure S8. X-Ray crystal data of **6**

Table S23. Crystal data and structure refinement for 6.

Identification code	6
Empirical formula	$C_{30}H_{24}ClNO_2$
Formula weight	465.95
Temperature/K	291(2)
Crystal system	monoclinic
Space group	I2/a
a/Å	18.3331(3)
b/Å	14.0402(2)
c/Å	20.1069(3)
α /°	90
β /°	107.894(2)
γ /°	90
Volume/Å^3	4925.17(14)
Z	8
ρ calcg/cm^3	1.257
μ /mm^{-1}	1.581
F(000)	1952.0
Crystal size/mm^3	0.280 × 0.250 × 0.220
Radiation	CuK$^{\alpha}$ (λ = 1.54184)

2Θ range for data collection/ °	7.81 to 142.704
Index ranges	$-22 \le h \le 20$, $-17 \le k \le 16$, $-24 \le l \le 23$
Reflections collected	9721
Independent reflections	4666 [R_{int} = 0.0174, R_{sigma} = 0.0190]
Data/restraints/parameters	4666/0/308
Goodness-of-fit on F^2	1.055
Final R indexes [I>=2σ (I)]	R_1 = 0.0542, wR_2 = 0.1569
Final R indexes [all data]	R_1 = 0.0607, wR_2 = 0.1635
Largest diff. peak/hole / e Å^{-3}	0.81/-0.36

Table S24. Fractional Atomic Coordinates (×10^4) and Equivalent Isotropic Displacement Parameters (Å^2 ×10^3) for 6. U_{eq} is defined as 1/3 of of the trace of the orthogonalised U_{IJ} tensor.

Atom	x	y	z	U(eq)
Cl1	8538.5(4)	1520.5(7)	4206.6(5)	106.7(3)
O2	3326.5(8)	3045.9(10)	2203.9(7)	55.7(4)
N1	4005.4(8)	3782.5(11)	3207.3(8)	45.0(4)
O1	4908(1)	51.5(12)	3086.2(11)	81.6(5)
C25	4715.3(10)	3896.0(13)	3764.3(9)	45.3(4)
C16	3889.3(10)	3069.7(13)	2724.4(9)	44.7(4)
C9	4237.8(11)	1554.7(12)	3860.6(9)	44.6(4)
C8	4616.1(10)	1631.7(13)	3306.9(9)	45.2(4)
C19	3428.3(11)	4507.4(13)	3134.8(9)	47.5(4)
C15	4493.8(11)	2315.0(14)	2819.4(9)	49.0(4)
C4	6004.5(12)	991.2(14)	3573.8(9)	50.2(4)
C10	3474.4(11)	1801.7(14)	3744.2(11)	52.1(5)
C7	5165.9(12)	824.7(14)	3307.9(10)	53.3(5)
C30	5363.6(12)	4220.3(15)	3625.1(11)	55.1(5)
C20	3639.1(13)	5452.8(15)	3182.7(11)	56.7(5)
C26	4735.5(13)	3713.8(15)	4445.6(10)	54.9(5)
C14	4659.1(12)	1224.8(15)	4523.6(10)	54.8(5)
C3	6305.1(12)	1861.5(16)	3858.9(12)	59.0(5)
C24	2665.8(12)	4265.5(17)	3005.8(12)	61.3(5)
C5	6502.9(13)	273.9(16)	3519.0(11)	59.9(5)
C29	6033.6(12)	4357.8(17)	4174.3(13)	65.6(6)
C27	5407.3(15)	3848.0(18)	4982.1(11)	67.1(6)
C11	3155.4(13)	1770.8(18)	4283.7(13)	66.0(6)
C6	7282.3(14)	435.3(19)	3719.2(13)	71.3(7)
C2	7087.3(14)	2023.2(18)	4061.8(13)	68.8(6)
C13	4335.7(15)	1185.5(18)	5059.9(12)	67.5(6)
C28	6053.8(14)	4169.1(17)	4849.1(12)	68.2(6)
C1	7560.9(13)	1309(2)	3980.5(13)	68.2(6)
C21	3087.9(17)	6157.3(17)	3092.4(13)	73.0(7)
C12	3587.5(16)	1466(2)	4941.1(14)	74.2(7)
C22	2328.6(16)	5916(2)	2940.4(15)	80.9(8)
C17	4884.3(17)	2363(2)	2249.2(13)	80.9(8)
C23	2118.2(14)	4973(2)	2900.2(15)	78.4(7)
C18	4445(2)	1979(3)	1573.3(16)	121.8(15)

Table S25. Anisotropic Displacement Parameters ($Å^2 \times 10^3$) for 6. The Anisotropic displacement factor exponent takes the form: $-2\pi^2[h^2a^{*2}U_{11}+2hka^*b^*U_{12}+\ldots]$.

Atom	U_{11}	U_{22}	U_{33}	U_{23}	U_{13}	U_{12}
Cl1	54.0(4)	139.8(8)	120.7(7)	31.0(5)	18.4(4)	16.3(4)
O2	50.6(7)	59.2(8)	50.9(7)	-6.4(6)	6.1(6)	4.4(6)
N1	44.1(8)	45.5(8)	44.6(8)	-0.8(6)	12.7(6)	2.2(6)
O1	75.9(11)	57.5(9)	102.6(13)	-29.1(9)	14.3(9)	5.8(8)
C25	48(1)	42.4(9)	44.7(9)	-2.2(7)	13.0(7)	1.8(7)
C16	46.1(9)	47.0(9)	42.5(9)	3.0(7)	15.7(7)	0.6(7)
C9	50.0(9)	38.5(8)	45.4(9)	-3.9(7)	14.9(7)	-2.6(7)
C8	46.1(9)	45.5(9)	42.6(8)	-6.3(7)	11.5(7)	3.0(7)
C19	50.9(10)	50.4(10)	41.4(9)	-1.5(7)	14.4(7)	5.6(8)
C15	49(1)	56.3(11)	43.2(9)	-1.7(8)	16.2(8)	7.3(8)
C4	59.6(11)	50.5(10)	42.2(9)	2.0(8)	18.4(8)	14.0(8)
C10	49.2(10)	50.7(10)	56.7(10)	1.3(8)	16.8(8)	-6.2(8)
C7	63.2(12)	49.5(10)	46.7(9)	-7.4(8)	16.1(9)	9.1(9)
C30	53.8(11)	58.9(11)	54.7(11)	1.8(9)	19.6(9)	-2.6(9)
C20	64.1(12)	51.2(11)	52.5(10)	-2.9(8)	14.5(9)	2.5(9)
C26	63.2(12)	56.3(11)	47(1)	-4.1(8)	19.5(9)	-6.2(9)
C14	57.9(11)	56.4(11)	50(1)	2.0(8)	16.6(9)	5.0(9)
C3	58.1(12)	54.6(11)	62.6(12)	-3.5(9)	15.9(9)	11.8(9)
C24	53.8(11)	62.8(12)	70.5(13)	-9.2(10)	23.5(10)	2.4(9)
C5	74.2(14)	53.2(11)	54.4(11)	4.6(9)	22.8(10)	19.3(10)
C29	49.3(11)	67.6(13)	78.4(15)	-4.6(11)	17.3(10)	-6.4(10)
C27	81.2(15)	70.1(14)	43.9(10)	-4.0(9)	10.2(10)	-5.8(12)
C11	56.8(12)	73.3(14)	75.5(14)	3.7(11)	31.5(11)	-4.4(10)
C6	70.7(15)	78.3(16)	69.5(14)	17.2(12)	28.5(12)	35.0(12)
C2	61.5(13)	66.5(14)	73.1(14)	2.2(11)	13.0(11)	6.0(11)
C13	81.4(16)	73.5(14)	50.4(11)	7.6(10)	24.4(11)	1.4(12)
C28	62.3(13)	66.6(13)	62.2(13)	-10.1(11)	-0.9(10)	-2.0(11)
C1	55.6(12)	86.8(17)	61.2(12)	19.1(12)	16.5(10)	17.8(11)
C21	96.4(19)	51.7(12)	67.2(14)	-4.6(10)	20.0(13)	15.7(12)
C12	80.7(16)	85.4(17)	70.5(15)	3.7(12)	43.7(13)	-6.7(13)
C22	79.7(17)	79.3(17)	81.0(16)	-10.1(13)	20.7(13)	32.4(14)
C17	91.2(18)	100(2)	63.2(13)	17.3(13)	40.4(13)	39.8(15)
C23	56.5(13)	92.8(19)	86.8(17)	-19.9(14)	23.1(12)	14.8(12)
C18	146(3)	162(4)	60.8(16)	-0.5(19)	36.4(19)	53(3)

Table S26. Bond Lengths for 6.

Atom	Atom	Length/Å	Atom	Atom	Length/Å
Cl1	C1	1.734(3)	C4	C7	1.483(3)
O2	C16	1.224(2)	C10	C11	1.382(3)
N1	C16	1.365(2)	C30	C29	1.390(3)
N1	C25	1.442(2)	C20	C21	1.386(3)
N1	C19	1.443(2)	C26	C27	1.378(3)
O1	C7	1.213(3)	C14	C13	1.382(3)
C25	C30	1.379(3)	C3	C2	1.384(3)
C25	C26	1.383(3)	C24	C23	1.381(3)
C16	C15	1.502(3)	C5	C6	1.379(4)
C9	C10	1.390(3)	C29	C28	1.372(4)
C9	C14	1.398(3)	C27	C28	1.369(4)
C9	C8	1.485(3)	C11	C12	1.383(4)
C8	C15	1.340(3)	C6	C1	1.371(4)
C8	C7	1.516(3)	C2	C1	1.368(3)
C19	C20	1.377(3)	C13	C12	1.376(4)
C19	C24	1.383(3)	C21	C22	1.373(4)
C15	C17	1.528(3)	C22	C23	1.374(4)
C4	C5	1.387(3)	C17	C18	1.454(5)
C4	C3	1.390(3)			

Table S27. Bond Angles for 6.

Atom	Atom	Atom	Angle/˚	Atom	Atom	Atom	Angle/˚
C16	N1	C25	122.68(15)	O1	C7	C4	121.15(18)
C16	N1	C19	119.76(15)	O1	C7	C8	118.98(19)
C25	N1	C19	117.35(14)	C4	C7	C8	119.86(17)
C30	C25	C26	119.98(18)	C25	C30	C29	119.4(2)
C30	C25	N1	120.49(17)	C19	C20	C21	120.1(2)
C26	C25	N1	119.48(17)	C27	C26	C25	119.7(2)
O2	C16	N1	122.31(17)	C13	C14	C9	120.9(2)
O2	C16	C15	119.00(16)	C2	C3	C4	120.6(2)
N1	C16	C15	118.62(15)	C23	C24	C19	119.8(2)
C10	C9	C14	118.41(18)	C6	C5	C4	120.4(2)
C10	C9	C8	122.24(17)	C28	C29	C30	120.5(2)
C14	C9	C8	119.35(17)	C28	C27	C26	120.7(2)
C15	C8	C9	126.05(17)	C10	C11	C12	120.2(2)
C15	C8	C7	119.85(17)	C1	C6	C5	119.3(2)
C9	C8	C7	114.08(16)	C1	C2	C3	118.9(2)
C20	C19	C24	119.69(19)	C12	C13	C14	119.8(2)
C20	C19	N1	119.44(18)	C27	C28	C29	119.7(2)
C24	C19	N1	120.86(18)	C2	C1	C6	121.8(2)
C8	C15	C16	123.29(16)	C2	C1	Cl1	119.1(2)
C8	C15	C17	124.63(18)	C6	C1	Cl1	119.15(19)
C16	C15	C17	111.78(17)	C22	C21	C20	120.1(2)
C5	C4	C3	118.9(2)	C13	C12	C11	120.1(2)
C5	C4	C7	119.49(19)	C21	C22	C23	119.9(2)
C3	C4	C7	121.53(17)	C18	C17	C15	115.6(3)
C11	C10	C9	120.5(2)	C22	C23	C24	120.4(2)

Table S28. Torsion Angles for 6.

A	B	C	D	Angle/°	A	B	C	D	Angle/°
C16	N1	C25	C30	72.3(2)	C26	C25	C30	C29	0.2(3)
C19	N1	C25	C30	-102.4(2)	N1	C25	C30	C29	177.60(19)
C16	N1	C25	C26	-110.3(2)	C24	C19	C20	C21	-0.9(3)
C19	N1	C25	C26	75.0(2)	N1	C19	C20	C21	177.97(19)
C25	N1	C16	O2	-170.52(17)	C30	C25	C26	C27	-0.7(3)
C19	N1	C16	O2	4.1(3)	N1	C25	C26	C27	-178.12(19)
C25	N1	C16	C15	6.5(3)	C10	C9	C14	C13	-2.9(3)
C19	N1	C16	C15	-178.97(16)	C8	C9	C14	C13	177.01(19)
C10	C9	C8	C15	36.6(3)	C5	C4	C3	C2	2.3(3)
C14	C9	C8	C15	-143.3(2)	C7	C4	C3	C2	-175.7(2)
C10	C9	C8	C7	-141.41(18)	C20	C19	C24	C23	2.4(3)
C14	C9	C8	C7	38.7(2)	N1	C19	C24	C23	-176.4(2)
C16	N1	C19	C20	-130.55(19)	C3	C4	C5	C6	-2.5(3)
C25	N1	C19	C20	44.3(2)	C7	C4	C5	C6	175.59(19)
C16	N1	C19	C24	48.3(3)	C25	C30	C29	C28	0.3(4)
C25	N1	C19	C24	-136.85(19)	C25	C26	C27	C28	0.7(4)
C9	C8	C15	C16	-5.8(3)	C9	C10	C11	C12	-1.9(3)
C7	C8	C15	C16	172.13(17)	C4	C5	C6	C1	0.7(3)
C9	C8	C15	C17	-178.9(2)	C4	C3	C2	C1	-0.3(4)
C7	C8	C15	C17	-1.0(3)	C9	C14	C13	C12	0.5(4)
O2	C16	C15	C8	-109.8(2)	C26	C27	C28	C29	-0.1(4)
N1	C16	C15	C8	73.1(2)	C30	C29	C28	C27	-0.4(4)
O2	C16	C15	C17	64.2(3)	C3	C2	C1	C6	-1.5(4)
N1	C16	C15	C17	-112.9(2)	C3	C2	C1	Cl1	177.88(19)
C14	C9	C10	C11	3.5(3)	C5	C6	C1	C2	1.3(4)
C8	C9	C10	C11	-176.35(19)	C5	C6	C1	Cl1	-178.05(17)
C5	C4	C7	O1	5.0(3)	C19	C20	C21	C22	-1.4(4)
C3	C4	C7	O1	-176.9(2)	C14	C13	C12	C11	1.2(4)
C5	C4	C7	C8	-174.61(17)	C10	C11	C12	C13	-0.5(4)
C3	C4	C7	C8	3.4(3)	C20	C21	C22	C23	2.1(4)
C15	C8	C7	O1	-103.1(3)	C8	C15	C17	C18	97.2(3)
C9	C8	C7	O1	75.0(2)	C16	C15	C17	C18	-76.7(3)
C15	C8	C7	C4	76.5(2)	C21	C22	C23	C24	-0.6(4)
C9	C8	C7	C4	-105.3(2)	C19	C24	C23	C22	-1.7(4)

Table S29. Hydrogen Atom Coordinates (Å×10^4) and Isotropic Displacement Parameters (Å^2 ×10^3) for 6.

Atom	x	y	z	U(eq)
H10	3176	1989	3300	62
H30	5352	4346	3168	66
H20	4152	5618	3276	68
H26	4298	3502	4541	66
H14	5164	1029	4605	66
H3	5977	2340	3914	71
H24	2522	3628	2990	74
H5	6310	-320	3346	72
H29	6472	4580	4084	79
H27	5422	3719	5440	81

H11	2649	1955	4205	79
H6	7615	-44	3677	86
H2	7288	2607	4250	83
H13	4623	970	5500	81
H28	6504	4259	5214	82
H21	3232	6795	3135	88
H12	3372	1452	5303	89
H22	1957	6389	2865	97
H17A	5003	3023	2187	97
H17B	5366	2020	2411	97
H23	1604	4810	2801	94
H18A	4736	2043	1251	183
H18B	3972	2322	1399	183
H18C	4338	1318	1623	183

NMR Spectra of New Compounds (^{1}H NMR, ^{19}F NMR, ^{13}C NMR)

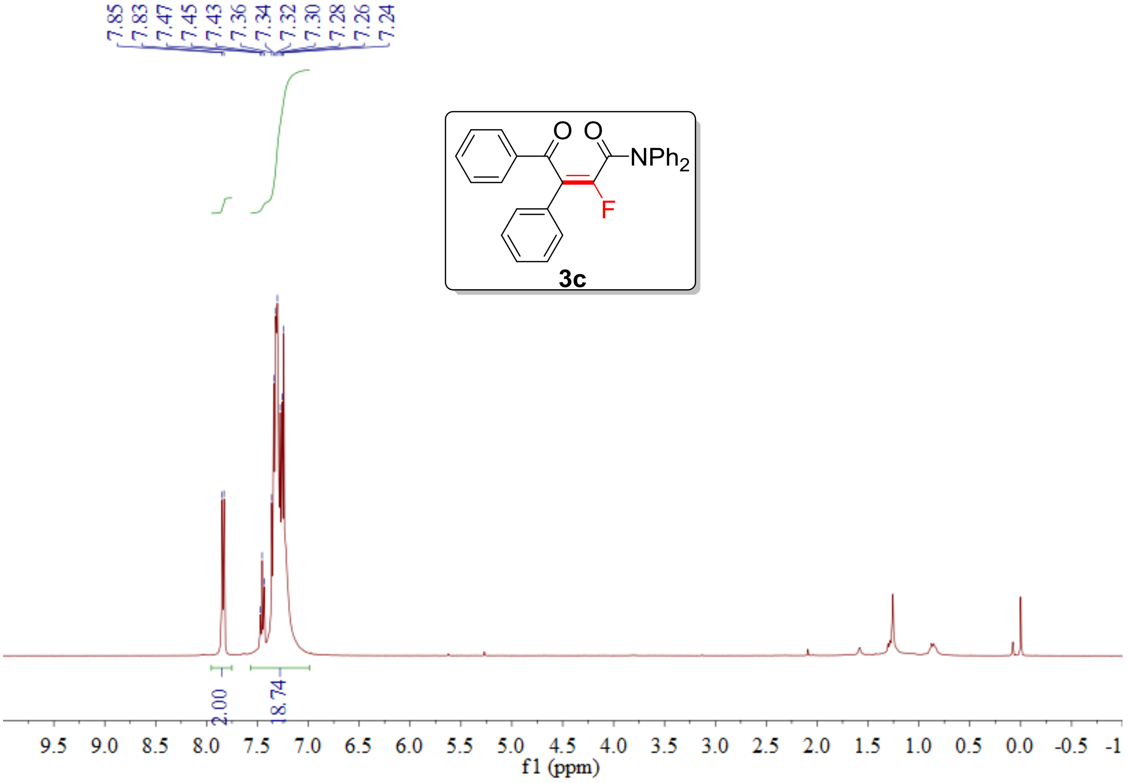

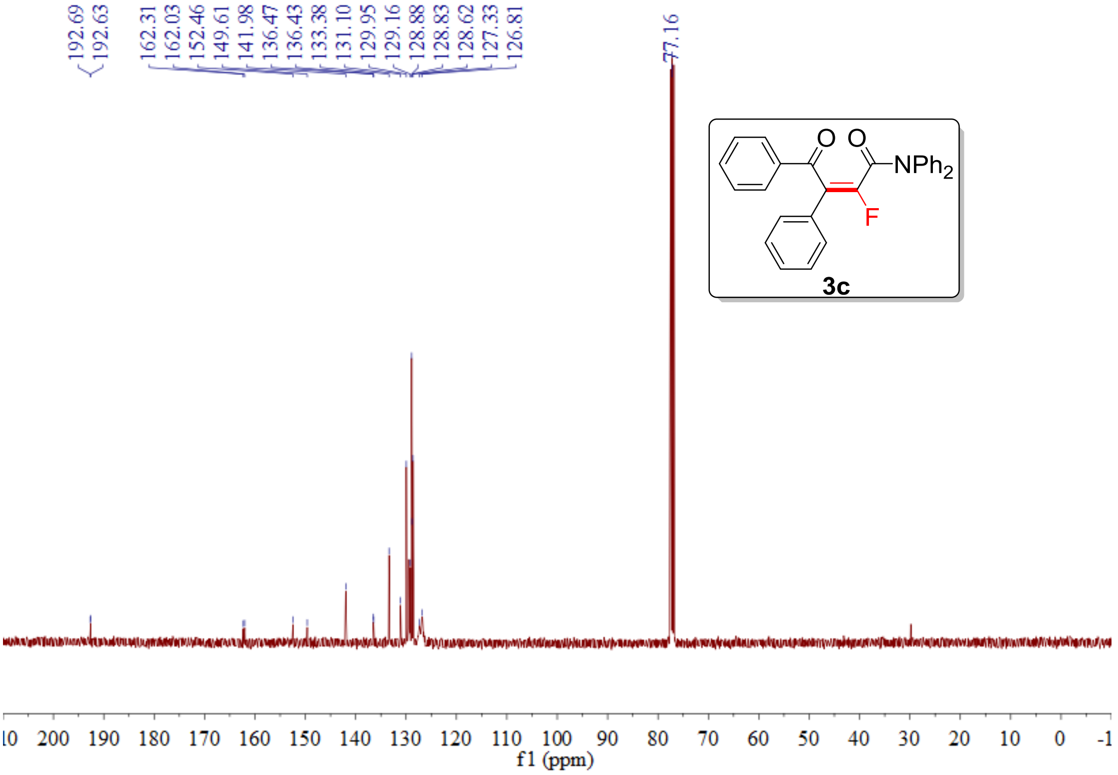

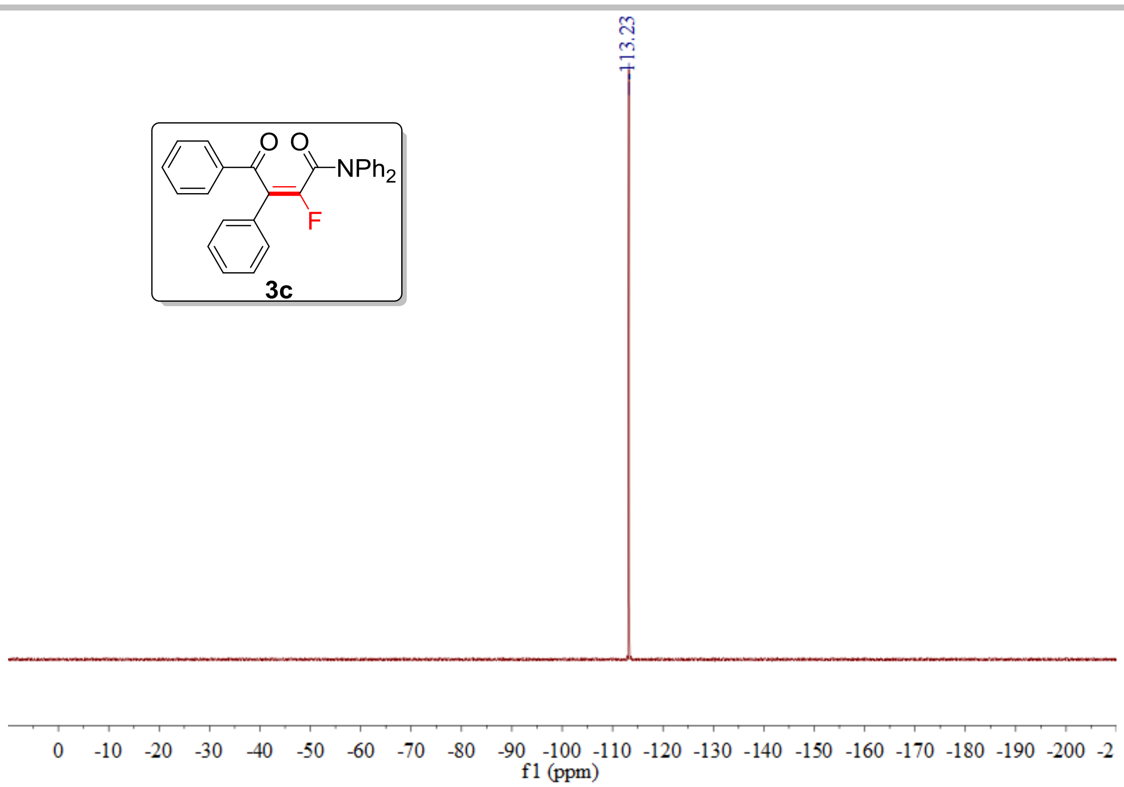
-113.23
NPh2
F
3c
0 -10 -20 -30 -40 -50 -60 -70 -80 -90 -100 -110 -120 -130 -140 -150 -160 -170 -180 -190 -200
f1 (ppm)

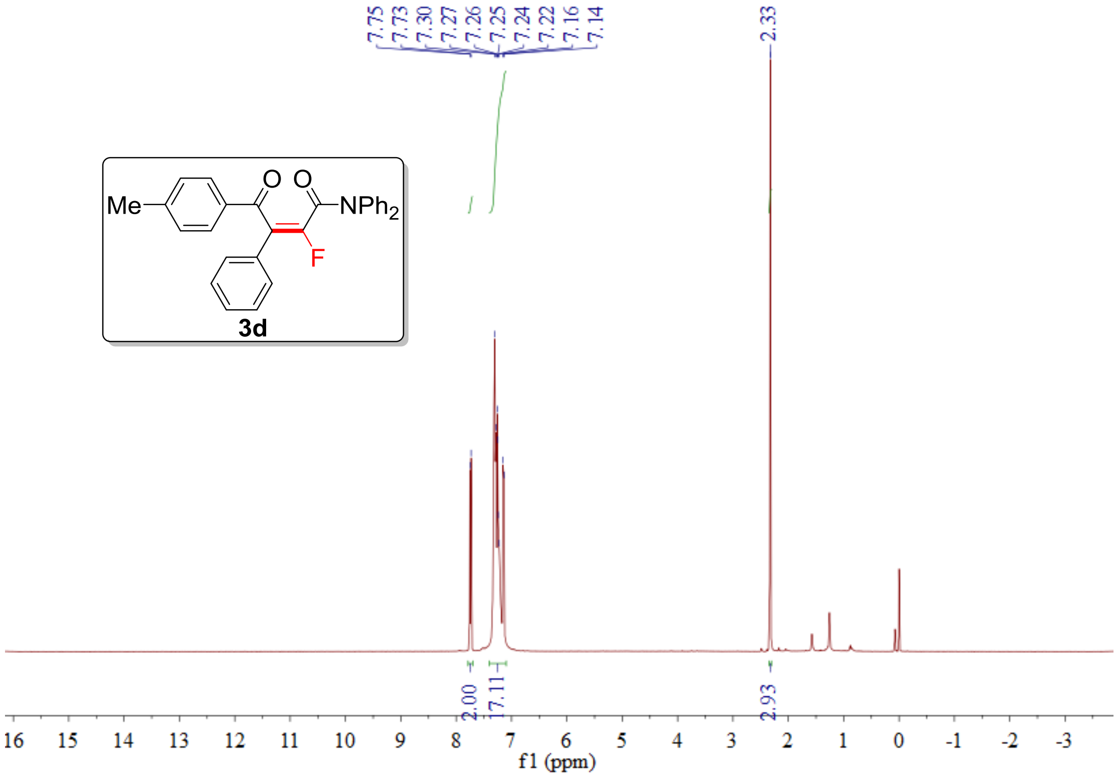
7.75 7.73 7.30 7.27 7.26 7.25 7.24 7.22 7.16 7.14
2.33
Me
NPh2
F
3d
2.00
17.11
2.93
16 15 14 13 12 11 10 9 8 7 6 5 4 3 2 1 0 -1 -2 -3
f1 (ppm)

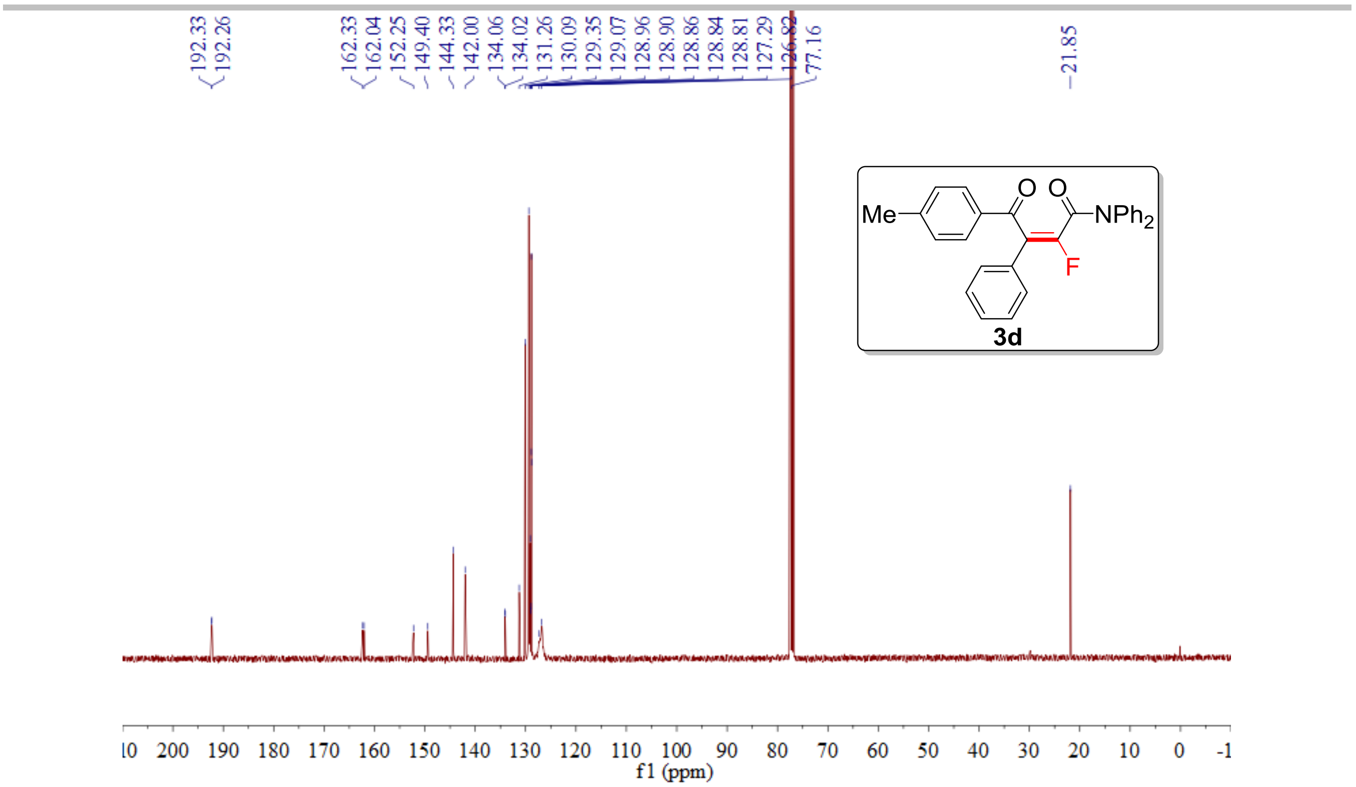
192.33
192.26
162.33
162.04
152.25
149.40
144.33
142.00
134.06
134.02
131.26
130.09
129.35
129.07
128.96
128.90
128.86
128.84
128.81
127.29
126.82
77.16
21.85
Me
O
O
NPh2
F
3d
f1 (ppm)

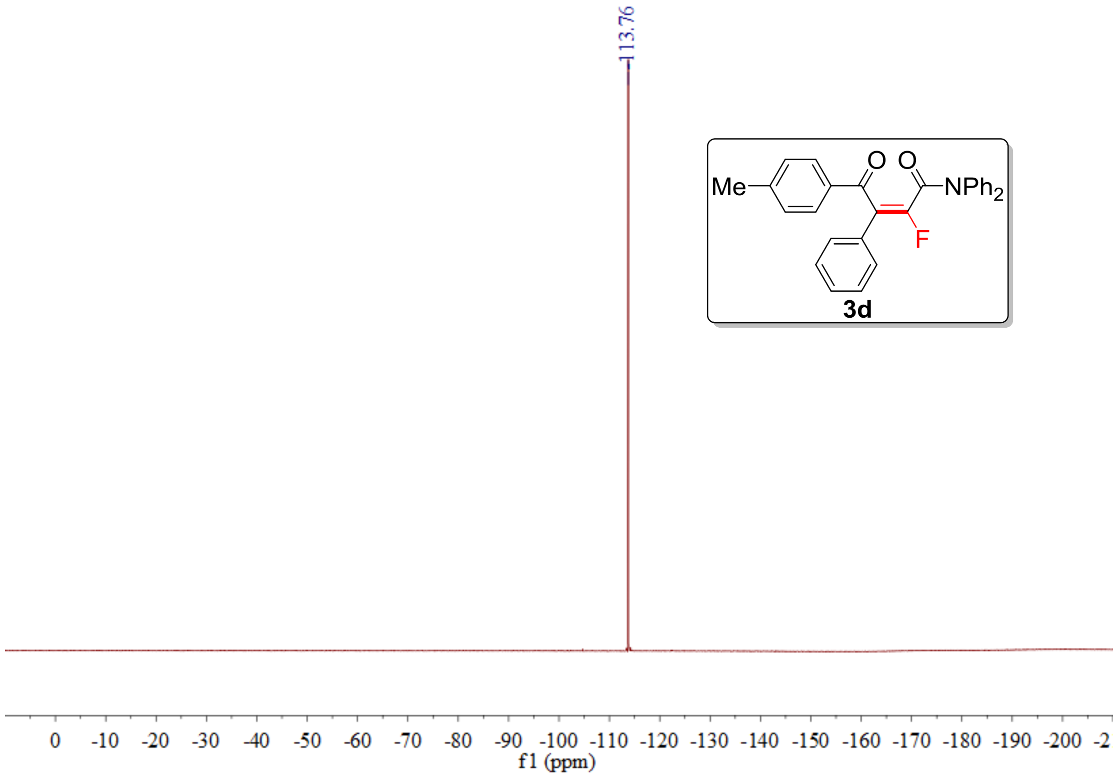
-113.76
Me
O
O
NPh2
F
3d
f1 (ppm)

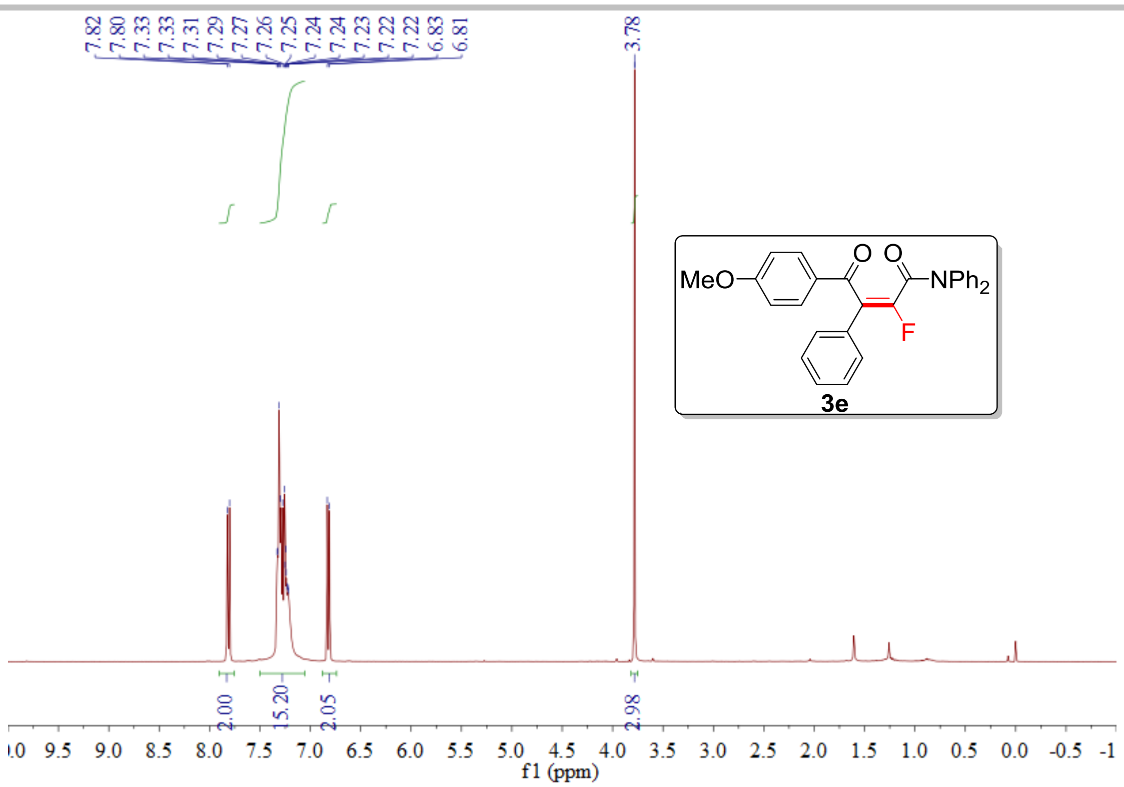
7.82 7.80 7.33 7.33 7.31 7.29 7.27 7.26 7.25 7.24 7.24 7.23 7.22 7.22 6.83 6.81
3.78
MeO
O O
NPh2
F
3e
2.00 15.20 2.05 2.98
f1 (ppm)

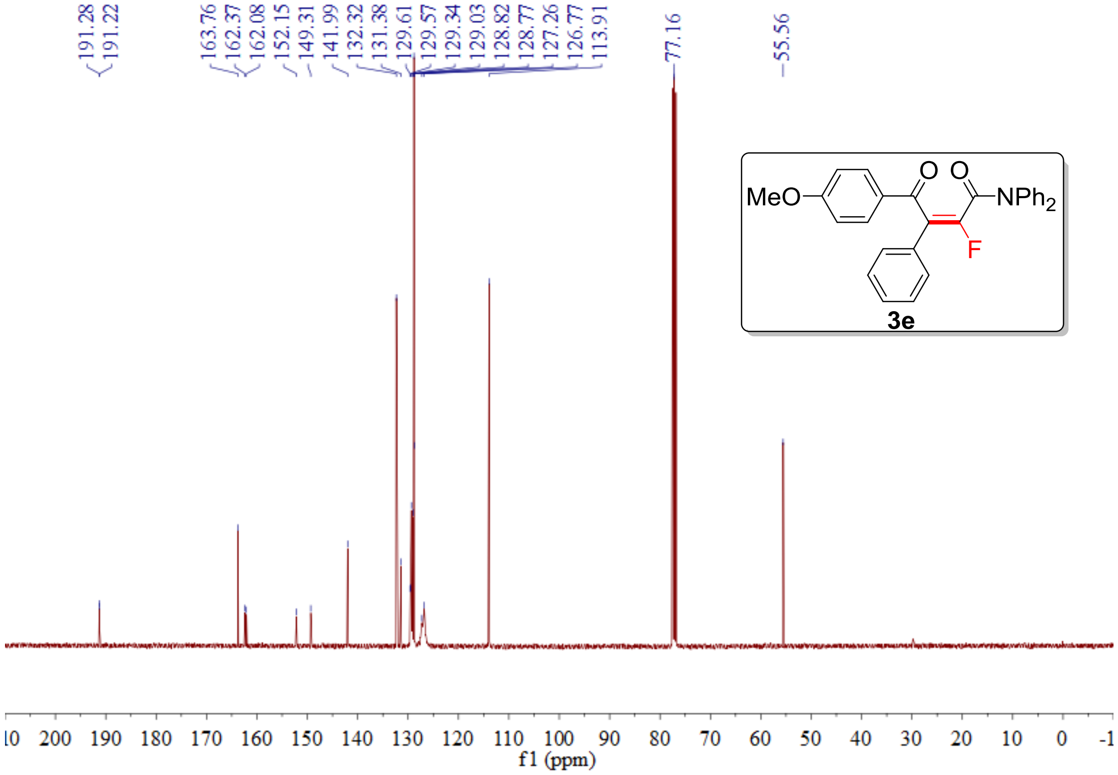
191.28 191.22
163.76 162.37 162.08 152.15 149.31 141.99 132.32 131.38 129.61 129.57 129.34 129.03 128.82 128.77 127.26 126.77 113.91
77.16
55.56
MeO
O O
NPh2
F
3e
f1 (ppm)

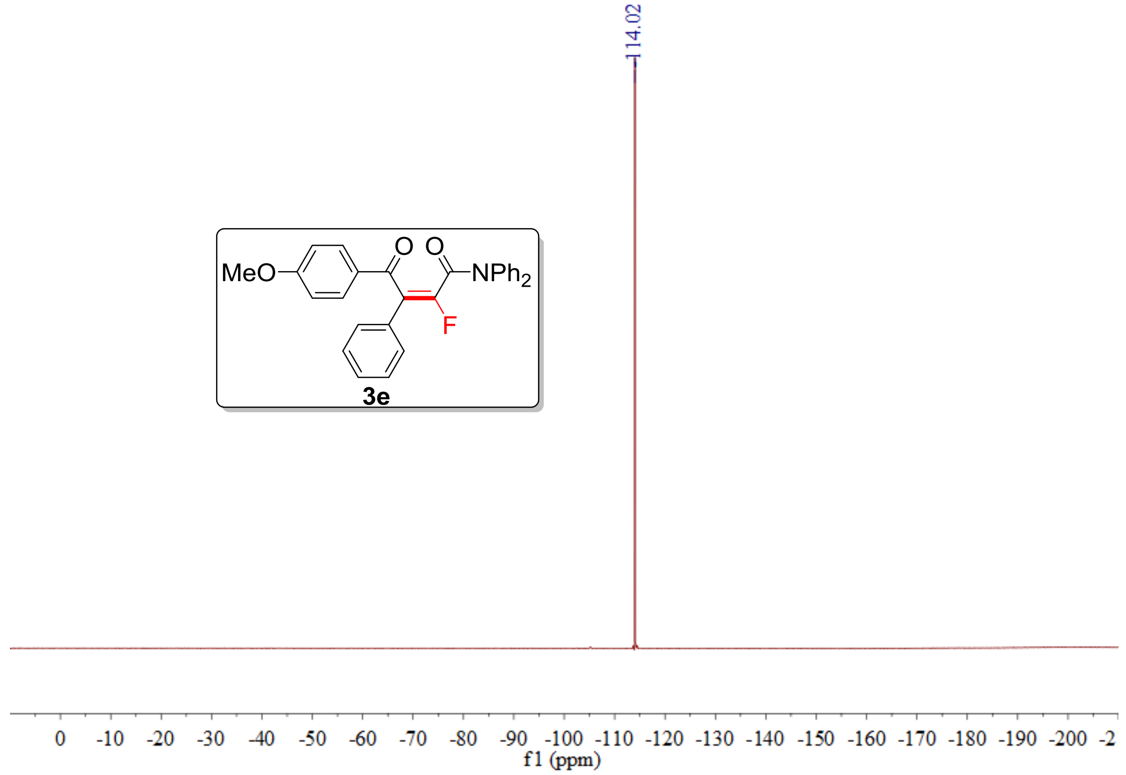
-114.02
MeO
O
O
NPh2
F
3e
0 -10 -20 -30 -40 -50 -60 -70 -80 -90 -100 -110 -120 -130 -140 -150 -160 -170 -180 -190 -200
f1 (ppm)

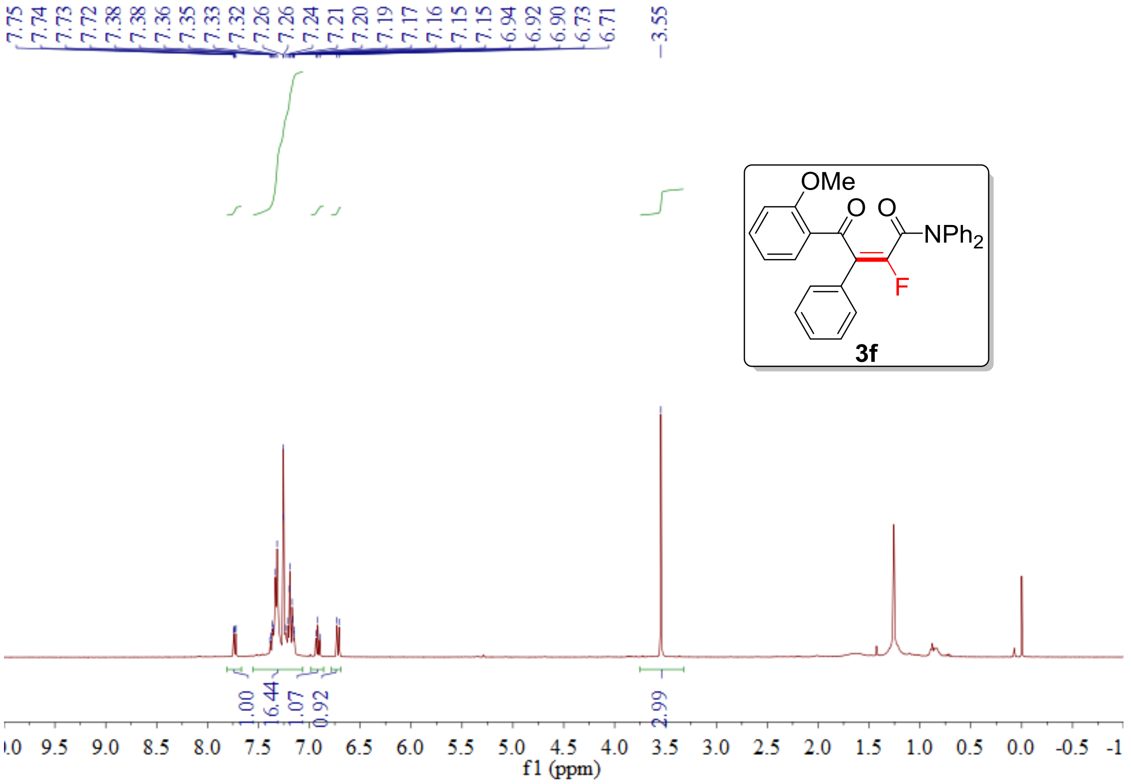
7.75 7.74 7.73 7.72 7.38 7.38 7.36 7.35 7.33 7.32 7.26 7.26 7.24 7.21 7.20 7.19 7.17 7.16 7.15 7.15 6.94 6.92 6.90 6.73 6.71
3.55
OMe
O
O
NPh2
F
3f
1.00
16.44
1.07
0.92
2.99
9.5 9.0 8.5 8.0 7.5 7.0 6.5 6.0 5.5 5.0 4.5 4.0 3.5 3.0 2.5 2.0 1.5 1.0 0.5 0.0 -0.5
f1 (ppm)

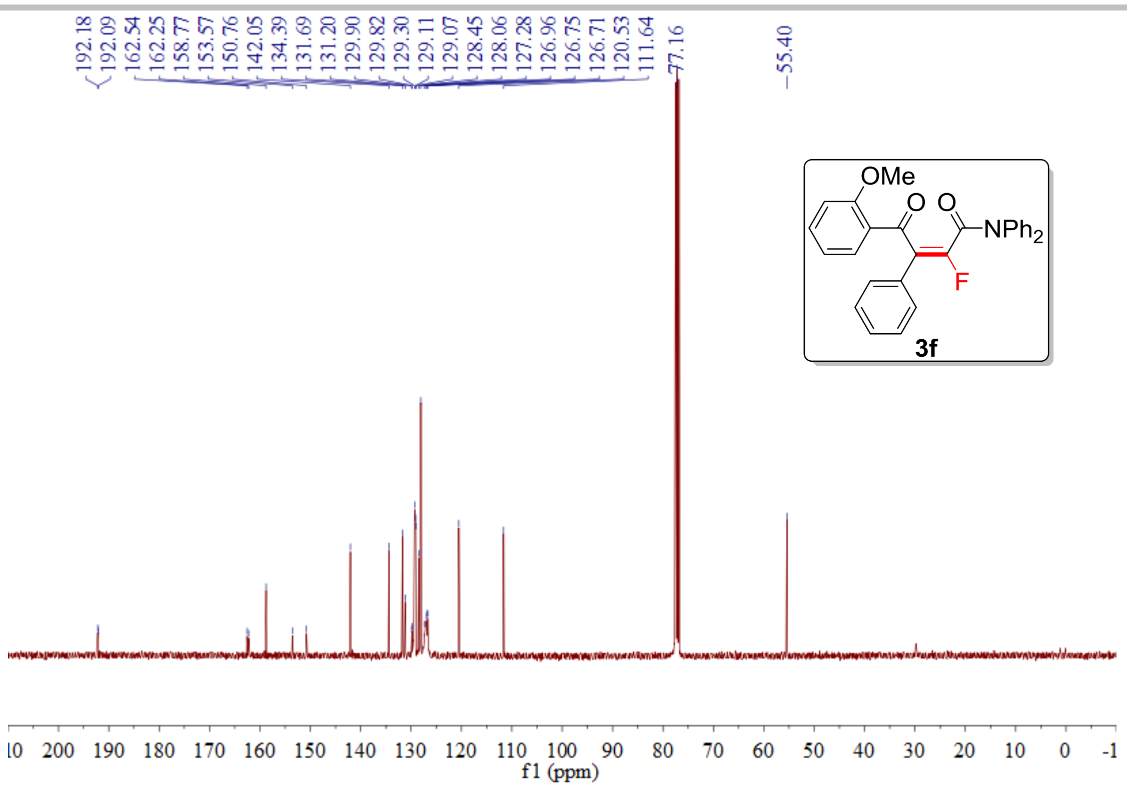
192.18
192.09
162.54
162.25
158.77
153.57
150.76
142.05
134.39
131.69
131.20
129.90
129.82
129.30
129.11
129.07
128.45
128.06
127.28
126.96
126.75
126.71
120.53
111.64
77.16
55.40
OMe
O
O
NPh2
F
3f
f1 (ppm)

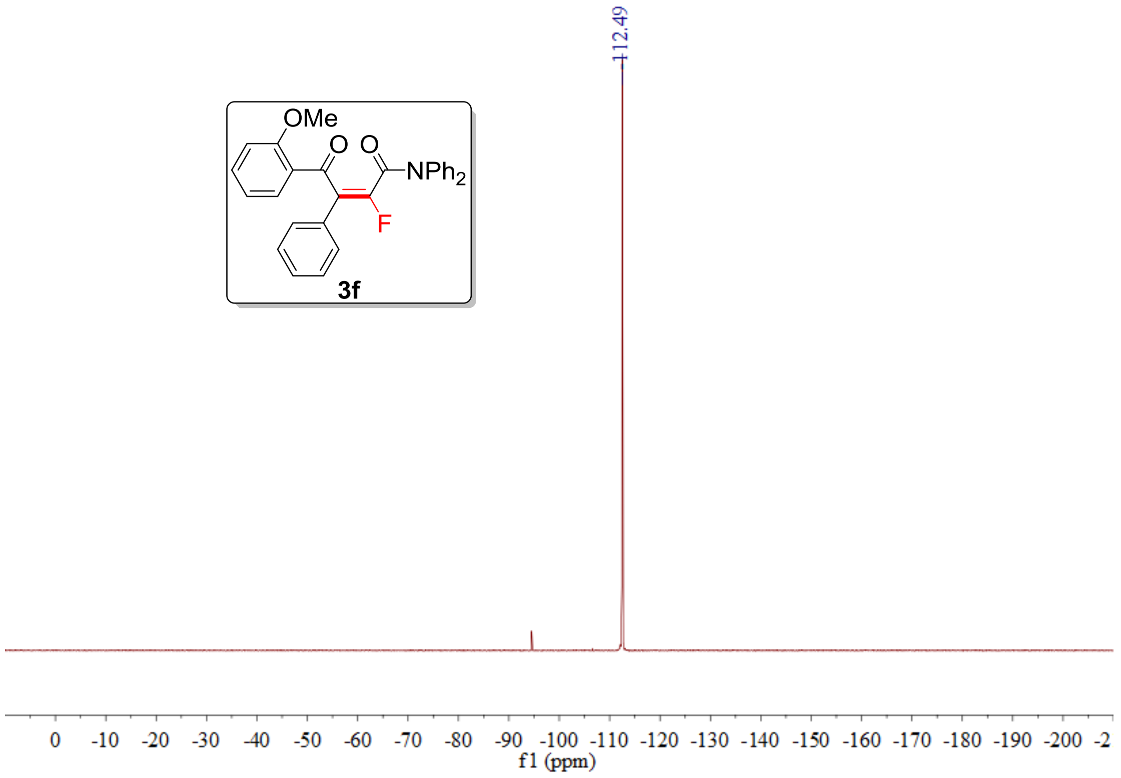
-112.49
OMe
O
O
NPh2
F
3f
f1 (ppm)

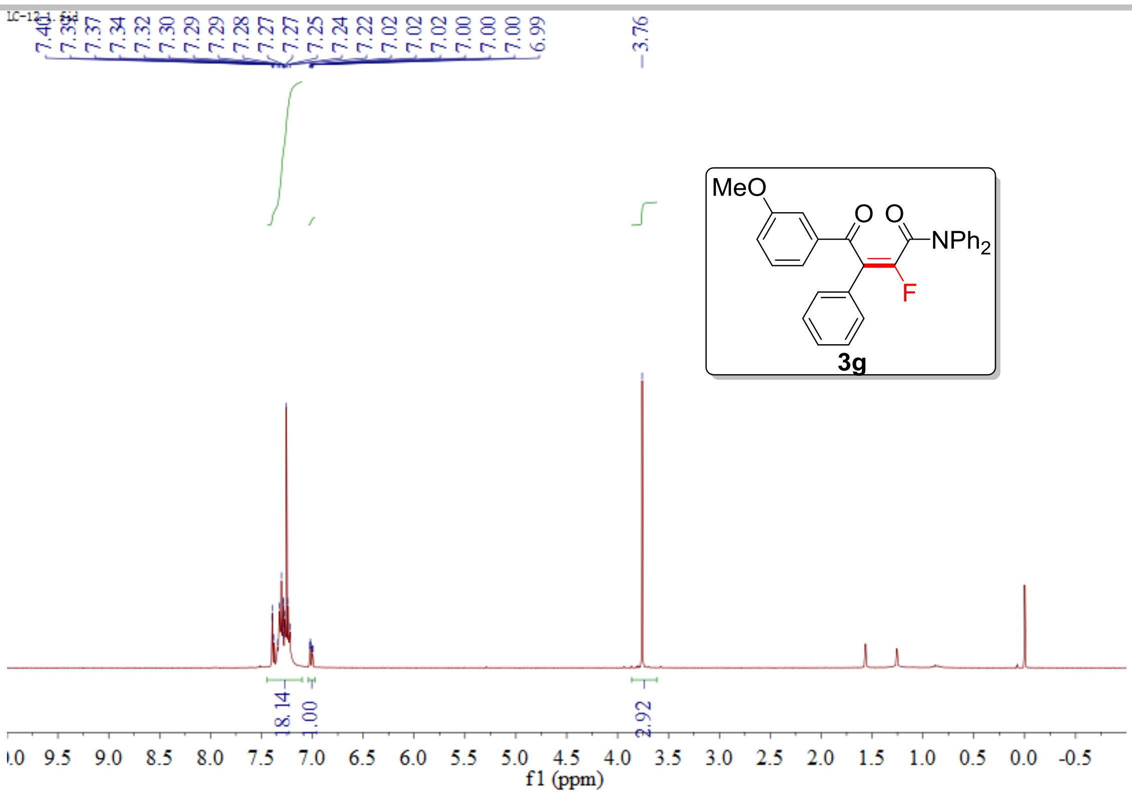

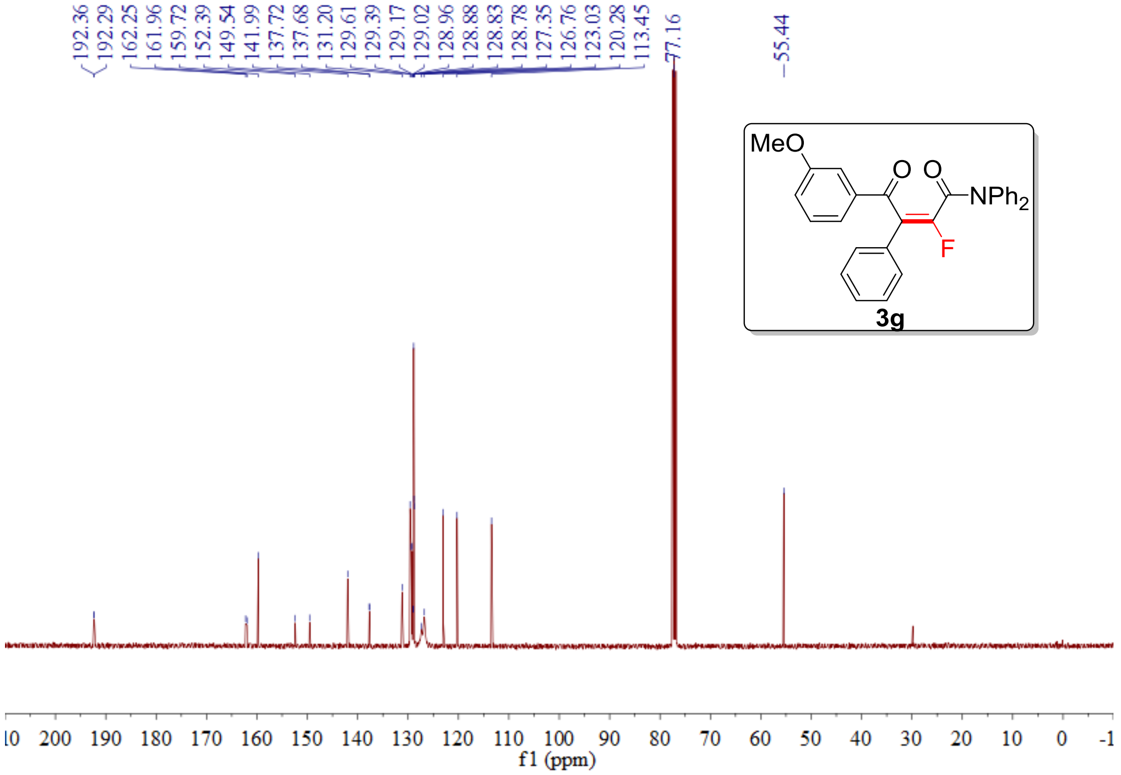

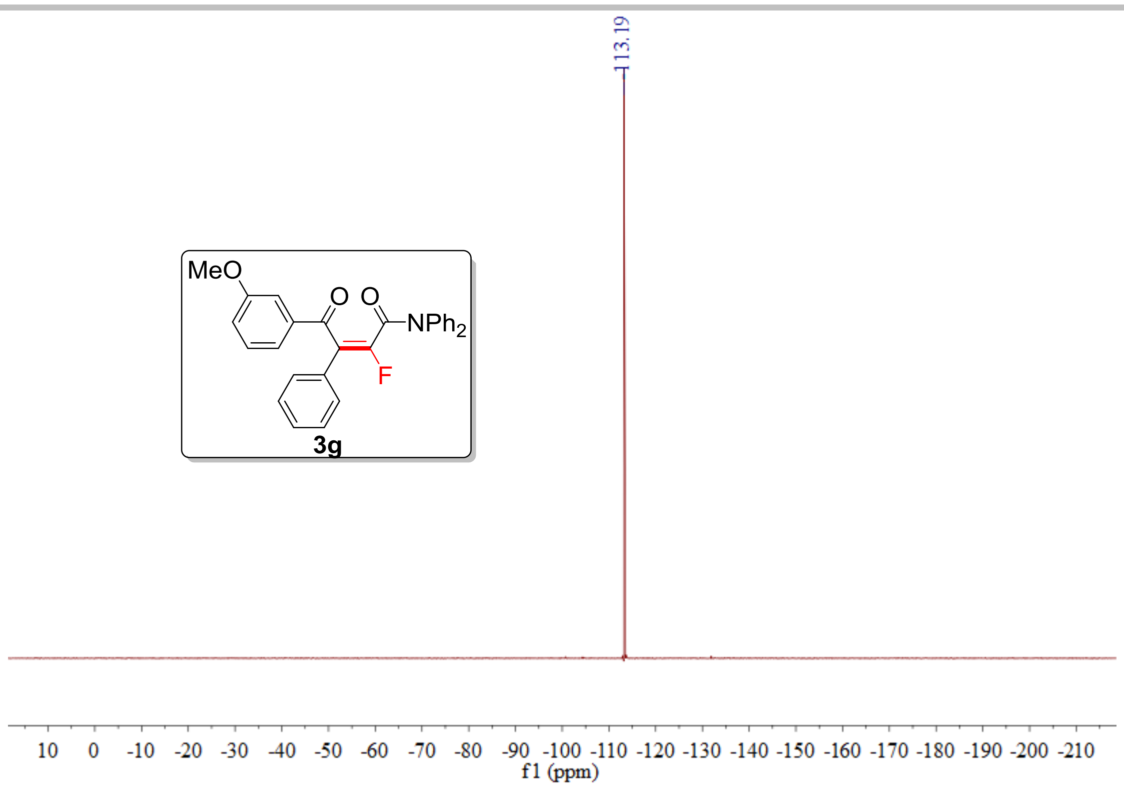
-113.19
MeO
O
O
NPh$_2$
F
3g
10 0 -10 -20 -30 -40 -50 -60 -70 -80 -90 -100 -110 -120 -130 -140 -150 -160 -170 -180 -190 -200 -210
f1 (ppm)

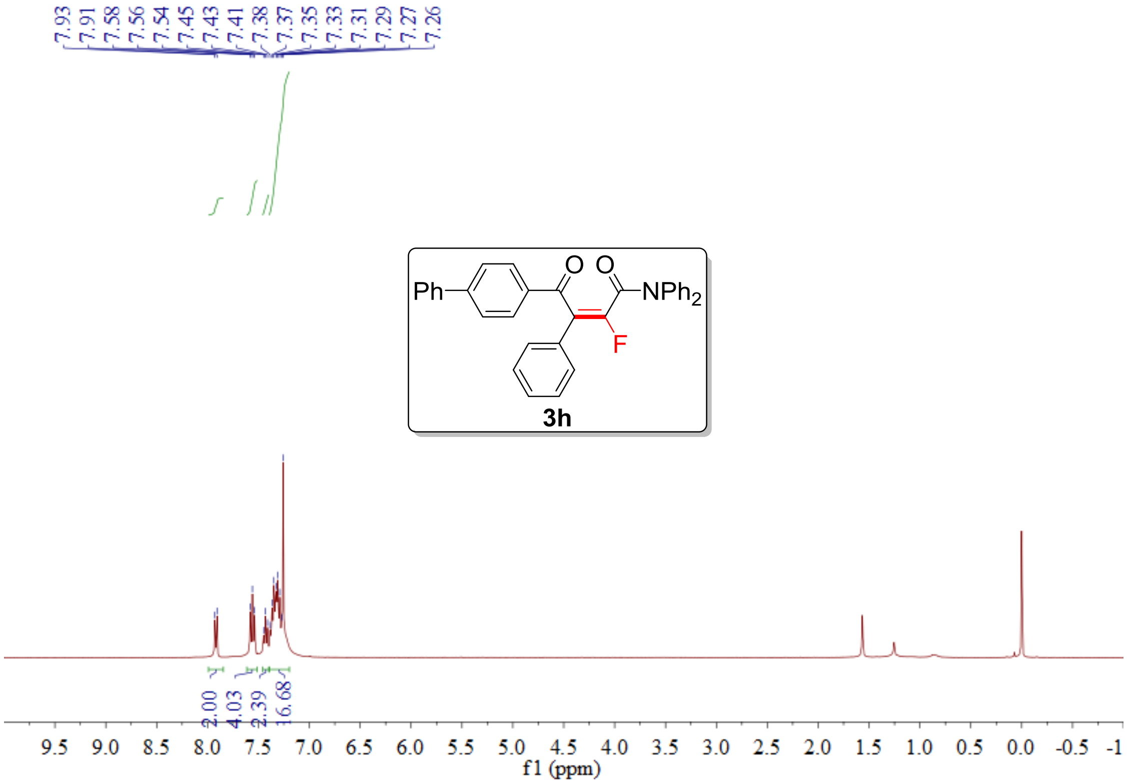
7.93
7.91
7.58
7.56
7.54
7.45
7.43
7.41
7.38
7.37
7.35
7.33
7.31
7.29
7.27
7.26
Ph
O
O
NPh$_2$
F
3h
2.00
4.03
2.39
16.68
9.5 9.0 8.5 8.0 7.5 7.0 6.5 6.0 5.5 5.0 4.5 4.0 3.5 3.0 2.5 2.0 1.5 1.0 0.5 0.0 -0.5 -1
f1 (ppm)

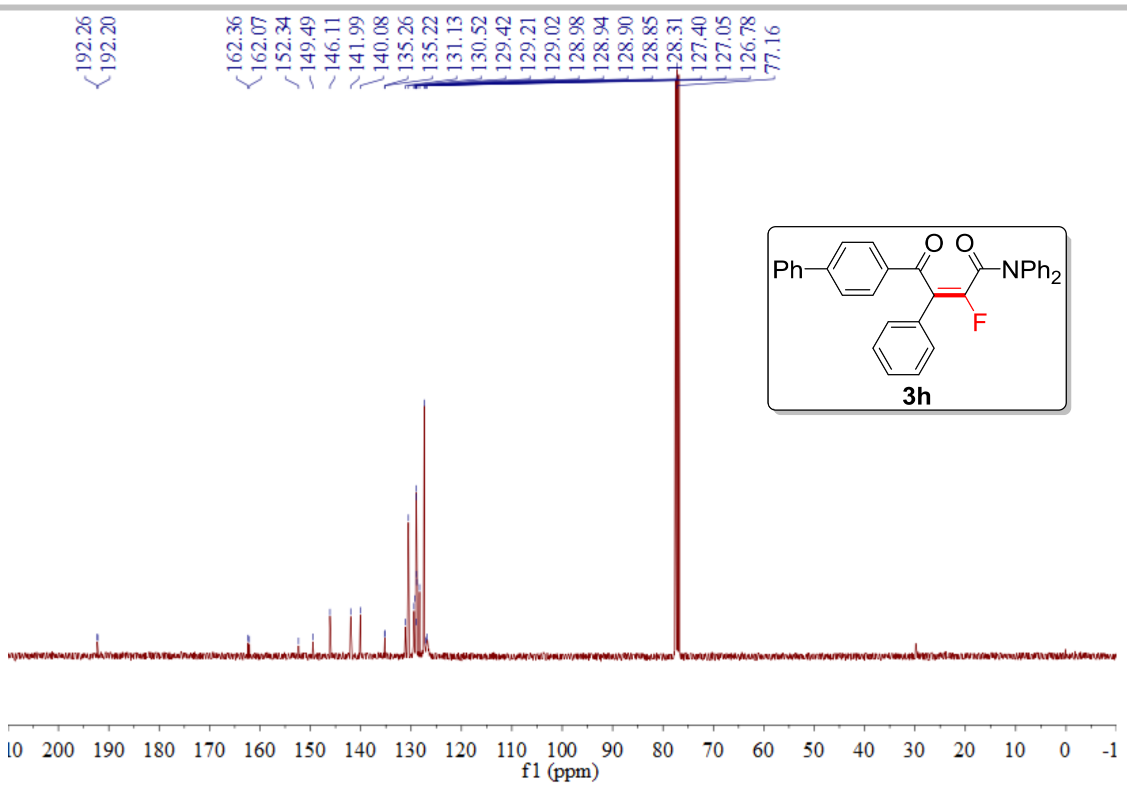
192.26
192.20
162.36
162.07
152.34
149.49
146.11
141.99
140.08
135.26
135.22
131.13
130.52
129.42
129.21
129.02
128.98
128.94
128.90
128.85
128.31
127.40
127.05
126.78
77.16
Ph
O
O
NPh2
F
3h
f1 (ppm)

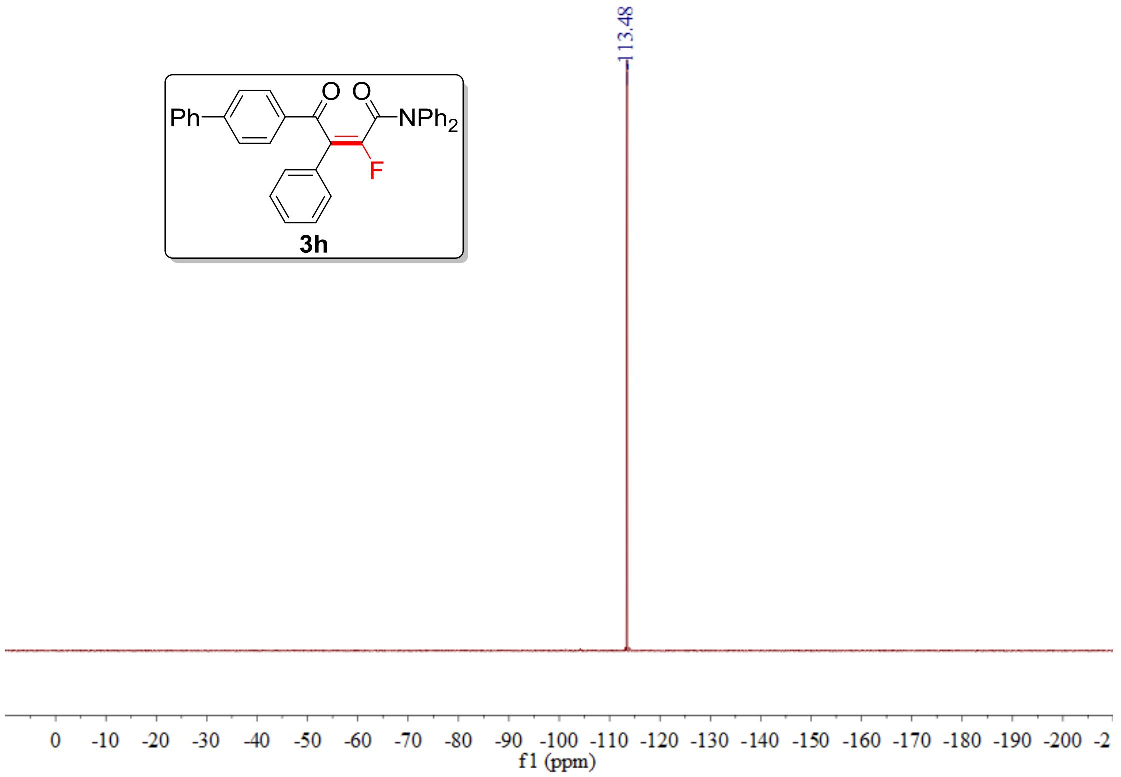
-113.48
Ph
O
O
NPh2
F
3h
f1 (ppm)

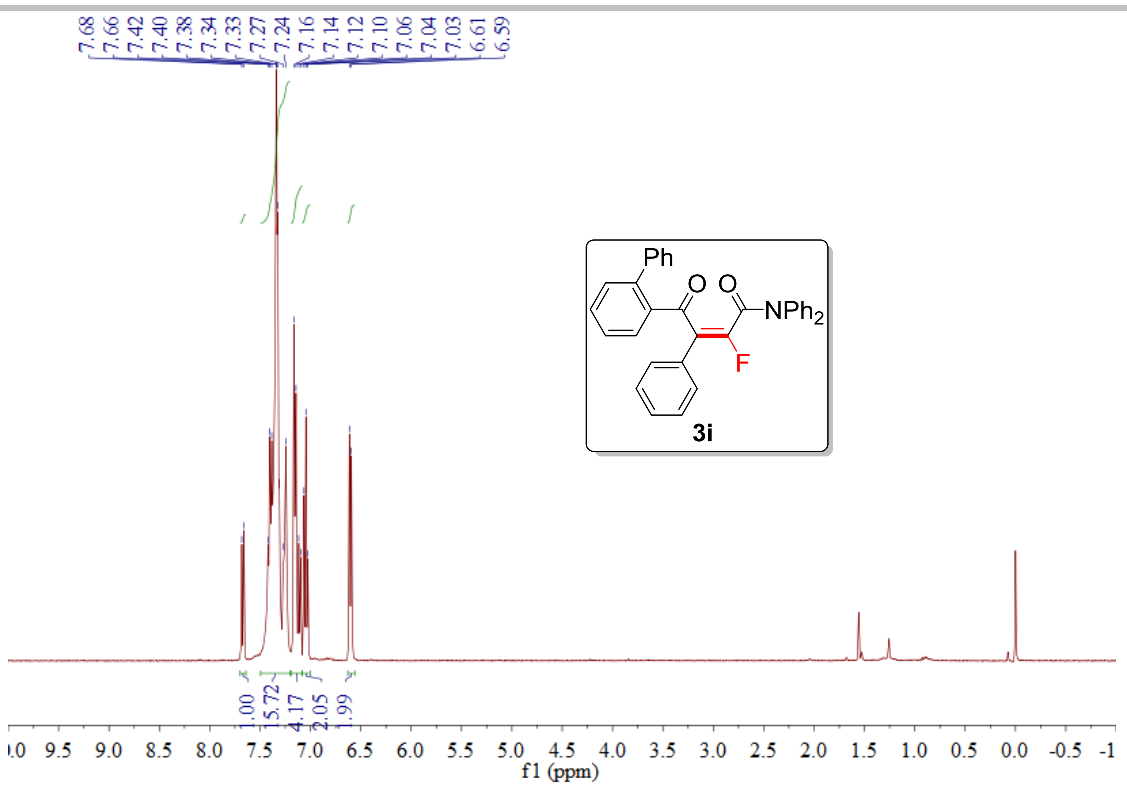
7.68
7.66
7.42
7.40
7.38
7.34
7.33
7.27
7.24
7.16
7.14
7.12
7.10
7.06
7.04
7.03
6.61
6.59
1.00
5.72
4.17
2.05
1.99
Ph
O
O
NPh2
F
3i
f1 (ppm)

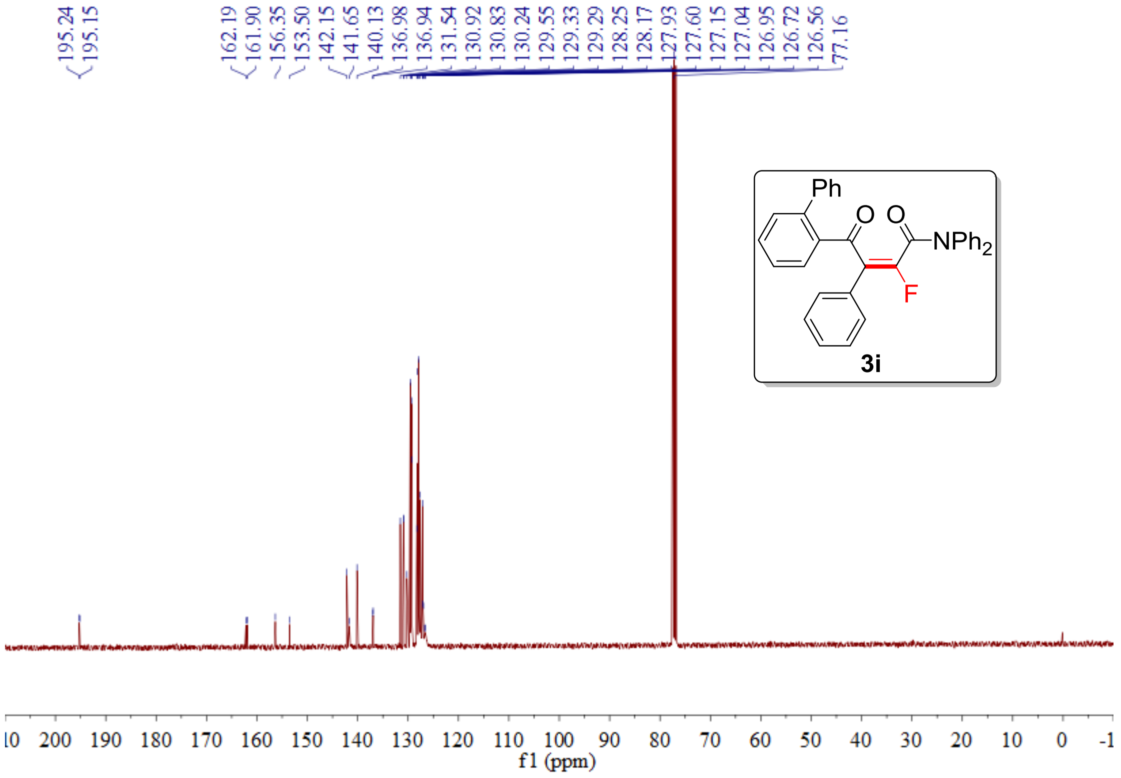
195.24
195.15
162.19
161.90
156.35
153.50
142.15
141.65
140.13
136.98
136.94
131.54
130.92
130.83
130.24
129.55
129.33
129.29
128.25
128.17
127.93
127.60
127.15
127.04
126.95
126.72
126.56
77.16
Ph
O
O
NPh2
F
3i
f1 (ppm)

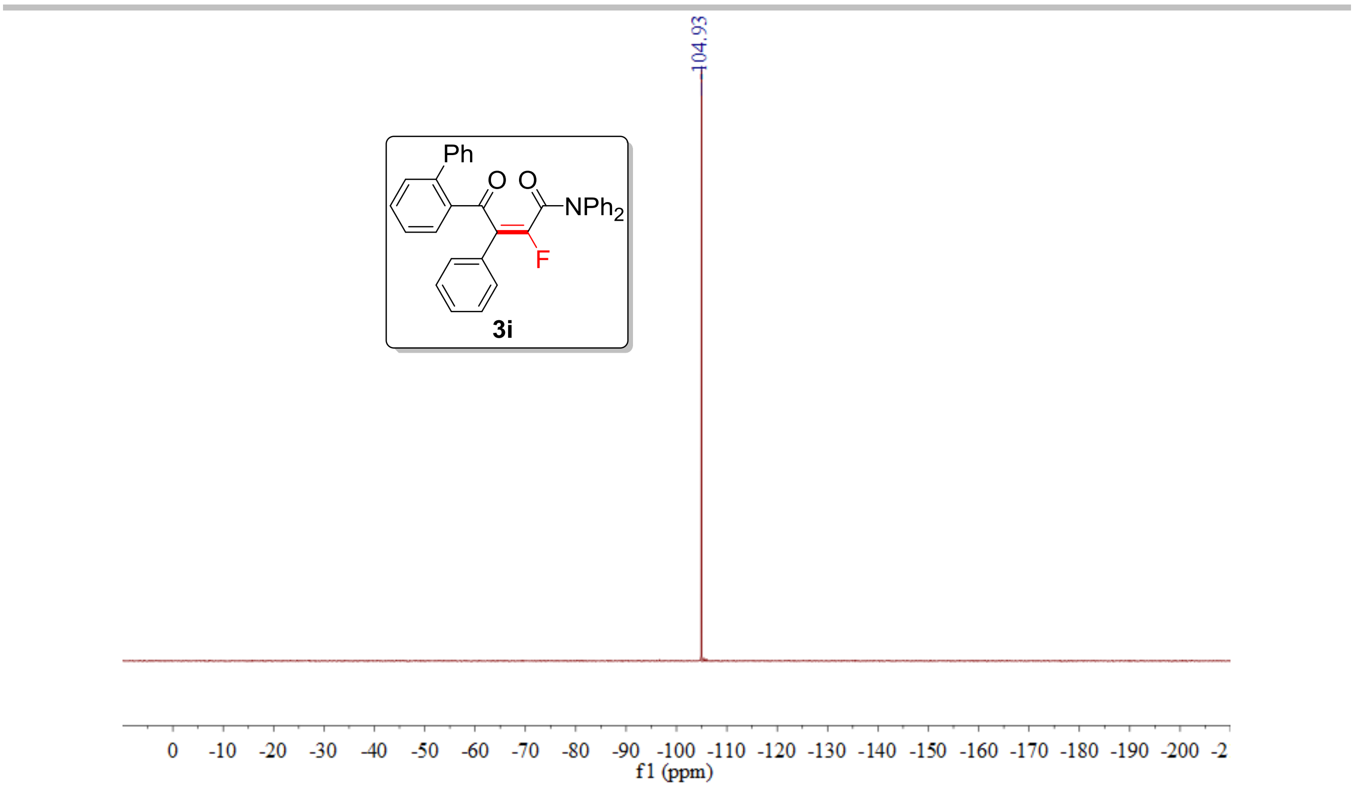
-104.93
Ph
O
O
NPh2
F
3i
f1 (ppm)

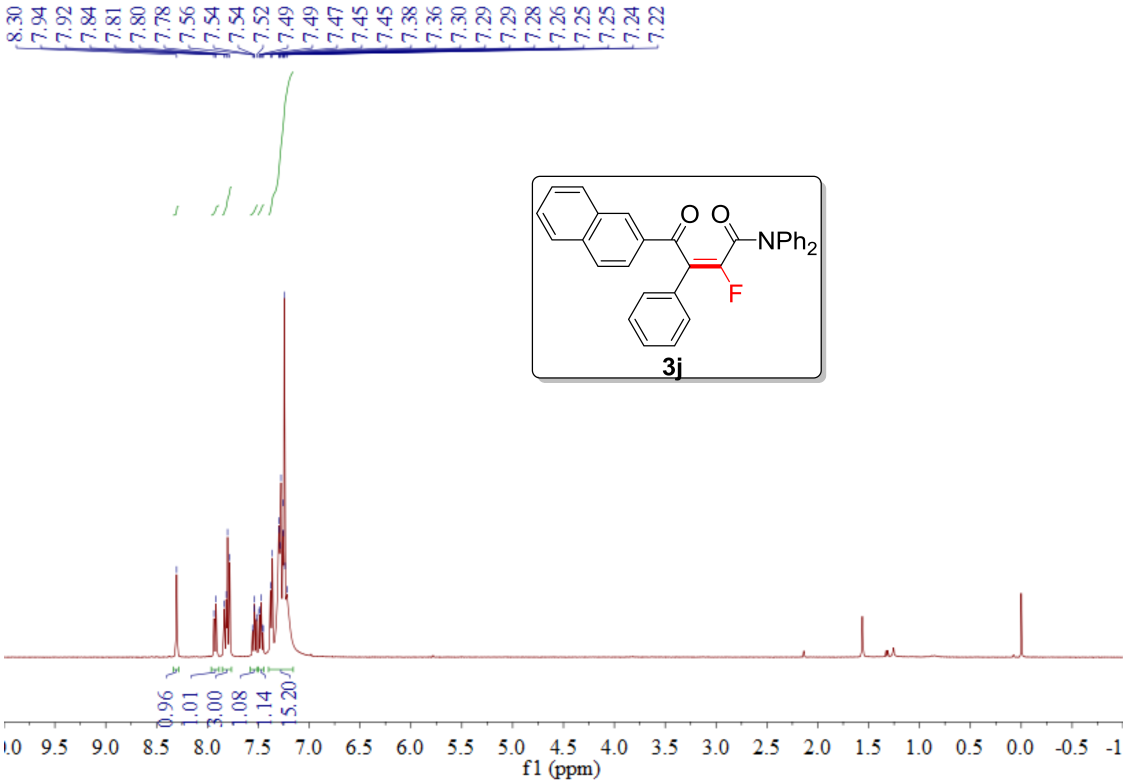
8.30
7.94
7.92
7.84
7.81
7.80
7.78
7.56
7.54
7.54
7.52
7.49
7.49
7.47
7.45
7.45
7.38
7.36
7.30
7.29
7.29
7.28
7.26
7.25
7.25
7.24
7.22
O
O
NPh2
F
3j
0.96
1.01
3.00
1.08
1.14
15.20
f1 (ppm)

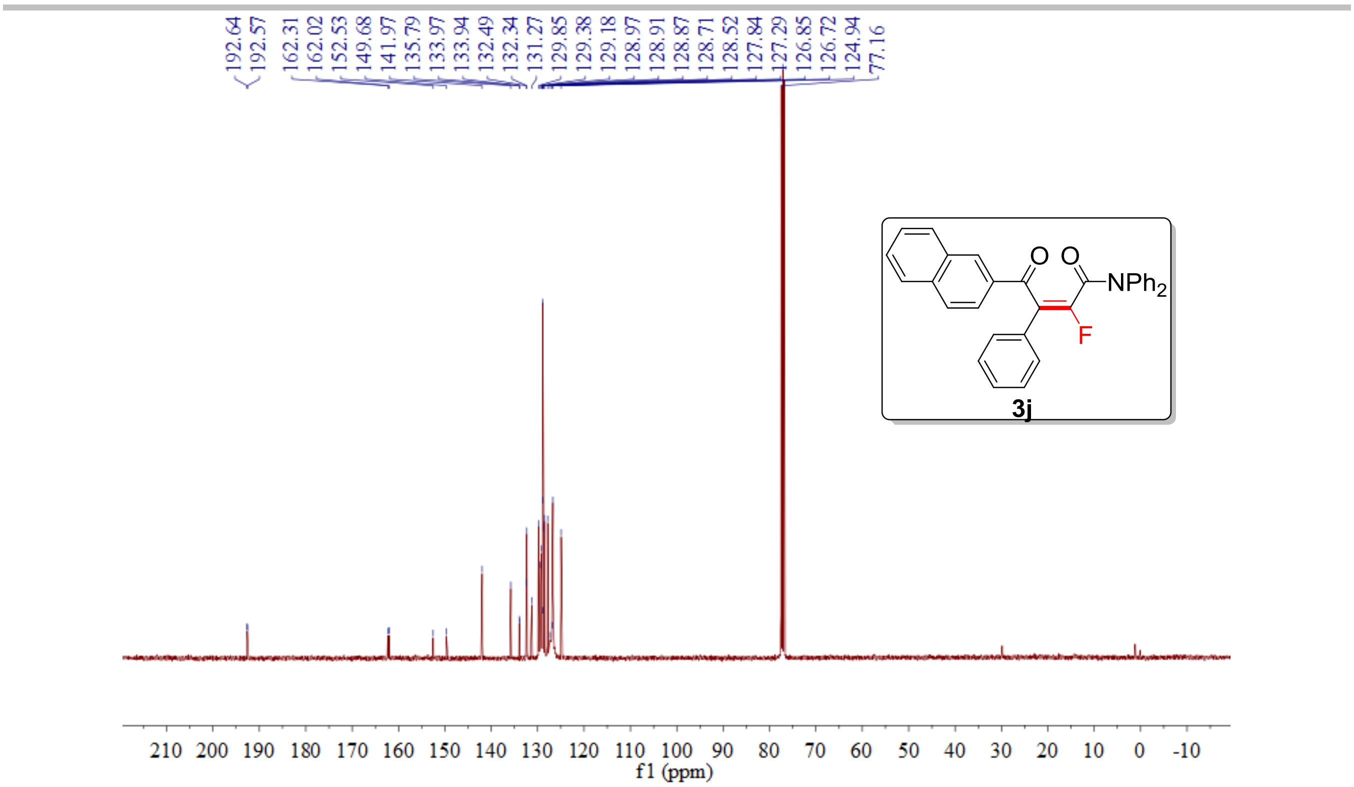
192.64
192.57
162.31
162.02
152.53
149.68
141.97
135.79
133.97
133.94
132.49
132.34
131.27
129.85
129.38
129.18
128.97
128.91
128.87
128.71
128.52
127.84
127.29
126.85
126.72
124.94
77.16
O O
NPh2
F
3j
210 200 190 180 170 160 150 140 130 120 110 100 90 80 70 60 50 40 30 20 10 0 -10
f1 (ppm)

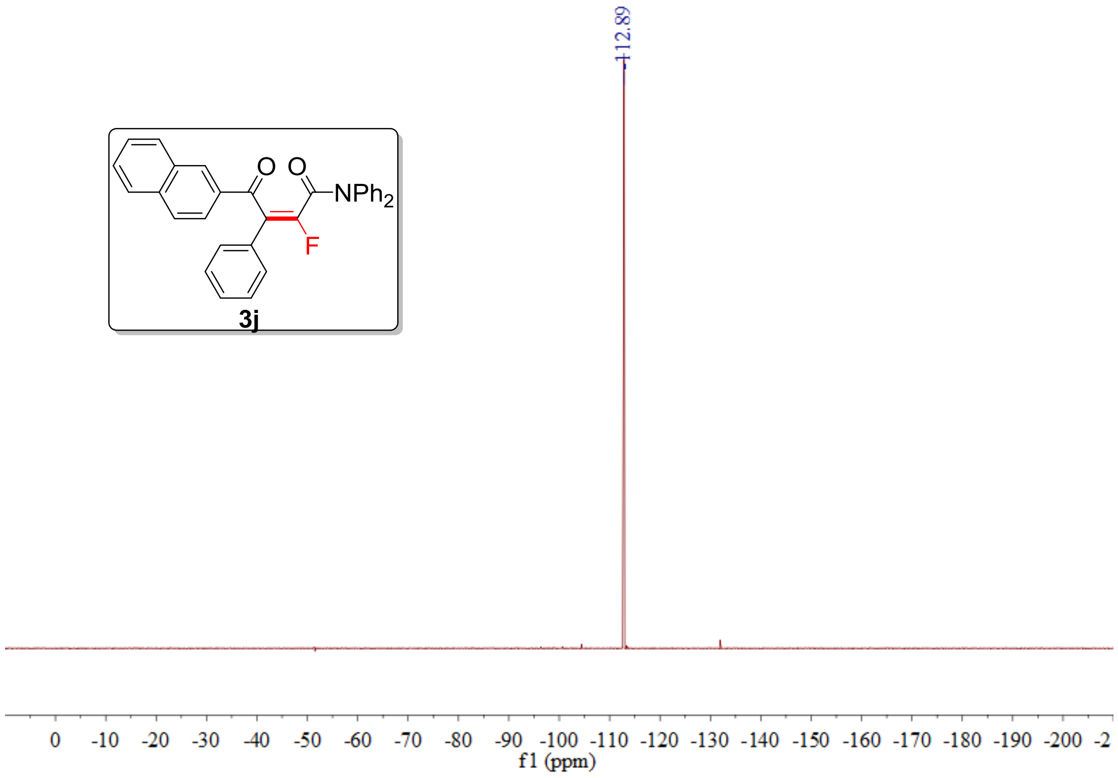
-112.89
O O
NPh2
F
3j
0 -10 -20 -30 -40 -50 -60 -70 -80 -90 -100 -110 -120 -130 -140 -150 -160 -170 -180 -190 -200 -2
f1 (ppm)

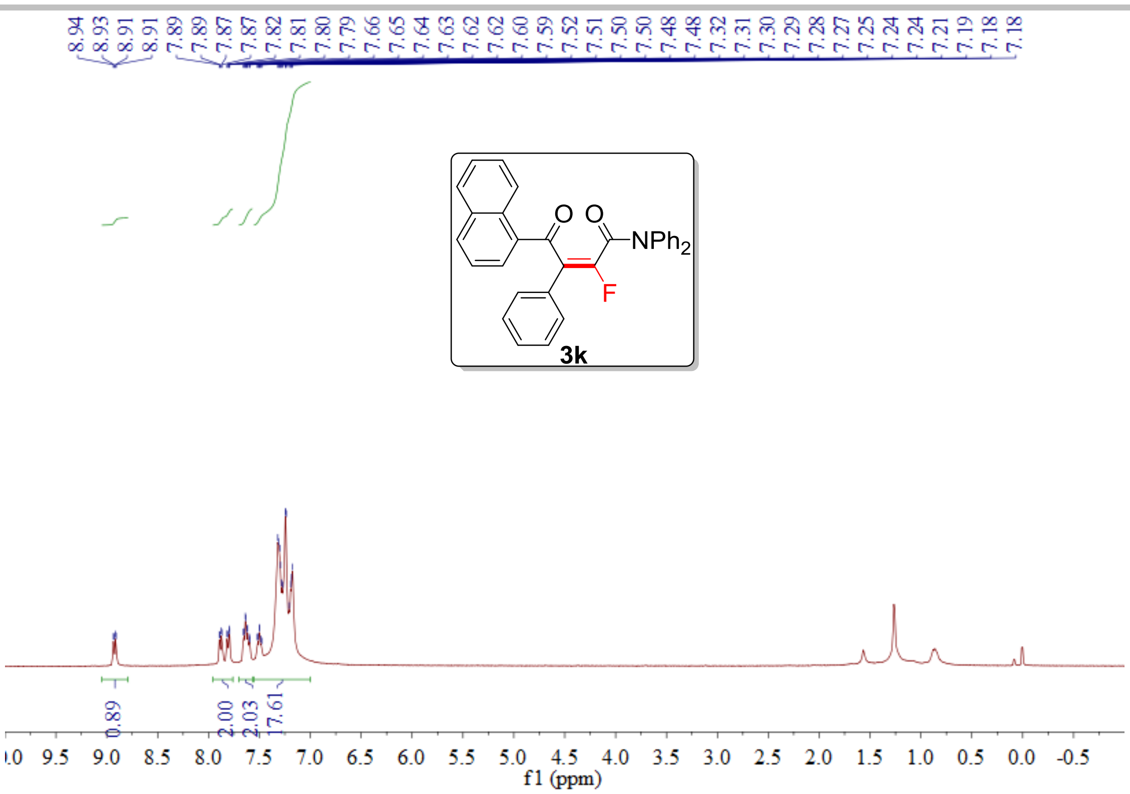
8.94 8.93 8.91 8.91 7.89 7.89 7.87 7.87 7.82 7.81 7.80 7.79 7.66 7.65 7.64 7.63 7.62 7.62 7.60 7.59 7.52 7.51 7.50 7.50 7.48 7.48 7.32 7.31 7.30 7.29 7.28 7.27 7.25 7.24 7.24 7.21 7.19 7.18 7.18
NPh2
F
3k
0.89
2.00
2.03
17.61
f1 (ppm)

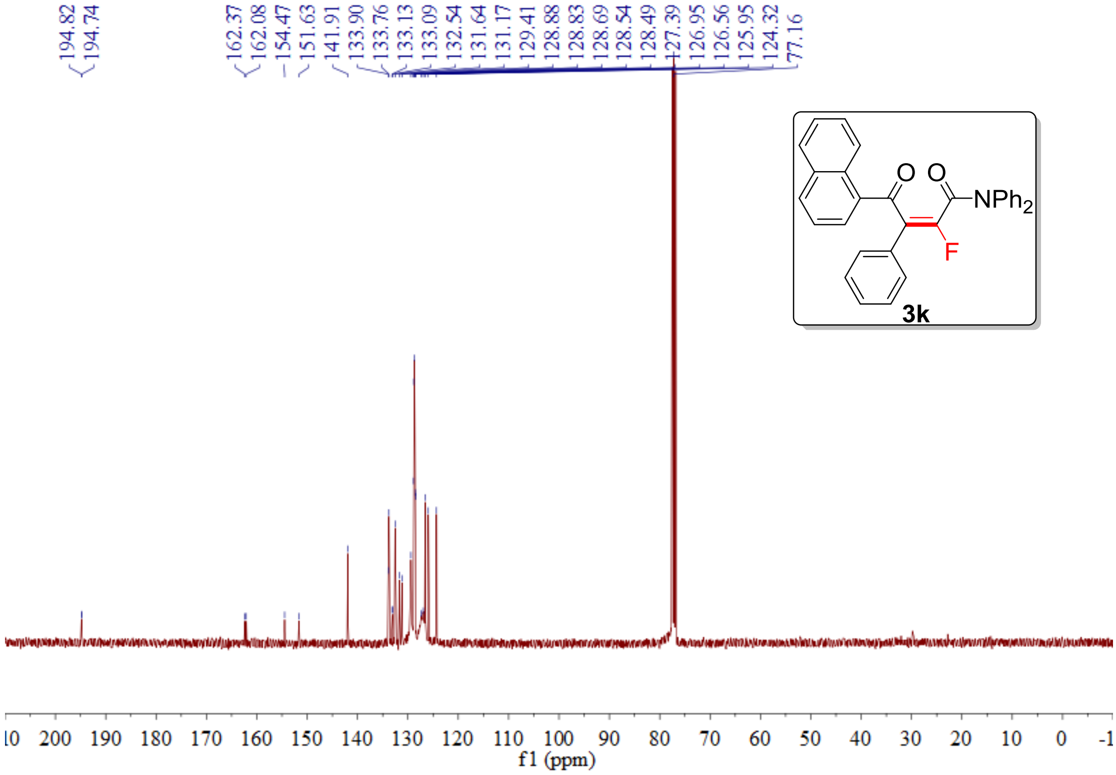
194.82 194.74
162.37 162.08 154.47 151.63 141.91 133.90 133.76 133.13 133.09 132.54 131.64 131.17 129.41 128.88 128.83 128.69 128.54 128.49 127.39 126.95 126.56 125.95 124.32 77.16
NPh2
F
3k
f1 (ppm)

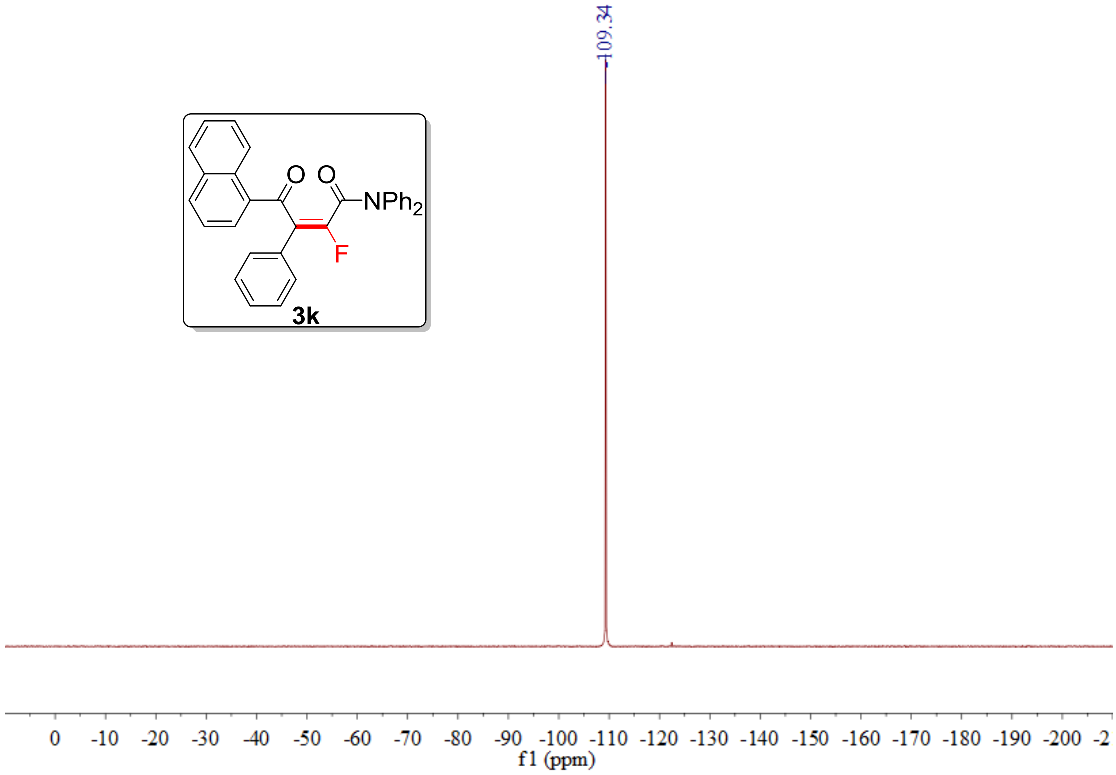
-109.34
O O
NPh$_2$
F
3k
0 -10 -20 -30 -40 -50 -60 -70 -80 -90 -100 -110 -120 -130 -140 -150 -160 -170 -180 -190 -200
f1 (ppm)

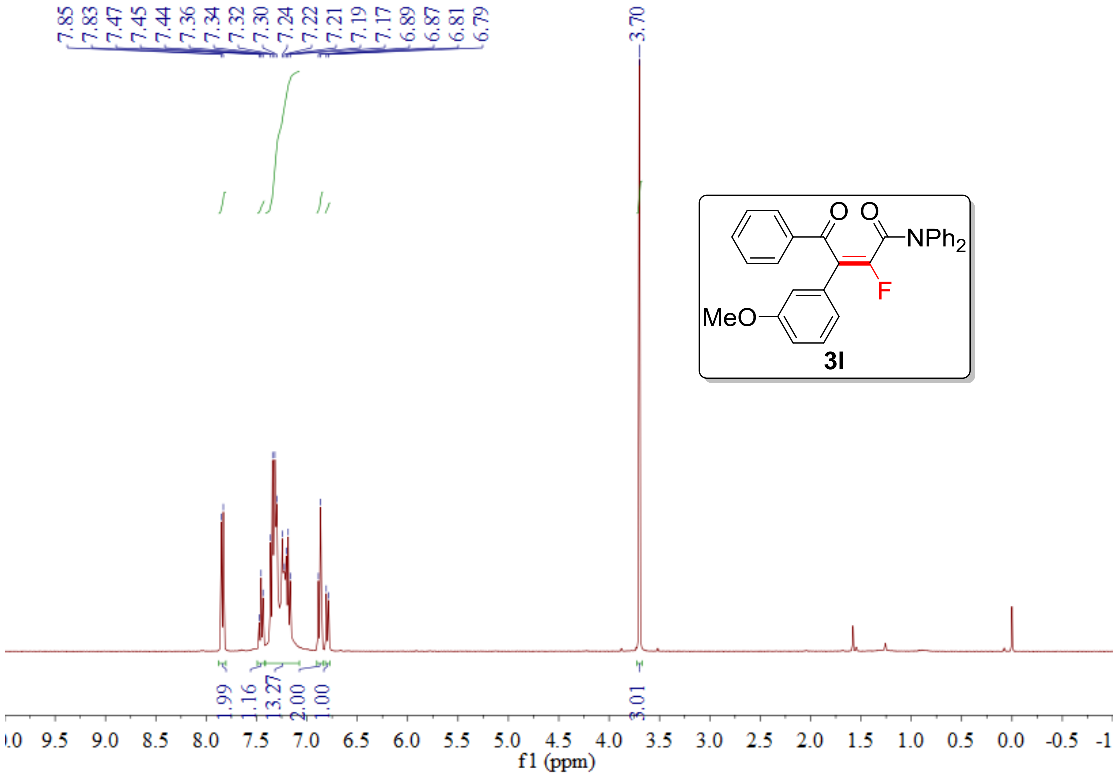
7.85 7.83 7.47 7.45 7.44 7.36 7.34 7.32 7.30 7.24 7.22 7.21 7.19 7.17 6.89 6.87 6.81 6.79
3.70
O O
NPh$_2$
F
MeO
3l
1.99 1.16 13.27 2.00 1.00
3.01
9.5 9.0 8.5 8.0 7.5 7.0 6.5 6.0 5.5 5.0 4.5 4.0 3.5 3.0 2.5 2.0 1.5 1.0 0.5 0.0 -0.5
f1 (ppm)

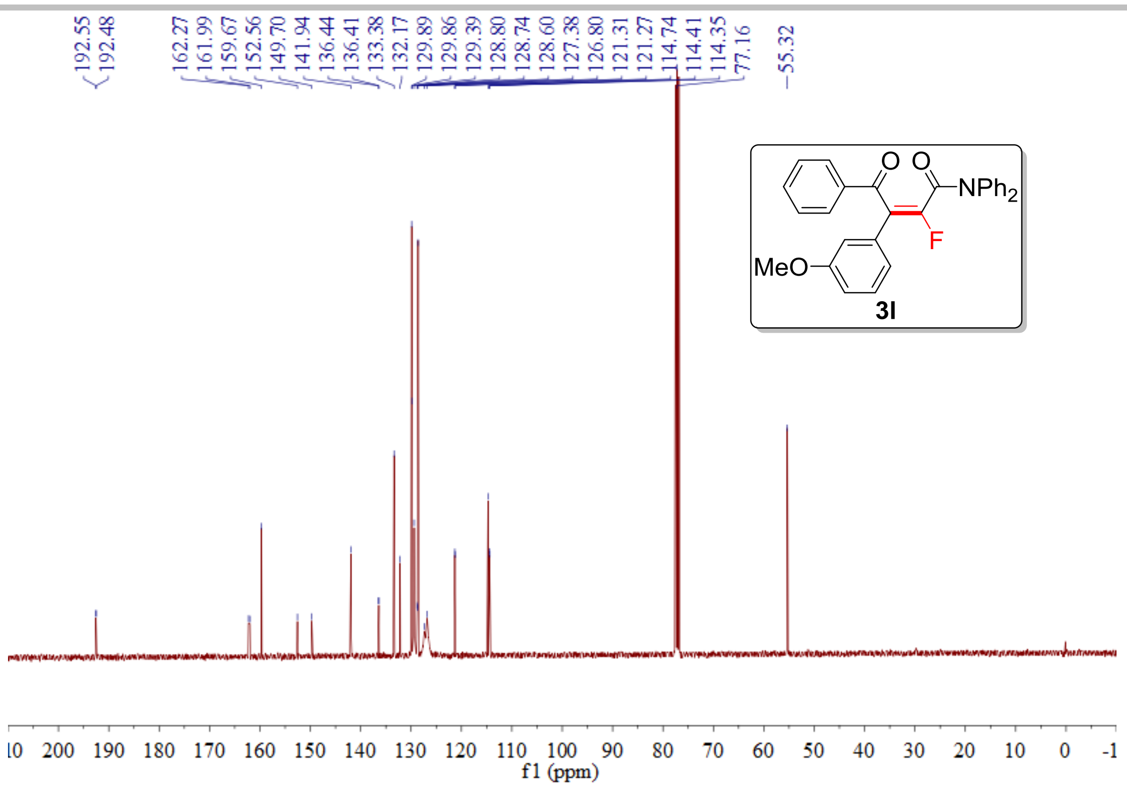
192.55
192.48
162.27
161.99
159.67
152.56
149.70
141.94
136.44
136.41
133.38
132.17
129.89
129.86
129.39
128.80
128.74
128.60
127.38
126.80
121.31
121.27
114.74
114.41
114.35
77.16
55.32
O
O
NPh2
F
MeO
3l
f1 (ppm)

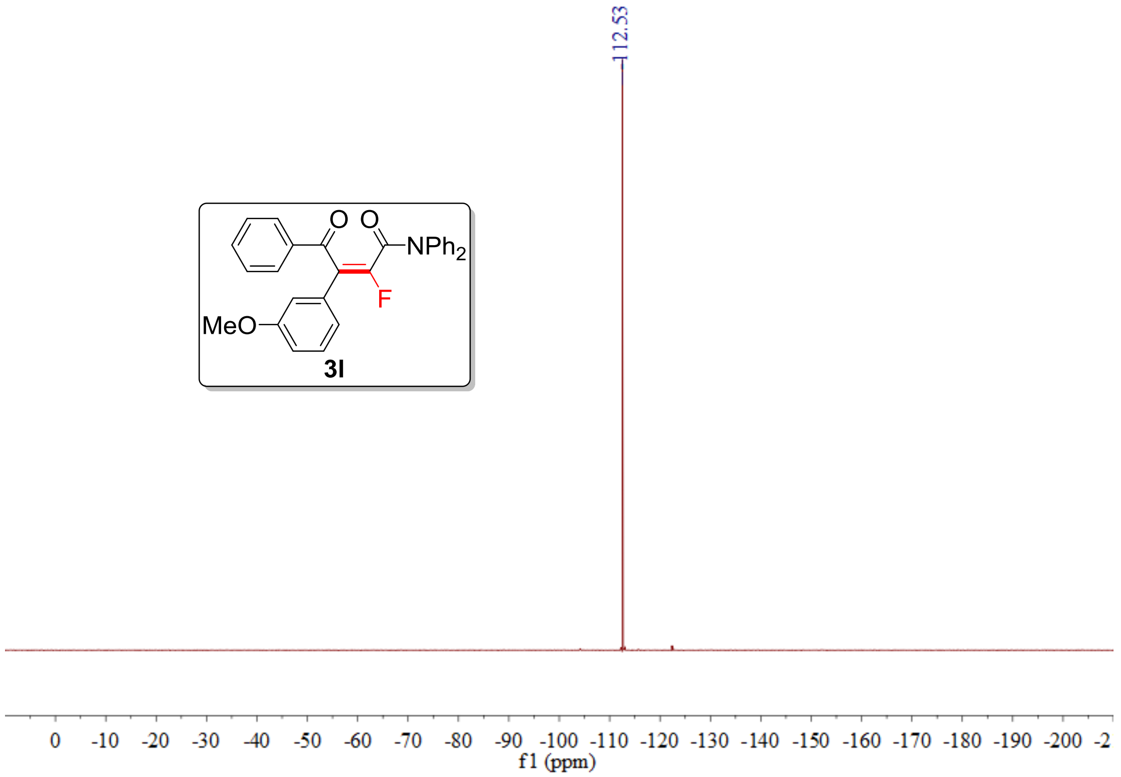
-112.53
O
O
NPh2
F
MeO
3l
f1 (ppm)

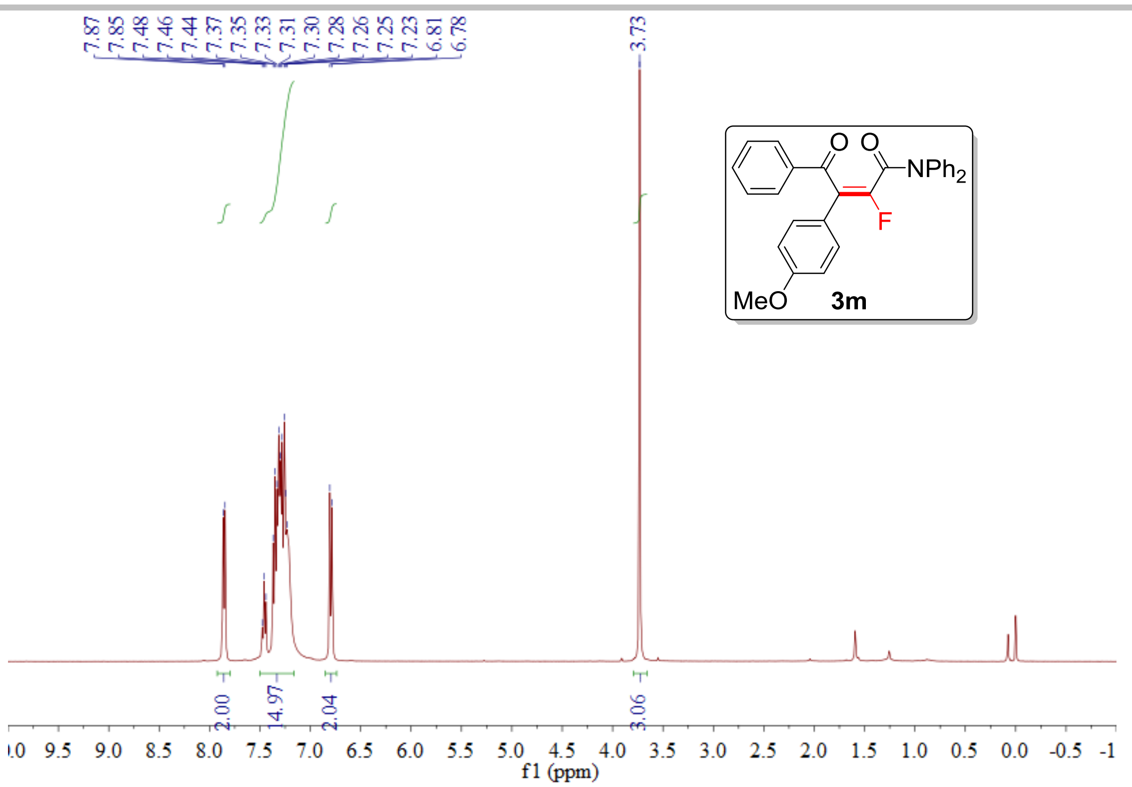
7.87
7.85
7.48
7.46
7.44
7.37
7.35
7.33
7.31
7.30
7.28
7.26
7.25
7.23
6.81
6.78
3.73
2.00
14.97
2.04
3.06
f1 (ppm)
O
O
NPh$_2$
F
MeO
3m

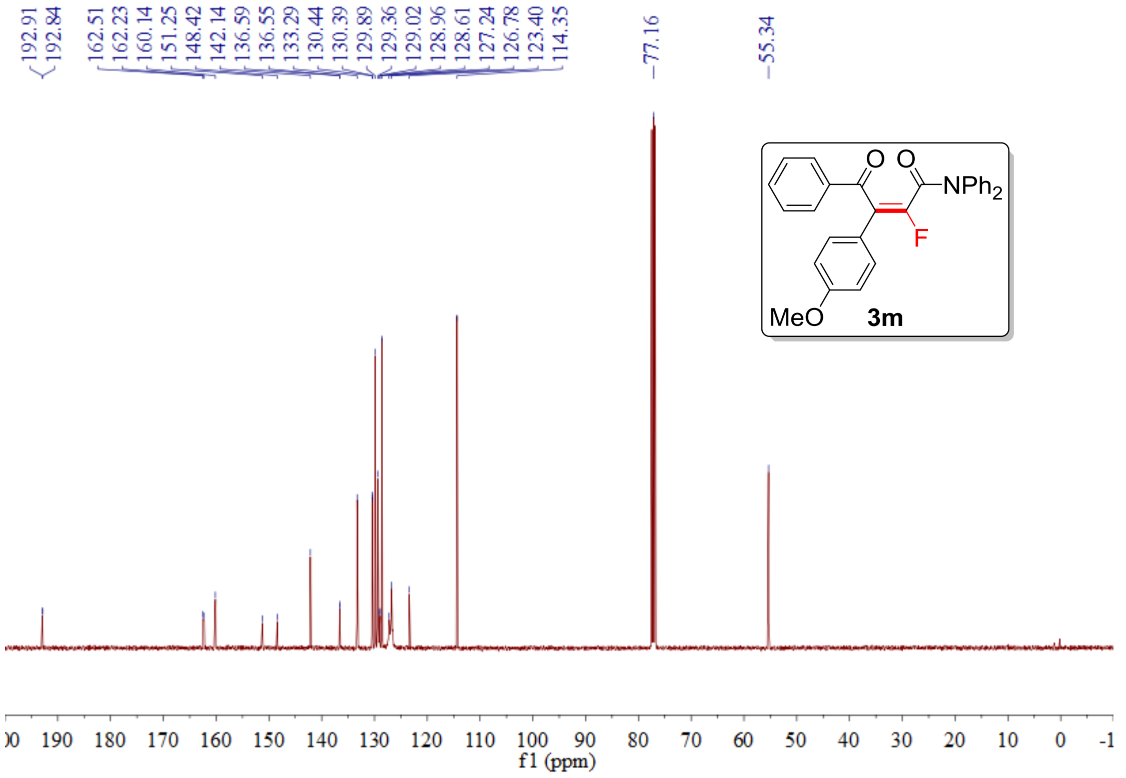
192.91
192.84
162.51
162.23
160.14
151.25
148.42
142.14
136.59
136.55
133.29
130.44
130.39
129.89
129.36
129.02
128.96
128.61
127.24
126.78
123.40
114.35
77.16
55.34
f1 (ppm)
O
O
NPh$_2$
F
MeO
3m

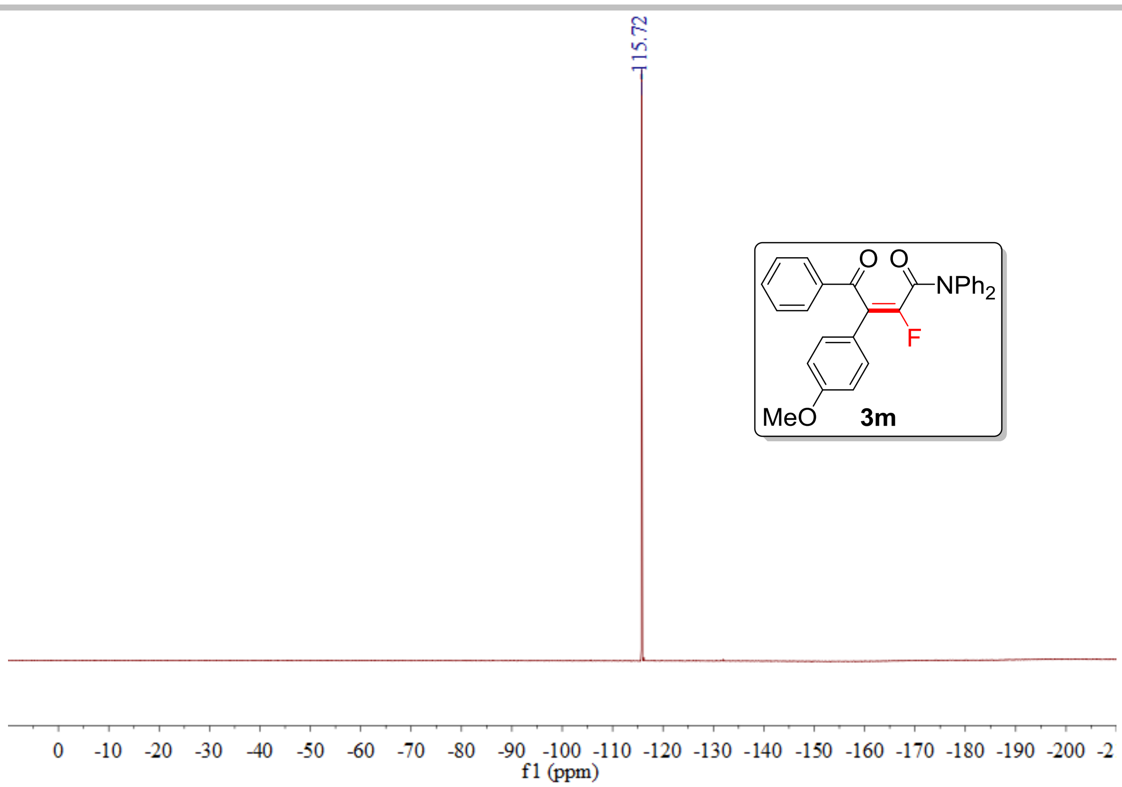
-115.72
NPh2
F
MeO
3m
f1 (ppm)

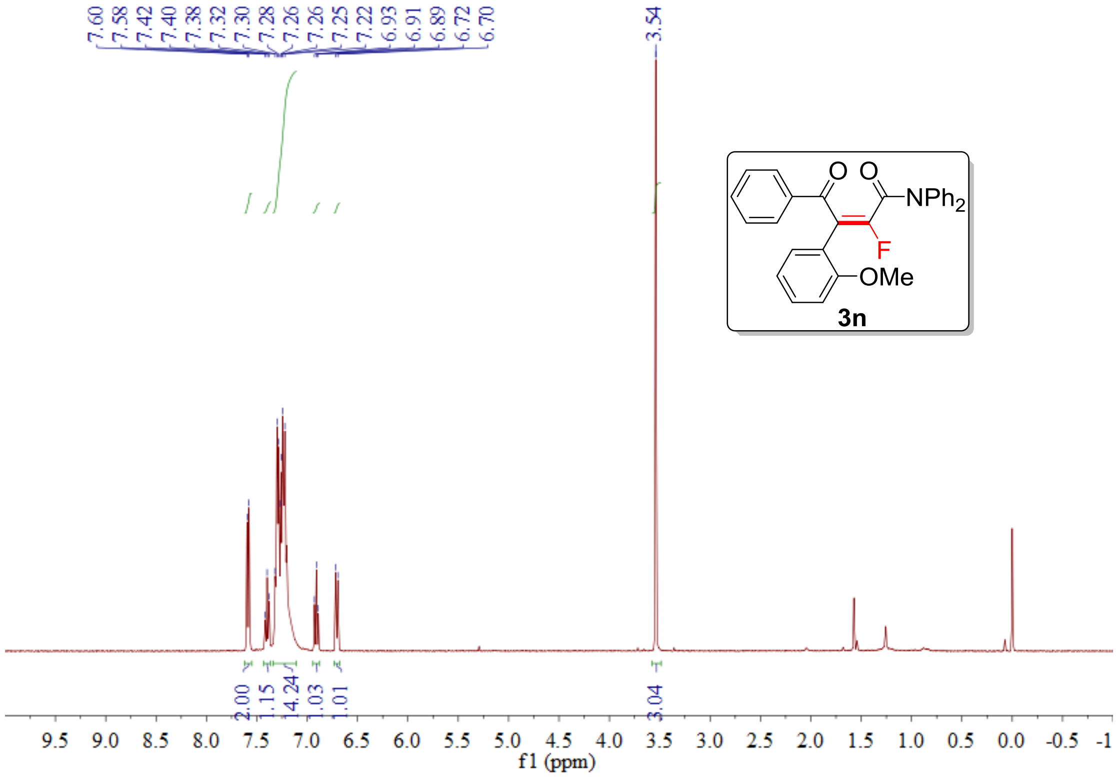
7.60 7.58 7.42 7.40 7.38 7.32 7.30 7.28 7.26 7.26 7.25 7.22 6.93 6.91 6.89 6.72 6.70
3.54
NPh2
F
OMe
3n
2.00 1.15 14.24 1.03 1.01
3.04
f1 (ppm)

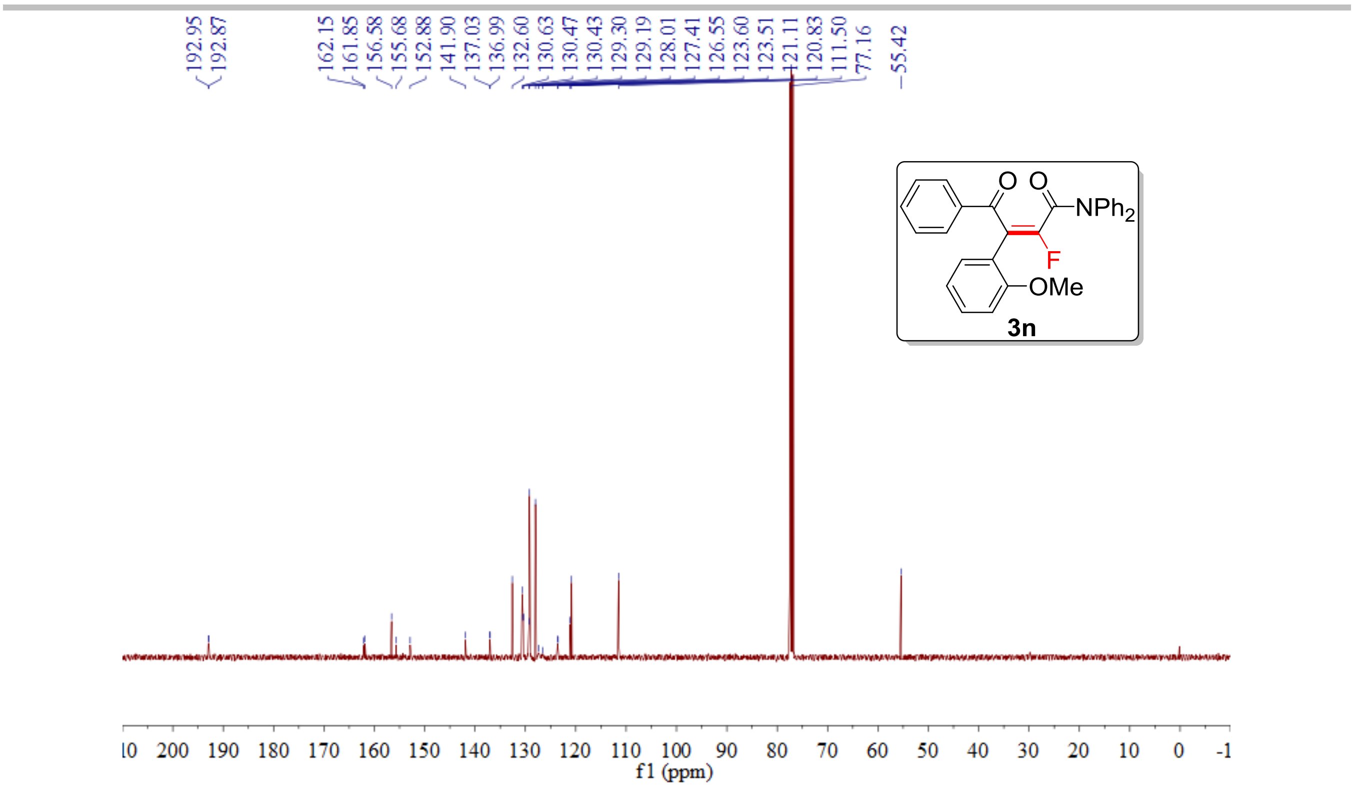
192.95
192.87
162.15
161.85
156.58
155.68
152.88
141.90
137.03
136.99
132.60
130.63
130.47
130.43
129.30
129.19
128.01
127.41
126.55
123.60
123.51
121.11
120.83
111.50
77.16
55.42
O
O
NPh2
F
OMe
3n
f1 (ppm)

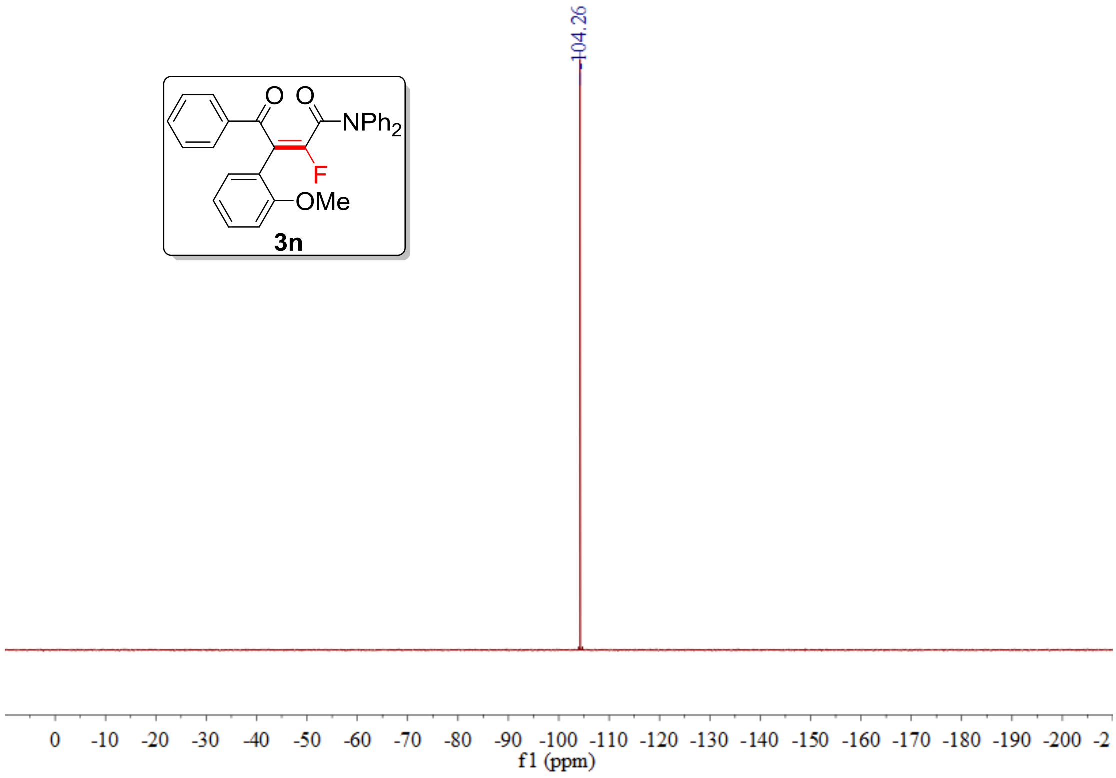
-104.26
O
O
NPh2
F
OMe
3n
f1 (ppm)

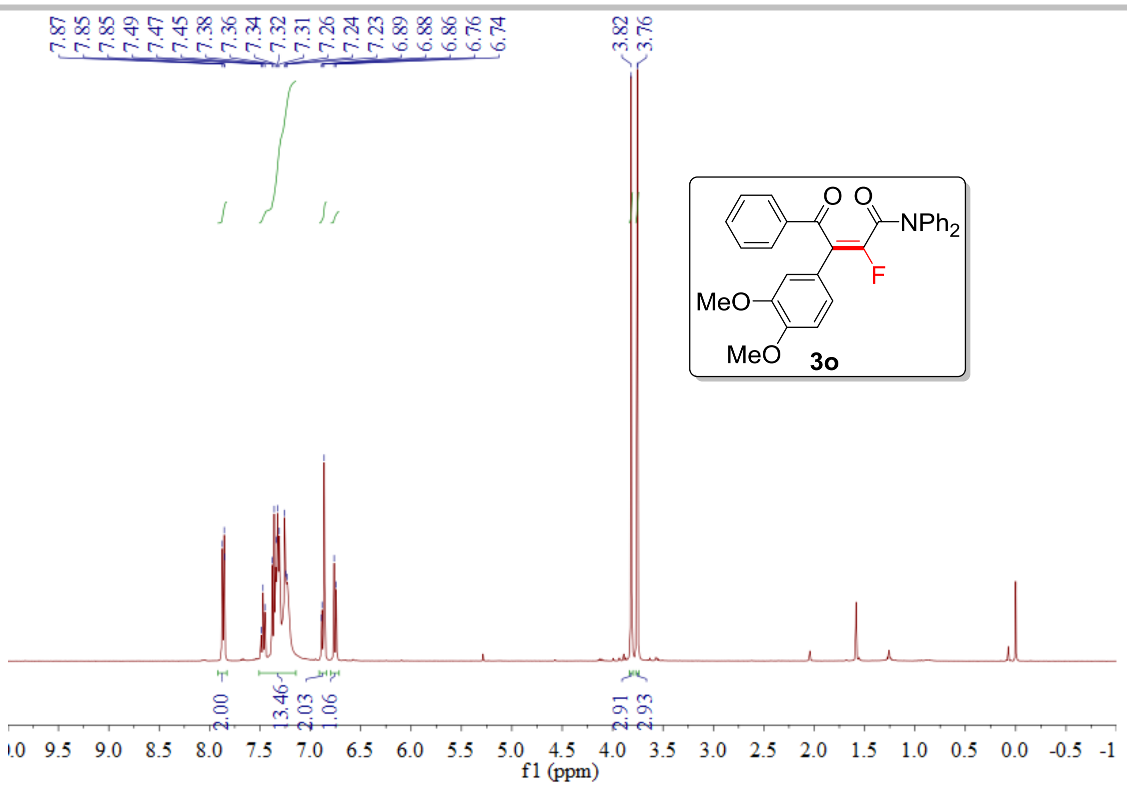
7.87 7.85 7.85 7.49 7.47 7.45 7.38 7.36 7.34 7.32 7.31 7.26 7.24 7.23 6.89 6.88 6.86 6.76 6.74
3.82 3.76
2.00 13.46 2.03 1.06 2.91 2.93
f1 (ppm)
MeO
MeO
NPh2
F
3o

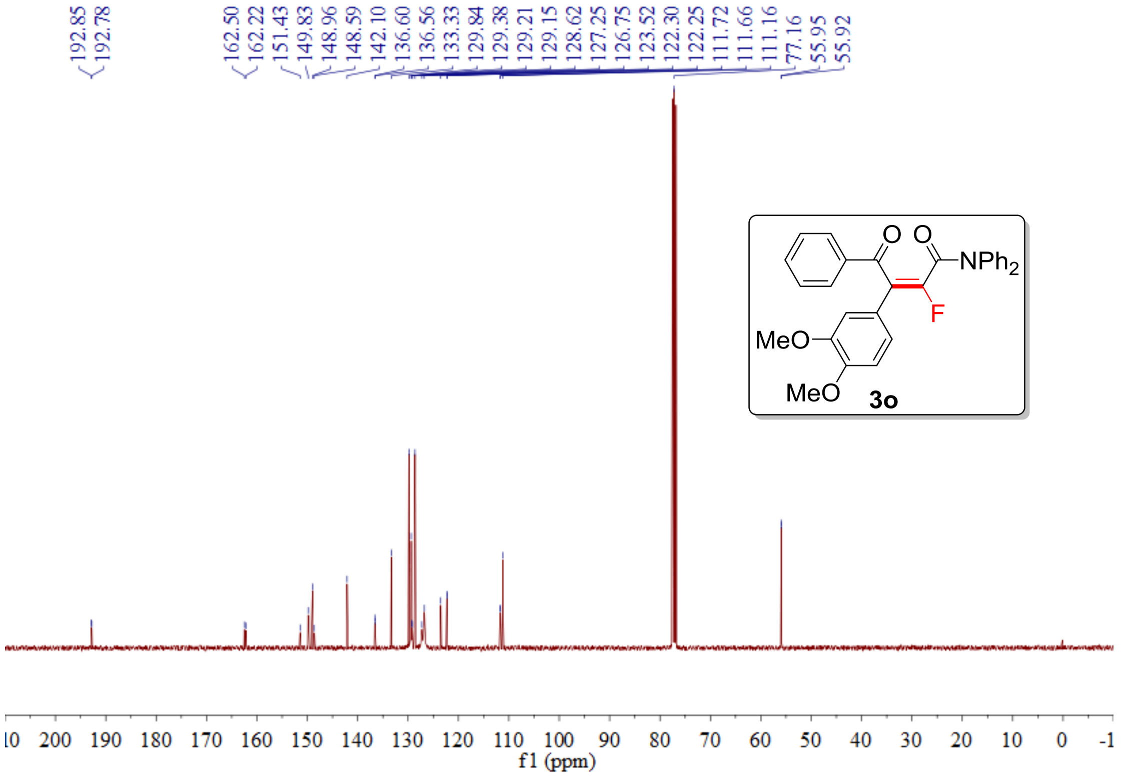
192.85 192.78
162.50 162.22 151.43 149.83 148.96 148.59 142.10 136.60 136.56 133.33 129.84 129.38 129.21 129.15 128.62 127.25 126.75 123.52 122.30 122.25 111.72 111.66 111.16 77.16 55.95 55.92
f1 (ppm)
MeO
MeO
NPh2
F
3o

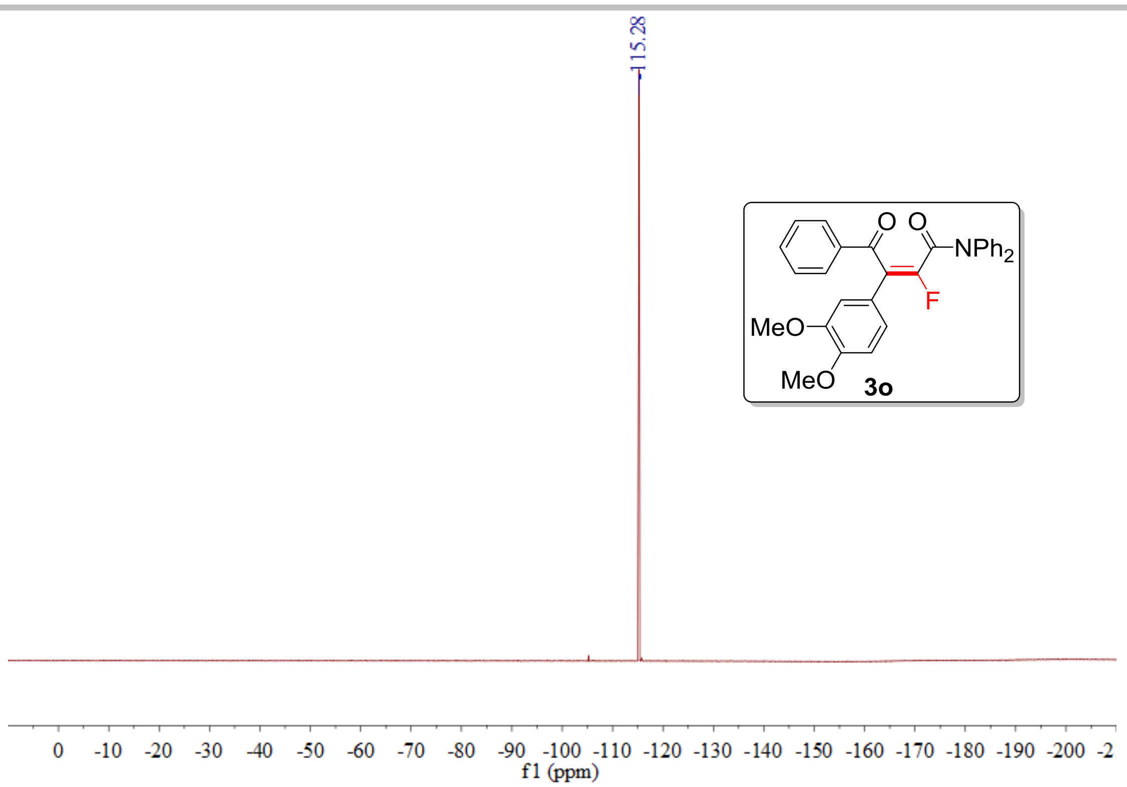

-115.28
O
O
NPh2
F
MeO
MeO
3o
0
-10
-20
-30
-40
-50
-60
-70
-80
-90
-100
-110
-120
-130
-140
-150
-160
-170
-180
-190
-200
f1 (ppm)

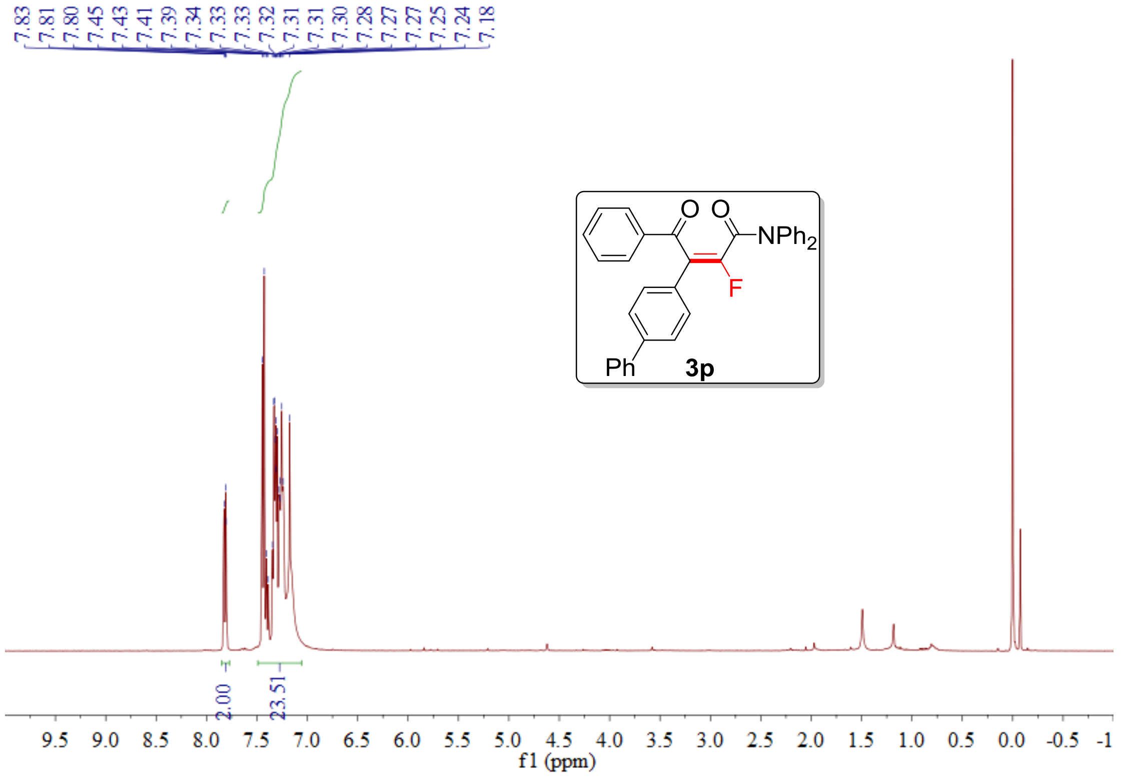

7.83
7.81
7.80
7.45
7.43
7.41
7.39
7.34
7.33
7.33
7.32
7.31
7.31
7.30
7.28
7.27
7.27
7.25
7.24
7.18
O
O
NPh2
F
Ph
3p
2.00
23.51
9.5
9.0
8.5
8.0
7.5
7.0
6.5
6.0
5.5
5.0
4.5
4.0
3.5
3.0
2.5
2.0
1.5
1.0
0.5
0.0
-0.5
-1
f1 (ppm)

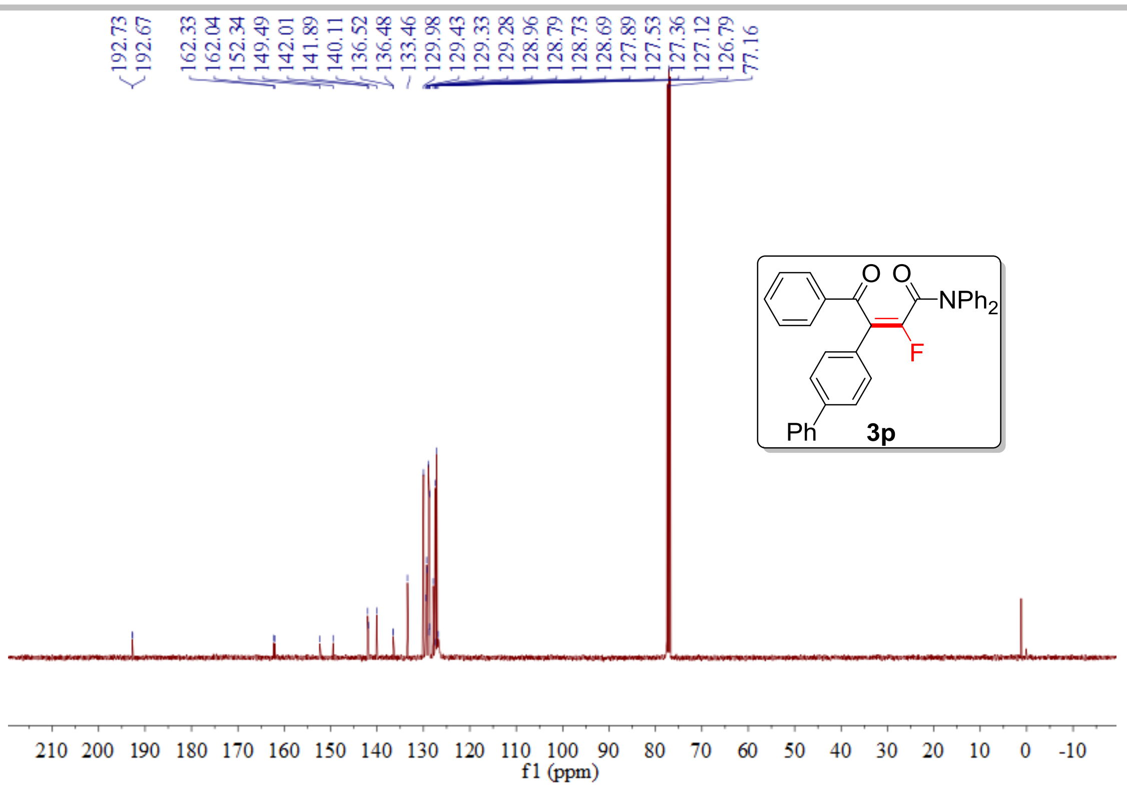
192.73
192.67
162.33
162.04
152.34
149.49
142.01
141.89
140.11
136.52
136.48
133.46
129.98
129.43
129.33
129.28
128.96
128.79
128.73
128.69
127.89
127.53
127.36
127.12
126.79
77.16
O
O
NPh2
F
Ph
3p
210 200 190 180 170 160 150 140 130 120 110 100 90 80 70 60 50 40 30 20 10 0 -10
f1 (ppm)

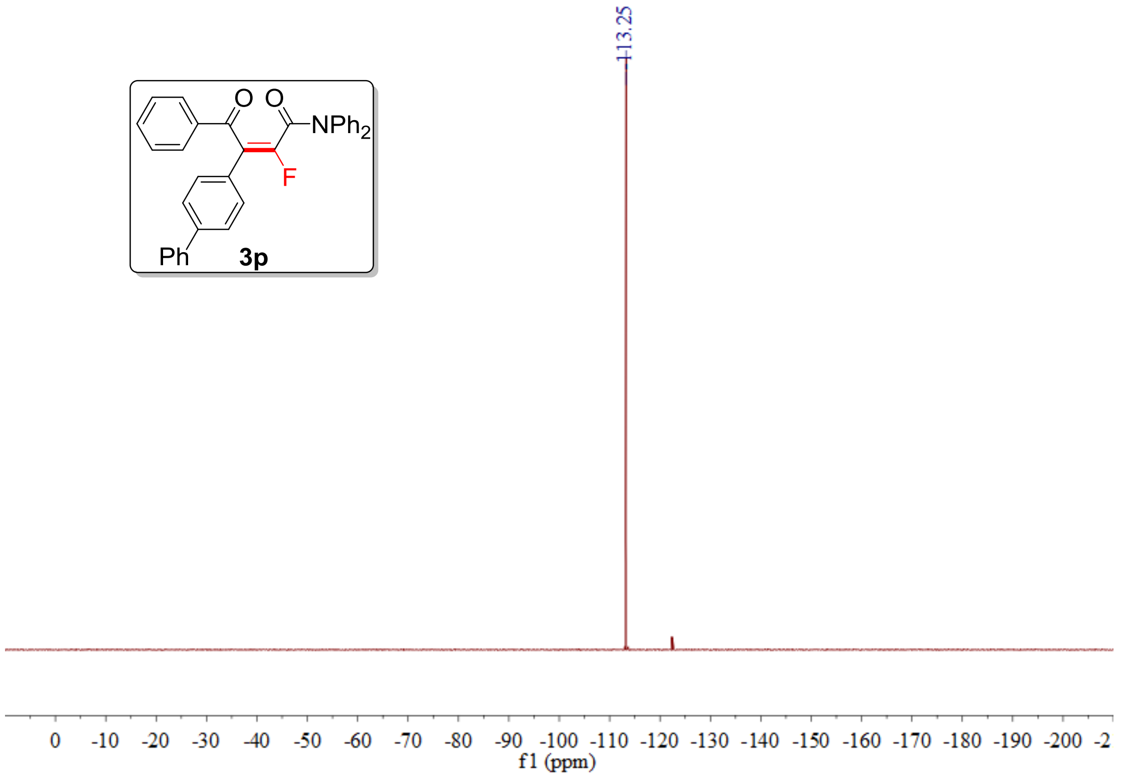
-113.25
O
O
NPh2
F
Ph
3p
0 -10 -20 -30 -40 -50 -60 -70 -80 -90 -100 -110 -120 -130 -140 -150 -160 -170 -180 -190 -200 -2
f1 (ppm)

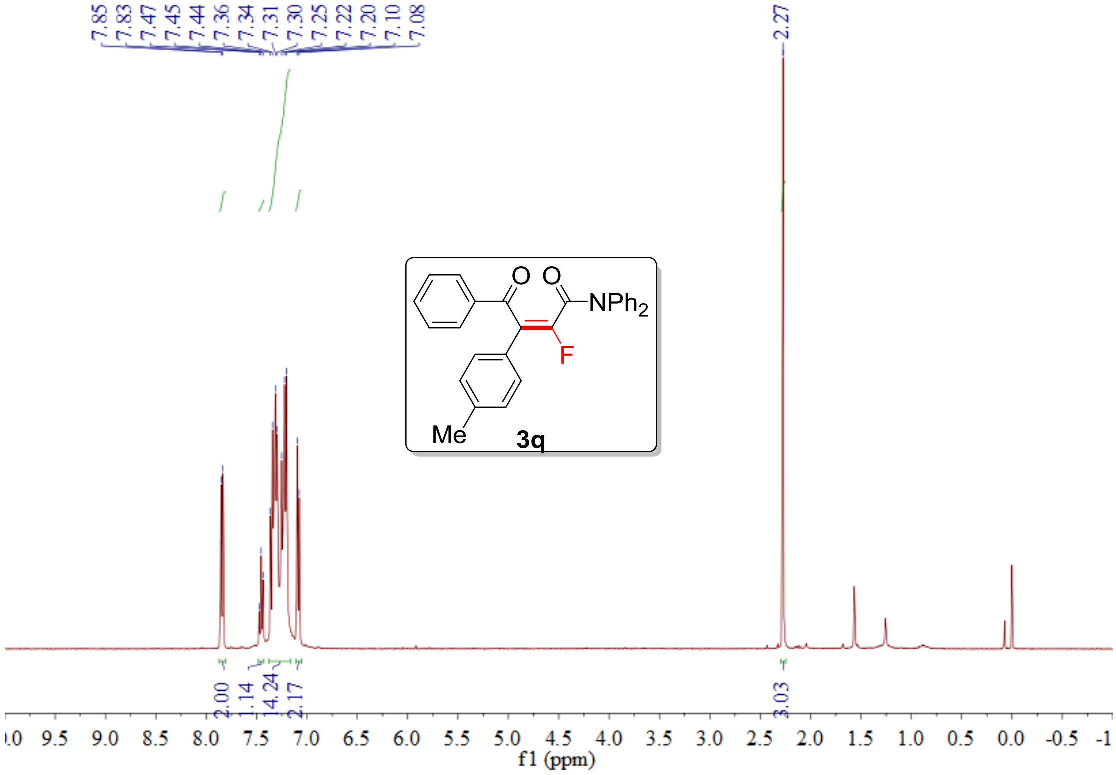

7.85
7.83
7.47
7.45
7.44
7.36
7.34
7.31
7.30
7.25
7.22
7.20
7.10
7.08
2.27
NPh2
F
Me
3q
2.00
1.14
14.24
2.17
3.03
f1 (ppm)

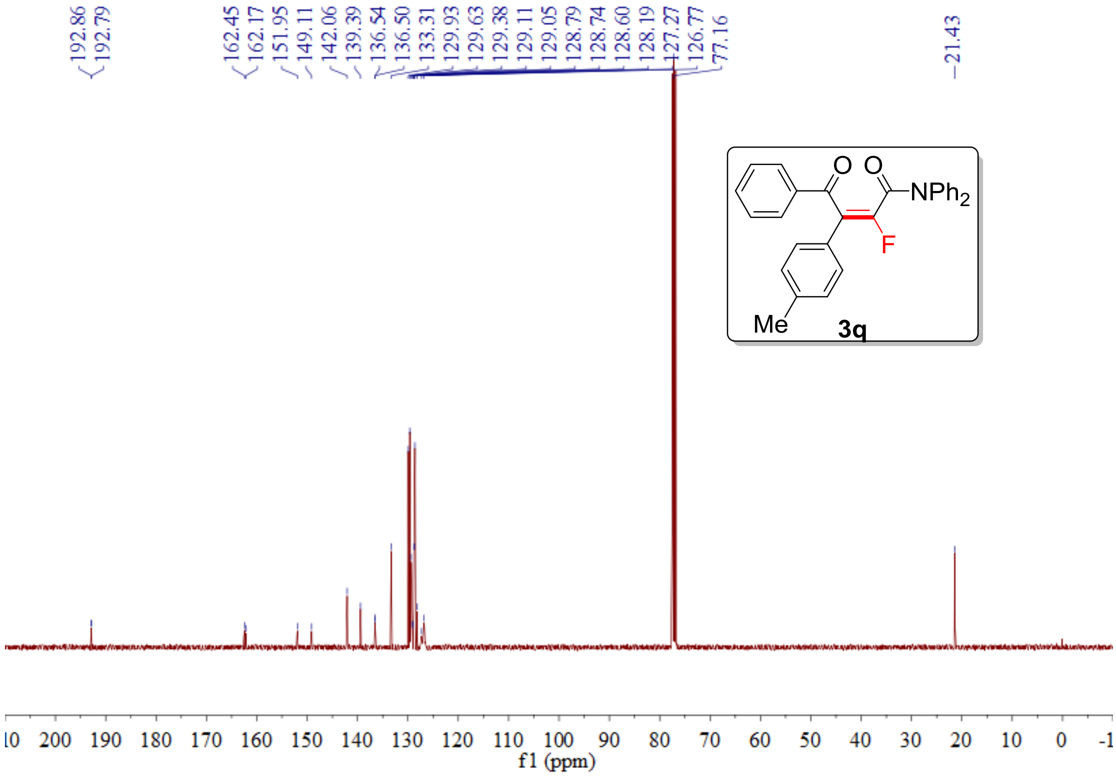

192.86
192.79
162.45
162.17
151.95
149.11
142.06
139.39
136.54
136.50
133.31
129.93
129.63
129.38
129.11
129.05
128.79
128.74
128.60
128.19
127.27
126.77
77.16
21.43
NPh2
F
Me
3q
f1 (ppm)

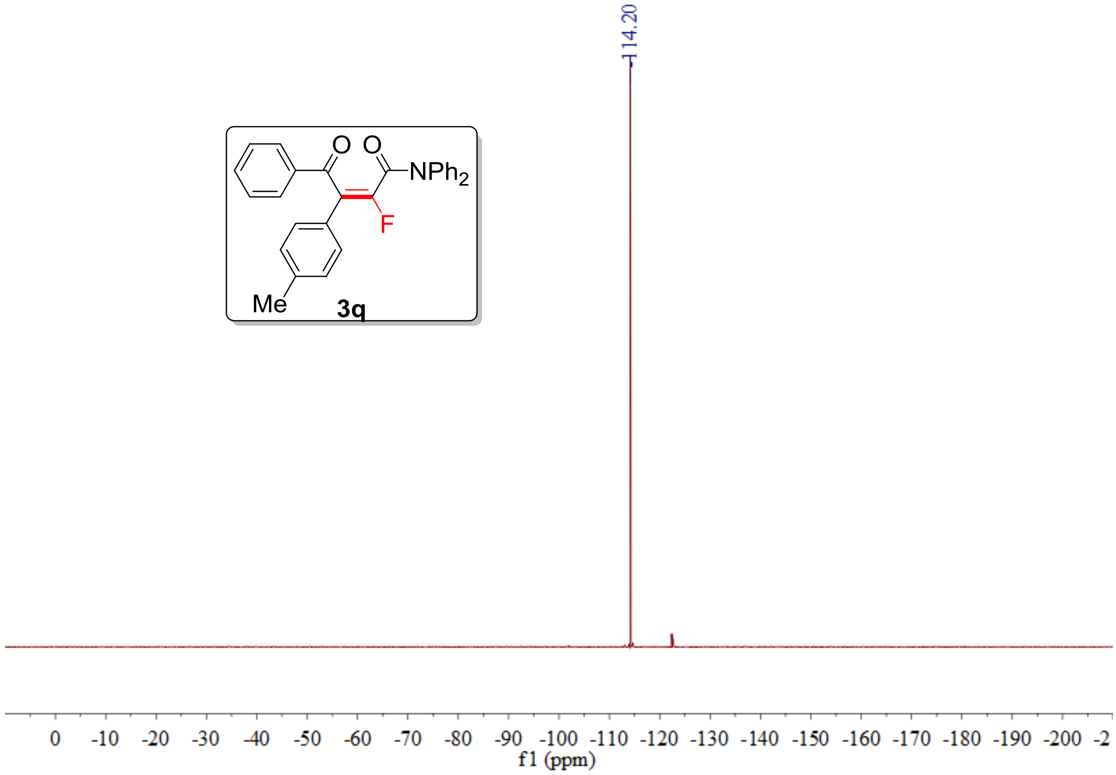
-114.20
NPh2
F
Me
3q
f1 (ppm)

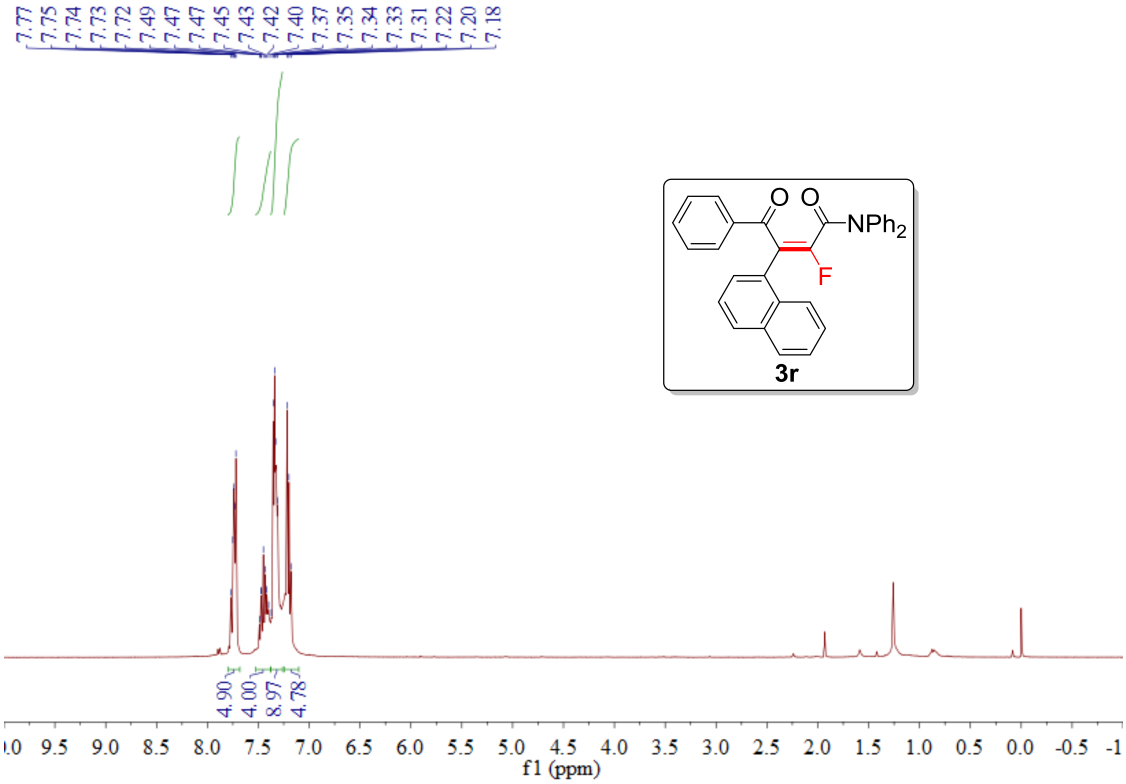
7.77 7.75 7.74 7.73 7.72 7.49 7.47 7.47 7.45 7.43 7.42 7.40 7.37 7.35 7.34 7.33 7.31 7.22 7.20 7.18
NPh2
F
3r
4.90 4.00 8.97 4.78
f1 (ppm)

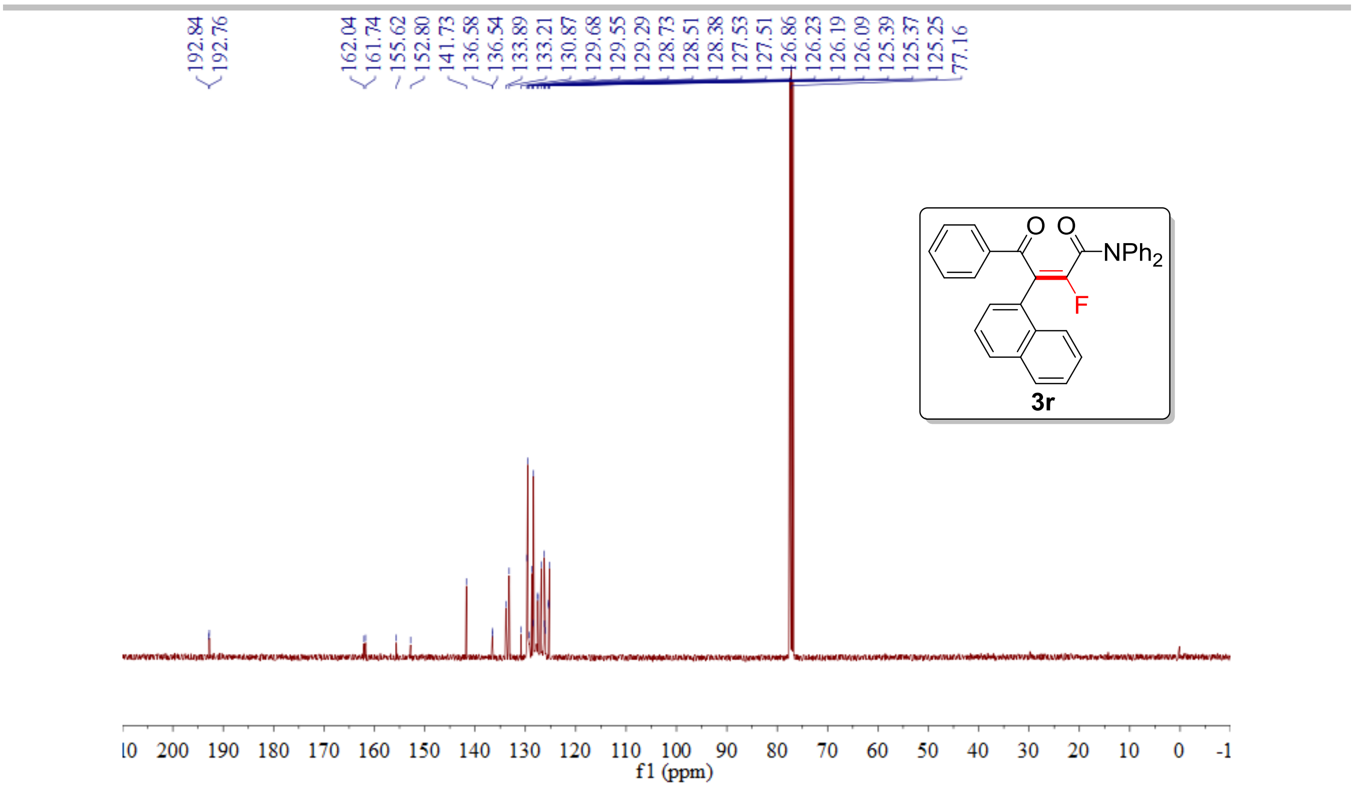
192.84
192.76
162.04
161.74
155.62
152.80
141.73
136.58
136.54
133.89
133.21
130.87
129.68
129.55
129.29
128.73
128.51
128.38
127.53
127.51
126.86
126.23
126.19
126.09
125.39
125.37
125.25
77.16
O O
NPh2
F
3r
f1 (ppm)

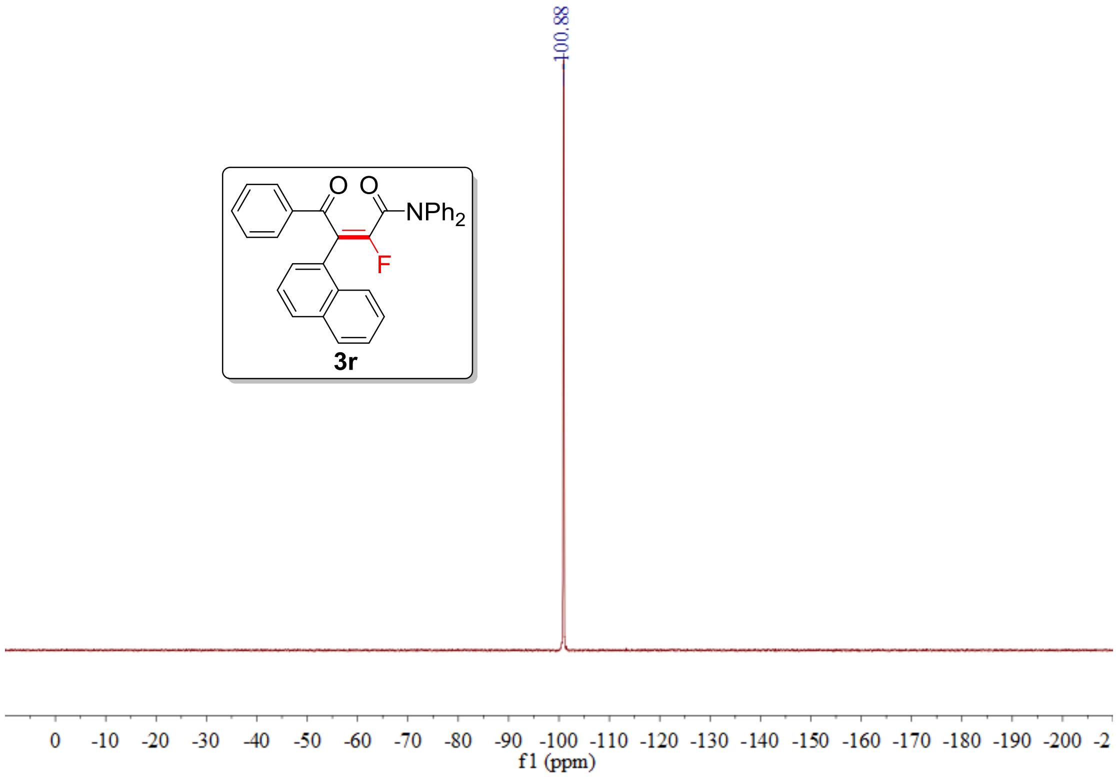
-100.88
O O
NPh2
F
3r
f1 (ppm)

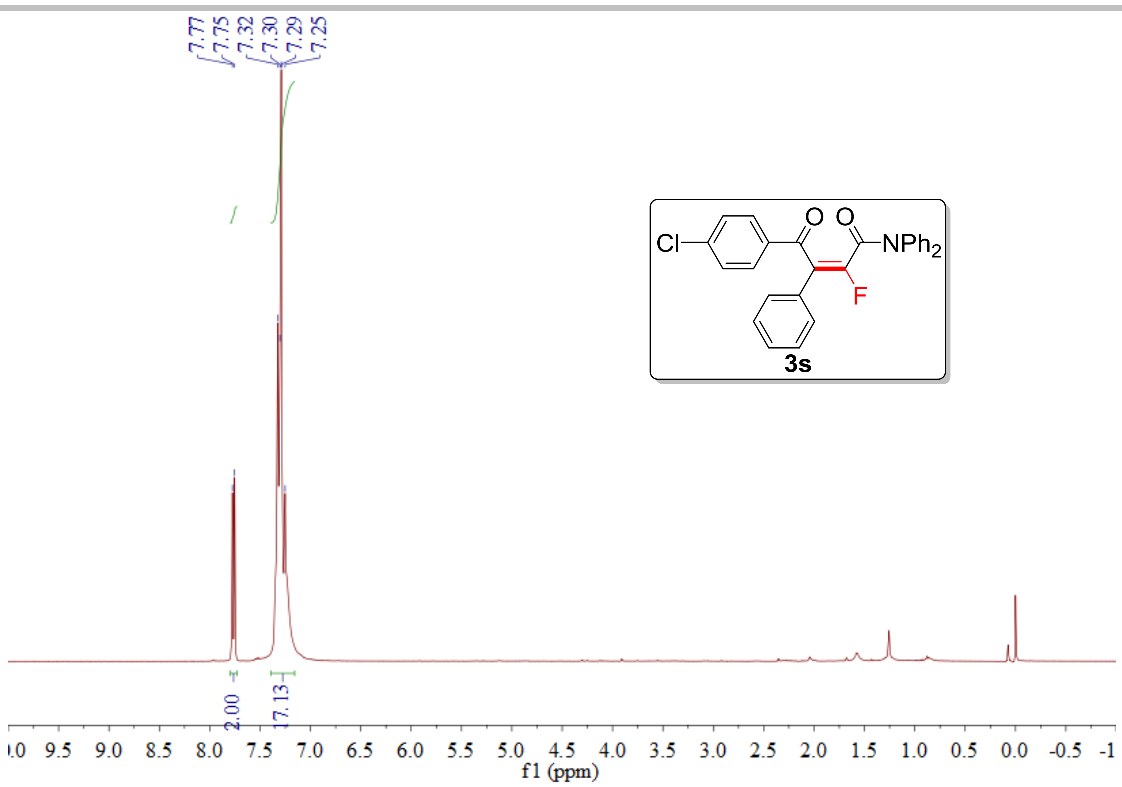
7.77
7.75
7.32
7.30
7.29
7.25
2.00
17.13
f1 (ppm)
3s

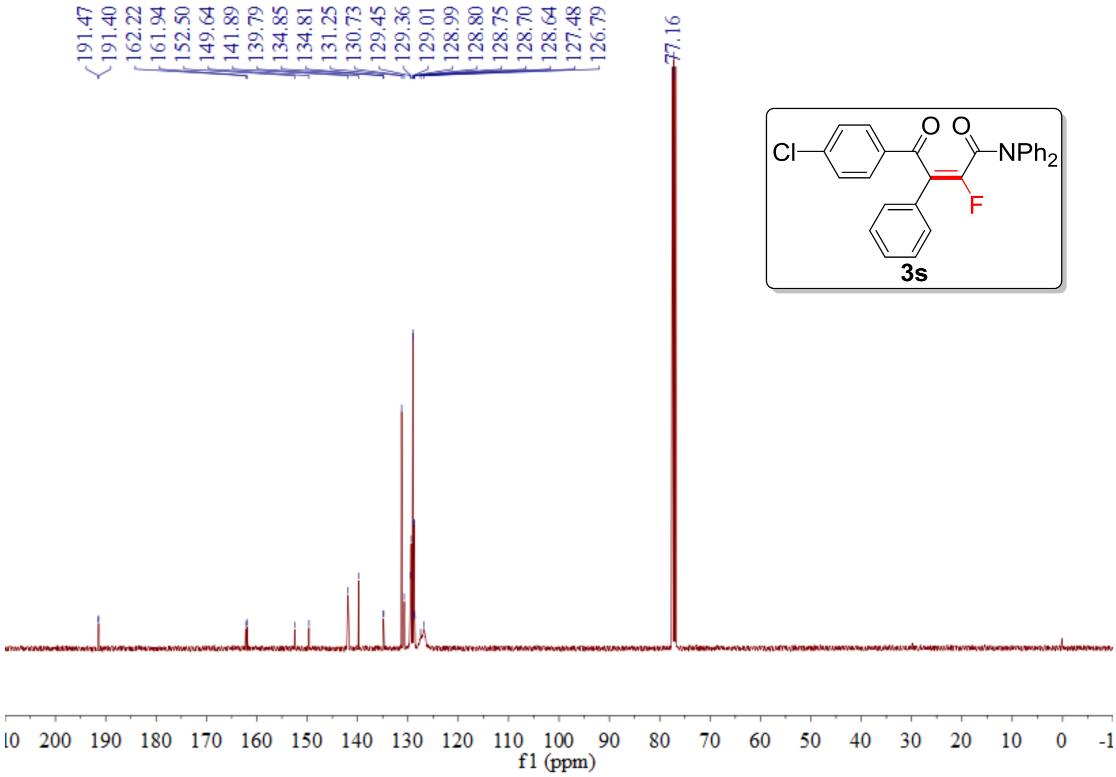
191.47
191.40
162.22
161.94
152.50
149.64
141.89
139.79
134.85
134.81
131.25
130.73
129.45
129.36
129.01
128.99
128.80
128.75
128.70
128.64
127.48
126.79
77.16
f1 (ppm)
3s

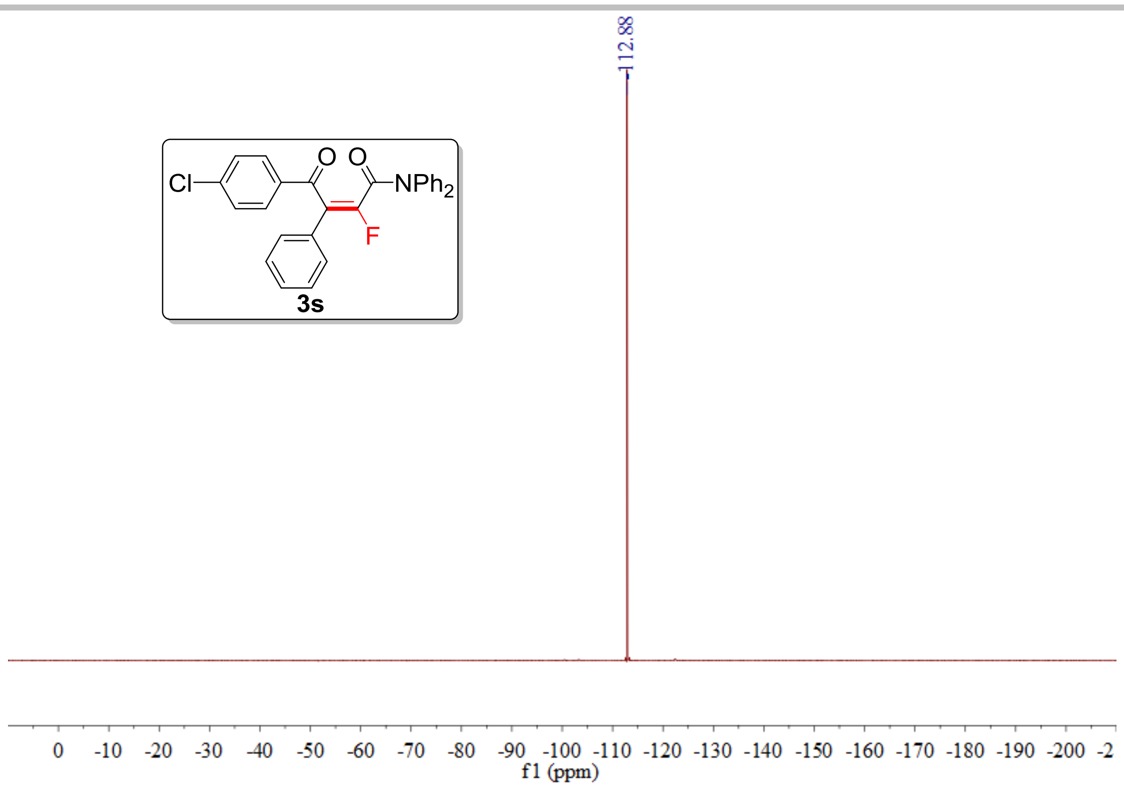

-112.88
Cl
O
O
NPh2
F
3s
0 -10 -20 -30 -40 -50 -60 -70 -80 -90 -100 -110 -120 -130 -140 -150 -160 -170 -180 -190 -200 -2
f1 (ppm)

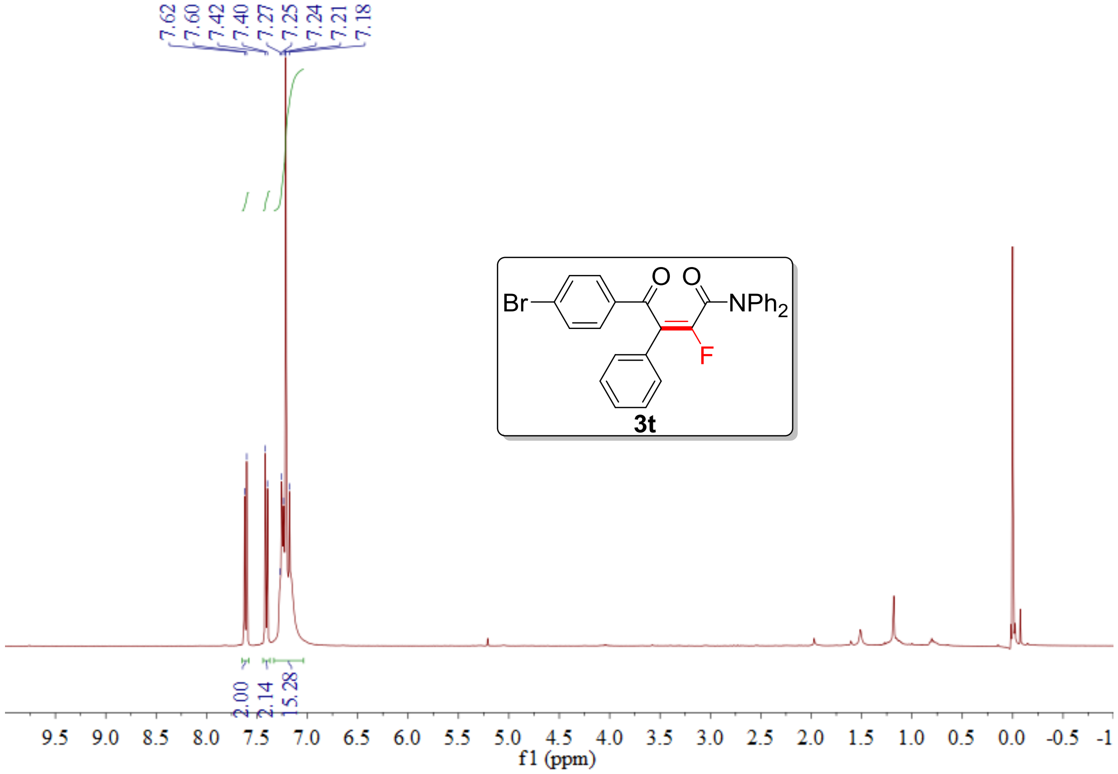

7.62
7.60
7.42
7.40
7.27
7.25
7.24
7.21
7.18
Br
O
O
NPh2
F
3t
2.00
2.14
15.28
9.5 9.0 8.5 8.0 7.5 7.0 6.5 6.0 5.5 5.0 4.5 4.0 3.5 3.0 2.5 2.0 1.5 1.0 0.5 0.0 -0.5 -1
f1 (ppm)

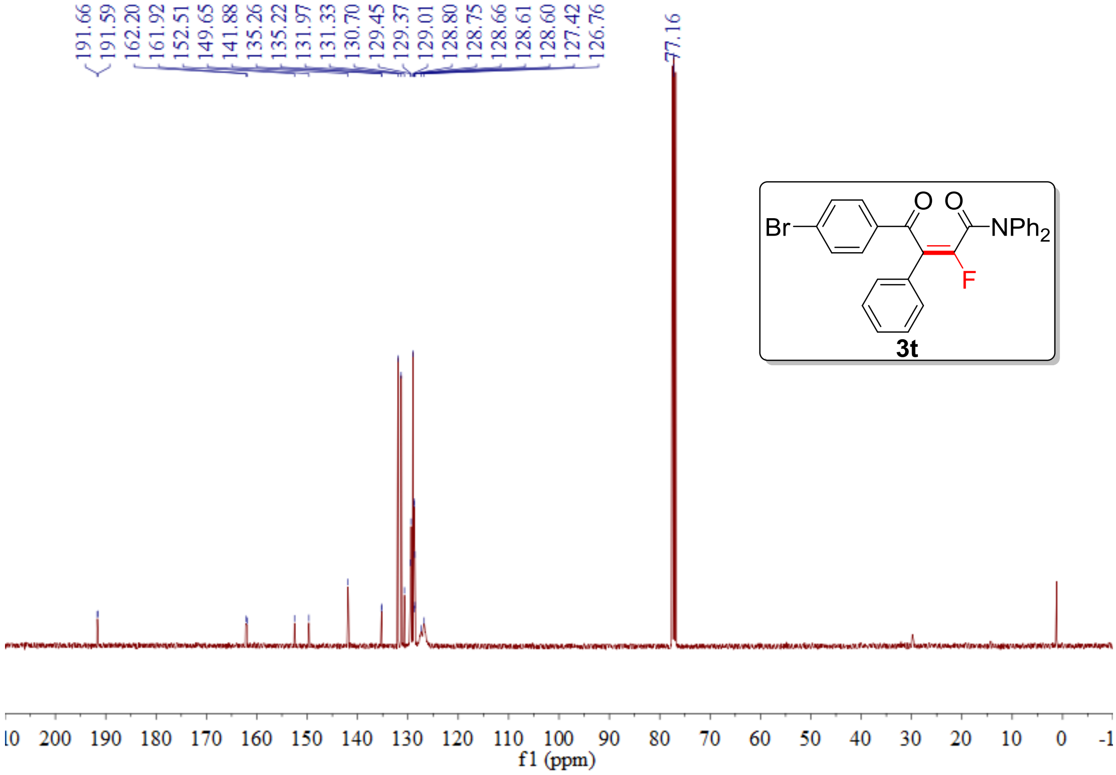
191.66
191.59
162.20
161.92
152.51
149.65
141.88
135.26
135.22
131.97
131.33
130.70
129.45
129.37
129.01
128.80
128.75
128.66
128.61
128.60
127.42
126.76
77.16
Br
O O
NPh2
F
3t
200 190 180 170 160 150 140 130 120 110 100 90 80 70 60 50 40 30 20 10 0
f1 (ppm)

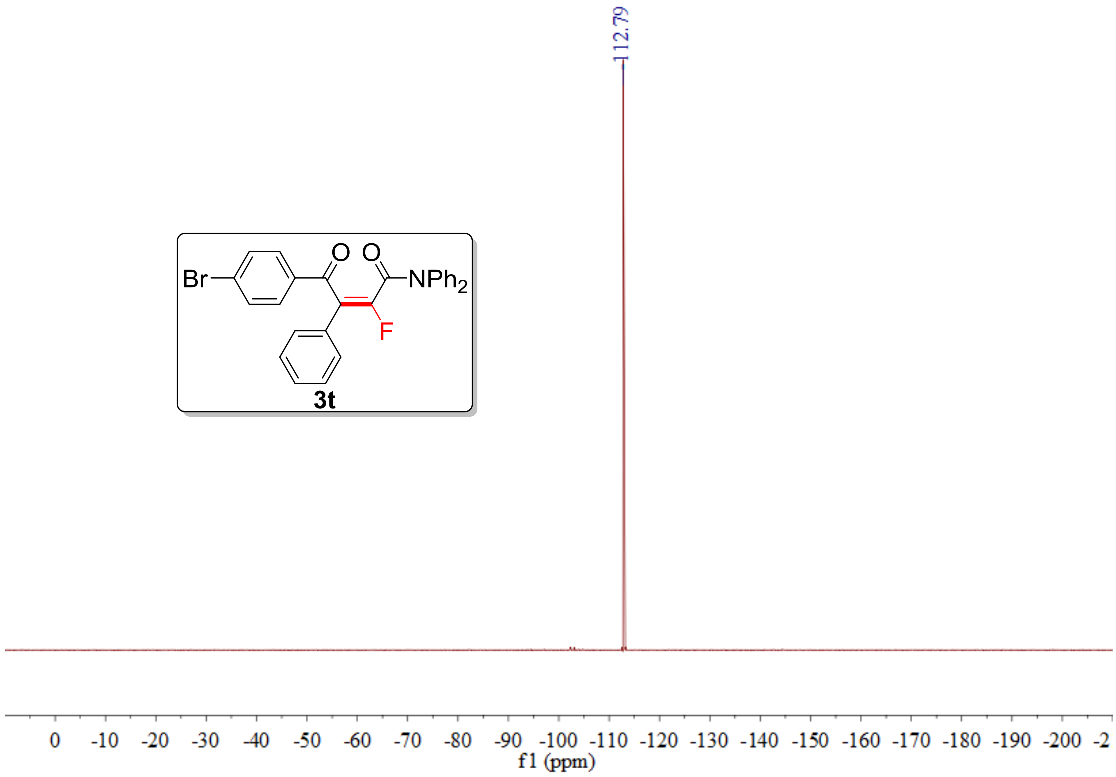
-112.79
Br
O O
NPh2
F
3t
0 -10 -20 -30 -40 -50 -60 -70 -80 -90 -100 -110 -120 -130 -140 -150 -160 -170 -180 -190 -200
f1 (ppm)

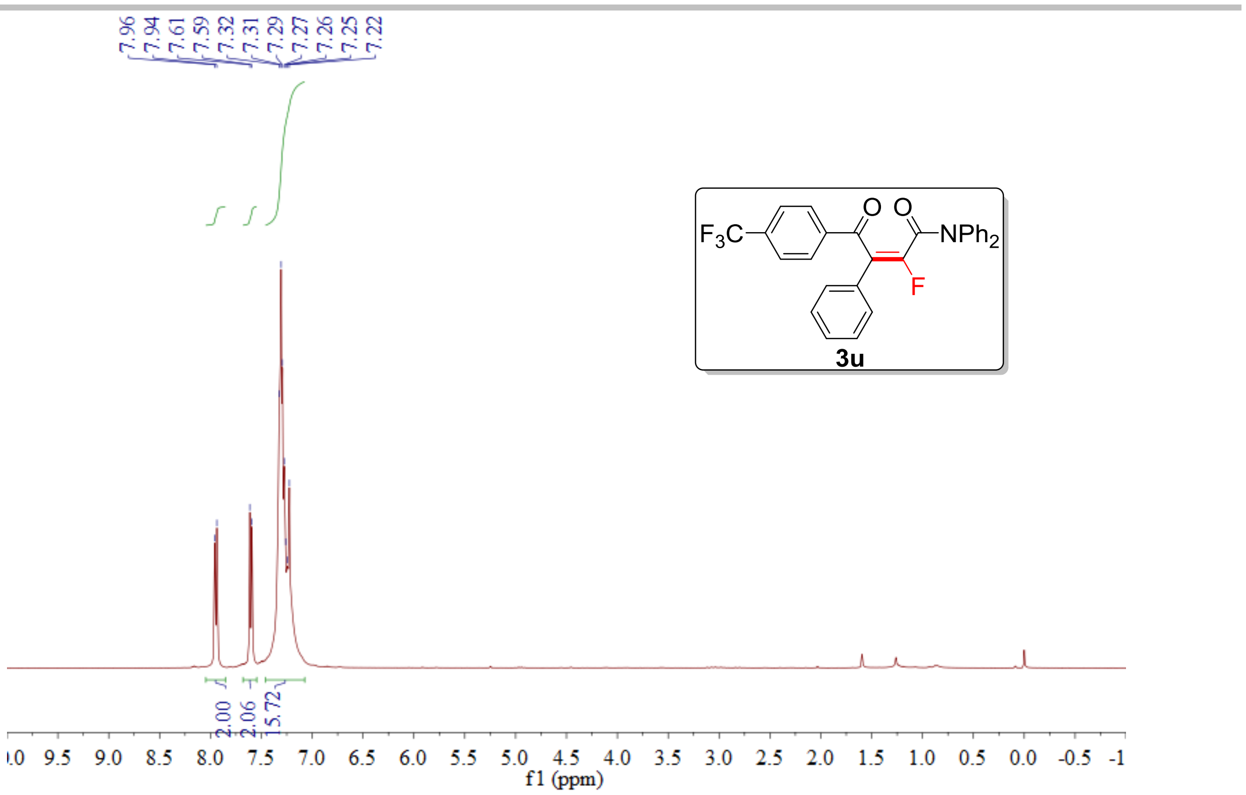
7.96
7.94
7.61
7.59
7.32
7.31
7.29
7.27
7.26
7.25
7.22
F3C
O O
NPh2
F
3u
2.00
2.06
15.72
f1 (ppm)

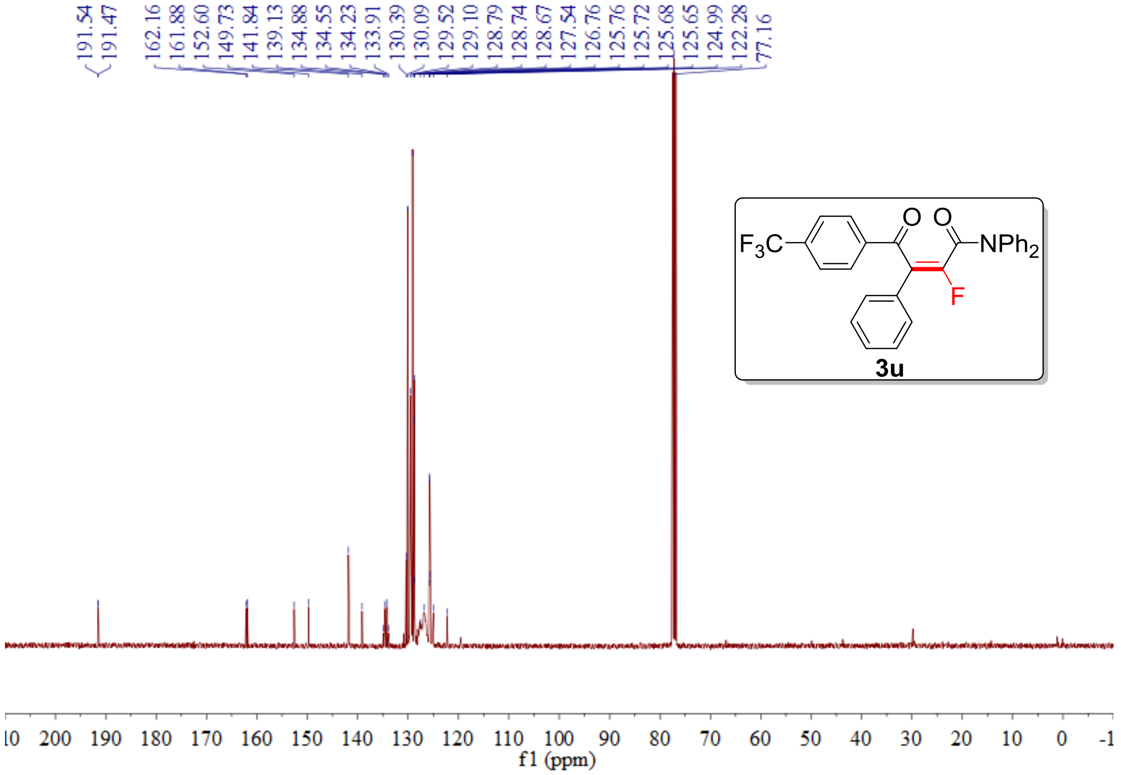
191.54
191.47
162.16
161.88
152.60
149.73
141.84
139.13
134.88
134.55
134.23
133.91
130.39
130.09
129.52
129.10
128.79
128.74
128.67
127.54
126.76
125.76
125.72
125.68
125.65
124.99
122.28
77.16
F3C
O O
NPh2
F
3u
f1 (ppm)

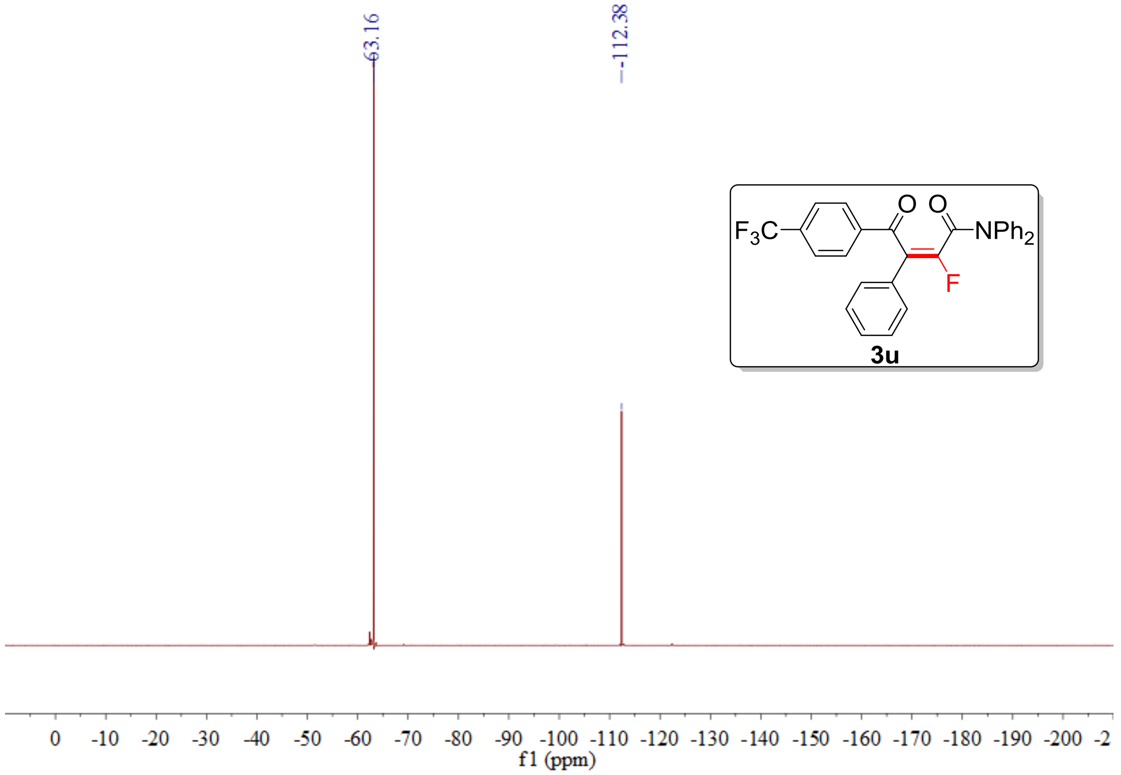

-63.16
-112.38
F_3C
O
O
NPh_2
F
3u
0
-10
-20
-30
-40
-50
-60
-70
-80
-90
-100
-110
-120
-130
-140
-150
-160
-170
-180
-190
-200
f1 (ppm)

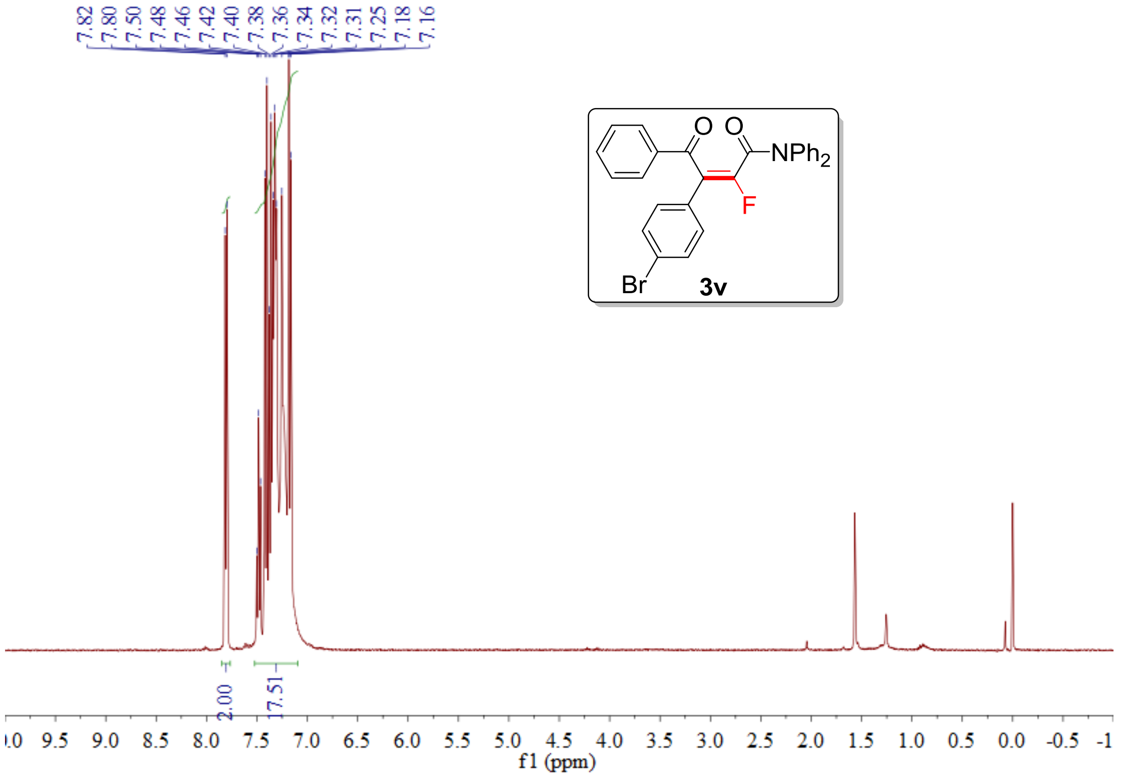

7.82
7.80
7.50
7.48
7.46
7.42
7.40
7.38
7.36
7.34
7.32
7.31
7.25
7.18
7.16
O
O
NPh_2
F
Br
3v
2.00
17.51
9.5
9.0
8.5
8.0
7.5
7.0
6.5
6.0
5.5
5.0
4.5
4.0
3.5
3.0
2.5
2.0
1.5
1.0
0.5
0.0
-0.5
f1 (ppm)

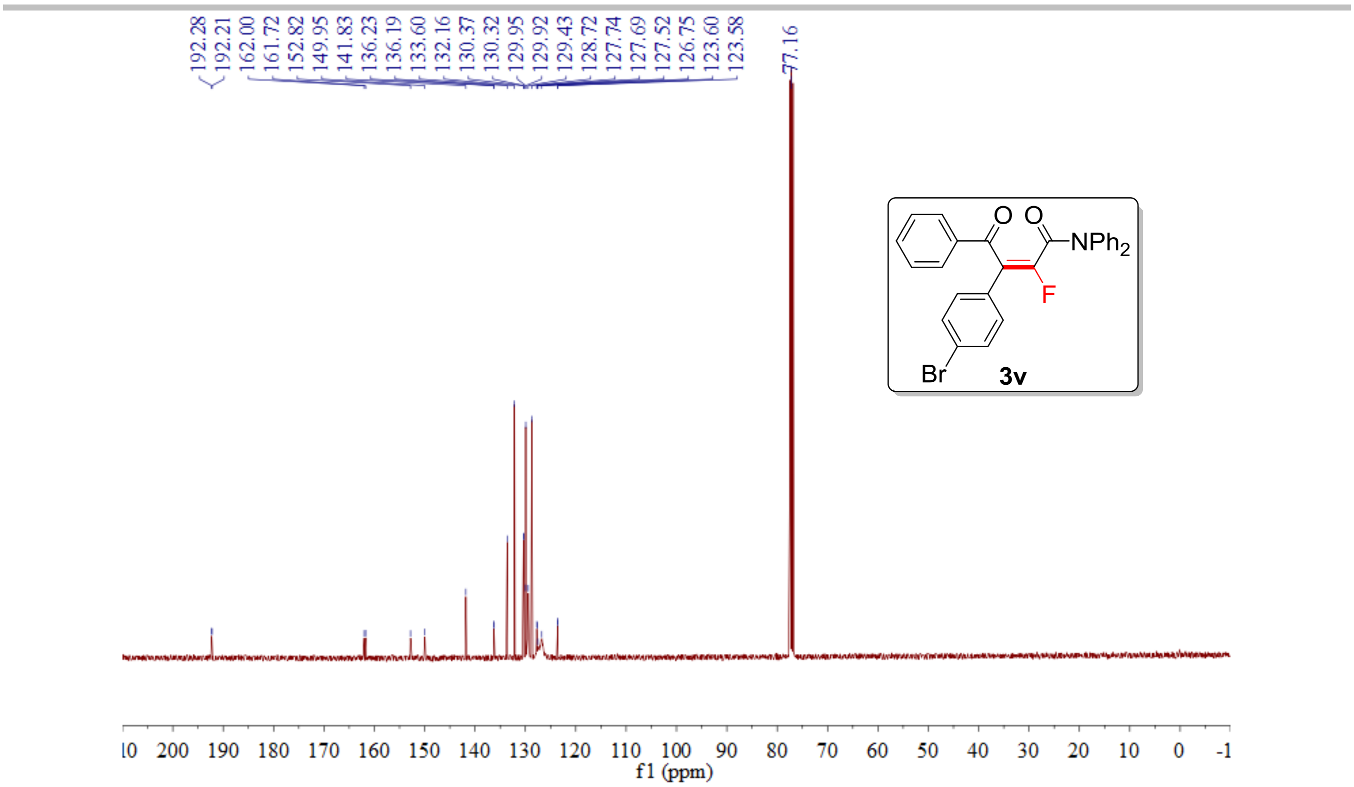
192.28
192.21
162.00
161.72
152.82
149.95
141.83
136.23
136.19
133.60
132.16
130.37
130.32
129.95
129.92
129.43
128.72
127.74
127.69
127.52
126.75
123.60
123.58
77.16
O O
NPh2
F
Br
3v
f1 (ppm)

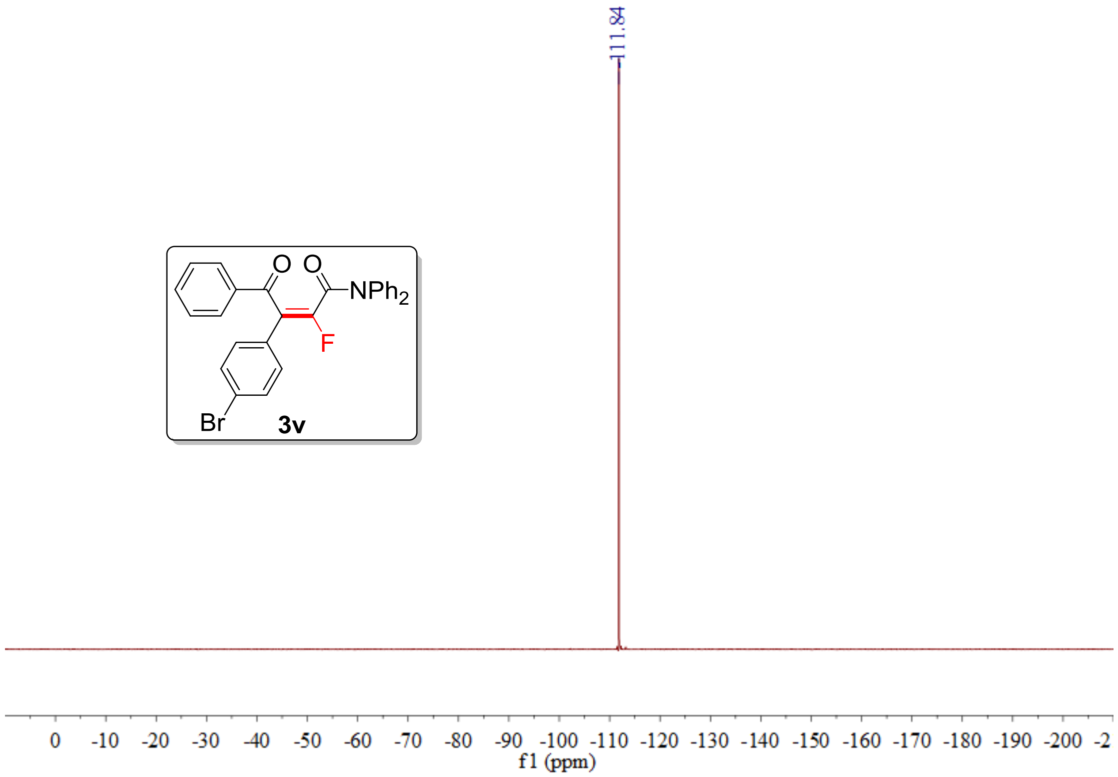
-111.84
O O
NPh2
F
Br
3v
f1 (ppm)

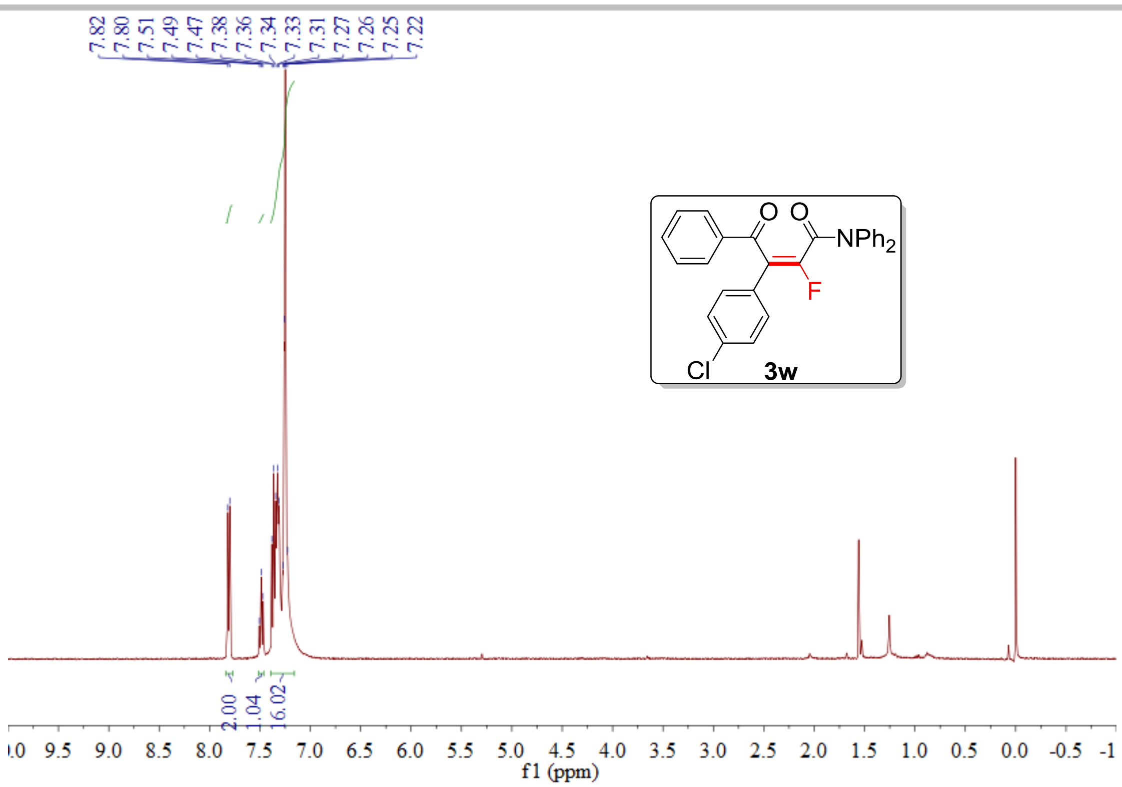

7.82
7.80
7.51
7.49
7.47
7.38
7.36
7.34
7.33
7.31
7.27
7.26
7.25
7.22
O
O
NPh2
F
Cl
3w
2.00
1.04
16.02
f1 (ppm)

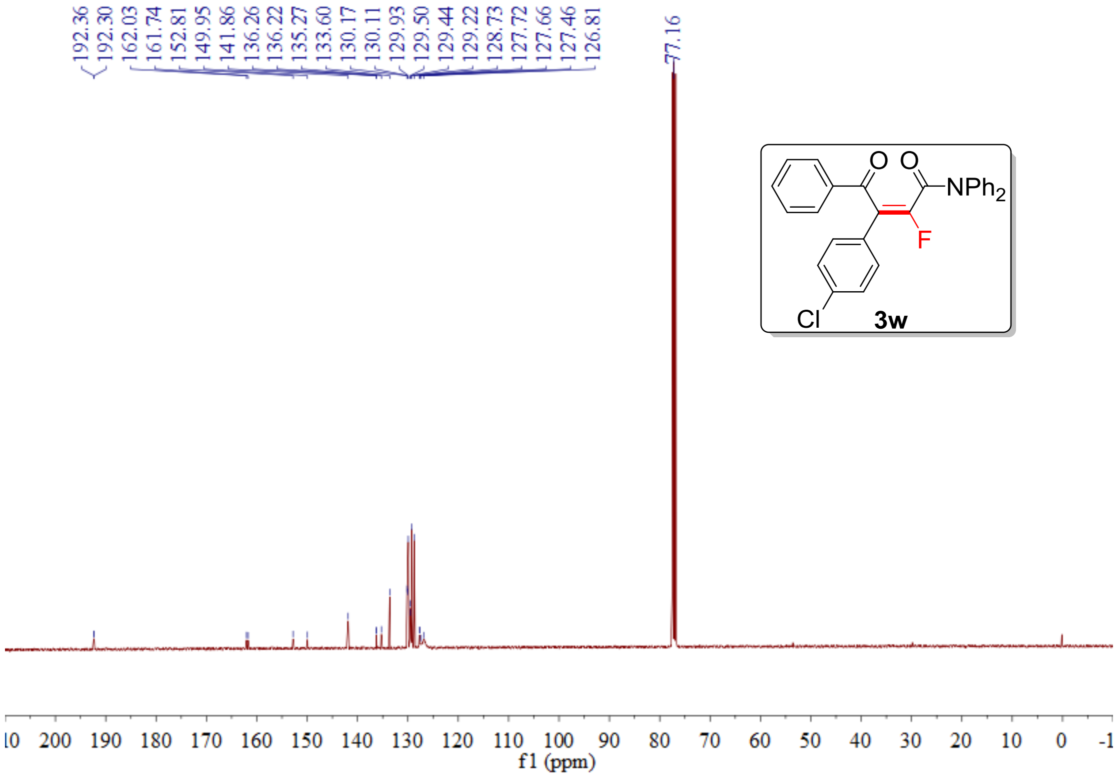

192.36
192.30
162.03
161.74
152.81
149.95
141.86
136.26
136.22
135.27
133.60
130.17
130.11
129.93
129.50
129.44
129.22
128.73
127.72
127.66
127.46
126.81
77.16
O
O
NPh2
F
Cl
3w
f1 (ppm)

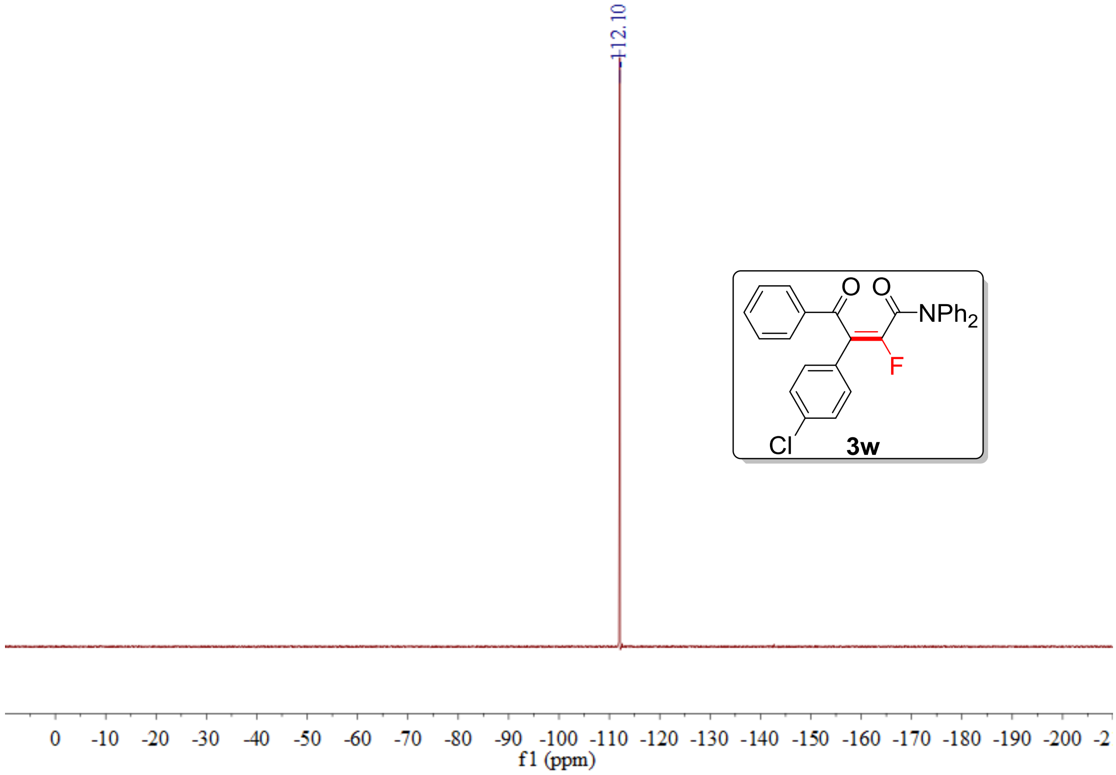

-112.10
NPh2
F
Cl
3w
0 -10 -20 -30 -40 -50 -60 -70 -80 -90 -100 -110 -120 -130 -140 -150 -160 -170 -180 -190 -200
f1 (ppm)

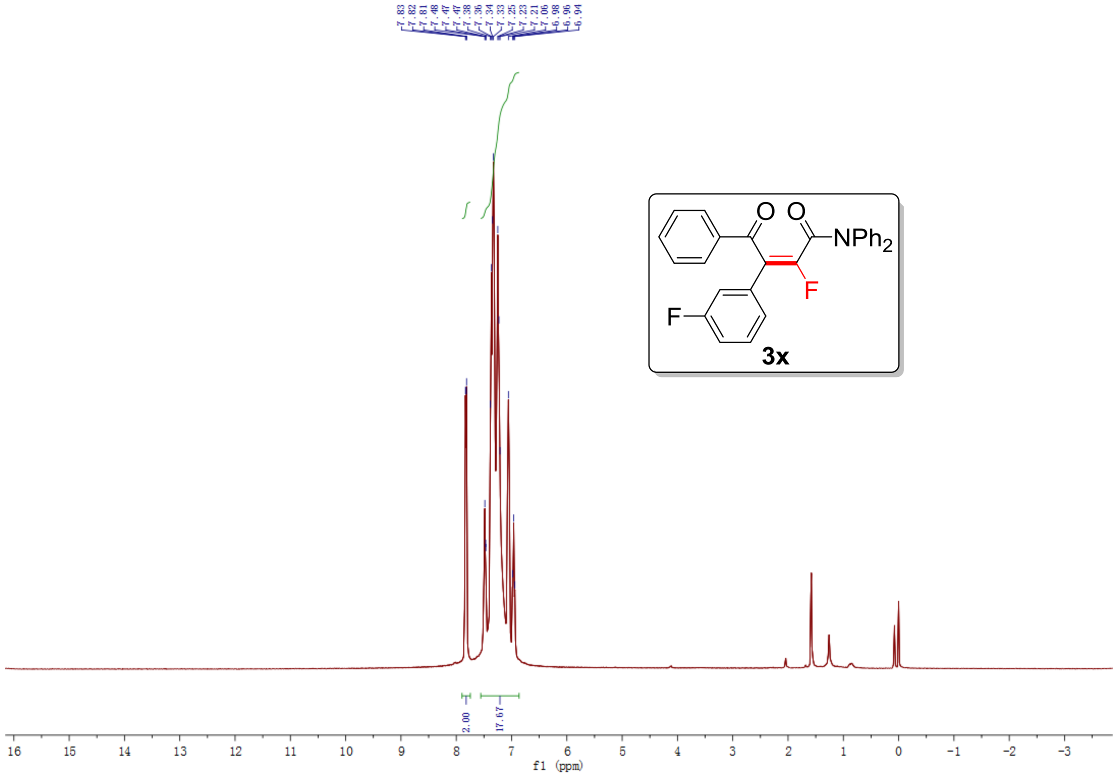

NPh2
F
F
3x
2.00
17.67
16 15 14 13 12 11 10 9 8 7 6 5 4 3 2 1 0 -1 -2 -3
f1 (ppm)

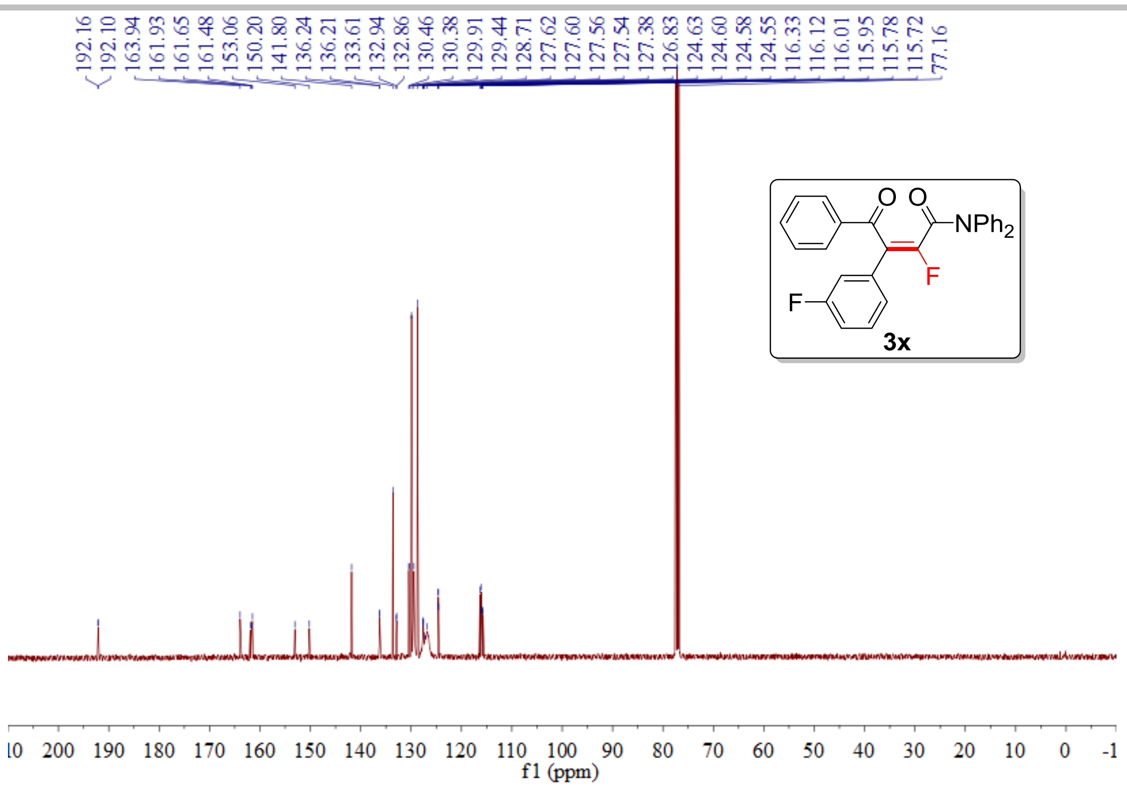
192.16
192.10
163.94
161.93
161.65
161.48
153.06
150.20
141.80
136.24
136.21
133.61
132.94
132.86
130.46
130.38
129.91
129.44
128.71
127.62
127.60
127.56
127.54
127.38
126.83
124.63
124.60
124.58
124.55
116.33
116.12
116.01
115.95
115.78
115.72
77.16
O
O
NPh2
F
F
3x
f1 (ppm)

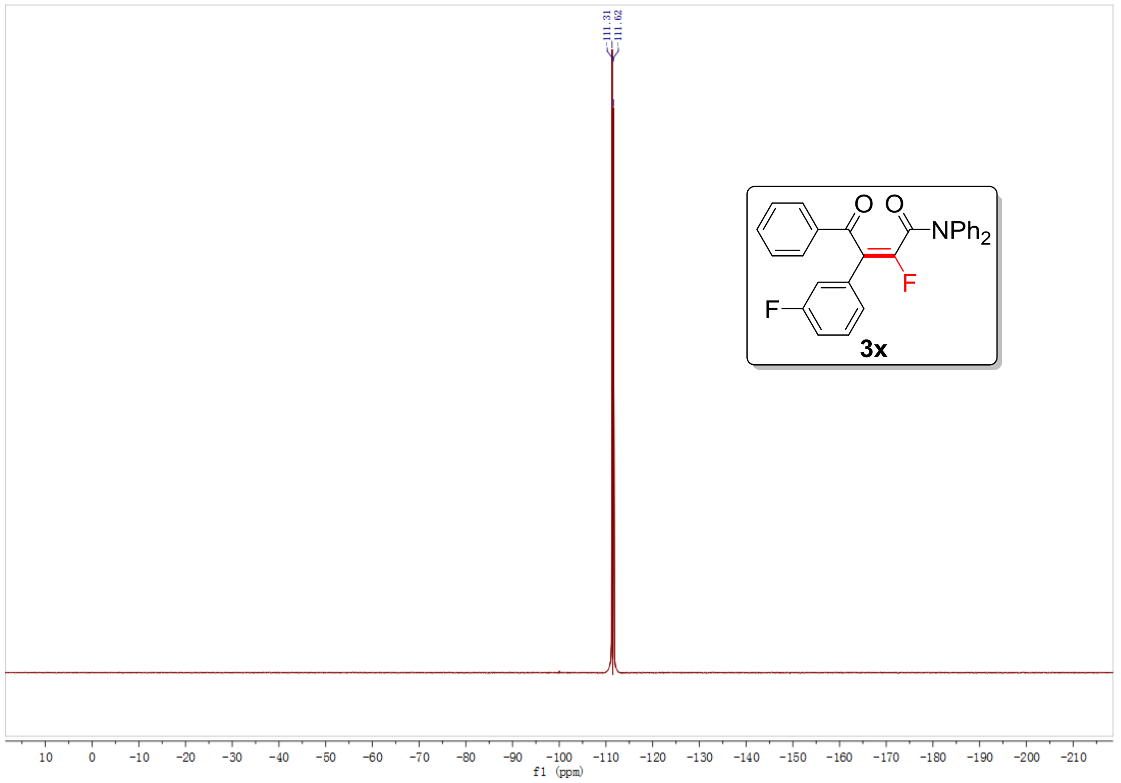
O
O
NPh2
F
F
3x
f1 (ppm)

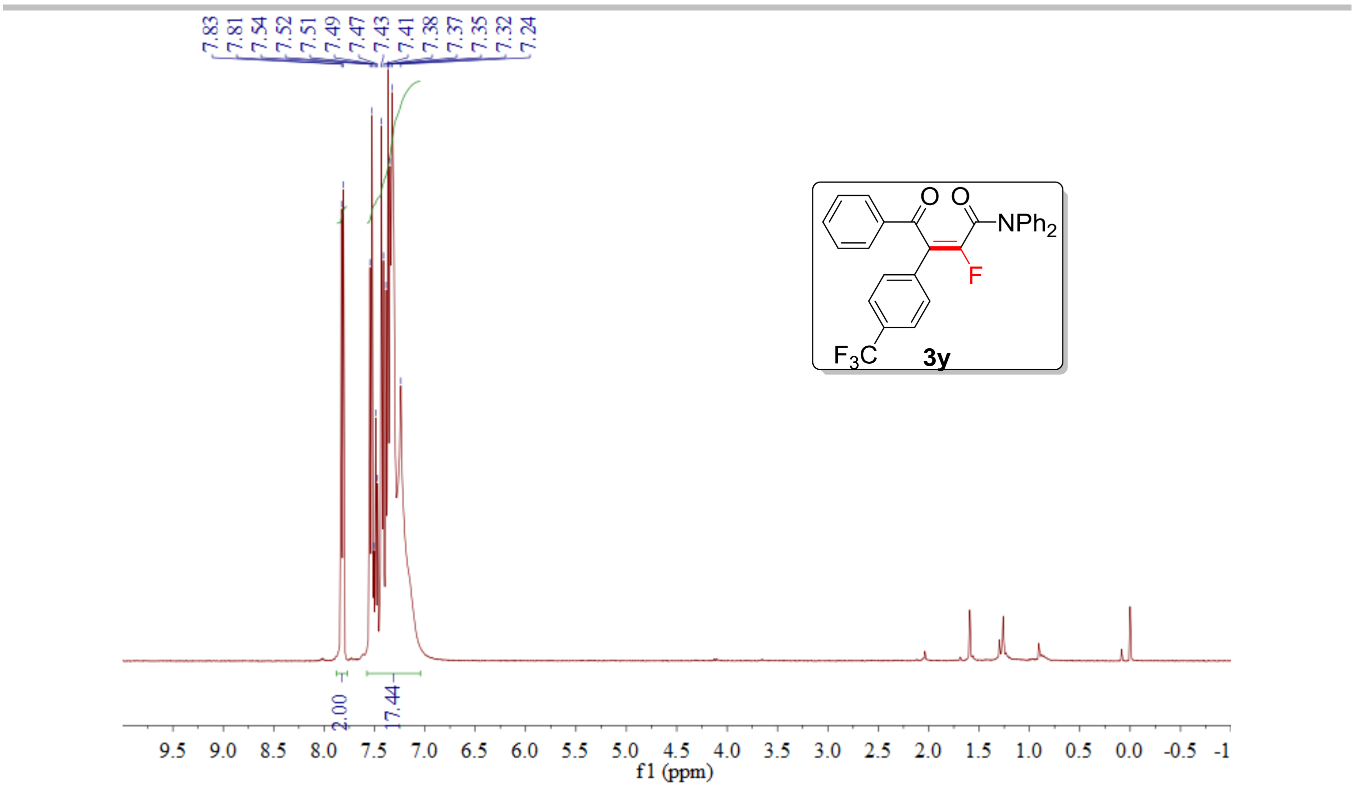

7.83
7.81
7.54
7.52
7.51
7.49
7.47
7.43
7.41
7.38
7.37
7.35
7.32
7.24
2.00
17.44
O
O
NPh2
F
F3C
3y
f1 (ppm)

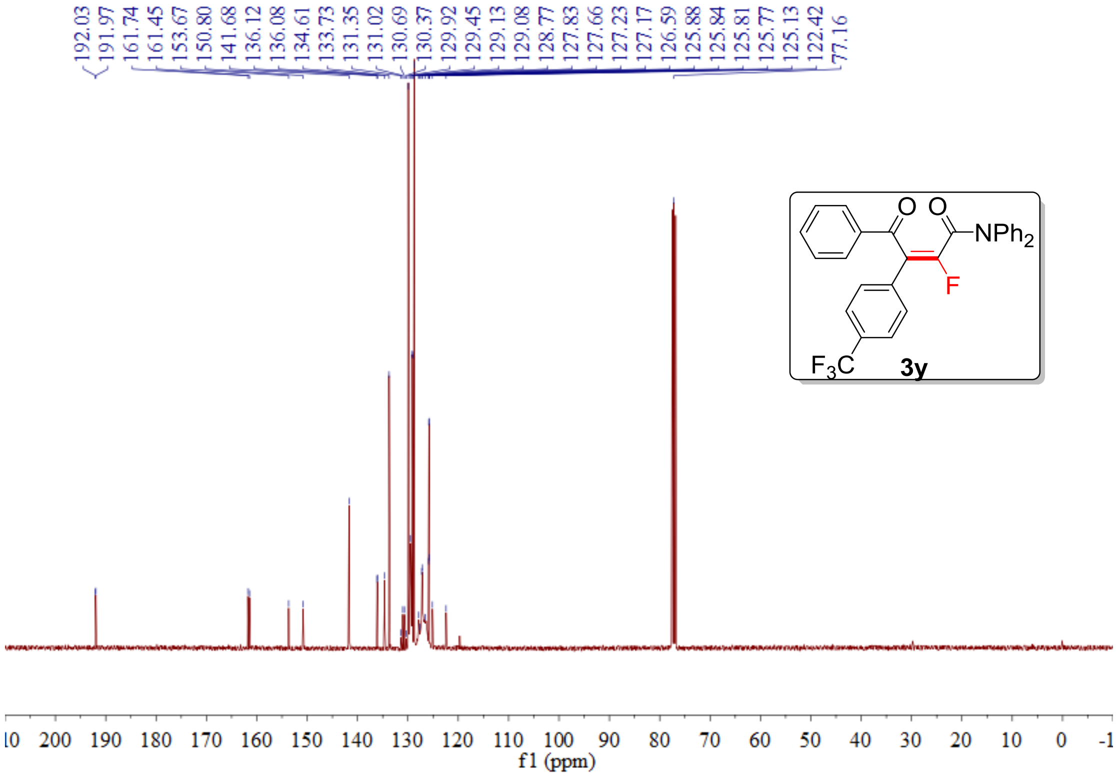

192.03
191.97
161.74
161.45
153.67
150.80
141.68
136.12
136.08
134.61
133.73
131.35
131.02
130.69
130.37
129.92
129.45
129.13
129.08
128.77
127.83
127.66
127.23
127.17
126.59
125.88
125.84
125.81
125.77
125.13
122.42
77.16
O
O
NPh2
F
F3C
3y
f1 (ppm)

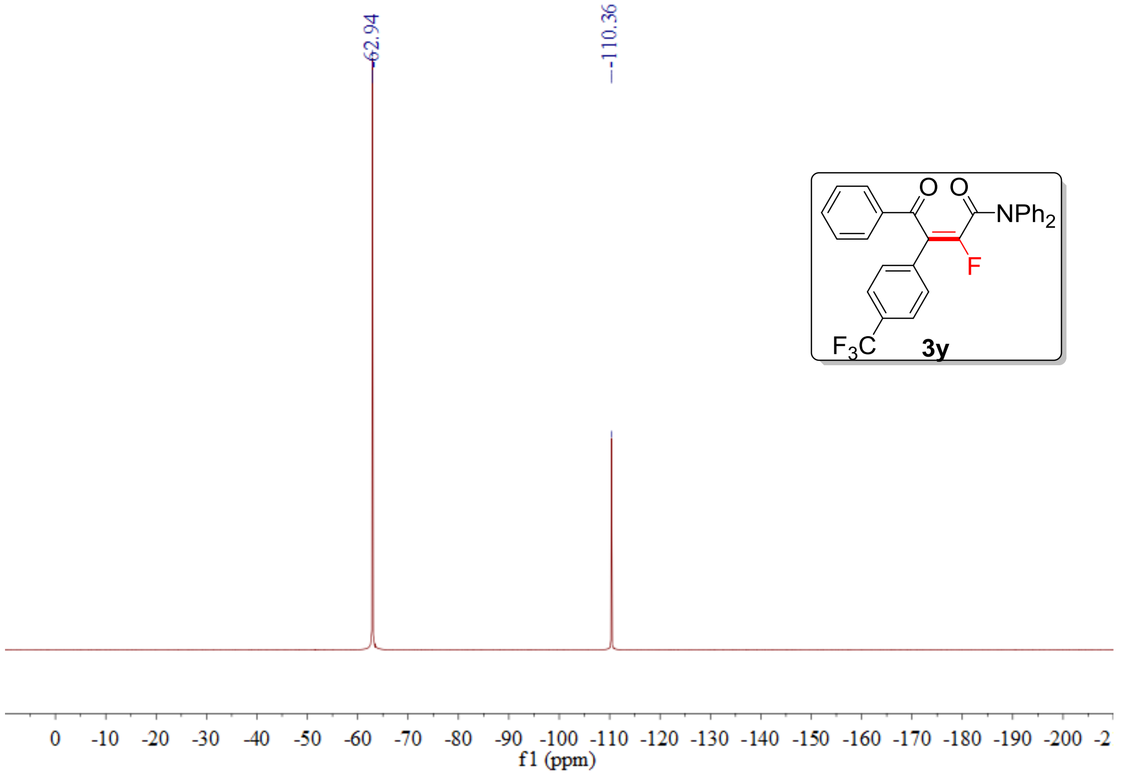
-62.94
-110.36
O
O
NPh2
F
F3C
3y
0
-10
-20
-30
-40
-50
-60
-70
-80
-90
-100
-110
-120
-130
-140
-150
-160
-170
-180
-190
-200
f1 (ppm)

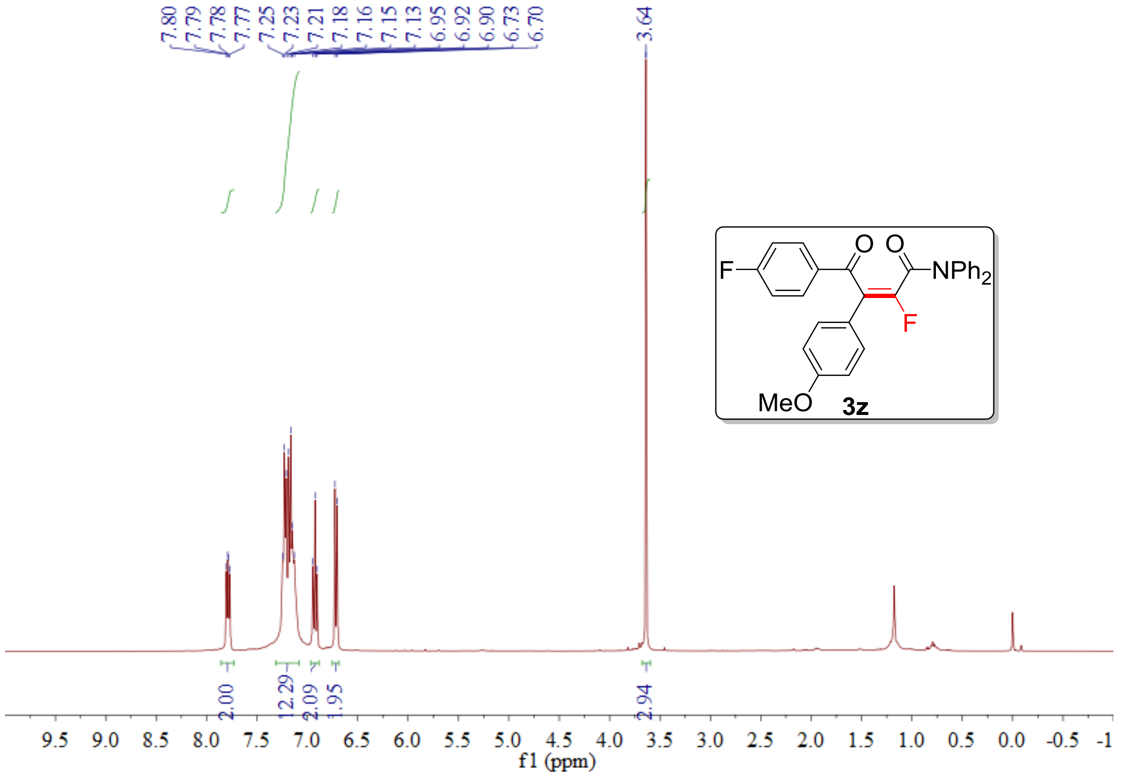
7.80
7.79
7.78
7.77
7.25
7.23
7.21
7.18
7.16
7.15
7.13
6.95
6.92
6.90
6.73
6.70
3.64
F
O
O
NPh2
F
MeO
3z
2.00
12.29
2.09
1.95
2.94
9.5
9.0
8.5
8.0
7.5
7.0
6.5
6.0
5.5
5.0
4.5
4.0
3.5
3.0
2.5
2.0
1.5
1.0
0.5
0.0
-0.5
f1 (ppm)

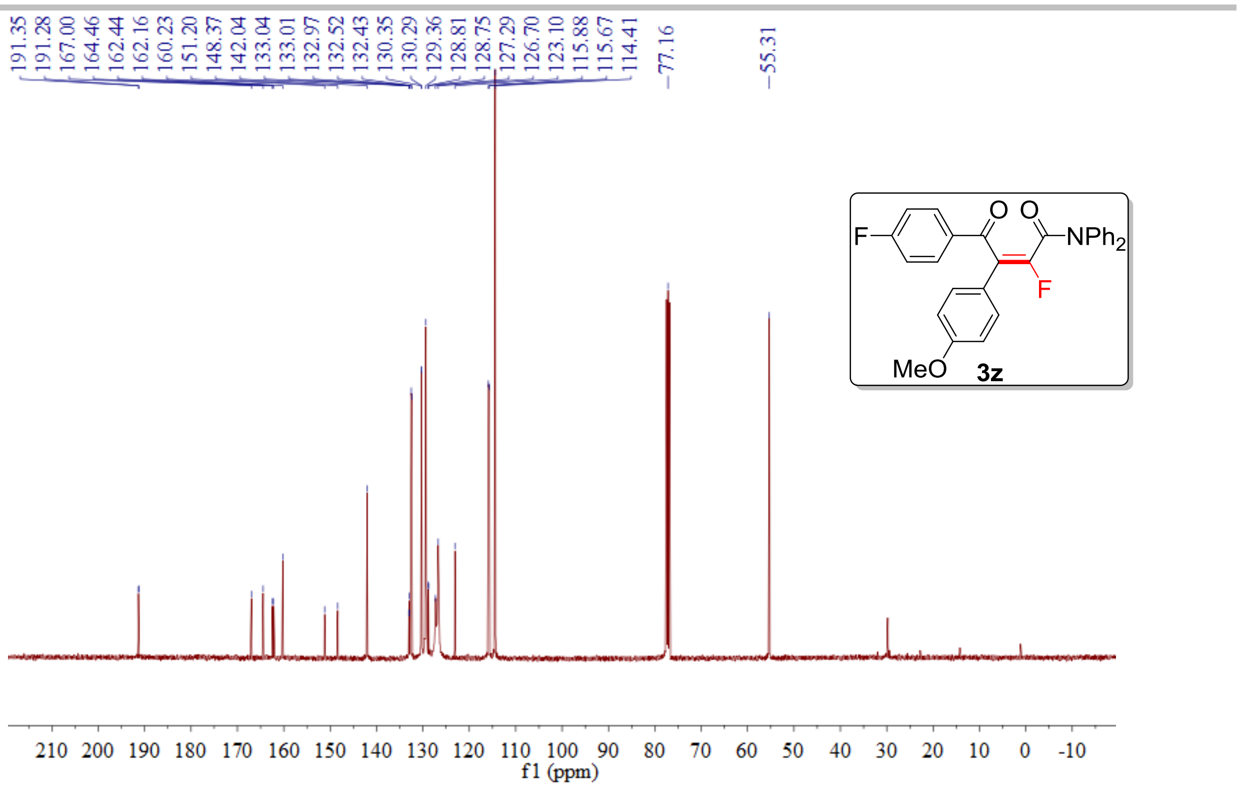
191.35
191.28
167.00
164.46
162.44
162.16
160.23
151.20
148.37
142.04
133.04
133.01
132.97
132.52
132.43
130.35
130.29
129.36
128.81
128.75
127.29
126.70
123.10
115.88
115.67
114.41
77.16
55.31
F
O
O
NPh2
F
MeO
3z
210 200 190 180 170 160 150 140 130 120 110 100 90 80 70 60 50 40 30 20 10 0 -10
f1 (ppm)

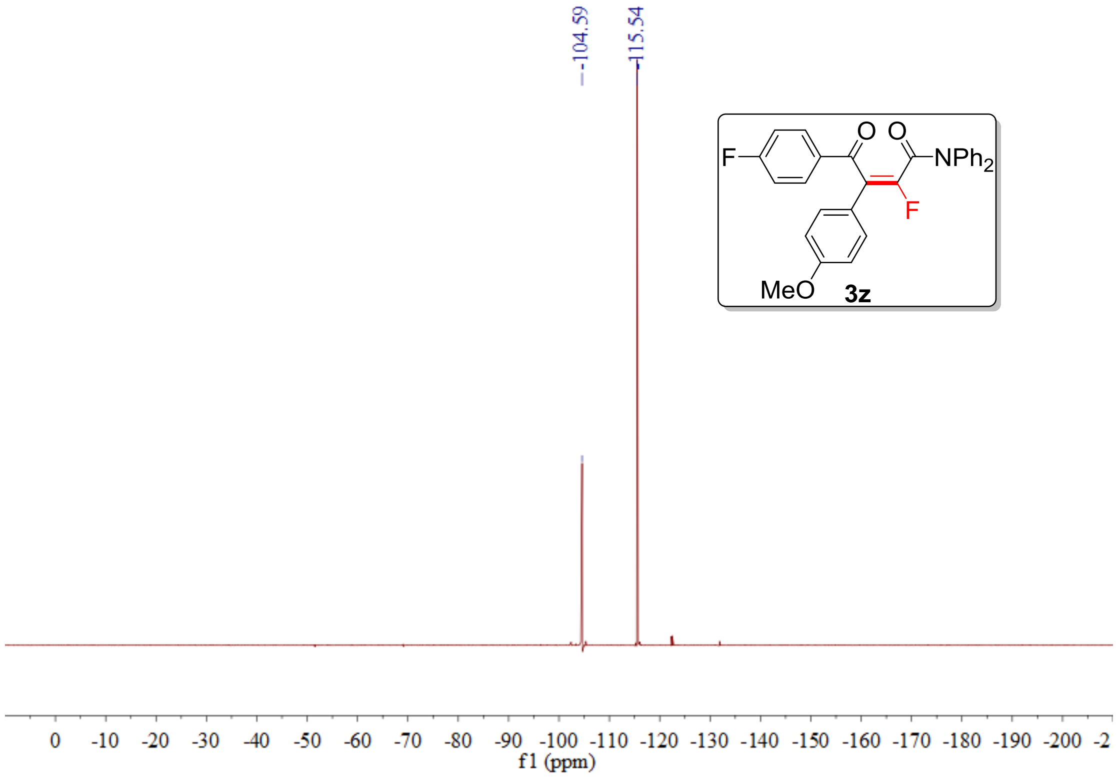
-104.59
-115.54
F
O
O
NPh2
F
MeO
3z
0 -10 -20 -30 -40 -50 -60 -70 -80 -90 -100 -110 -120 -130 -140 -150 -160 -170 -180 -190 -200 -2
f1 (ppm)

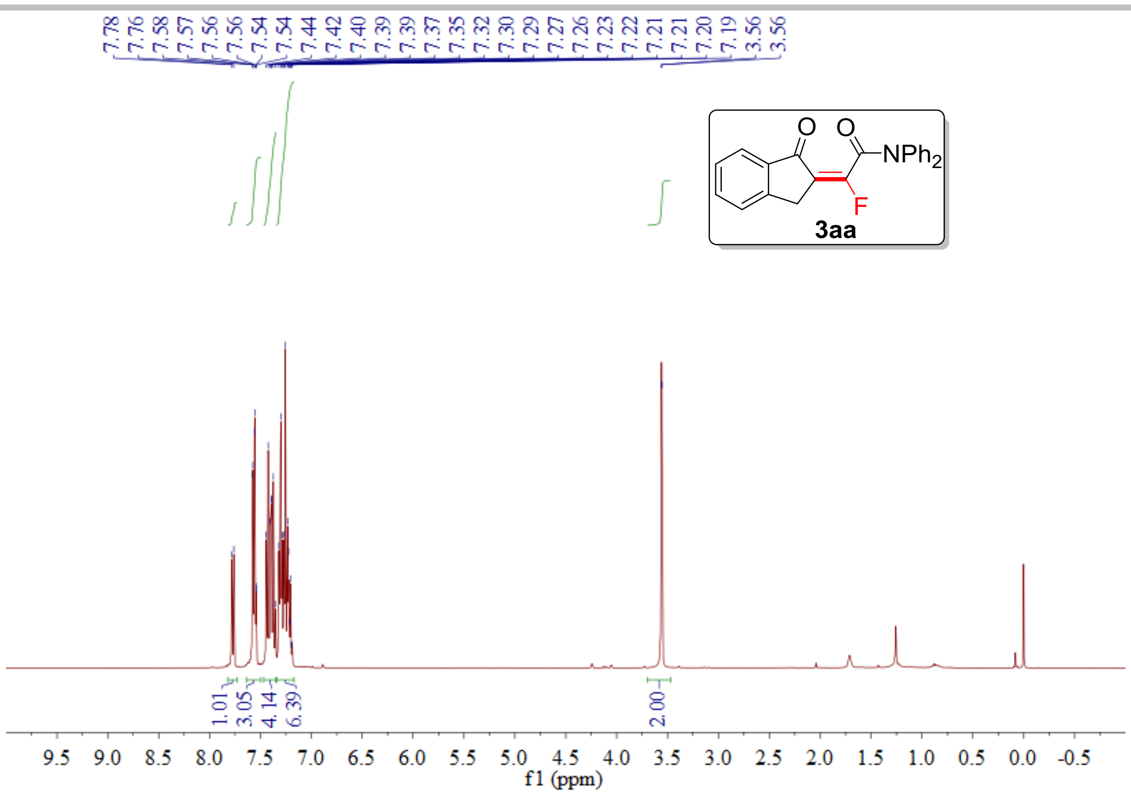
7.78
7.76
7.58
7.57
7.56
7.56
7.54
7.54
7.44
7.42
7.40
7.39
7.39
7.37
7.35
7.32
7.30
7.29
7.27
7.26
7.23
7.22
7.21
7.21
7.20
7.19
3.56
3.56
3aa
1.01
3.05
4.14
6.39
2.00
f1 (ppm)

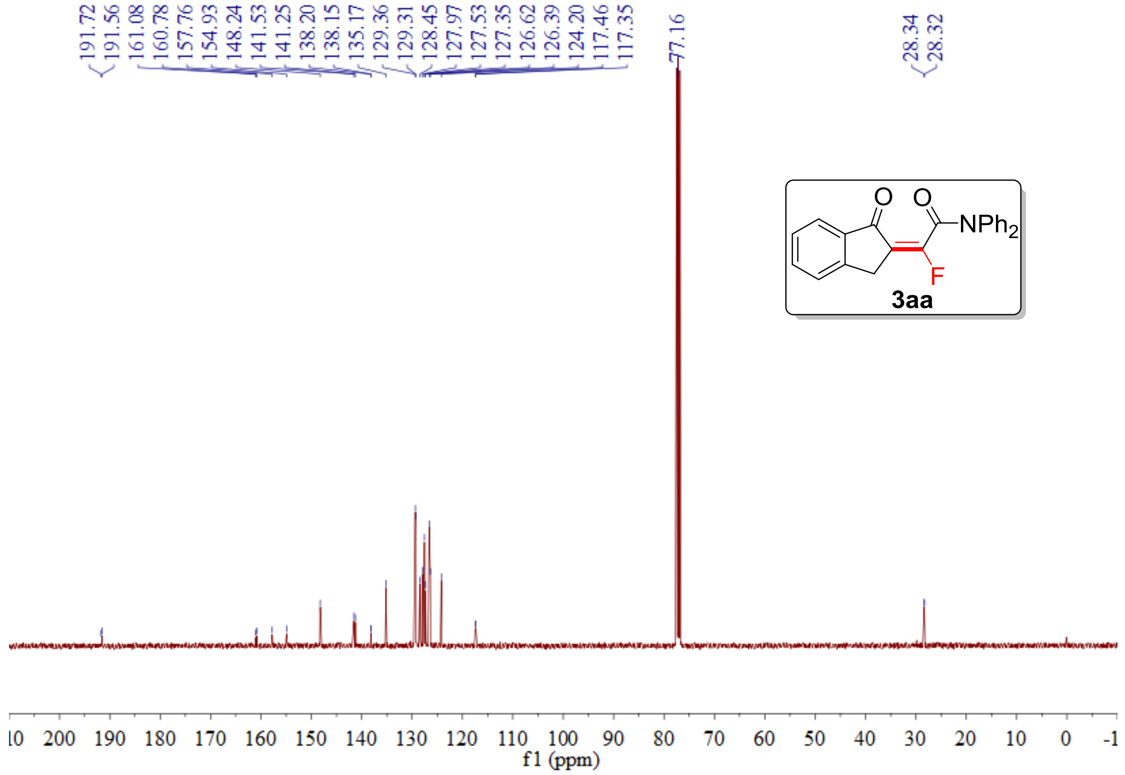
191.72
191.56
161.08
160.78
157.76
154.93
148.24
141.53
141.25
138.20
138.15
135.17
129.36
129.31
128.45
127.97
127.53
127.35
126.62
126.39
124.20
117.46
117.35
77.16
28.34
28.32
3aa
f1 (ppm)

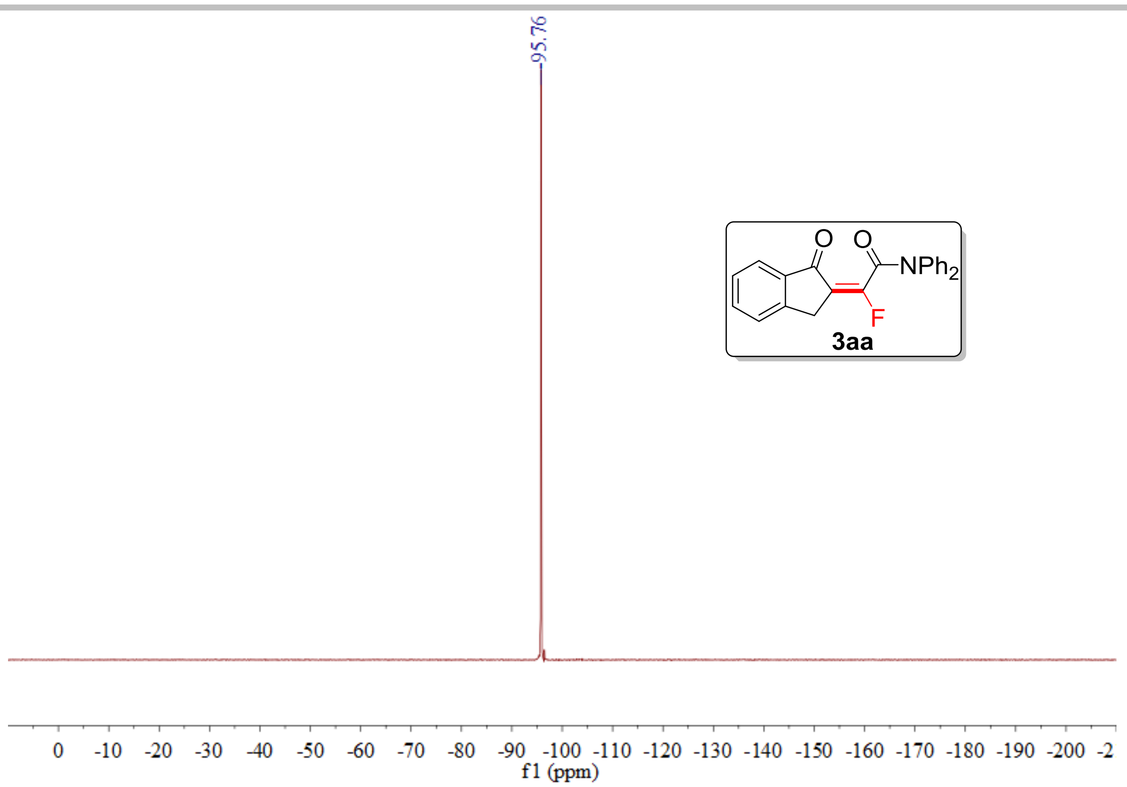
-95.76
NPh2
F
3aa
0 -10 -20 -30 -40 -50 -60 -70 -80 -90 -100 -110 -120 -130 -140 -150 -160 -170 -180 -190 -200
f1 (ppm)

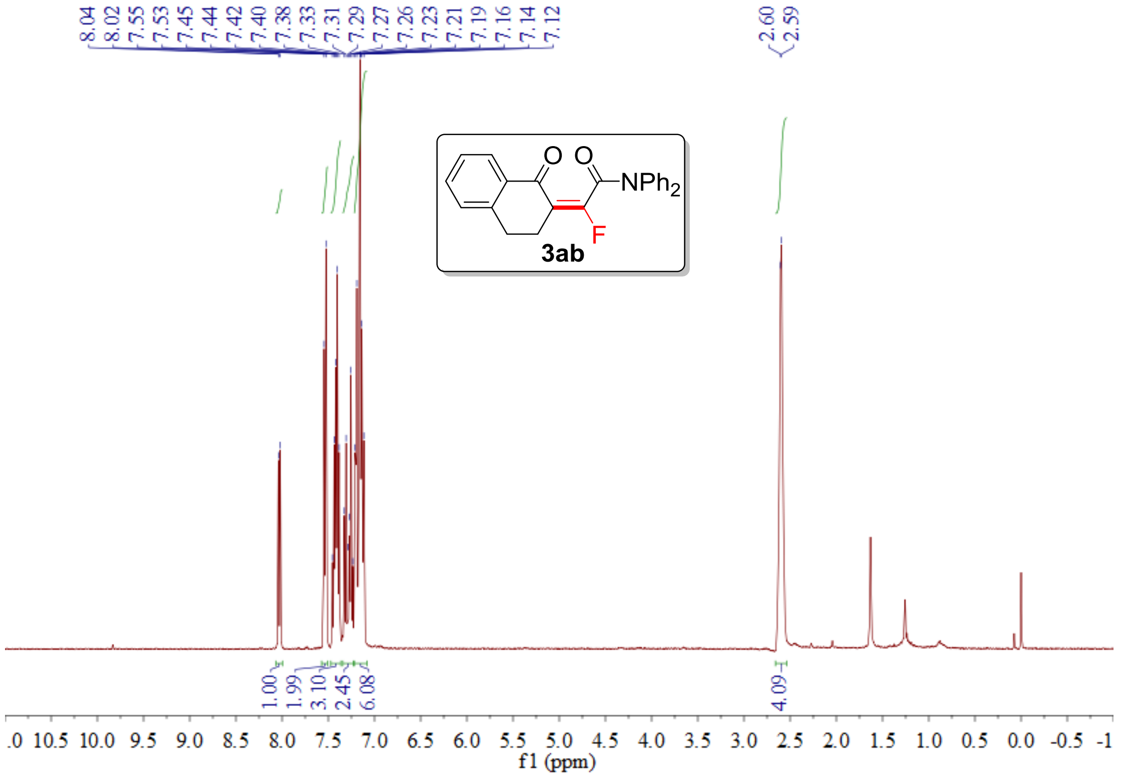
8.04 8.02 7.55 7.53 7.45 7.44 7.42 7.40 7.38 7.33 7.31 7.29 7.27 7.26 7.23 7.21 7.19 7.16 7.14 7.12
2.60 2.59
NPh2
F
3ab
1.00 1.99 3.10 2.45 6.08
4.09
10.5 10.0 9.5 9.0 8.5 8.0 7.5 7.0 6.5 6.0 5.5 5.0 4.5 4.0 3.5 3.0 2.5 2.0 1.5 1.0 0.5 0.0 -0.5
f1 (ppm)

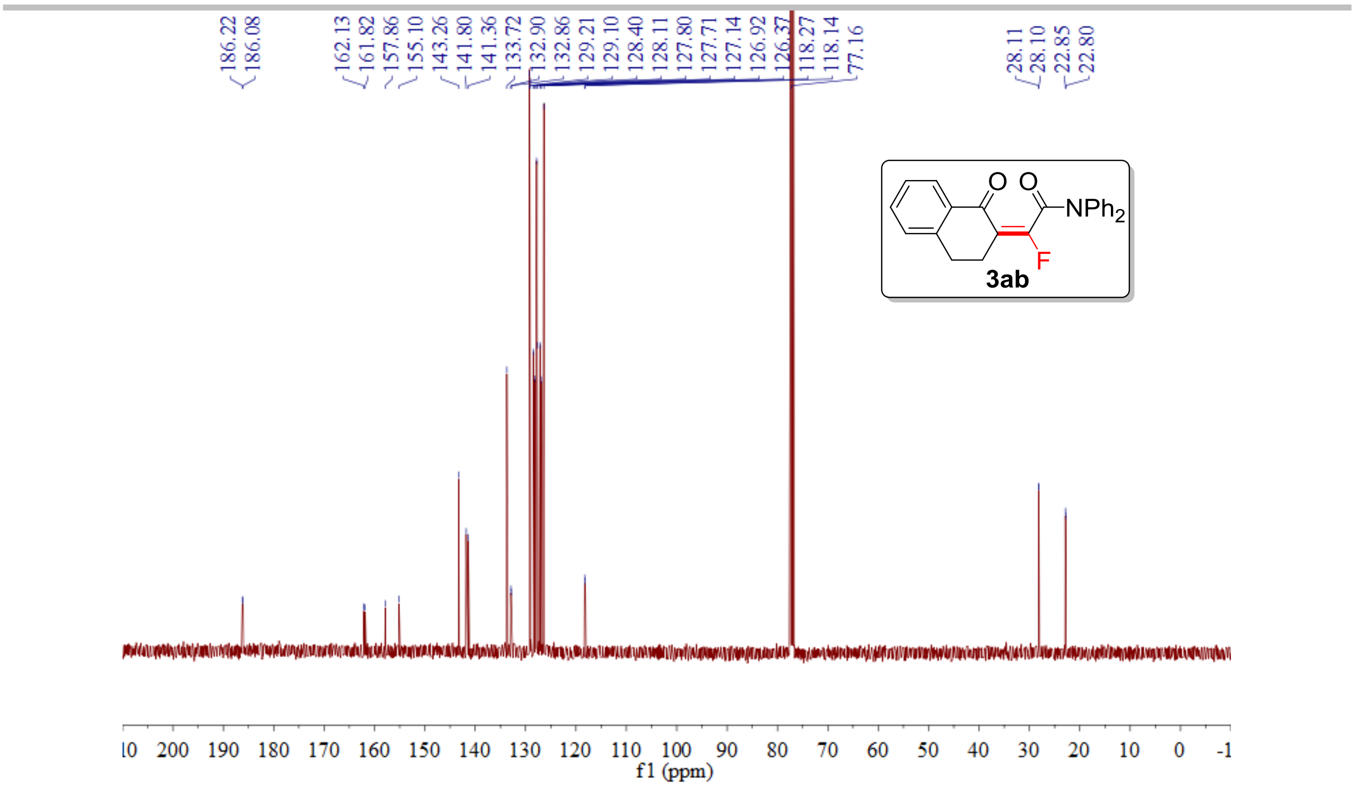

186.22
186.08
162.13
161.82
157.86
155.10
143.26
141.80
141.36
133.72
132.90
132.86
129.21
129.10
128.40
128.11
127.80
127.71
127.14
126.92
126.37
118.27
118.14
77.16
28.11
28.10
22.85
22.80
O O
NPh2
F
3ab
f1 (ppm)

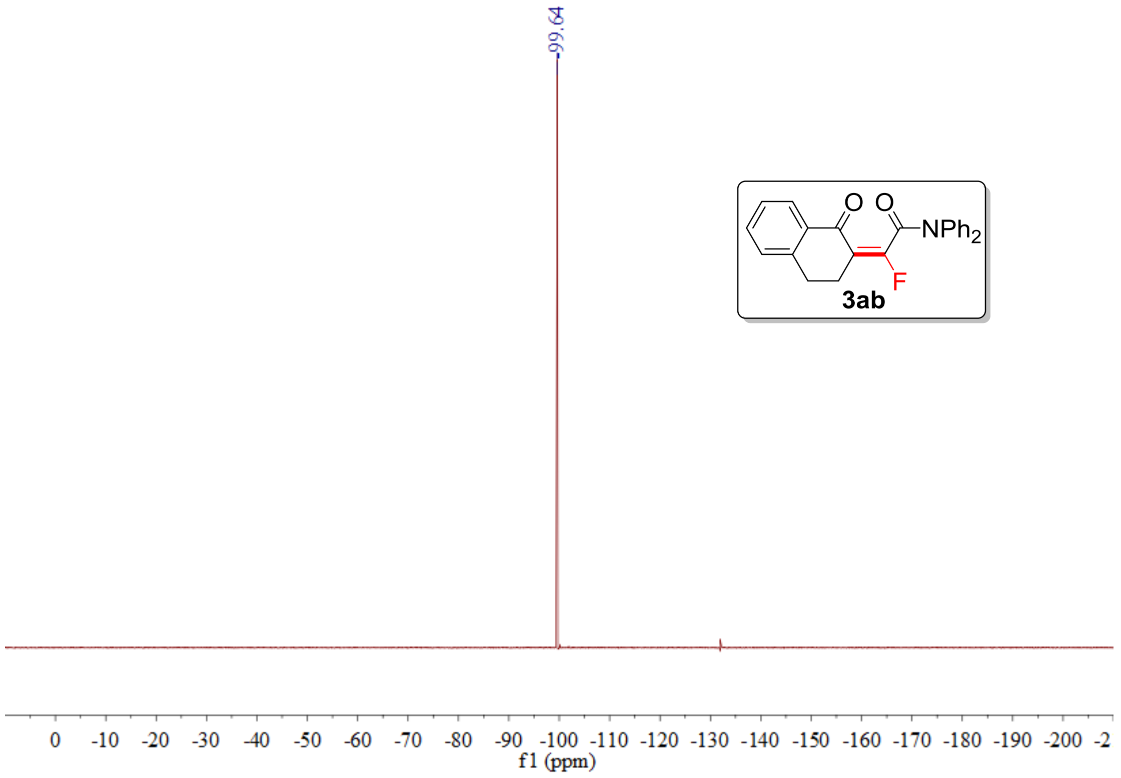

-99.64
O O
NPh2
F
3ab
f1 (ppm)

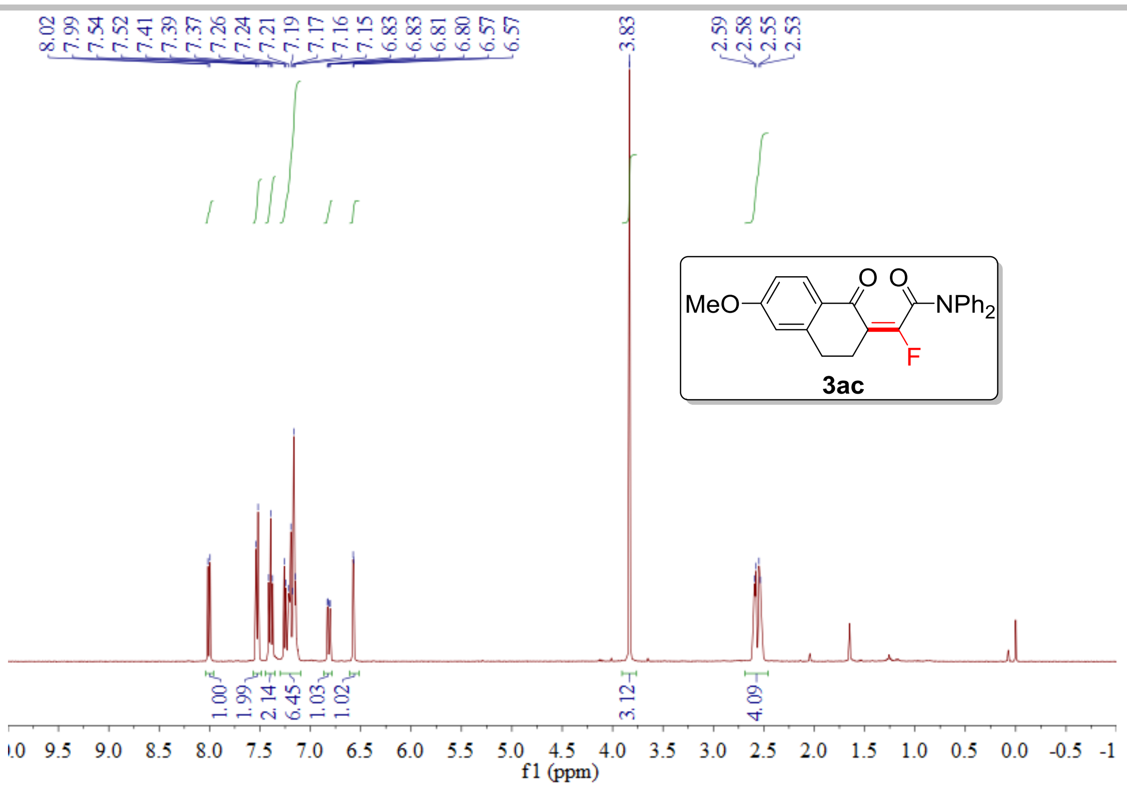
8.02 7.99 7.54 7.52 7.41 7.39 7.37 7.26 7.24 7.21 7.19 7.17 7.16 7.15 6.83 6.83 6.81 6.80 6.57 6.57
3.83
2.59 2.58 2.55 2.53
MeO
O
O
NPh2
F
3ac
1.00 1.99 2.14 6.45 1.03 1.02
3.12
4.09
f1 (ppm)

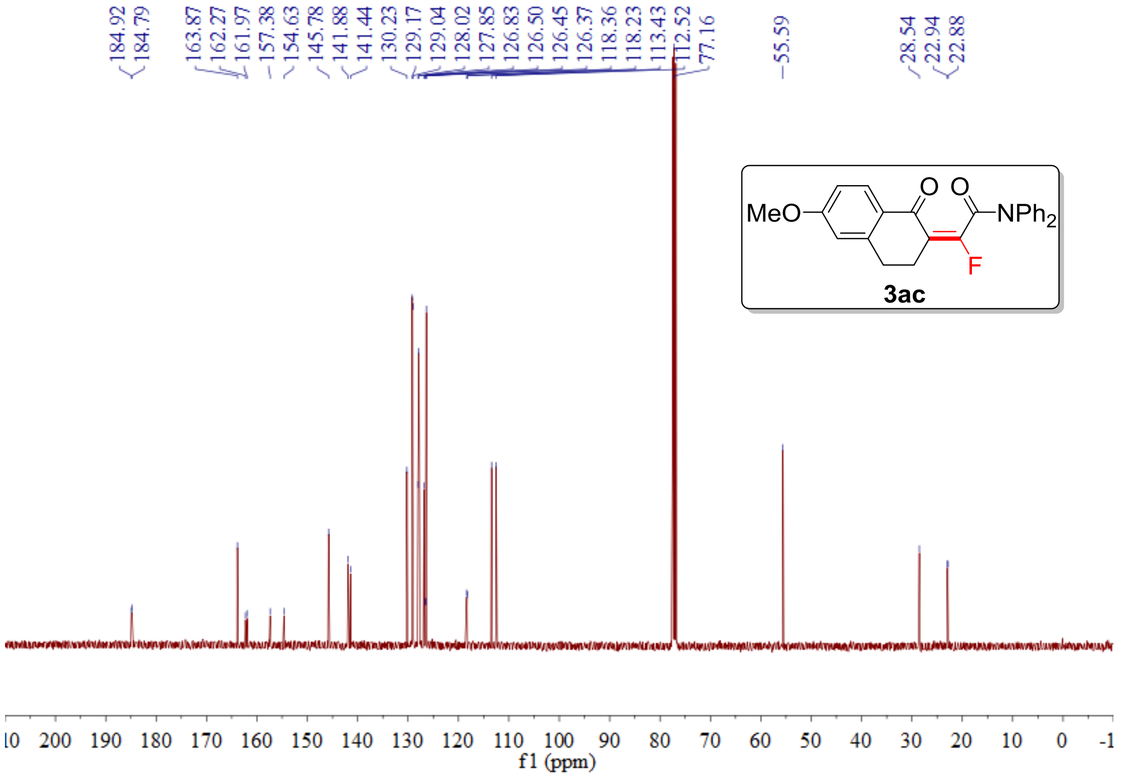
184.92 184.79
163.87 162.27 161.97 157.38 154.63 145.78 141.88 141.44 130.23 129.17 129.04 128.02 127.85 126.83 126.50 126.45 126.37 118.36 118.23 113.43 112.52 77.16
55.59
28.54 22.94 22.88
MeO
O
O
NPh2
F
3ac
f1 (ppm)

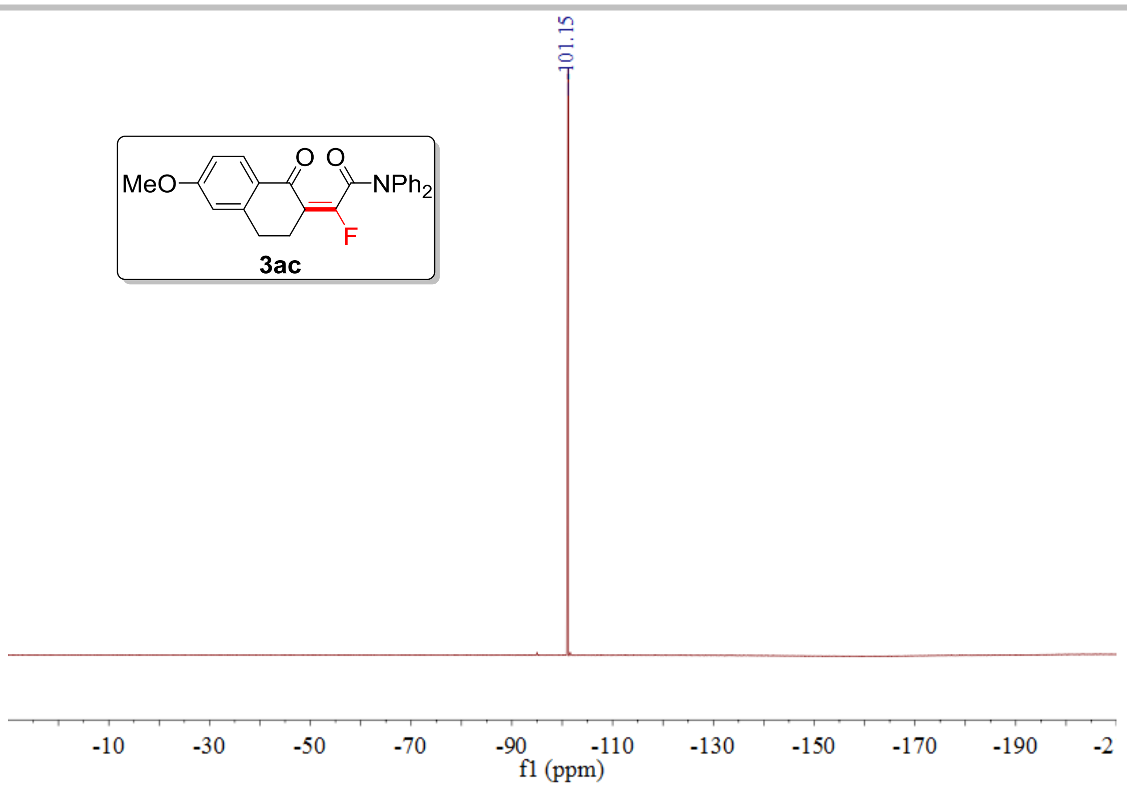
MeO
O
O
NPh2
F
3ac
-101.15
-10
-30
-50
-70
-90
-110
-130
-150
-170
-190
f1 (ppm)

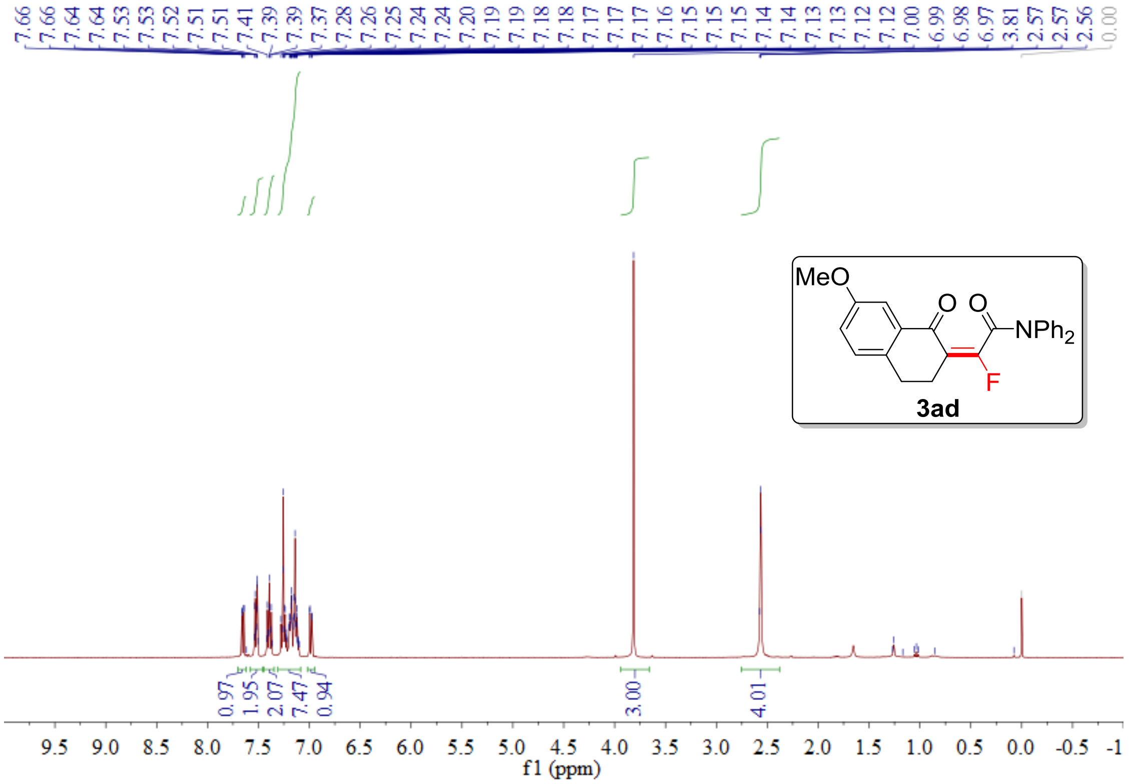
MeO
O
O
NPh2
F
3ad
3.81
2.57
2.56
0.00
0.97
1.95
2.07
7.47
0.94
3.00
4.01
f1 (ppm)

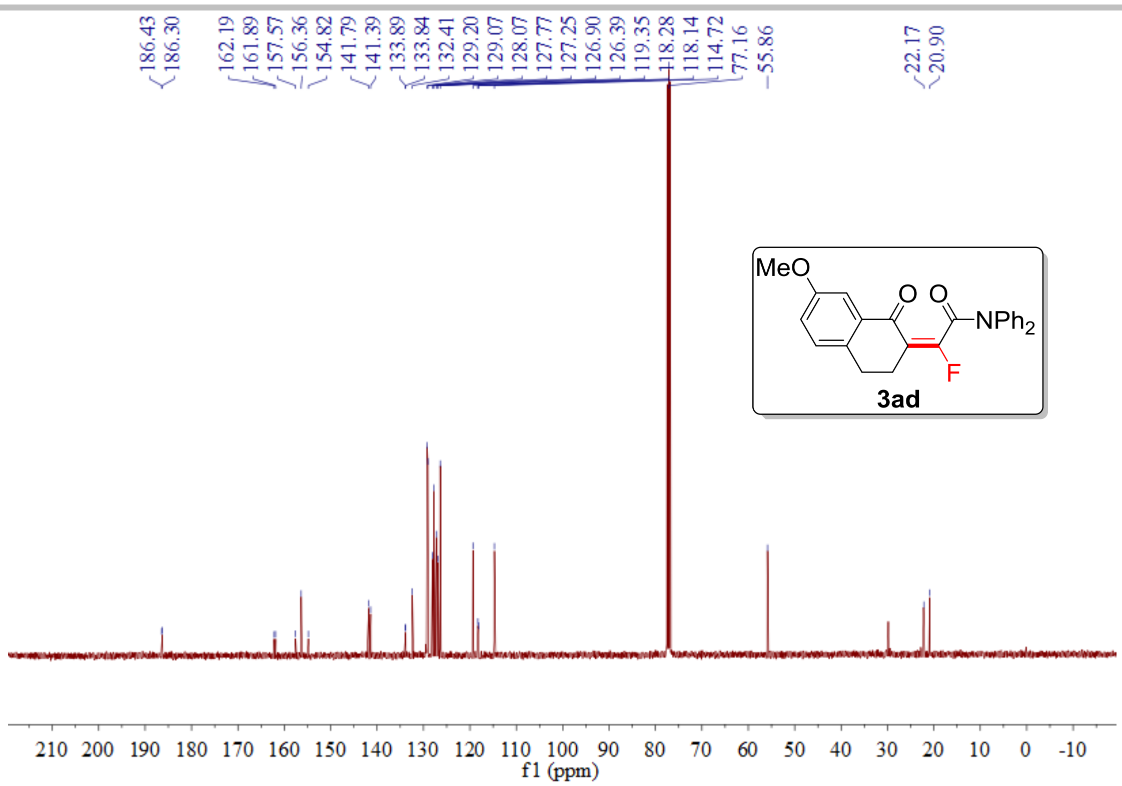
186.43
186.30
162.19
161.89
157.57
156.36
154.82
141.79
141.39
133.89
133.84
132.41
129.20
129.07
128.07
127.77
127.25
126.90
126.39
119.35
118.28
118.14
114.72
77.16
55.86
22.17
20.90
MeO
O
O
NPh2
F
3ad
210 200 190 180 170 160 150 140 130 120 110 100 90 80 70 60 50 40 30 20 10 0 -10
f1 (ppm)

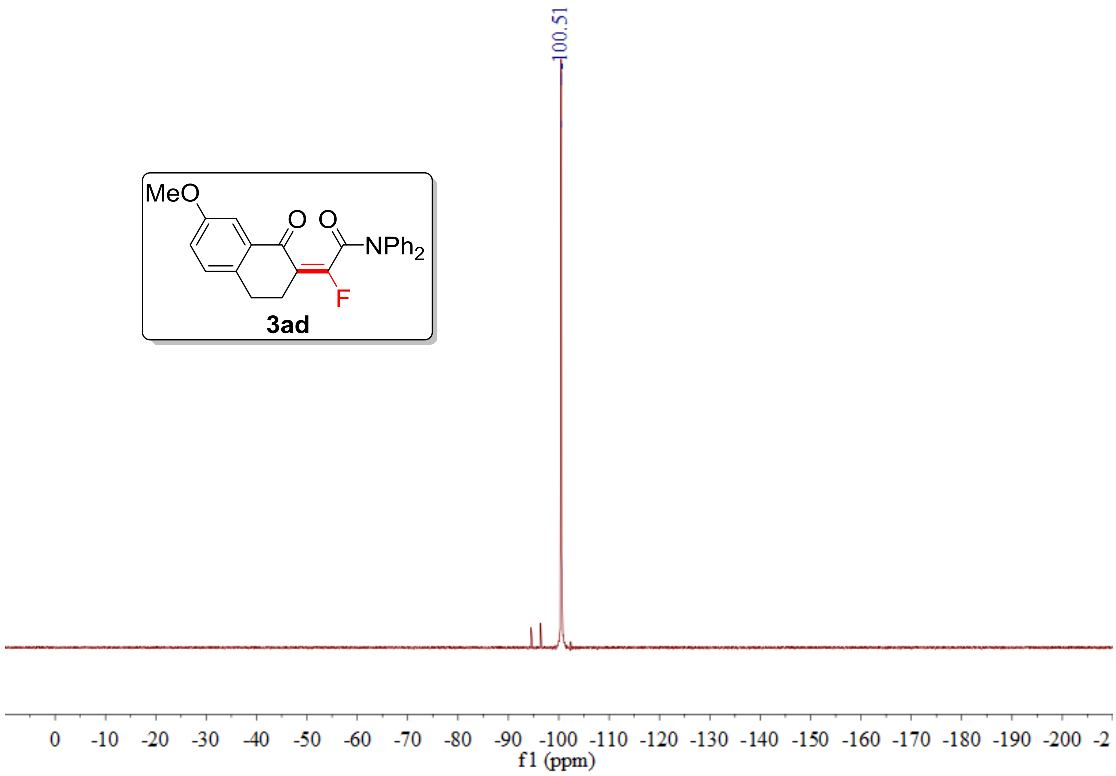
100.51
MeO
O
O
NPh2
F
3ad
0 -10 -20 -30 -40 -50 -60 -70 -80 -90 -100 -110 -120 -130 -140 -150 -160 -170 -180 -190 -200 -2
f1 (ppm)

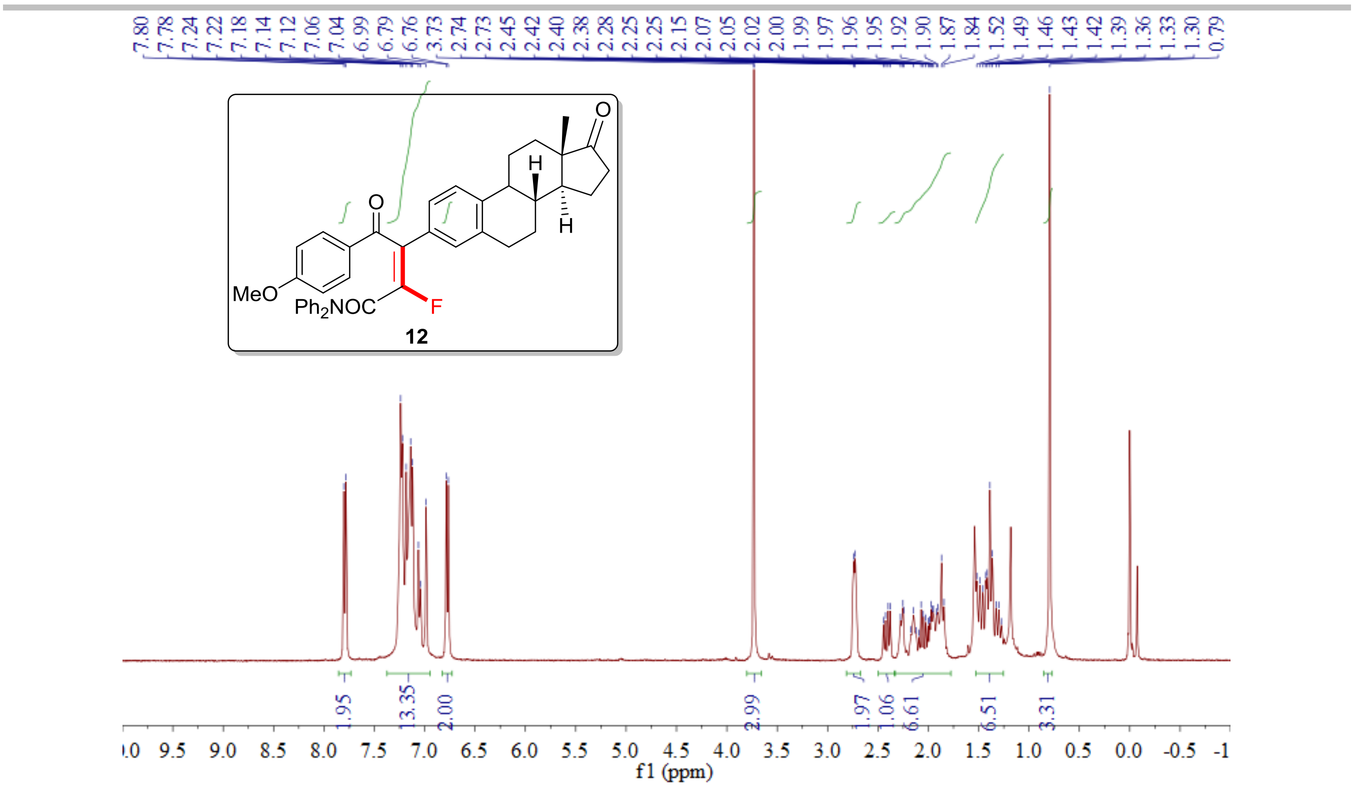

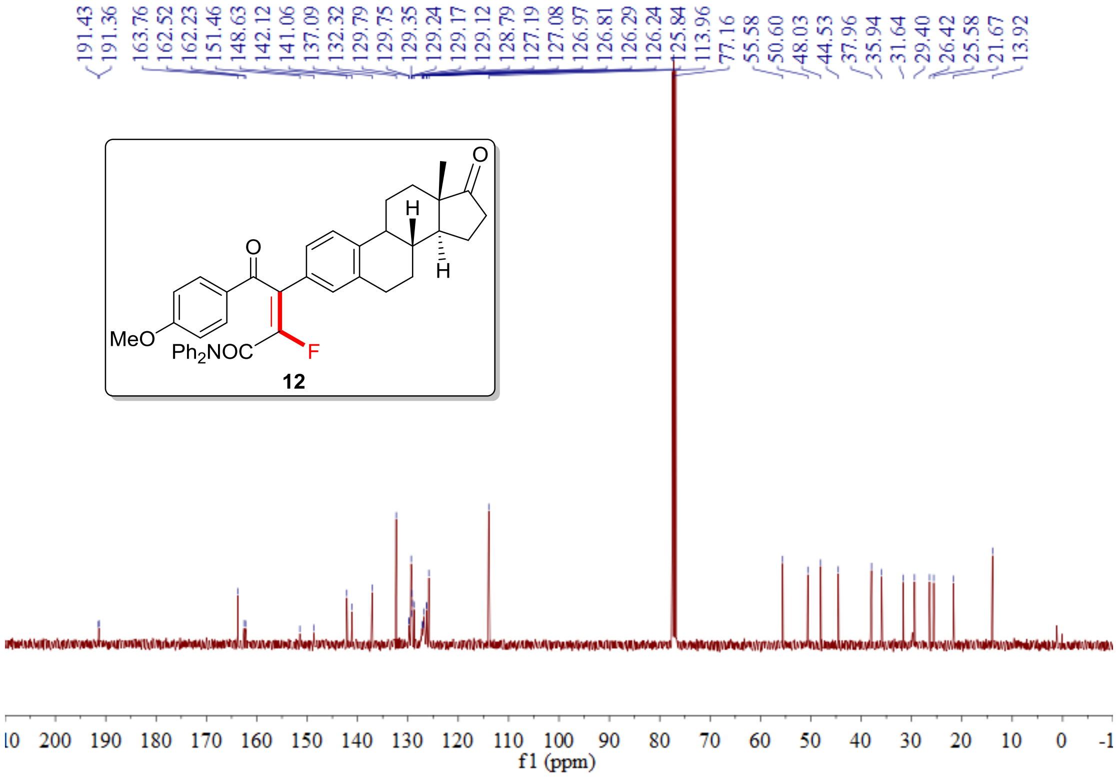

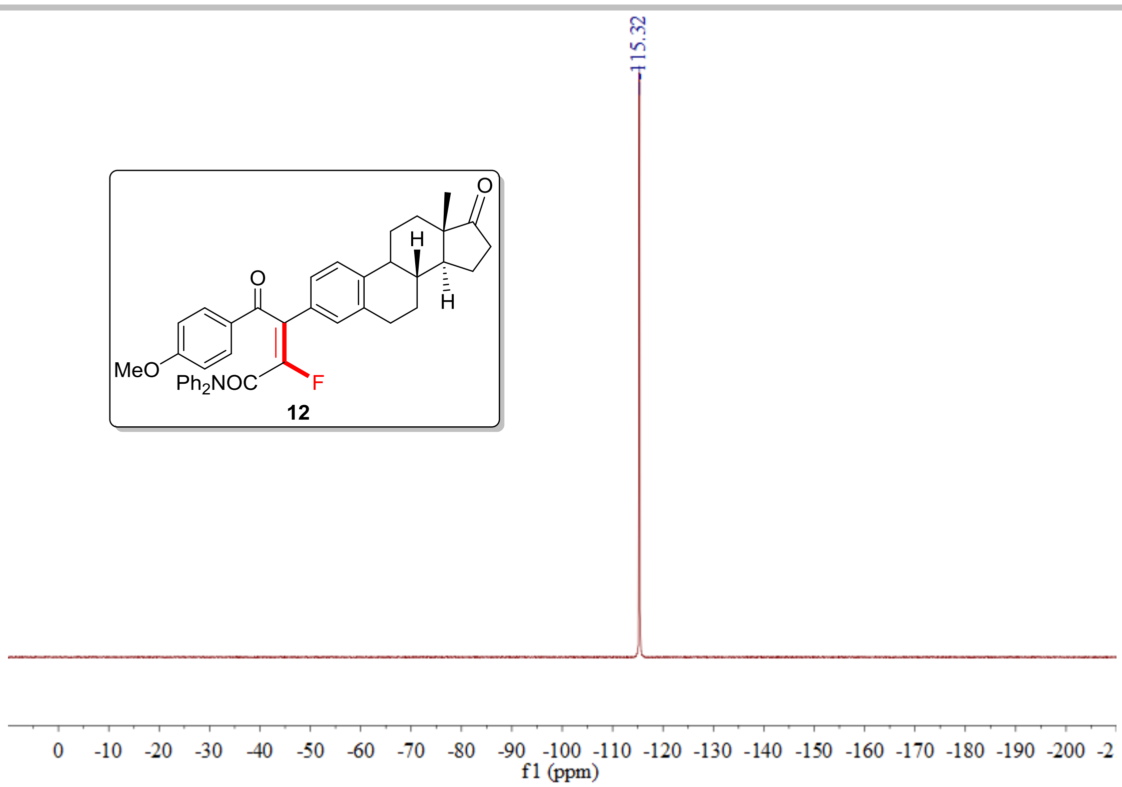

-115.32
MeO
Ph2NOC
F
O
H
H
12
0 -10 -20 -30 -40 -50 -60 -70 -80 -90 -100 -110 -120 -130 -140 -150 -160 -170 -180 -190 -200
f1 (ppm)

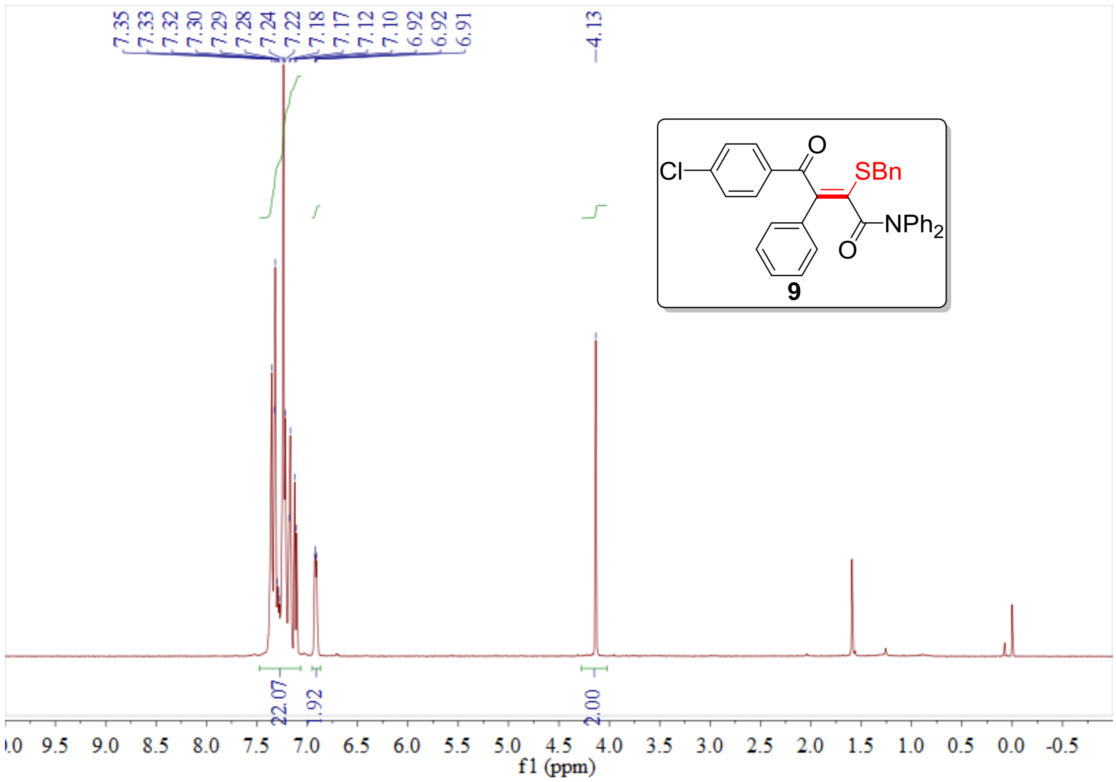

7.35 7.33 7.32 7.30 7.29 7.28 7.24 7.22 7.18 7.17 7.12 7.10 6.92 6.92 6.91
4.13
Cl
O
SBn
NPh2
O
9
22.07
1.92
2.00
9.5 9.0 8.5 8.0 7.5 7.0 6.5 6.0 5.5 5.0 4.5 4.0 3.5 3.0 2.5 2.0 1.5 1.0 0.5 0.0 -0.5
f1 (ppm)

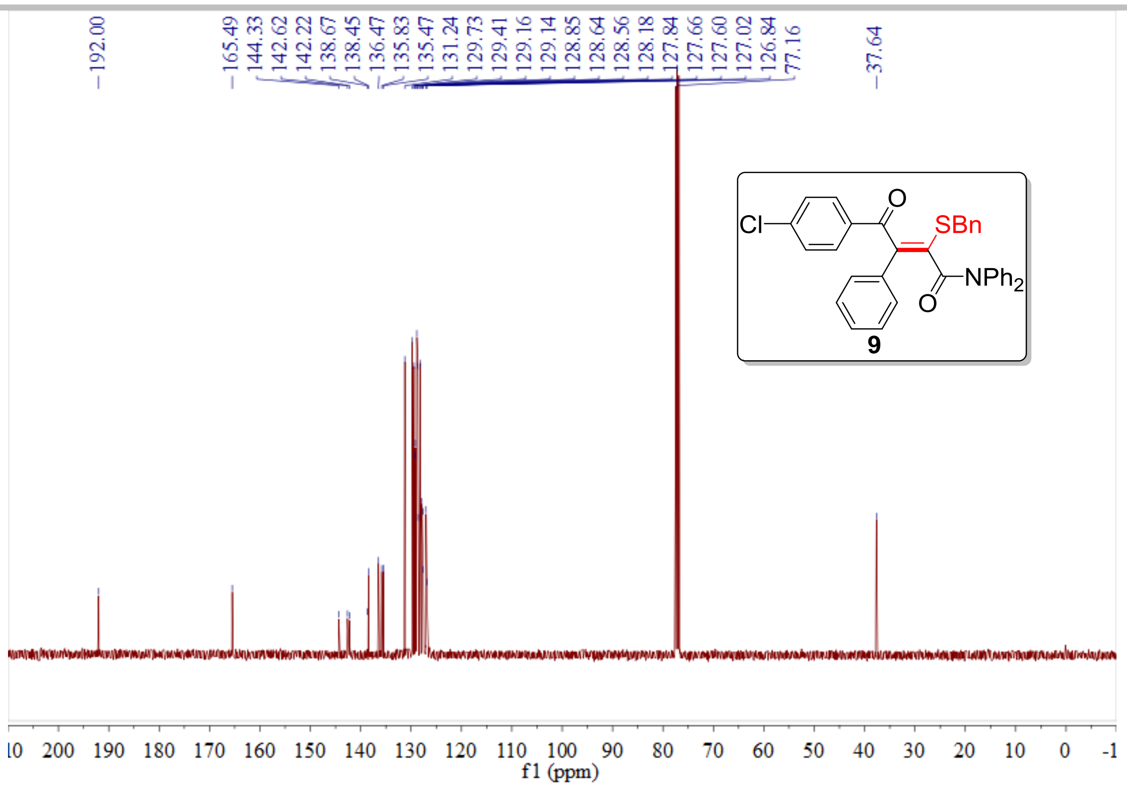

192.00
165.49
144.33
142.62
142.22
138.67
138.45
136.47
135.83
135.47
131.24
129.73
129.41
129.16
129.14
128.85
128.64
128.56
128.18
127.84
127.66
127.60
127.02
126.84
77.16
37.64
Cl
O
SBn
NPh2
O
9
f1 (ppm)

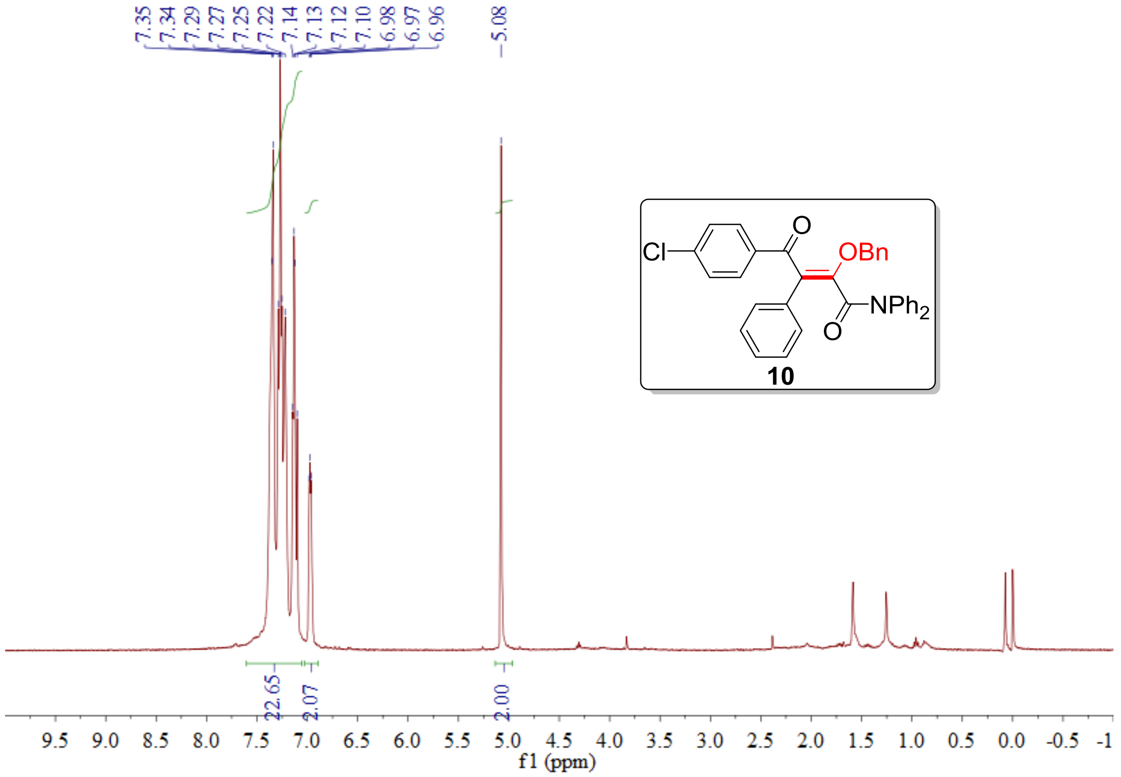

7.35
7.34
7.29
7.27
7.25
7.22
7.14
7.13
7.12
7.10
6.98
6.97
6.96
5.08
Cl
O
OBn
NPh2
O
10
22.65
2.07
2.00
f1 (ppm)

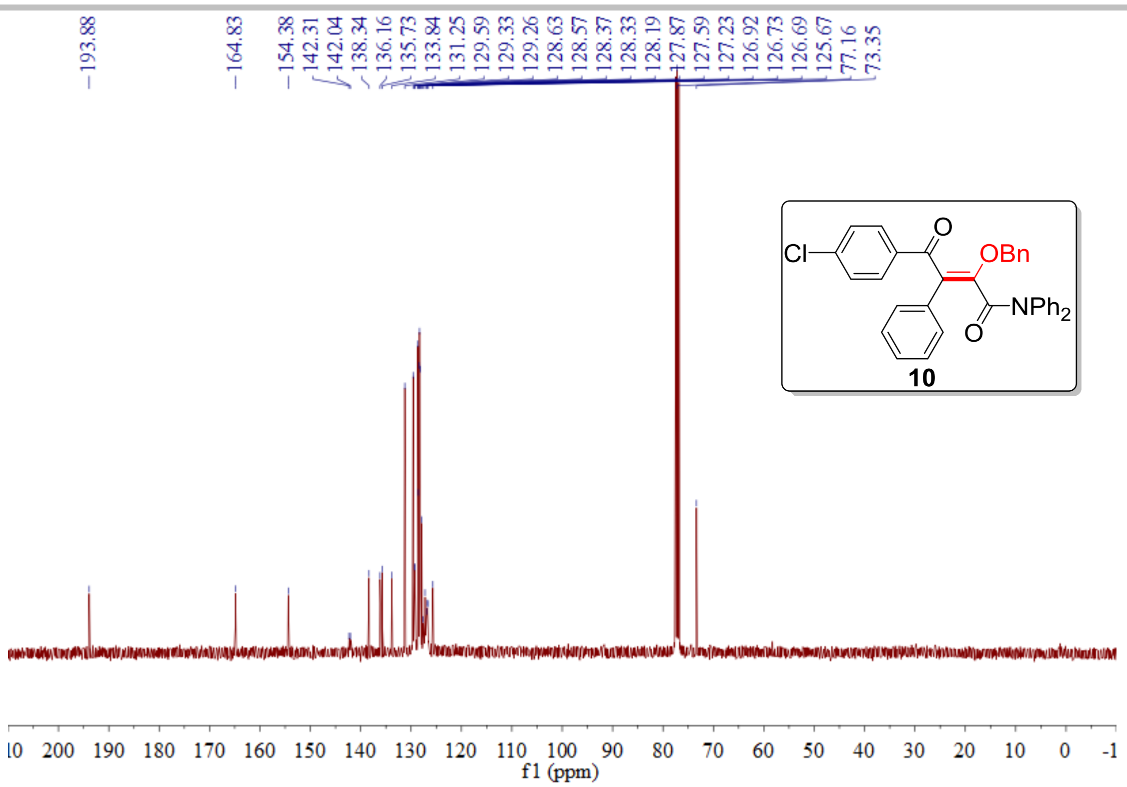
193.88
164.83
154.38
142.31
142.04
138.34
136.16
135.73
133.84
131.25
129.59
129.33
129.26
128.63
128.57
128.37
128.33
128.19
127.87
127.59
127.23
126.92
126.73
126.69
125.67
77.16
73.35
Cl
O
OBn
NPh2
O
10
f1 (ppm)

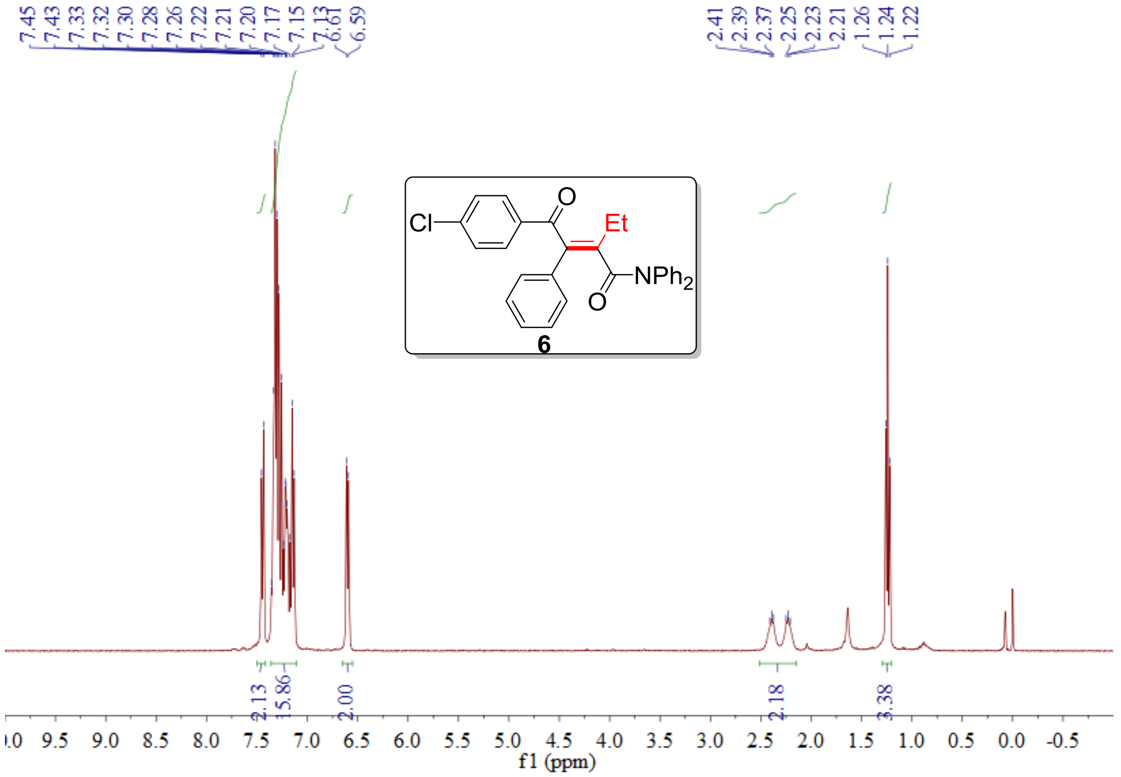
7.45
7.43
7.33
7.32
7.30
7.28
7.26
7.22
7.21
7.20
7.17
7.15
7.13
6.61
6.59
2.41
2.39
2.37
2.25
2.23
2.21
1.26
1.24
1.22
Cl
O
Et
NPh2
O
6
2.13
15.86
2.00
2.18
3.38
f1 (ppm)

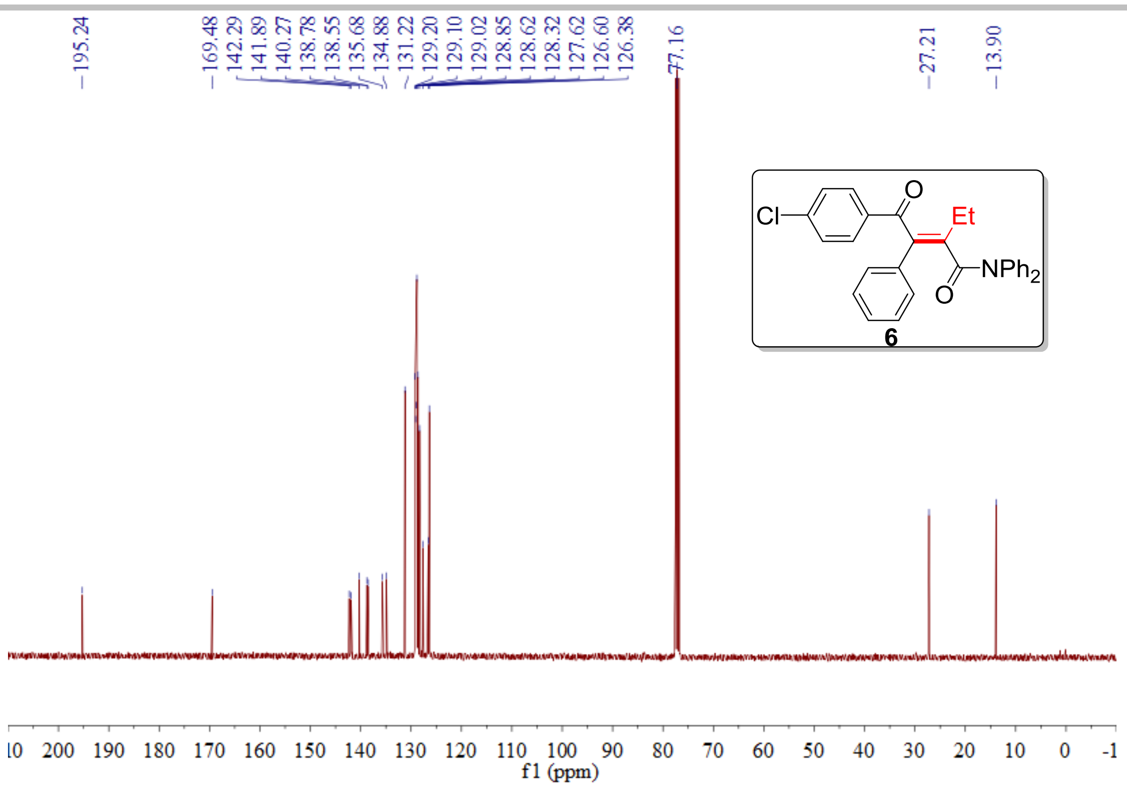
195.24
169.48
142.29
141.89
140.27
138.78
138.55
135.68
134.88
131.22
129.20
129.10
129.02
128.85
128.62
128.32
127.62
126.60
126.38
77.16
27.21
13.90
Cl
O
Et
NPh2
O
6
f1 (ppm)

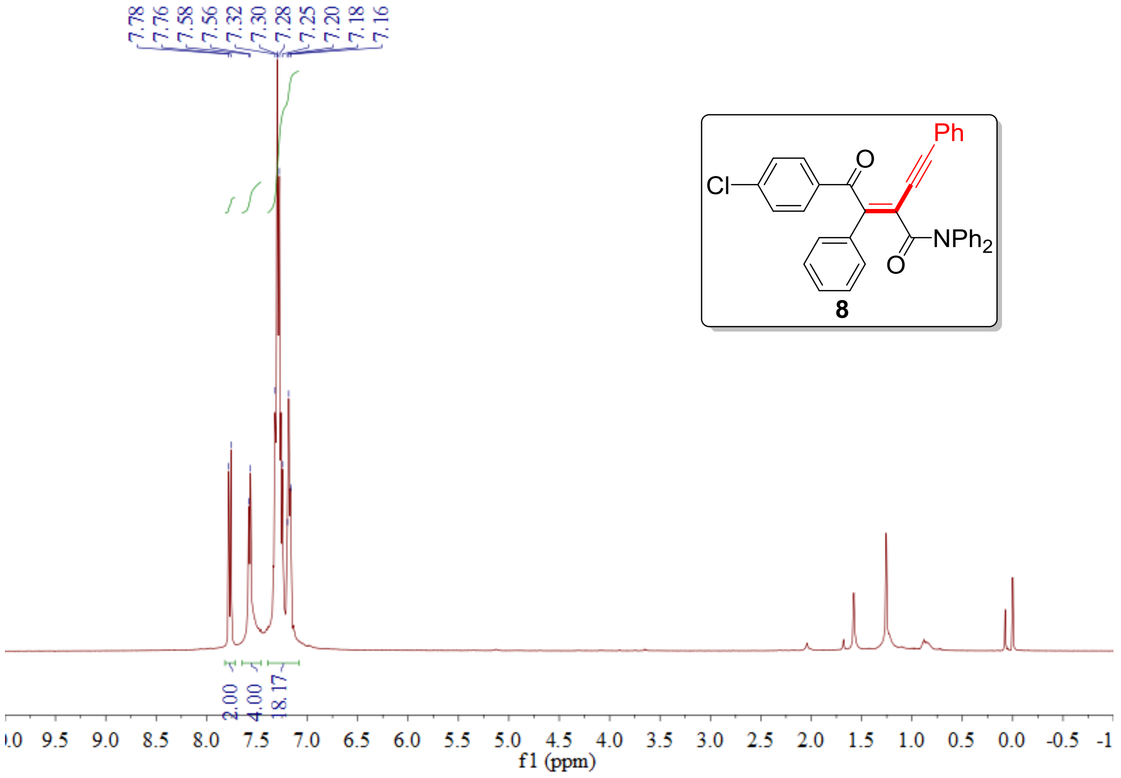
7.78
7.76
7.58
7.56
7.32
7.30
7.28
7.25
7.20
7.18
7.16
Ph
Cl
O
NPh2
O
8
2.00
4.00
18.17
f1 (ppm)

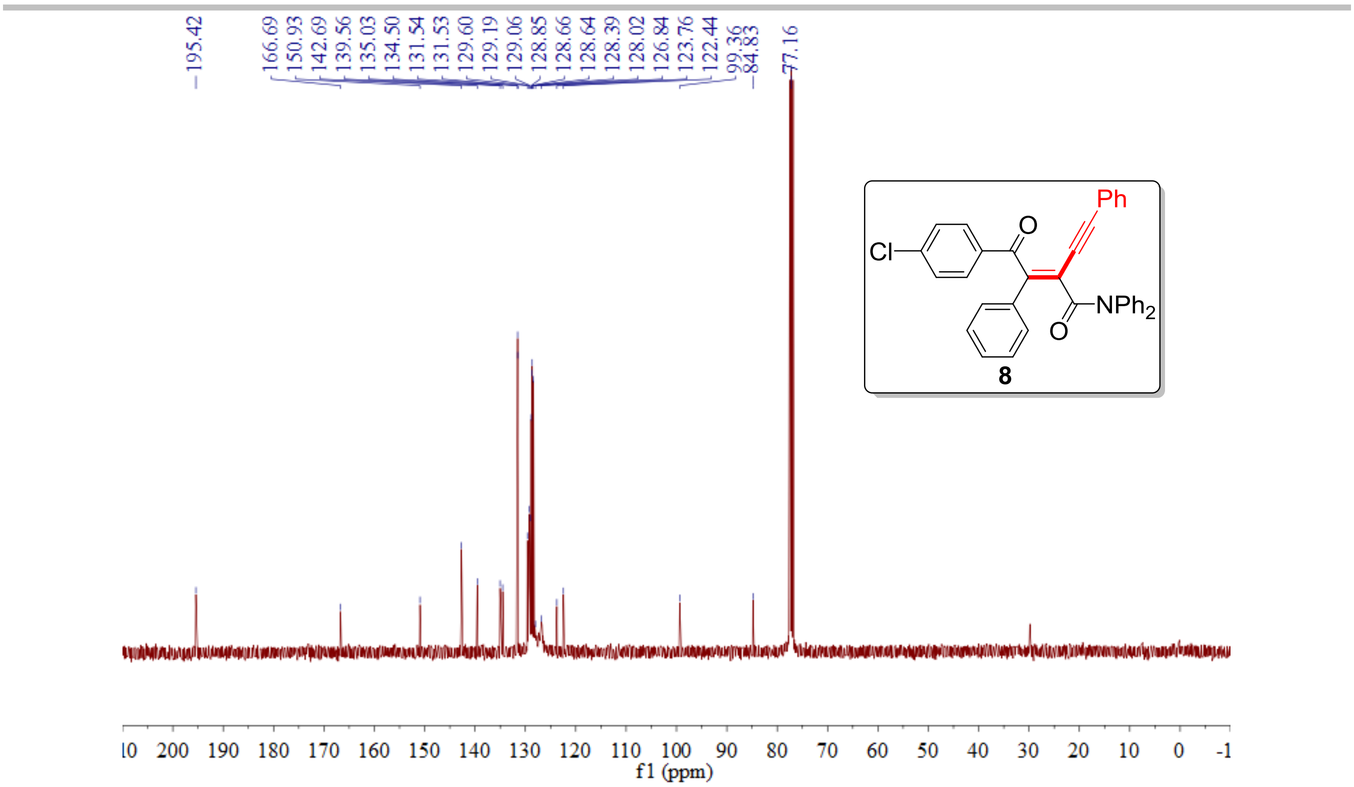
195.42
166.69
150.93
142.69
139.56
135.03
134.50
131.54
131.53
129.60
129.19
129.06
128.85
128.66
128.64
128.39
128.02
126.84
123.76
122.44
99.36
84.83
77.16
Cl
O
Ph
NPh2
O
8
f1 (ppm)

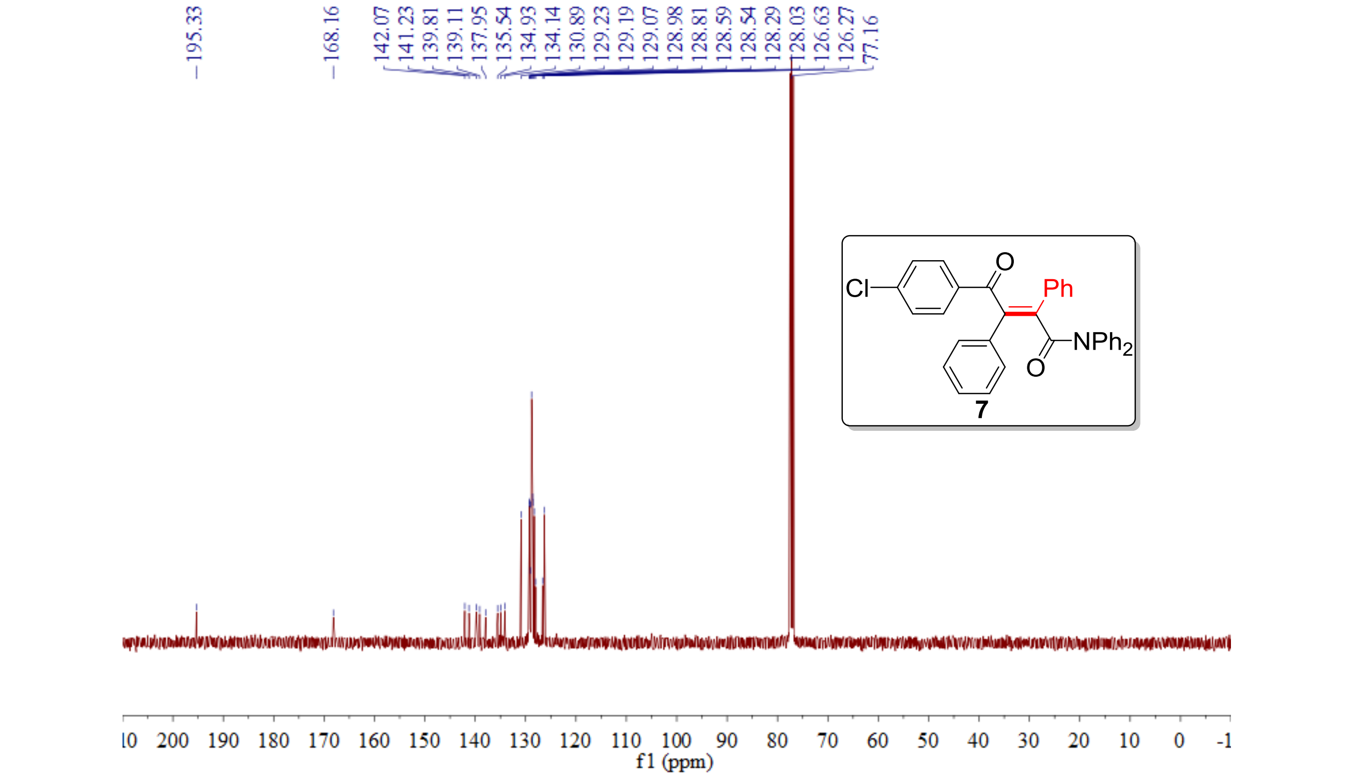
195.33
168.16
142.07
141.23
139.81
139.11
137.95
135.54
134.93
134.14
130.89
129.23
129.19
129.07
128.98
128.81
128.59
128.54
128.29
128.03
126.63
126.27
77.16
Cl
O
Ph
NPh2
O
7
f1 (ppm)

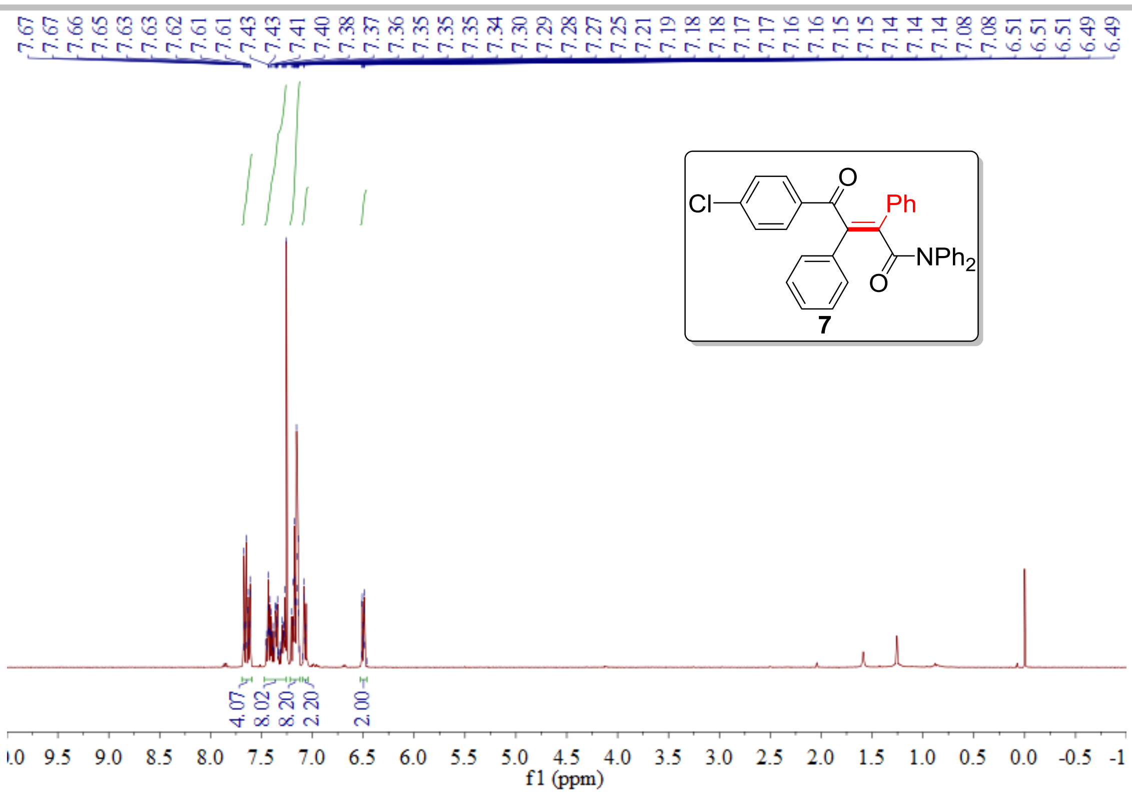
Cl
O
Ph
NPh2
O
7
f1 (ppm)

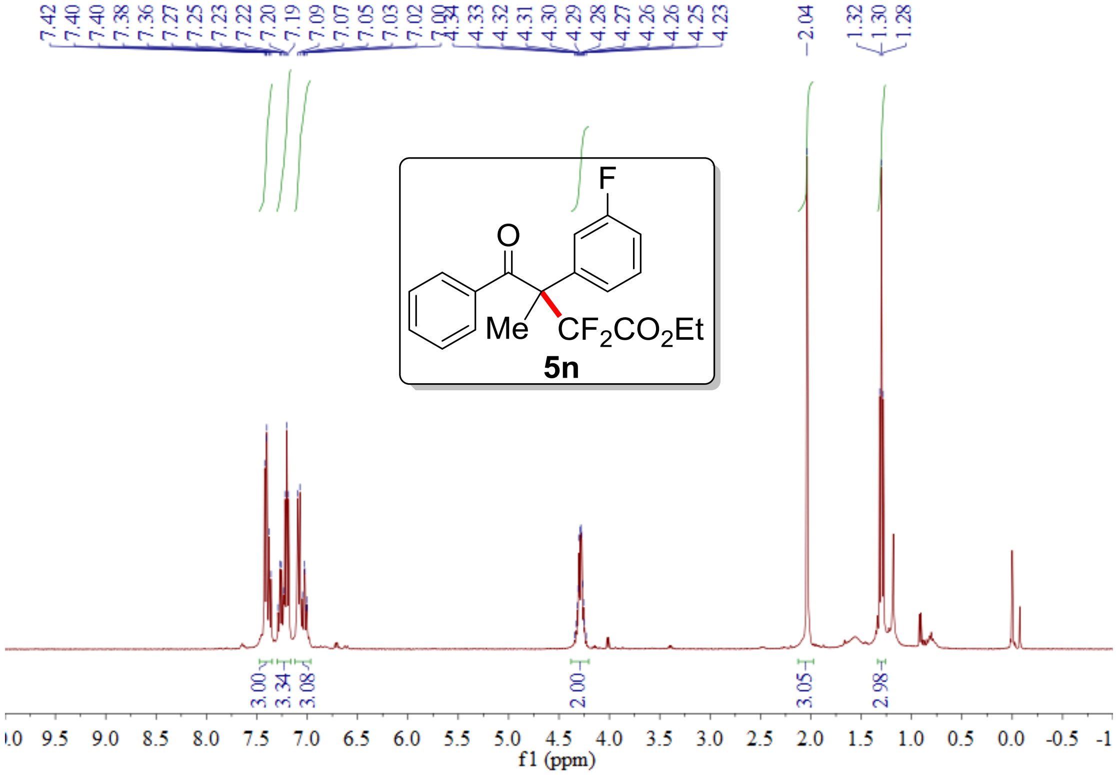
F
O
Me
CF2CO2Et
5n
f1 (ppm)

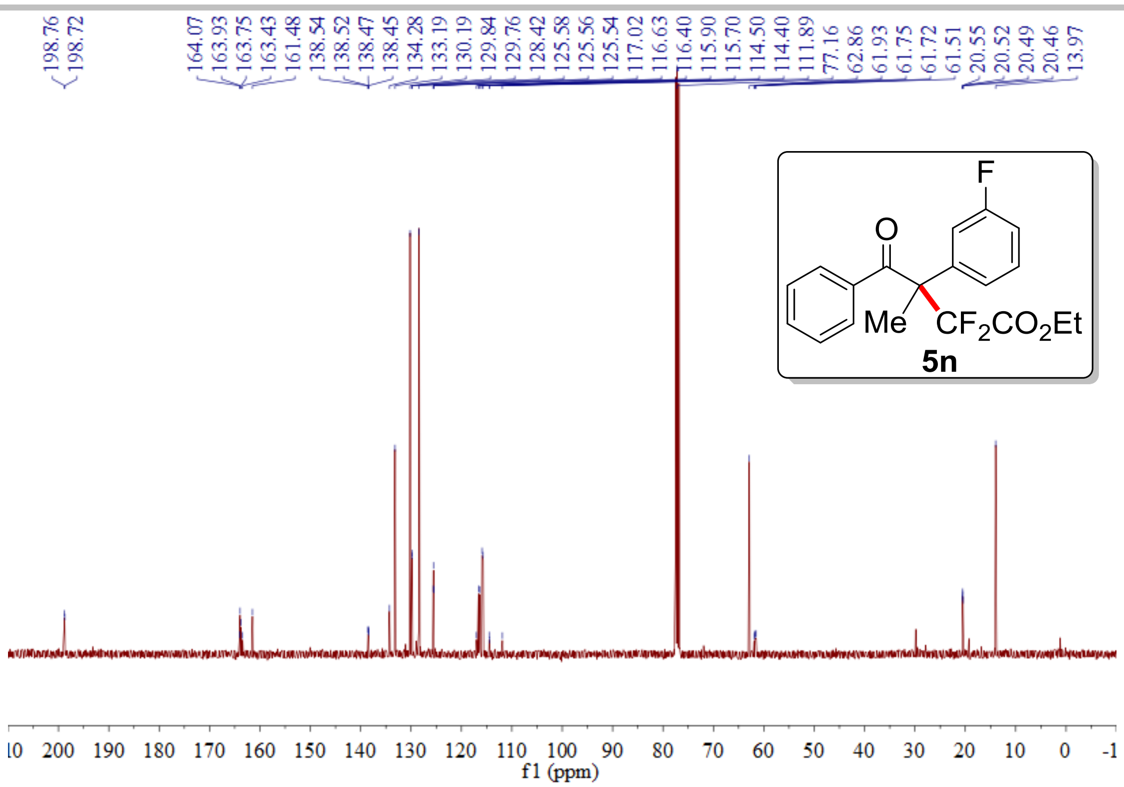
198.76
198.72
164.07
163.93
163.75
163.43
161.48
138.54
138.52
138.47
138.45
134.28
133.19
130.19
129.84
129.76
128.42
125.58
125.56
125.54
117.02
116.63
116.40
115.90
115.70
114.50
114.40
111.89
77.16
62.86
61.93
61.75
61.72
61.51
20.55
20.52
20.49
20.46
13.97
F
O
Me
CF_2CO_2Et
5n
f1 (ppm)

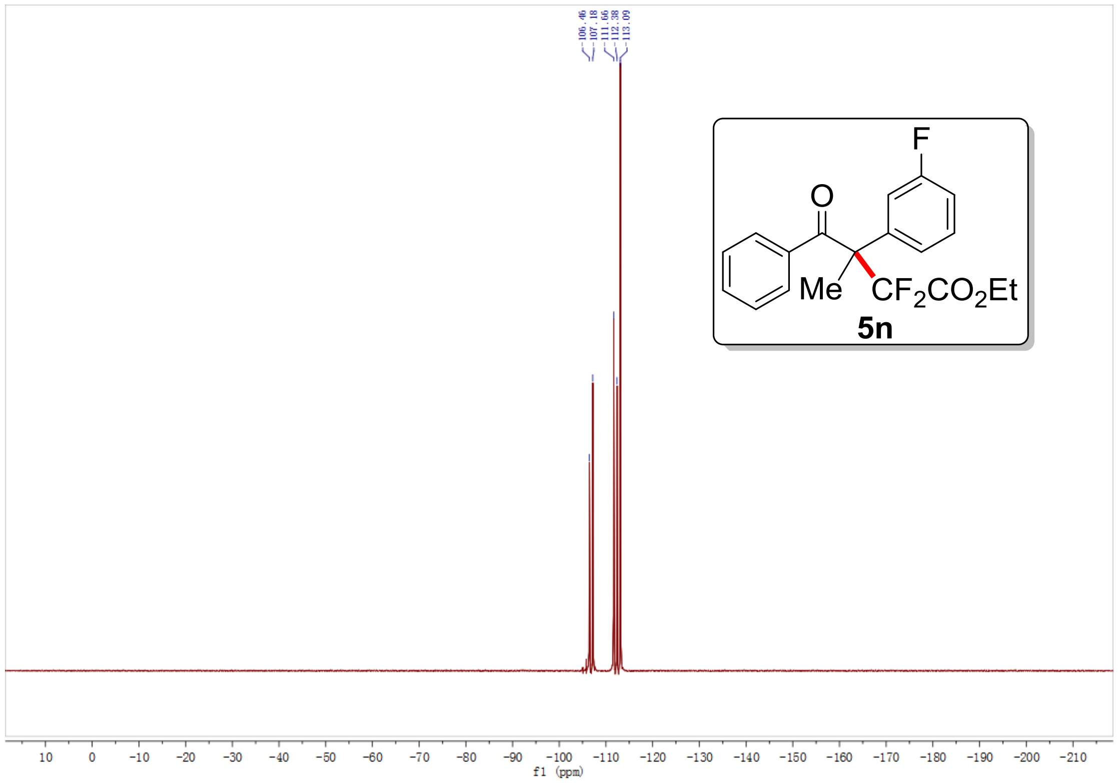
106.46
107.18
111.66
112.38
113.09
F
O
Me
CF_2CO_2Et
5n
f1 (ppm)

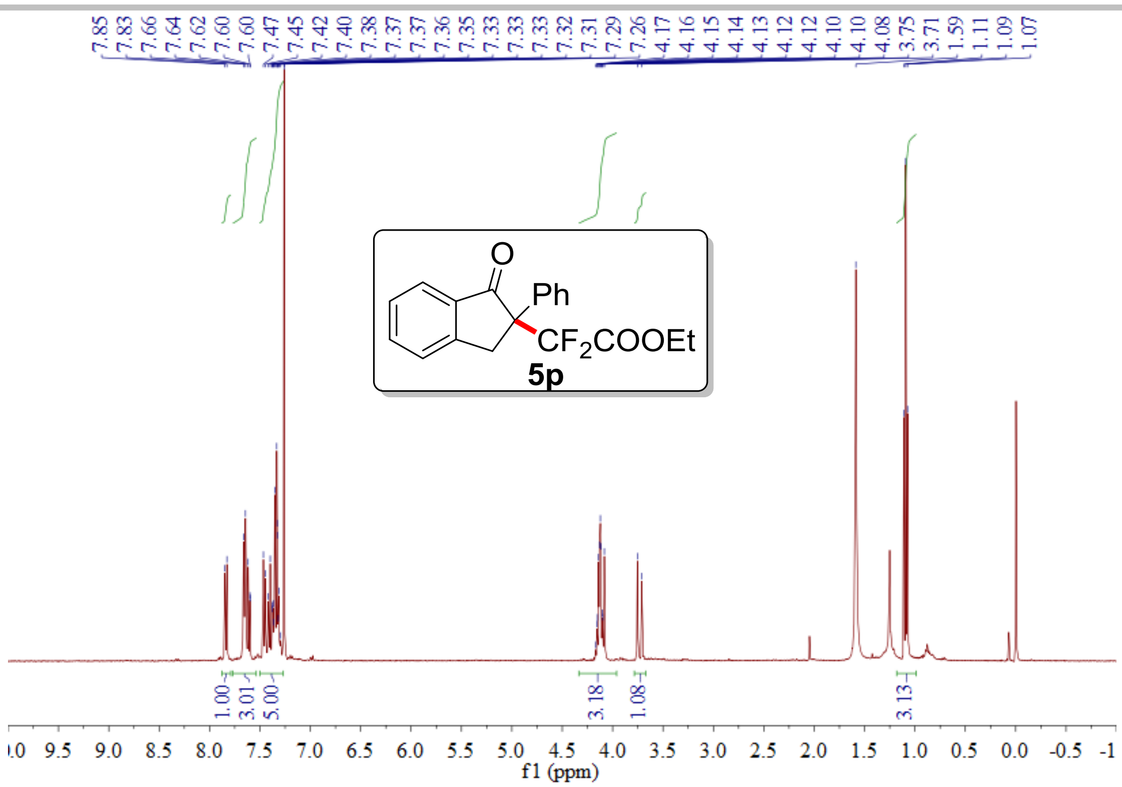
7.85
7.83
7.66
7.64
7.62
7.60
7.60
7.47
7.45
7.42
7.40
7.38
7.37
7.37
7.36
7.35
7.33
7.33
7.33
7.32
7.31
7.29
7.26
4.17
4.16
4.15
4.14
4.13
4.12
4.12
4.10
4.10
4.08
3.75
3.71
1.59
1.11
1.09
1.07
O
Ph
CF2COOEt
5p
1.00
3.01
5.00
3.18
1.08
3.13
f1 (ppm)

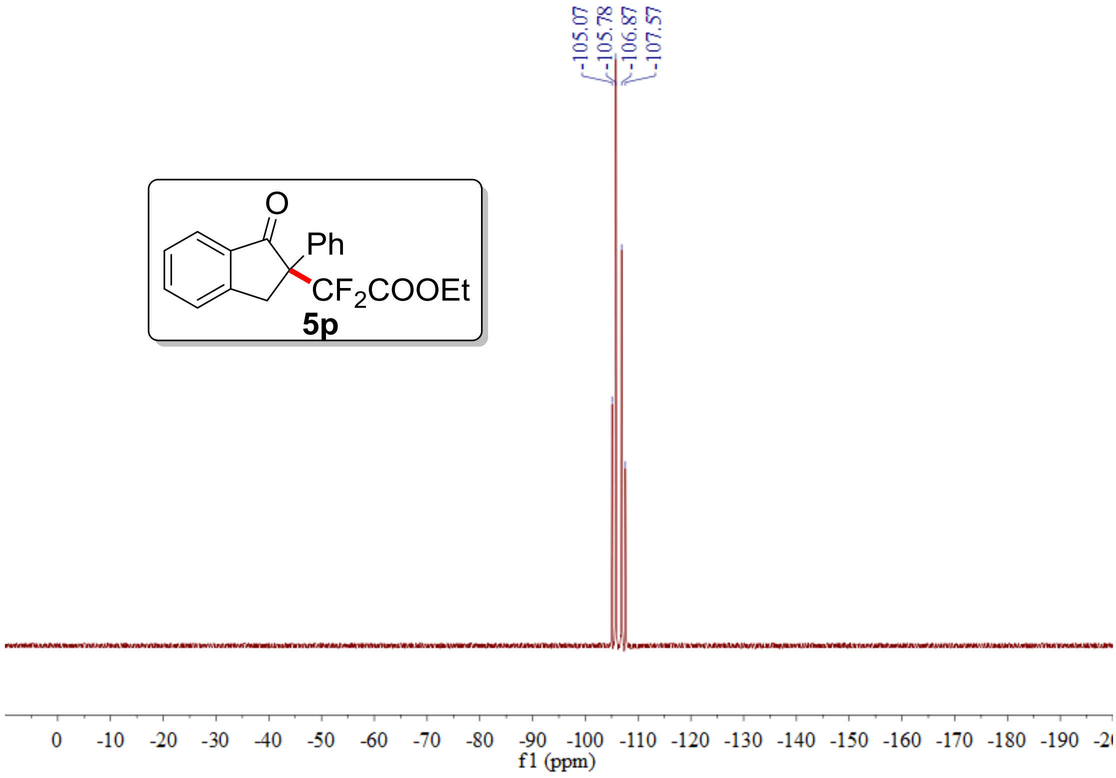
-105.07
-105.78
-106.87
-107.57
O
Ph
CF2COOEt
5p
f1 (ppm)

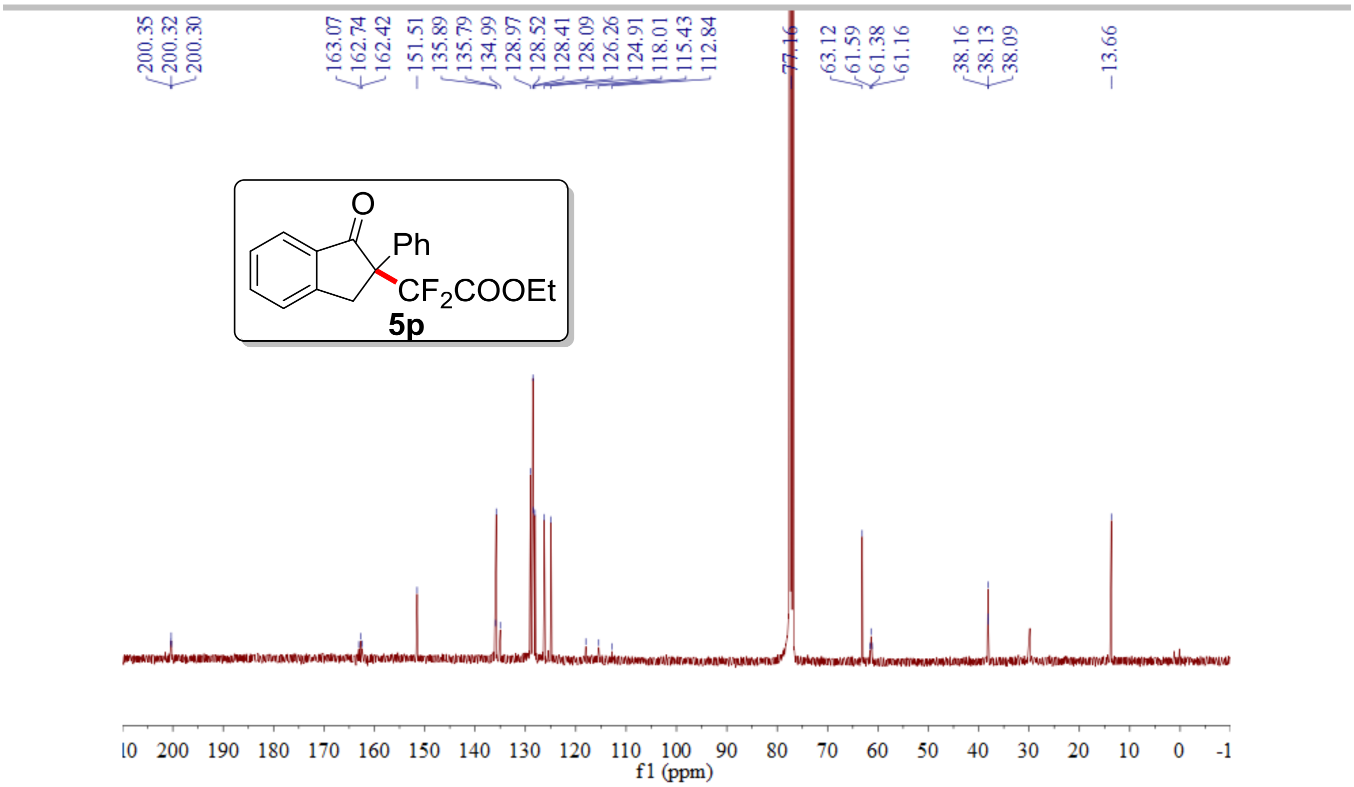
200.35
200.32
200.30
163.07
162.74
162.42
151.51
135.89
135.79
134.99
128.97
128.52
128.41
128.09
126.26
124.91
118.01
115.43
112.84
77.16
63.12
61.59
61.38
61.16
38.16
38.13
38.09
13.66
O
Ph
CF2COOEt
5p
f1 (ppm)

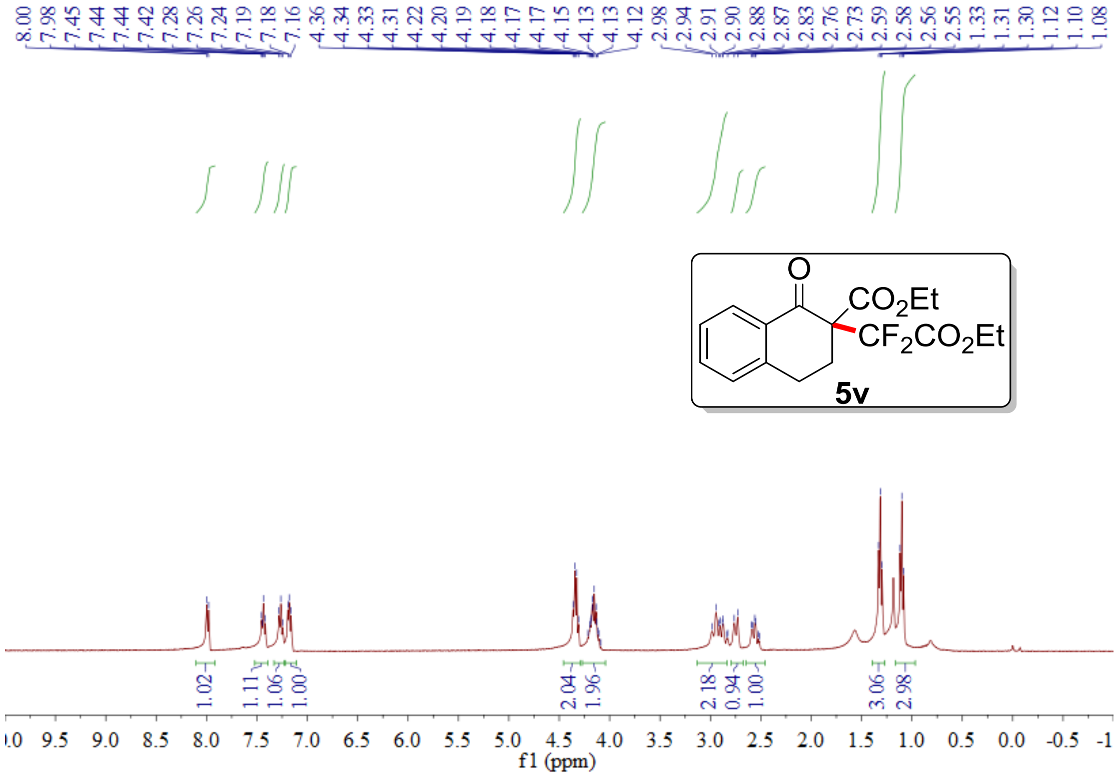
8.00
7.98
7.45
7.44
7.44
7.42
7.28
7.26
7.24
7.19
7.18
7.16
4.36
4.34
4.33
4.31
4.22
4.20
4.19
4.18
4.17
4.17
4.15
4.13
4.13
4.12
2.98
2.94
2.91
2.90
2.88
2.87
2.83
2.76
2.73
2.59
2.58
2.56
2.55
1.33
1.31
1.30
1.12
1.10
1.08
O
CO2Et
CF2CO2Et
5v
1.02
1.11
1.06
1.00
2.04
1.96
2.18
0.94
1.00
3.06
2.98
f1 (ppm)

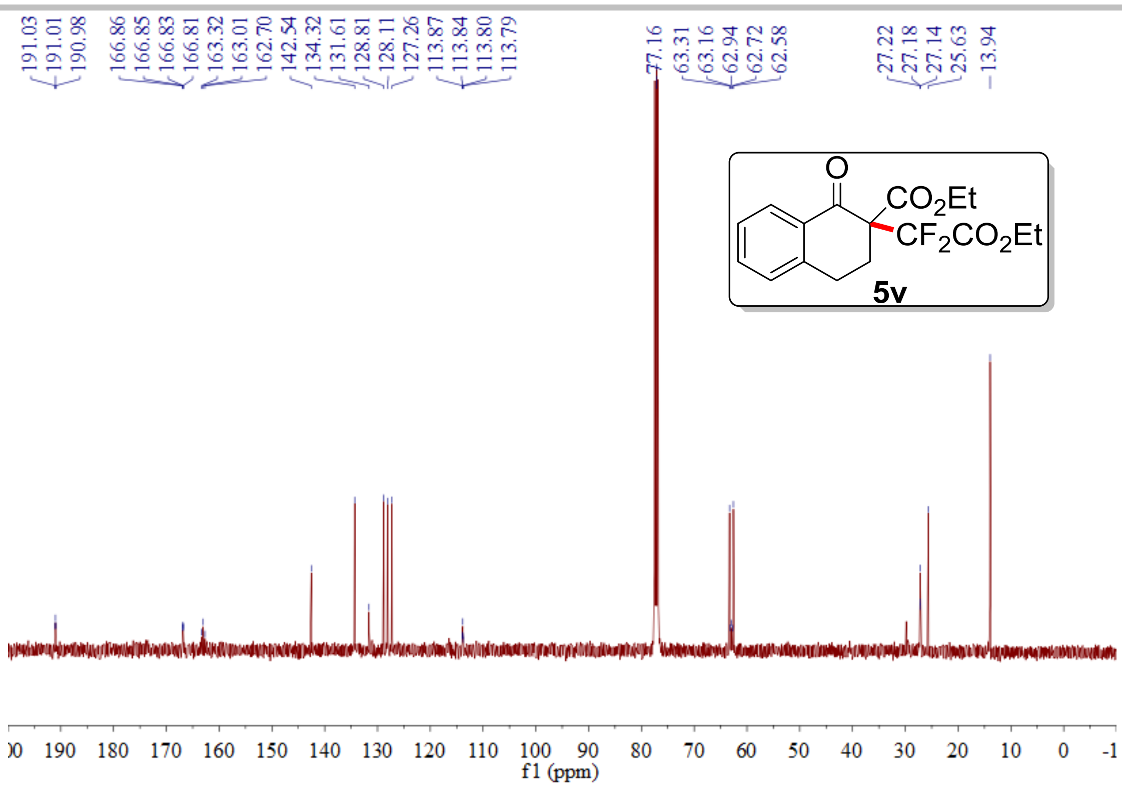

191.03
191.01
190.98
166.86
166.85
166.83
166.81
163.32
163.01
162.70
142.54
134.32
131.61
128.81
128.11
127.26
113.87
113.84
113.80
113.79
77.16
63.31
63.16
62.94
62.72
62.58
27.22
27.18
27.14
25.63
13.94
O
CO_2Et
CF_2CO_2Et
5v
f1 (ppm)

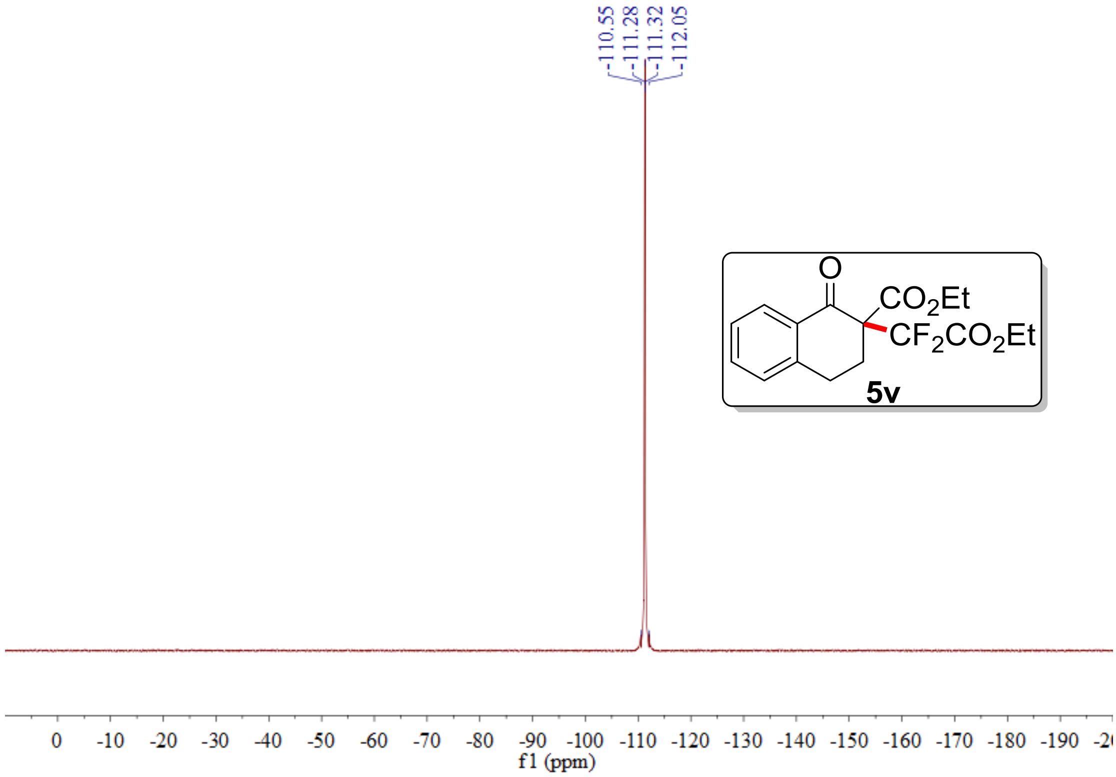

-110.55
-111.28
-111.32
-112.05
O
CO_2Et
CF_2CO_2Et
5v
f1 (ppm)

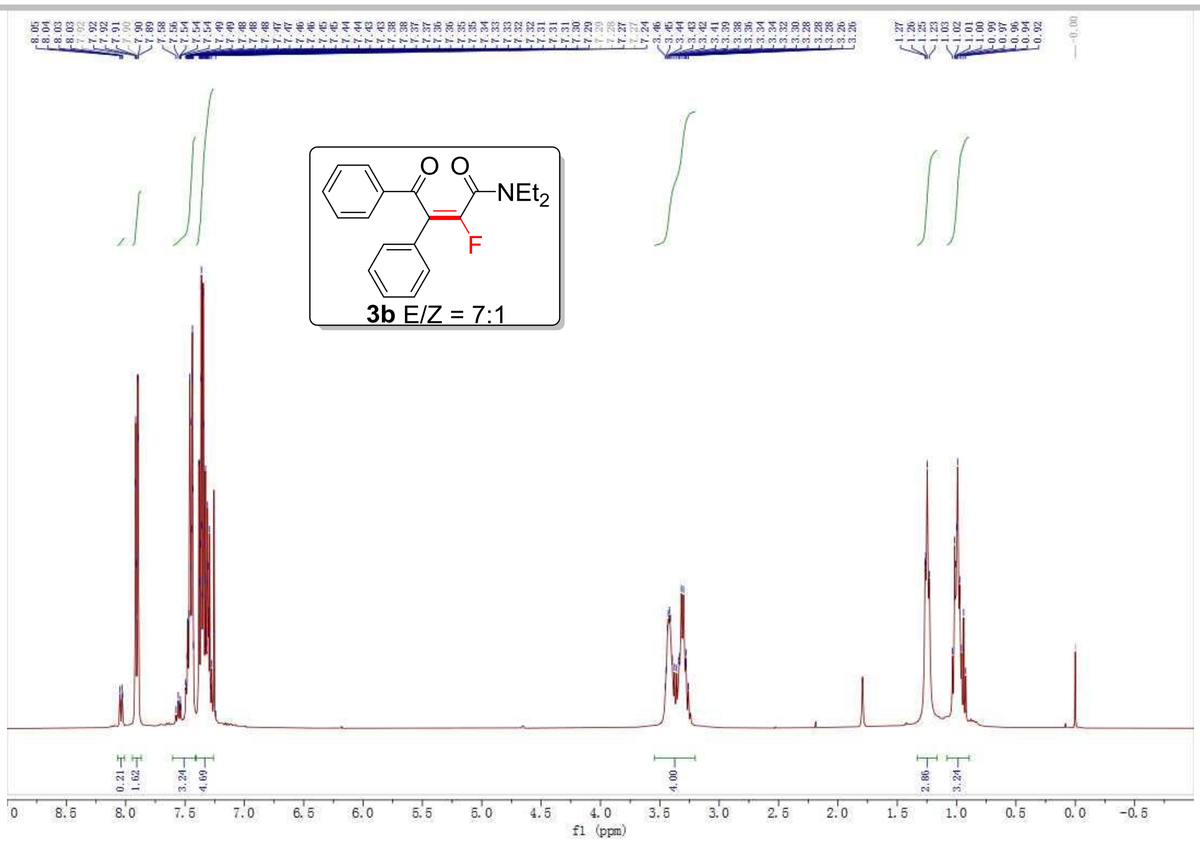
O O
NEt2
F
3b E/Z = 7:1
f1 (ppm)

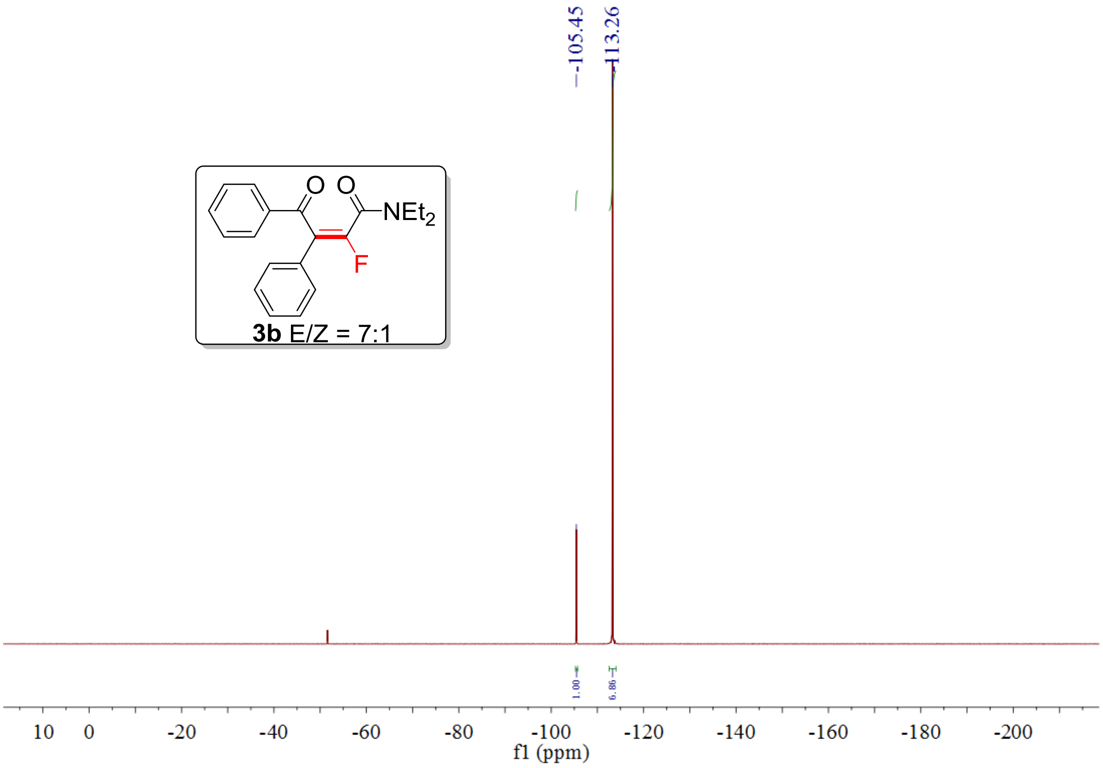
-105.45
-113.26
O O
NEt2
F
3b E/Z = 7:1
f1 (ppm)

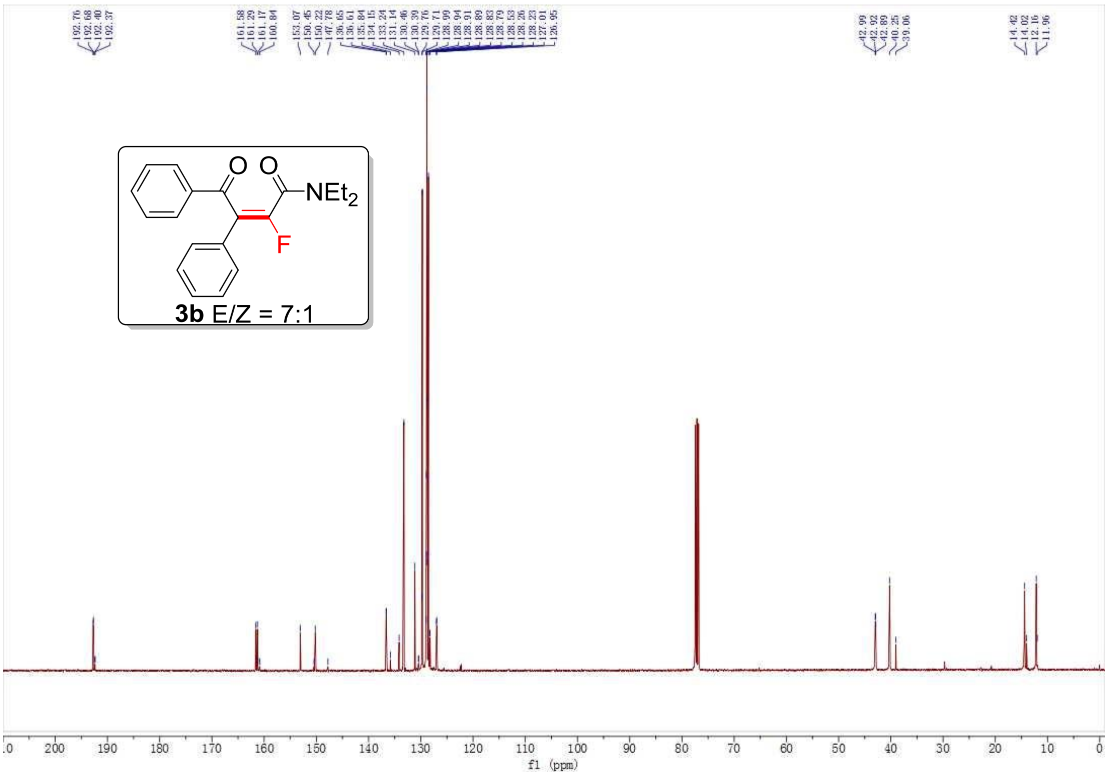
3b E/Z = 7:1
NEt2
F
f1 (ppm)
